# Mapping age- and sex-specific HIV prevalence in adults in sub-Saharan Africa, 2000–2018

**DOI:** 10.1186/s12916-022-02639-z

**Published:** 2022-12-19

**Authors:** Emily Haeuser, Audrey L. Serfes, Michael A. Cork, Mingyou Yang, Hedayat Abbastabar, E. S. Abhilash, Maryam Adabi, Oladimeji M. Adebayo, Victor Adekanmbi, Daniel Adedayo Adeyinka, Saira Afzal, Bright Opoku Ahinkorah, Keivan Ahmadi, Muktar Beshir Ahmed, Yonas Akalu, Rufus Olusola Akinyemi, Chisom Joyqueenet Akunna, Fares Alahdab, Fahad Mashhour Alanezi, Turki M. Alanzi, Kefyalew Addis Alene, Robert Kaba Alhassan, Vahid Alipour, Amir Almasi-Hashiani, Nelson Alvis-Guzman, Edward Kwabena Ameyaw, Saeed Amini, Dickson A. Amugsi, Robert Ancuceanu, Davood Anvari, Seth Christopher Yaw Appiah, Jalal Arabloo, Olatunde Aremu, Mulusew A. Asemahagn, Mohammad Asghari Jafarabadi, Atalel Fentahun Awedew, Beatriz Paulina Ayala Quintanilla, Martin Amogre Ayanore, Yared Asmare Aynalem, Samad Azari, Zelalem Nigussie Azene, B. B. Darshan, Tesleem Kayode Babalola, Atif Amin Baig, Maciej Banach, Till Winfried Bärnighausen, Arielle Wilder Bell, Akshaya Srikanth Bhagavathula, Nikha Bhardwaj, Pankaj Bhardwaj, Krittika Bhattacharyya, Ali Bijani, Zebenay Workneh Bitew, Somayeh Bohlouli, Obasanjo Afolabi Bolarinwa, Archith Boloor, Ivana Bozicevic, Zahid A. Butt, Rosario Cárdenas, Felix Carvalho, Jaykaran Charan, Vijay Kumar Chattu, Mohiuddin Ahsanul Kabir Chowdhury, Dinh-Toi Chu, Richard G. Cowden, Saad M. A. Dahlawi, Giovanni Damiani, Eugene Kofuor Maafo Darteh, Aso Mohammad Darwesh, José das Neves, Nicole Davis Weaver, Diego De Leo, Jan-Walter De Neve, Kebede Deribe, Keshab Deuba, Samath Dharmaratne, Mostafa Dianatinasab, Daniel Diaz, Alireza Didarloo, Shirin Djalalinia, Fariba Dorostkar, Eleonora Dubljanin, Bereket Duko, Maha El Tantawi, Shaimaa I. El-Jaafary, Babak Eshrati, Sharareh Eskandarieh, Oghenowede Eyawo, Ifeanyi Jude Ezeonwumelu, Sayeh Ezzikouri, Farshad Farzadfar, Nazir Fattahi, Nelsensius Klau Fauk, Eduarda Fernandes, Irina Filip, Florian Fischer, Nataliya A. Foigt, Masoud Foroutan, Takeshi Fukumoto, Mohamed M. Gad, Abhay Motiramji Gaidhane, Birhan Gebresillassie Gebregiorgis, Ketema Bizuwork Gebremedhin, Lemma Getacher, Keyghobad Ghadiri, Ahmad Ghashghaee, Mahaveer Golechha, Mohammed Ibrahim Mohialdeen Gubari, Harish Chander Gugnani, Rafael Alves Guimarães, Mohammad Rifat Haider, Arvin Haj-Mirzaian, Samer Hamidi, Abdiwahab Hashi, Soheil Hassanipour, Hadi Hassankhani, Khezar Hayat, Claudiu Herteliu, Hung Chak Ho, Ramesh Holla, Mostafa Hosseini, Mehdi Hosseinzadeh, Bing-Fang Hwang, Segun Emmanuel Ibitoye, Olayinka Stephen Ilesanmi, Irena M. Ilic, Milena D. Ilic, Rakibul M. Islam, Chidozie C. D. Iwu, Mihajlo Jakovljevic, Ravi Prakash Jha, John S. Ji, Kimberly B. Johnson, Nitin Joseph, Vasna Joshua, Farahnaz Joukar, Jacek Jerzy Jozwiak, Leila R. Kalankesh, Rohollah Kalhor, Naser Kamyari, Tanuj Kanchan, Behzad Karami Matin, Salah Eddin Karimi, Gbenga A. Kayode, Ali Kazemi Karyani, Maryam Keramati, Ejaz Ahmad Khan, Gulfaraz Khan, Md Nuruzzaman Khan, Khaled Khatab, Jagdish Khubchandani, Yun Jin Kim, Adnan Kisa, Sezer Kisa, Jacek A. Kopec, Soewarta Kosen, Sindhura Lakshmi Koulmane Laxminarayana, Ai Koyanagi, Kewal Krishan, Barthelemy Kuate Defo, Nuworza Kugbey, Vaman Kulkarni, Manasi Kumar, Nithin Kumar, Dian Kusuma, Carlo La Vecchia, Dharmesh Kumar Lal, Iván Landires, Heidi Jane Larson, Savita Lasrado, Paul H. Lee, Shanshan Li, Xuefeng Liu, Afshin Maleki, Preeti Malik, Mohammad Ali Mansournia, Francisco Rogerlândio Martins-Melo, Walter Mendoza, Ritesh G. Menezes, Endalkachew Worku Mengesha, Tuomo J. Meretoja, Tomislav Mestrovic, Andreea Mirica, Babak Moazen, Osama Mohamad, Yousef Mohammad, Abdollah Mohammadian-Hafshejani, Reza Mohammadpourhodki, Salahuddin Mohammed, Shafiu Mohammed, Ali H. Mokdad, Masoud Moradi, Paula Moraga, Sumaira Mubarik, Getaneh Baye B. Mulu, Lillian Mwanri, Ahamarshan Jayaraman Nagarajan, Mukhammad David Naimzada, Muhammad Naveed, Javad Nazari, Rawlance Ndejjo, Ionut Negoi, Frida N. Ngalesoni, Georges Nguefack-Tsague, Josephine W. Ngunjiri, Cuong Tat Nguyen, Huong Lan Thi Nguyen, Chukwudi A. Nnaji, Jean Jacques Noubiap, Virginia Nuñez-Samudio, Vincent Ebuka Nwatah, Bogdan Oancea, Oluwakemi Ololade Odukoya, Andrew T. Olagunju, Babayemi Oluwaseun Olakunde, Bolajoko Olubukunola Olusanya, Jacob Olusegun Olusanya, Ahmed Omar Bali, Obinna E. Onwujekwe, Orish Ebere Orisakwe, Nikita Otstavnov, Stanislav S. Otstavnov, Mayowa O. Owolabi, P. A. Mahesh, Jagadish Rao Padubidri, Adrian Pana, Ashok Pandey, Seithikurippu R. Pandi-Perumal, Fatemeh Pashazadeh Kan, George C. Patton, Shrikant Pawar, Emmanuel K. Peprah, Maarten J. Postma, Liliana Preotescu, Zahiruddin Quazi Syed, Navid Rabiee, Amir Radfar, Alireza Rafiei, Fakher Rahim, Vafa Rahimi-Movaghar, Amir Masoud Rahmani, Kiana Ramezanzadeh, Juwel Rana, Chhabi Lal Ranabhat, Sowmya J. Rao, David Laith Rawaf, Salman Rawaf, Reza Rawassizadeh, Lemma Demissie Regassa, Nima Rezaei, Aziz Rezapour, Mavra A. Riaz, Ana Isabel Ribeiro, Jennifer M. Ross, Enrico Rubagotti, Susan Fred Rumisha, Godfrey M. Rwegerera, Sahar Saeedi Moghaddam, Rajesh Sagar, Biniyam Sahiledengle, Maitreyi Sahu, Marwa Rashad Salem, Hossein Samadi Kafil, Abdallah M. Samy, Benn Sartorius, Brijesh Sathian, Abdul-Aziz Seidu, Amira A. Shaheen, Masood Ali Shaikh, Morteza Shamsizadeh, Wondimeneh Shibabaw Shiferaw, Jae Il Shin, Roman Shrestha, Jasvinder A. Singh, Valentin Yurievich Skryabin, Anna Aleksandrovna Skryabina, Shahin Soltani, Mu’awiyyah Babale Sufiyan, Takahiro Tabuchi, Eyayou Girma Tadesse, Nuno Taveira, Fisaha Haile Tesfay, Rekha Thapar, Marcos Roberto Tovani-Palone, Gebiyaw Wudie Tsegaye, Chukwuma David Umeokonkwo, Bhaskaran Unnikrishnan, Jorge Hugo Villafañe, Francesco S. Violante, Bay Vo, Giang Thu Vu, Yohannes Dibaba Wado, Yasir Waheed, Richard G. Wamai, Yanzhong Wang, Paul Ward, Nuwan Darshana Wickramasinghe, Katherine Wilson, Sanni Yaya, Paul Yip, Naohiro Yonemoto, Chuanhua Yu, Mikhail Sergeevich Zastrozhin, Yunquan Zhang, Zhi-Jiang Zhang, Simon I. Hay, Laura Dwyer-Lindgren, on behalf of Local Burden of Disease sub-Saharan Africa HIV Prevalence Collaborators

**Affiliations:** 1 Institute for Health Metrics and Evaluation, University of Washington, Seattle, WA USA; 2 Advanced Diagnostic and Interventional Radiology Research Center, Tehran University of Medical Sciences, Tehran, Iran; 3 Department of Botany, Sree Narayana Guru College Chelannur, Kozhikode, India; 4 Hamadan University of Medical Sciences, Hamadan, Iran; 5 College of Medicine, University College Hospital, Ibadan, Ibadan, Nigeria; 6 Department of Population Medicine, Cardiff University, Cardiff, UK; 7 Department of Community Health and Epidemiology, University of Saskatchewan, Saskatoon, SK Canada; 8 Department of Public Health, Federal Ministry of Health, Abuja, Nigeria; 9 Department of Community Medicine, King Edward Memorial Hospital, Lahore, Pakistan; 10 Department of Public Health, Public Health Institute, Lahore, Pakistan; 11 The Australian Centre for Public and Population Health Research (ACPPHR), University of Technology Sydney, Sydney, NSW Australia; 12 School of Public Health, Imperial College London, London, UK; 13 Department of Epidemiology, Jimma University, Jimma, Ethiopia; 14 Australian Center for Precision Health, University of South Australia, Adelaide, SA Australia; 15 Department of Medical Physiology, University of Gondar, Gondar, Ethiopia; 16 Institute for Advanced Medical Research and Training, University of Ibadan, Ibadan, Nigeria; 17 Institute of Neuroscience, Newcastle University, Newcastle upon Tyne, UK; 18 Department of Public Health, The Intercountry Centre for Oral Health (ICOH) for Africa, Jos, Nigeria; 19 Department of Public Health, Federal Ministry of Health, Garki, Nigeria; 20 Mayo Evidence-based Practice Center, Mayo Clinic Foundation for Medical Education and Research, Rochester, MN USA; 21 Imam Abdulrahman Bin Faisal University, Dammam, Saudi Arabia; 22 Health Information Management and Technology Department, Imam Abdulrahman Bin Faisal University, Dammam, Saudi Arabia; 23 Faculty of Health Sciences, Curtin University, Perth, WA Australia; 24 Wesfarmers Centre of Vaccines and Infectious Diseases, Telethon Kids Institute, Perth, WA Australia; 25 Institute of Health Research, University of Health and Allied Sciences, Ho, Ghana; 26 Health Management and Economics Research Center, Iran University of Medical Sciences, Tehran, Iran; 27 Department of Health Economics, Iran University of Medical Sciences, Tehran, Iran; 28 Department of Epidemiology, Arak University of Medical Sciences, Arak, Iran; 29 Research Group in Hospital Management and Health Policies, Universidad de la Costa (University of the Coast), Barranquilla, Colombia; 30 Research Group in Health Economics, University of Cartagena, Cartagena, Colombia; 31 Department of Health Services Management, Khomein University of Medical Sciences, Khomein, Iran; 32 Department of Maternal and Child Wellbeing, African Population and Health Research Center, Nairobi, Kenya; 33 Pharmacy Department, Carol Davila University of Medicine and Pharmacy, Bucharest, Romania; 34 Department of Parasitology, Mazandaran University of Medical Sciences, Sari, Iran; 35 Department of Parasitology, Iranshahr University of Medical Sciences, Iranshahr, Iran; 36 Department of Sociology and Social Work, Kwame Nkrumah University of Science and Technology, Kumasi, Ghana; 37 Center for International Health, Ludwig Maximilians University, Munich, Germany; 38 Department of Public Health, Birmingham City University, Birmingham, UK; 39 School of Public Health, Bahir Dar University, Bahir Dar, Ethiopia; 40 Department of Biostatistics and Epidemiology, Tabriz University of Medical Sciences, Tabriz, Iran; 41 Department of Biostatistics and Epidemiology, Zanjan University of Medical Sciences, Zanjan, Iran; 42 Department of Surgery, Addis Ababa University, Addis Ababa, Ethiopia; 43 The Judith Lumley Centre, La Trobe University, Melbourne, VIC Australia; 44 San Martin de Porres University, Lima, Peru; 45 Department of Health Policy Planning and Management, University of Health and Allied Sciences, Ho, Ghana; 46 Department of Health Economics, Centre for Health Policy Advocacy Innovation & Research in Africa (CHPAIR-Africa), Accra, Ghana; 47 Department of Nursing, Debre Berhan University, Debre Berhan, Ethiopia; 48 Hospital Management Research Center, Iran University of Medical Sciences, Tehran, Iran; 49 Department of Reproductive Health, University of Gondar, Gondar, Ethiopia; 50 Kasturba Medical College, Mangalore, Manipal Academy of Higher Education, Manipal, India; 51 Discipline of Public Health Medicine, University of KwaZulu-Natal, Durban, South Africa; 52 Department of Community Health and Primary Care, University of Lagos, Lagos, Nigeria; 53 Unit of Biochemistry, Universiti Sultan Zainal Abidin (Sultan Zainal Abidin University), Kuala Terengganu, Malaysia; 54 Department of Hypertension, Medical University of Lodz, Lodz, Poland; 55 Polish Mothers’ Memorial Hospital Research Institute, Lodz, Poland; 56 Heidelberg Institute of Global Health (HIGH), Heidelberg University, Heidelberg, Germany; 57 T.H. Chan School of Public Health, Harvard University, Boston, MA USA; 58 Department of Global Health and Social Medicine, Harvard University, Boston, MA USA; 59 Department of Social Services, Tufts Medical Center, Boston, MA USA; 60 Department of Social and Clinical Pharmacy, Charles University, Hradec Kralova, Czech Republic; 61 Institute of Public Health, United Arab Emirates University, Al Ain, United Arab Emirates; 62 Department of Anatomy, All India Institute of Medical Sciences, Jodhpur, India; 63 Department of Community Medicine and Family Medicine, All India Institute of Medical Sciences, Jodhpur, India; 64 School of Public Health, All India Institute of Medical Sciences, Jodhpur, India; 65 Department of Statistical and Computational Genomics, National Institute of Biomedical Genomics, Kalyani, India; 66 Department of Statistics, University of Calcutta, Kolkata, India; 67 Social Determinants of Health Research Center, Babol University of Medical Sciences, Babol, Iran; 68 Nutrition Department, St. Paul’s Hospital Millennium Medical College, Addis Ababa, Ethiopia; 69 St. Paul’s Hospital Millennium Medical College, Addis Ababa, Ethiopia; 70 Department of Veterinary Medicine, Islamic Azad University, Kermanshah, Iran; 71 Department of Internal Medicine, Manipal Academy of Higher Education, Mangalore, India; 72 WHO Collaborating Centre for HIV Strategic Information, University of Zagreb, Zagreb, Croatia; 73 Faculty of Public Health and Policy, London School of Hygiene & Tropical Medicine, London, UK; 74 School of Public Health and Health Systems, University of Waterloo, Waterloo, ON Canada; 75 Al Shifa School of Public Health, Al Shifa Trust Eye Hospital, Rawalpindi, Pakistan; 76 Department of Health Care, Metropolitan Autonomous University, Mexico City, Mexico; 77 Research Unit on Applied Molecular Biosciences (UCIBIO), University of Porto, Porto, Portugal; 78 Department of Pharmacology, All India Institute of Medical Sciences, Jodhpur, India; 79 Department of Community Medicine, Datta Meghe Institute of Medical Sciences, Sawangi, India; 80 Saveetha Medical College, Saveetha University, Chennai, India; 81 James P Grant School of Public Health, BRAC University, Dhaka, Bangladesh; 82 Department of Epidemiology and Biostatistics, University of South Carolina, Columbia, SC USA; 83 Center for Biomedicine and Community Health, VNU-International School, Hanoi, Vietnam; 84 Department of Psychology, University of the Free State, Park West, South Africa; 85 Environmental Health Department, Imam Abdulrahman Bin Faisal University, Dammam, Saudi Arabia; 86 IRCCS Istituto Ortopedico Galeazzi (Galeazzi Orthopedic Institute IRCCS), University of Milan, Milan, Italy; 87 Department of Dermatology, Case Western Reserve University, Cleveland, OH USA; 88 Department of Population and Health, University of Cape Coast, Cape Coast, Ghana; 89 Department of Information Technology, University of Human Development, Sulaymaniyah, Iraq; 90 Institute for Research and Innovation in Health, University of Porto, Porto, Portugal; 91 Institute of Biomedical Engineering (INEB), University of Porto, Porto, Portugal; 92 Australian Institute for Suicide Research and Prevention, Griffith University, Mount Gravatt, QLD Australia; 93 Wellcome Trust Brighton and Sussex Centre for Global Health Research, Brighton and Sussex Medical School, Brighton, UK; 94 School of Public Health, Addis Ababa University, Addis Ababa, Ethiopia; 95 National Centre for AIDS and STD Control, Save the Children, Kathmandu, Nepal; 96 Department of Global Public Health, Karolinska Institute, Stockholm, Sweden; 97 Department of Community Medicine, University of Peradeniya, Peradeniya, Sri Lanka; 98 Department of Health Metrics Sciences, School of Medicine, University of Washington, Seattle, WA USA; 99 Department of Epidemiology, Maastricht University, Maastricht, Netherlands; 100 Department of Epidemiology, Shiraz University of Medical Sciences, Shiraz, Iran; 101 Center of Complexity Sciences, National Autonomous University of Mexico, Mexico City, Mexico; 102 Faculty of Veterinary Medicine and Zootechnics, Autonomous University of Sinaloa, Rosales, Culiacán, Mexico; 103 Department of Community Medicine and Public Health, Urmia University of Medical Science, Urmia, Iran; 104 Development of Research and Technology Center, Ministry of Health and Medical Education, Tehran, Iran; 105 Department of Medical Laboratory Sciences, Iran University of Medical Sciences, Tehran, Iran; 106 Institute of Microbiology and Immunology, University of Belgrade, Belgrade, Serbia; 107 School of Public Health, Hawassa University, Hawassa, Ethiopia; 108 School of Public Health, Curtin University, Perth, WA Australia; 109 Pediatric Dentistry and Dental Public Health Department, Alexandria University, Alexandria, Egypt; 110 Department of Neurology, Cairo University, Cairo, Egypt; 111 Preventive Medicine and Public Health Research Center, Iran University of Medical Sciences, Tehran, Iran; 112 Multiple Sclerosis Research Center, Tehran University of Medical Sciences, Tehran, Iran; 113 Faculty of Health, York University, Toronto, BC Canada; 114 Institute for Health Science Research Germans Trias i Pujol, Autonomous University of Barcelona, Badalona, Spain; 115 IrsiCaixa AIDS Research Institute, Badalona, Spain; 116 Department of Virology, Pasteur Institute of Morocco, Casablanca, Morocco; 117 Non-communicable Diseases Research Center, Tehran University of Medical Sciences, Tehran, Iran; 118 Research Center for Environmental Determinants of Health, Kermanshah University of Medical Sciences, Kermanshah, Iran; 119 Torrens University Australia, Adelaide, SA Australia; 120 Institute of Resource Governance and Social Change, Kupang, Indonesia; 121 Associated Laboratory for Green Chemistry (LAQV), University of Porto, Porto, Portugal; 122 Psychiatry Department, Kaiser Permanente, Fontana, CA USA; 123 School of Health Sciences, A.T. Still University, Mesa, AZ USA; 124 Institute of Public Health, Charité Universitätsmedizin Berlin (Charité Medical University Berlin), Berlin, Germany; 125 Institute of Gerontology, National Academy of Medical Sciences of Ukraine, Kyiv, Ukraine; 126 Department of Medical Parasitology, Abadan University of Medical Sciences, Abadan, Iran; 127 Faculty of Medicine, Abadan University of Medical Sciences, Abadan, Iran; 128 Department of Dermatology, Kobe University, Kobe, Japan; 129 Department of Cardiovascular Medicine, Cleveland Clinic, Cleveland, OH USA; 130 Gillings School of Global Public Health, University of North Carolina Chapel Hill, Chapel Hill, NC USA; 131 Department of Community Medicine, Datta Meghe Institute of Medical Sciences, Wardha, India; 132 Department of Nursing and Midwifery, Addis Ababa University, Addis Ababa, Ethiopia; 133 Department of Public Health, Debre Berhan University, Debre Berhan, Ethiopia; 134 Infectious Disease Research Center, Kermanshah University of Medical Sciences, Kermanshah, Iran; 135 Pediatric Department, Kermanshah University of Medical Sciences, Kermanshah, Iran; 136 School of Public Health, Qazvin University of Medical Sciences, Qazvin, Iran; 137 Health Systems and Policy Research, Indian Institute of Public Health, Gandhinagar, India; 138 Department of Family and Community Medicine, University Of Sulaimani, Sulaimani, Iraq; 139 Department of Microbiology, Saint James School of Medicine, The Valley, Anguilla; 140 Department of Epidemiology, Saint James School of Medicine, The Valley, Anguilla; 141 Faculty of Nursing, Federal University of Goiás, Goiânia, Brazil; 142 Department of Health Policy and Management, University of Georgia, Athens, GA USA; 143 Department of Pharmacology, Shahid Beheshti University of Medical Sciences, Tehran, Iran; 144 Obesity Research Center, Shahid Beheshti University of Medical Sciences, Tehran, Iran; 145 School of Health and Environmental Studies, Hamdan Bin Mohammed Smart University, Dubai, United Arab Emirates; 146 Department of Public Health, Jigjiga University, Jijiga, Ethiopia; 147 Gastrointestinal and Liver Diseases Research Center, Guilan University of Medical Sciences, Rasht, Iran; 148 Caspian Digestive Disease Research Center, Guilan University of Medical Sciences, Rasht, Iran; 149 School of Nursing and Midwifery, Tabriz University of Medical Sciences, Tabriz, Iran; 150 Independent Consultant, Tabriz, Iran; 151 Institute of Pharmaceutical Sciences, University of Veterinary and Animal Sciences, Lahore, Pakistan; 152 Department of Pharmacy Administration and Clinical Pharmacy, Xian Jiaotong University, Xian, China; 153 Department of Statistics and Econometrics, Bucharest University of Economic Studies, Bucharest, Romania; 154 School of Business, London South Bank University, London, UK; 155 Department of Urban Planning and Design, University of Hong Kong, Hong Kong, China; 156 Department of Epidemiology and Biostatistics, Tehran University of Medical Sciences, Tehran, Iran; 157 Pediatric Chronic Kidney Disease Research Center, Tehran University of Medical Sciences, Tehran, Iran; 158 Institute of Research and Development, Duy Tan University, Da Nang, Vietnam; 159 Department of Computer Science, University of Human Development, Sulaymaniyah, Iraq; 160 Department of Occupational Safety and Health, China Medical University, Taichung, Taiwan; 161 Department of Health Promotion and Education, University of Ibadan, Ibadan, Nigeria; 162 Department of Community Medicine, University of Ibadan, Ibadan, Nigeria; 163 Department of Community Medicine, University College Hospital, Ibadan, Ibadan, Nigeria; 164 Faculty of Medicine, University of Belgrade, Belgrade, Serbia; 165 Department of Epidemiology, University of Kragujevac, Kragujevac, Serbia; 166 Department of Epidemiology and Preventive Medicine, Monash University, Melbourne, VIC Australia; 167 School of Health Systems and Public Health, University of Pretoria, Pretoria, South Africa; 168 Institute of Advanced Manufacturing Technologies, Peter the Great St. Petersburg Polytechnic University, St. Petersburg, Russia; 169 Institute of Comparative Economic Studies, Hosei University, Tokyo, Japan; 170 Department of Community Medicine, Dr. Baba Saheb Ambedkar Medical College & Hospital, Delhi, India; 171 Department of Community Medicine, Banaras Hindu University, Varanasi, India; 172 Vanke School of Public Health, Tsinghua University, Beijing, China; 173 Department of Community Medicine, Manipal Academy of Higher Education, Mangalore, India; 174 National Institute of Epidemiology, Indian Council of Medical Research, Chennai, India; 175 Department of Family Medicine and Public Health, University of Opole, Opole, Poland; 176 School of Management and Medical Informatics, Tabriz University of Medical Sciences, Tabriz, Iran; 177 Institute for Prevention of Non-communicable Diseases, Qazvin University of Medical Sciences, Qazvin, Iran; 178 Health Services Management Department, Qazvin University of Medical Sciences, Qazvin, Iran; 179 Department of Biostatistics, Abadan University of Medical Sciences, Abadan, Iran; 180 Department of Forensic Medicine and Toxicology, All India Institute of Medical Sciences, Jodhpur, India; 181 Social Determinants of Health Research Center, Tabriz University of Medical Sciences, Tabriz, Iran; 182 International Research Center of Excellence, Institute of Human Virology Nigeria, Abuja, Nigeria; 183 Julius Centre for Health Sciences and Primary Care, Utrecht University, Utrecht, Netherlands; 184 Mashhad University of Medical Sciences, Mashhad, Iran; 185 Department of Epidemiology and Biostatistics, Health Services Academy, Islamabad, Pakistan; 186 Department of Medical Microbiology & Immunology, United Arab Emirates University, Al Ain, United Arab Emirates; 187 Department of Population Science, Jatiya Kabi Kazi Nazrul Islam University, Mymensingh, Bangladesh; 188 Faculty of Health and Wellbeing, Sheffield Hallam University, Sheffield, UK; 189 College of Arts and Sciences, Ohio University, Zanesville, OH USA; 190 Department of Public Health, New Mexico State University, Las Cruces, NM USA; 191 School of Traditional Chinese Medicine, Xiamen University Malaysia, Sepang, Malaysia; 192 School of Health Sciences, Kristiania University College, Oslo, Norway; 193 Department of Global Community Health and Behavioral Sciences, Tulane University, New Orleans, LA USA; 194 Department of Nursing and Health Promotion, Oslo Metropolitan University, Oslo, Norway; 195 School of Population and Public Health, University of British Columbia, Vancouver, BC Canada; 196 Arthritis Research Canada, Richmond, Canada; 197 Independent Consultant, Jakarta, Indonesia; 198 Kasturba Medical College, Udupi, India; 199 Biomedical Research Networking Center for Mental Health Network (CIBERSAM), San Juan de Dios Sanitary Park, Sant Boi de Llobregat, Spain; 200 Catalan Institution for Research and Advanced Studies (ICREA), Barcelona, Spain; 201 Department of Anthropology, Panjab University, Chandigarh, India; 202 Department of Demography, University of Montreal, Montreal, QC Canada; 203 Department of Social and Preventive Medicine, University of Montreal, Montreal, Canada; 204 University of Environment and Sustainable Development, Somanya, Ghana; 205 Department of Psychiatry, University of Nairobi, Nairobi, Kenya; 206 Division of Psychology and Language Sciences, University College London, London, UK; 207 Imperial College Business School, Imperial College London, London, UK; 208 Faculty of Public Health, University of Indonesia, Depok, Indonesia; 209 Department of Clinical Sciences and Community Health, University of Milan, Milan, Italy; 210 Public Health Foundation of India, Gurugram, India; 211 Unit of Genetics and Public Health, Institute of Medical Sciences, Las Tablas, Panama; 212 Ministry of Health, Herrera, Panama; 213 Department of Infectious Disease Epidemiology, London School of Hygiene & Tropical Medicine, London, UK; 214 Department of Otorhinolaryngology, Father Muller Medical College, Mangalore, India; 215 Department of Health Sciences, University of Leicester, Leicester, UK; 216 School of Public Health and Preventive Medicine, Monash University, Melbourne, VIC Australia; 217 Lerner Research Institute, Cleveland Clinic, Cleveland, OH USA; 218 Department of Quantitative Health Science, Case Western Reserve University, Cleveland, OH USA; 219 Department of Environmental Health Engineering, Tehran University of Medical Sciences, Tehran, Iran; 220 Environmental Health Research Center, Kurdistan University of Medical Sciences, Sanandaj, Iran; 221 Department of Pediatrics, Montefiore Medical Center, New York, NY USA; 222 Department of Environmental Medicine and Public Health, Icahn School of Medicine at Mount Sinai, New York, NY USA; 223 Campus Caucaia, Federal Institute of Education, Science and Technology of Ceará, Caucaia, Brazil; 224 Peru Country Office, United Nations Population Fund (UNFPA), Lima, Peru; 225 Forensic Medicine Division, Imam Abdulrahman Bin Faisal University, Dammam, Saudi Arabia; 226 Department of Reproductive Health and Population Studies, Bahir Dar University, Bahir Dar, Ethiopia; 227 Breast Surgery Unit, Helsinki University Hospital, Helsinki, Finland; 228 University of Helsinki, Helsinki, Finland; 229 Clinical Microbiology and Parasitology Unit, Dr. Zora Profozic Polyclinic, Zagreb, Croatia; 230 University Centre Varazdin, University North, Varazdin, Croatia; 231 Institute of Addiction Research (ISFF), Frankfurt University of Applied Sciences, Frankfurt, Germany; 232 Department of Radiation Oncology, University of California San Francisco, San Francisco, CA USA; 233 Internal Medicine Department, King Saud University, Riyadh, Saudi Arabia; 234 Department of Epidemiology and Biostatistics, Shahrekord University of Medical Sciences, Shahrekord, Iran; 235 Department of Nursing, Mashhad University of Medical Sciences, Mashhad, Iran; 236 Department of Biomolecular Sciences, University of Mississippi, Oxford, MS USA; 237 Department of Pharmacy, Mizan-Tepi University, Mizan, Ethiopia; 238 Health Systems and Policy Research Unit, Ahmadu Bello University, Zaria, Nigeria; 239 Department of Health Care Management, Technical University of Berlin, Berlin, Germany; 240 Computer, Electrical, and Mathematical Sciences and Engineering Division, King Abdullah University of Science and Technology, Thuwal, Saudi Arabia; 241 Department of Epidemiology and Biostatistics, Wuhan University, Wuhan, China; 242 Department of Pediatrics and Child Health, Debre Berhan University, Debre Berhan, Ethiopia; 243 College of Medicine and Public Health, Flinders University, Adeaide, SA Australia; 244 Research and Analytics Department, Initiative for Financing Health and Human Development, Chennai, India; 245 Department of Research and Analytics, Bioinsilico Technologies, Chennai, India; 246 Laboratory of Public Health Indicators Analysis and Health Digitalization, Moscow Institute of Physics and Technology, Dolgoprudny, Russia; 247 Experimental Surgery and Oncology Laboratory, Kursk State Medical University, Kursk, Russia; 248 Department of Biotechnology, University of Central Punjab, Lahore, Pakistan; 249 Department of Pediatrics, Arak University of Medical Sciences, Arak, Iran; 250 Department of Disease Control and Environmental Health, Makerere University, Kampala, Uganda; 251 Department of General Surgery, Carol Davila University of Medicine and Pharmacy, Bucharest, Romania; 252 Department of General Surgery, Emergency Hospital of Bucharest, Bucharest, Romania; 253 Ministry of Health, Community Development, Gender, Elderly and Children, Dar es Salaam, Tanzania; 254 Department of Public Health, University of Yaoundé I, Yaoundé, Cameroon; 255 Department of Biological Sciences, University of Embu, Embu, Kenya; 256 Institute for Global Health Innovations, Duy Tan University, Hanoi, Vietnam; 257 South African Medical Research Council, Cape Town, South Africa; 258 School of Public Health and Family Medicine, University of Cape Town, Cape Town, South Africa; 259 Centre for Heart Rhythm Disorders, University of Adelaide, Adelaide, SA Australia; 260 Unit of Microbiology and Public Health, Institute of Medical Sciences, Las Tablas, Panama; 261 Department of Public Health, Ministry of Health, Herrera, Panama; 262 Department of Pediatrics, National Hospital, Abuja, Nigeria; 263 Department of International Public Health, University of Liverpool, Liverpool, UK; 264 Administrative and Economic Sciences Department, University of Bucharest, Bucharest, Romania; 265 Department of Community Health and Primary Care, University of Lagos, Idi Araba, Nigeria; 266 Department of Family and Preventive Medicine, University of Utah, Salt Lake City, UT USA; 267 Department of Psychiatry and Behavioural Neurosciences, McMaster University, Hamilton, ON Canada; 268 Department of Psychiatry, University of Lagos, Lagos, Nigeria; 269 Community Prevention and Care Services, National AIDS Control Committee, Abuja, Nigeria; 270 Centre for Healthy Start Initiative, Lagos, Nigeria; 271 Diplomacy and Public Relations Department, University of Human Development, Sulaymaniyah, Iraq; 272 Department of Pharmacology and Therapeutics, University of Nigeria Nsukka, Enugu, Nigeria; 273 University of Port Harcourt, Port Harcourt, Nigeria; 274 Department of Project Management, National Research University Higher School of Economics, Moscow, Russia; 275 Department of Medicine, University of Ibadan, Ibadan, Nigeria; 276 Department of Medicine, University College Hospital, Ibadan, Ibadan, Nigeria; 277 Department of Respiratory Medicine, Jagadguru Sri Shivarathreeswara Academy of Health Education and Research, Mysore, India; 278 Department of Forensic Medicine and Toxicology, Manipal Academy of Higher Education, Manipal, India; 279 Department of Health Metrics, Center for Health Outcomes & Evaluation, Bucharest, Romania; 280 Research Department, Nepal Health Research Council, Kathmandu, Nepal; 281 Research Department, Public Health Research Society Nepal, Kathmandu, Nepal; 282 Saveetha Medical College and Hospitals, Saveetha University, Chennai, India; 283 Iran University of Medical Sciences, Tehran, Iran; 284 Department of Pediatrics, University of Melbourne, Melbourne, VIC Australia; 285 Population Health Theme, Murdoch Children’s Research Institute, Melbourne, VIC Australia; 286 Department of Genetics, Yale University, New Haven, CT USA; 287 School of Global Public Health, New York University, New York, NY USA; 288 University Medical Center Groningen, University of Groningen, Groningen, Netherlands; 289 School of Economics and Business, University of Groningen, Groningen, Netherlands; 290 National Institute of Infectious Diseases, Bucuresti, Romania; 291 Department of Infectious Diseases, Carol Davila University of Medicine and Pharmacy, Bucharest, Romania; 292 School of Engineering, Macquarie University, Sydney, NSW Australia; 293 Pohang University of Science and Technology, Pohang, South Korea; 294 College of Medicine, University of Central Florida, Orlando, FL USA; 295 Department of Immunology, Mazandaran University of Medical Sciences, Sari, Iran; 296 Molecular and Cell Biology Research Center, Mazandaran University of Medical Sciences, Sari, Iran; 297 Metabolomics and Genomics Research Center, Tehran University of Medical Sciences, Tehran, Iran; 298 Sina Trauma and Surgery Research Center, Tehran University of Medical Sciences, Tehran, Iran; 299 Future Technology Research Center, National Yunlin University of Science and Technology, Yunlin, Taiwan; 300 Department of Epidemiology, Biostatistics and Occupational Health, McGill University, Montreal, QC Canada; 301 Research and Innovation Division, South Asian Institute for Social Transformation (SAIST), Dhaka, Bangladesh; 302 Research Department, Policy Research Institute, Kathmandu, Nepal; 303 Health and Public Policy Department, Global Center for Research and Development, Kathmandu, Nepal; 304 Department of Oral Pathology, Sharavathi Dental College and Hospital, Shimogga, India; 305 WHO Collaborating Centre for Public Health Education and Training, Imperial College London, London, UK; 306 University College London Hospitals, London, UK; 307 Department of Primary Care and Public Health, Imperial College London, London, UK; 308 Academic Public Health England, Public Health England, London, UK; 309 Department of Computer Science, Boston University, Boston, MA USA; 310 Department of Epidemiology and Biostatistics, Haramaya University, Harar, Ethiopia; 311 Research Center for Immunodeficiencies, Tehran University of Medical Sciences, Tehran, Iran; 312 Network of Immunity in Infection, Malignancy and Autoimmunity (NIIMA), Universal Scientific Education and Research Network (USERN), Tehran, Iran; 313 Faculty of Business and Management, Universiti Sultan Zainal Abidin (Sultan Zainal Abidin University), Kuala Terengganu, Malaysia; 314 Epidemiology Research Unit (EPIUnit), University of Porto, Porto, Portugal; 315 Department of Global Health, University of Washington, Seattle, WA USA; 316 Department of Medicine, University of Washington, Seattle, WA USA; 317 African Genome Center, Mohammed VI Polytechnic University (UM6P), Ben Guerir, Morocco; 318 Centro de Investigaciones en Anomalías Congénitas y Enfermedades Raras (Center for Research in Congenital Anomalies and Rare Diseases), Universidad ICESI (ICESI University), Cali, Colombia; 319 Malaria Atlas Project, Telethon Kids Institute, Perth, Australia; 320 Department of Health Statistics, National Institute for Medical Research, Dar es Salaam, Tanzania; 321 Department of Internal Medicine, University of Botswana, Gaborone, Botswana; 322 Department of Psychiatry, All India Institute of Medical Sciences, New Delhi, India; 323 Department of Public Health, Madda Walabu University, Bale Robe, Ethiopia; 324 Public Health and Community Medicine Department, Cairo University, Giza, Egypt; 325 Drug Applied Research Center, Tabriz University of Medical Sciences, Tabriz, Iran; 326 Department of Entomology, Ain Shams University, Cairo, Egypt; 327 Centre for Tropical Medicine and Global Health, University of Oxford, Oxford, UK; 328 Nuffield Department of Medicine, University of Oxford, Oxford, UK; 329 Geriatric and Long Term Care Department, Hamad Medical Corporation, Doha, Qatar; 330 Faculty of Health & Social Sciences, Bournemouth University, Bournemouth, UK; 331 College of Public Health, Medical and Veterinary Sciences, James Cook University, QLD, Townsville, Australia; 332 Public Health Division, An-Najah National University, Nablus, Palestine; 333 Independent Consultant, Karachi, Pakistan; 334 Faculty of Caring Science, Work Life, and Social Welfare, University of Borås, Borås, Sweden; 335 College of Medicine, Yonsei University, Seoul, South Korea; 336 Department of Internal Medicine, Yale University, New Haven, CT USA; 337 School of Medicine, University of Alabama at Birmingham, Birmingham, AL USA; 338 Medicine Service, US Department of Veterans Affairs (VA), Birmingham, AL USA; 339 Department No.16, Moscow Research and Practical Centre on Addictions, Moscow, Russia; 340 Department of Infectious Diseases and Epidemiology, Pirogov Russian National Research Medical University, Moscow, Russia; 341 Department of Community Medicine, Ahmadu Bello University, Zaria, Nigeria; 342 Cancer Control Center, Osaka International Cancer Institute, Osaka, Japan; 343 Department of Biomedical Sciences, Arba Minch University, Arba Minch, Ethiopia; 344 University Institute “Egas Moniz”, Monte da Caparica, Portugal; 345 Research Institute for Medicines, University of Lisbon, Lisbon, Portugal; 346 School of Public Health, Mekelle University, Mekelle, Ethiopia; 347 Southgate Institute for Health and Society, Flinders University, Adelaide, SA Australia; 348 Department of Pathology and Legal Medicine, University of São Paulo, Ribeirão Preto, Brazil; 349 Modestum LTD, London, UK; 350 College of Medicine and Health Sciences, Bahir Dar University, Bahir Dar, Ethiopia; 351 Department of Community Medicine, Alex Ekwueme Federal University Teaching Hospital Abakaliki, Abakaliki, Nigeria; 352 Kasturba Medical College, Manipal Academy of Higher Education, Mangalore, India; 353 Clinical Research Department, IRCCS Fondazione Don Carlo Gnocchi, Milan, Italy; 354 Department of Medical and Surgical Sciences, University of Bologna, Bologna, Italy; 355 Occupational Health Unit, Sant’Orsola Malpighi Hospital, Bologna, Italy; 356 Faculty of Information Technology, HUTECH University, Ho Chi Minh City, Vietnam; 357 Center of Excellence in Behavioral Medicine, Nguyen Tat Thanh University, Ho Chi Minh City, Vietnam; 358 Population Dynamics and Sexual and Reproductive Health, African Population and Health Research Center, Nairobi, Kenya; 359 Foundation University Medical College, Foundation University Islamabad, Islamabad, Pakistan; 360 Department of Cultures, Societies and Global Studies, Northeastern University, Boston, MA USA; 361 School of Public Health, University of Nairobi, Nairobi, Kenya; 362 School of Population Health and Environmental Sciences, King’s College London, London, UK; 363 Centre for Health Policy Research, Torrens University Australia, Adelaide, SA Australia; 364 Department of community Medicine, Rajarata University of Sri Lanka, Anuradhapura, Sri Lanka; 365 Department of Biostatistics, University of Washington, Seattle, WA USA; 366 School of International Development and Global Studies, University of Ottawa, Ottawa, ON Canada; 367 The George Institute for Global Health, University of Oxford, Oxford, UK; 368 Centre for Suicide Research and Prevention, University of Hong Kong, Hong Kong, China; 369 Department of Social Work and Social Administration, University of Hong Kong, Hong Kong, China; 370 Department of Neuropsychopharmacology, National Center of Neurology and Psychiatry, Kodaira, Japan; 371 Department of Public Health, Juntendo University, Tokyo, Japan; 372 Department of Bioengineering and Therapeutic Sciences, University of California San Francisco, San Francisco, CA USA; 373 Addictology Department, Russian Medical Academy of Continuous Professional Education, Moscow, Russia; 374 School of Public Health, Wuhan University of Science and Technology, Wuhan, China; 375 Hubei Province Key Laboratory of Occupational Hazard Identification and Control, Wuhan University of Science and Technology, Wuhan, China; 376 School of Medicine, Wuhan University, Wuhan, China

**Keywords:** HIV, Mapping, Africa, Geostatistics, Spatial statistics, HIV prevalence, Demographics

## Abstract

**Background:**

Human immunodeficiency virus and acquired immune deficiency syndrome (HIV/AIDS) is still among the leading causes of disease burden and mortality in sub-Saharan Africa (SSA), and the world is not on track to meet targets set for ending the epidemic by the Joint United Nations Programme on HIV/AIDS (UNAIDS) and the United Nations Sustainable Development Goals (SDGs). Precise HIV burden information is critical for effective geographic and epidemiological targeting of prevention and treatment interventions. Age- and sex-specific HIV prevalence estimates are widely available at the national level, and region-wide local estimates were recently published for adults overall. We add further dimensionality to previous analyses by estimating HIV prevalence at local scales, stratified into sex-specific 5-year age groups for adults ages 15–59 years across SSA.

**Methods:**

We analyzed data from 91 seroprevalence surveys and sentinel surveillance among antenatal care clinic (ANC) attendees using model-based geostatistical methods to produce estimates of HIV prevalence across 43 countries in SSA, from years 2000 to 2018, at a 5 × 5-km resolution and presented among second administrative level (typically districts or counties) units.

**Results:**

We found substantial variation in HIV prevalence across localities, ages, and sexes that have been masked in earlier analyses. Within-country variation in prevalence in 2018 was a median 3.5 times greater across ages and sexes, compared to for all adults combined. We note large within-district prevalence differences between age groups: for men, 50% of districts displayed at least a 14-fold difference between age groups with the highest and lowest prevalence, and at least a 9-fold difference for women. Prevalence trends also varied over time; between 2000 and 2018, 70% of all districts saw a reduction in prevalence greater than five percentage points in at least one sex and age group. Meanwhile, over 30% of all districts saw at least a five percentage point prevalence increase in one or more sex and age group.

**Conclusions:**

As the HIV epidemic persists and evolves in SSA, geographic and demographic shifts in prevention and treatment efforts are necessary. These estimates offer epidemiologically informative detail to better guide more targeted interventions, vital for combating HIV in SSA.

**Supplementary Information:**

The online version contains supplementary material available at 10.1186/s12916-022-02639-z.

## Background

Four decades after its discovery, human immunodeficiency virus (HIV) continues to impact millions of people worldwide, remains one of the leading causes of morbidity and mortality globally [1, 2] and incurs billions of dollars annually in direct health care costs and indirect socioeconomic costs [3]. In sub-Saharan Africa (SSA) in 2019, an estimated 26 million people were living with HIV [2]. In recent years, international bodies have set goals to end the HIV epidemic: in 2014, the Joint United Nations Programme on HIV/AIDS (UNAIDS) introduced the “95-95-95” targets—that by 2030, 95% of people living with HIV globally would know their status, 95% of all people with diagnosed HIV infection would receive sustained antiretroviral therapy, and 95% of people living with HIV receiving antiretroviral therapy (ART) would be virally suppressed [4, 5]. The United Nations Sustainable Development Goals also call for an end to the AIDS epidemic by 2030 [6]. Unfortunately, despite a significant increase in ART coverage over the last 20 years and major progress in terms of reductions in HIV incidence and mortality [1], the latest estimates and projections indicate that the world is not on track to meet these goals [2, 7, 8], and progress may stall further as a consequence of the COVID-19 pandemic [9].

Differences in HIV prevalence both within and between nations in SSA have been well-documented [10–14], as have differences between sexes [2, 12–14] and age groups [2]. These differences have also changed over time [1, 10], impacted in part by the onset, duration, location, and demographic targeting of different prevention and treatment interventions [15–17]. Epidemiologically targeted interventions are understood to be more effective compared to homogeneous interventions [18] and are increasingly important at a time when the future of funding for HIV prevention and treatment is both uncertain and highly variable [19, 20], particularly in the wake of disruptions related to the COVID-19 pandemic [21]. Evidence suggests that interventions are most effective when tailored to account for differences in the intensity of the epidemic by geographic location [14, 22], sex [23], and age [24]. Locally and demographically precise HIV prevalence information, however, is necessary in order to maximize the benefit of such methods; at present, such information in SSA is lacking.

HIV prevalence estimates stratified by age and sex are available at the national level through the Global Burden of Disease (GBD) [2] and from UNAIDS [25]. Both sources also provide subnational estimates at the first administrative level (e.g., province, state) in select countries. Recently, Dwyer-Lindgren et al. [10] presented aggregated adult HIV prevalence estimates for the years 2000–2017 at local scales in SSA, generalizing estimates for males and females combined, and across ages 15–49 years. Some studies have gone further to present subnational prevalence estimates separated by sex [26–29] or age [30]; however, these studies focused on single countries, and/or presented estimates for only one point in time, without describing any temporal trajectories in prevalence. To our knowledge, no previous studies have presented age- and sex-specific HIV prevalence estimates across SSA at local scales over time.

We built upon the HIV prevalence model from Dwyer-Lindgren et al. [10] to produce HIV prevalence estimates for 43 countries in SSA for males and females ages 15–59 years, stratified into nine 5-year age groups, for the years spanning 2000 to 2018. Countries, age groups, and time period were selected according to data availability. We expanded upon existing Bayesian spatiotemporal methods to model these estimates at a 5 × 5-km resolution and present them here aggregated to the second administrative level (which varies by country but is typically equivalent to e.g., districts, municipalities), which is the level typically considered most relevant to policymakers and stakeholders. Prevalence estimates for all demographic groups at all levels of geographic aggregation, as well as number of people living with HIV (count estimates), are publicly available from the Global Health Data Exchange (https://ghdx.healthdata.org/record/ihme-data/sub-saharan-africa-hiv-prevalence-geospatial-estimates-2000-2018) and through a user-friendly data visualization tool (http://vizhub.healthdata.org/lbd/hiv-prev-disagg).

## Methods

### Overview

This ecological study follows the Guidelines for Accurate and Transparent Health Estimates Reporting (GATHER) [31] (Additional file 1: Section 1). This analysis relies secondary data sources to provide estimates of HIV prevalence on a 5 × 5-km grid in 43 countries in SSA for males and females ages 15–59 years residing at each location, stratified into five-year age bins (i.e., ages 15–19, 20–24, 25–29, 30–34, 35–39, 40–44, 45–49, 50–54, 55–59), with annual resolution from year 2000 to 2018 inclusive, calibrated to national estimates from the GBD [2]. The period of 2000–2018 and the age range of 15–59 years were selected to optimize the contemporaneousness of the estimates and to account for data availability—there were relatively few large-scale seroprevalence surveys conducted before 2000, and most seroprevalence surveys focus on adults, with little reporting outside the 15–59 years age range. We produced estimates for sex rather than gender binaries because sex is more predominantly reported in the available data sources. Due to data availability limitations we were unable to produce prevalence estimates for sex minority individuals outside the male/female binary. The 43 countries analyzed were also selected according to data availability—Mauritania was excluded as there were no HIV prevalence data available. We included six countries—Djibouti, Guinea-Bissau, Madagascar, Somalia, South Sudan and Sudan—where no seroprevalence survey data were available, but where sentinel surveillance data collected from antenatal care clinic (ANC) attendees (described below) were available. The implications of these and other limitations are expanded upon in the “Methodological advantages and limitations” section in the “Discussion” section.

**Fig. 1:**
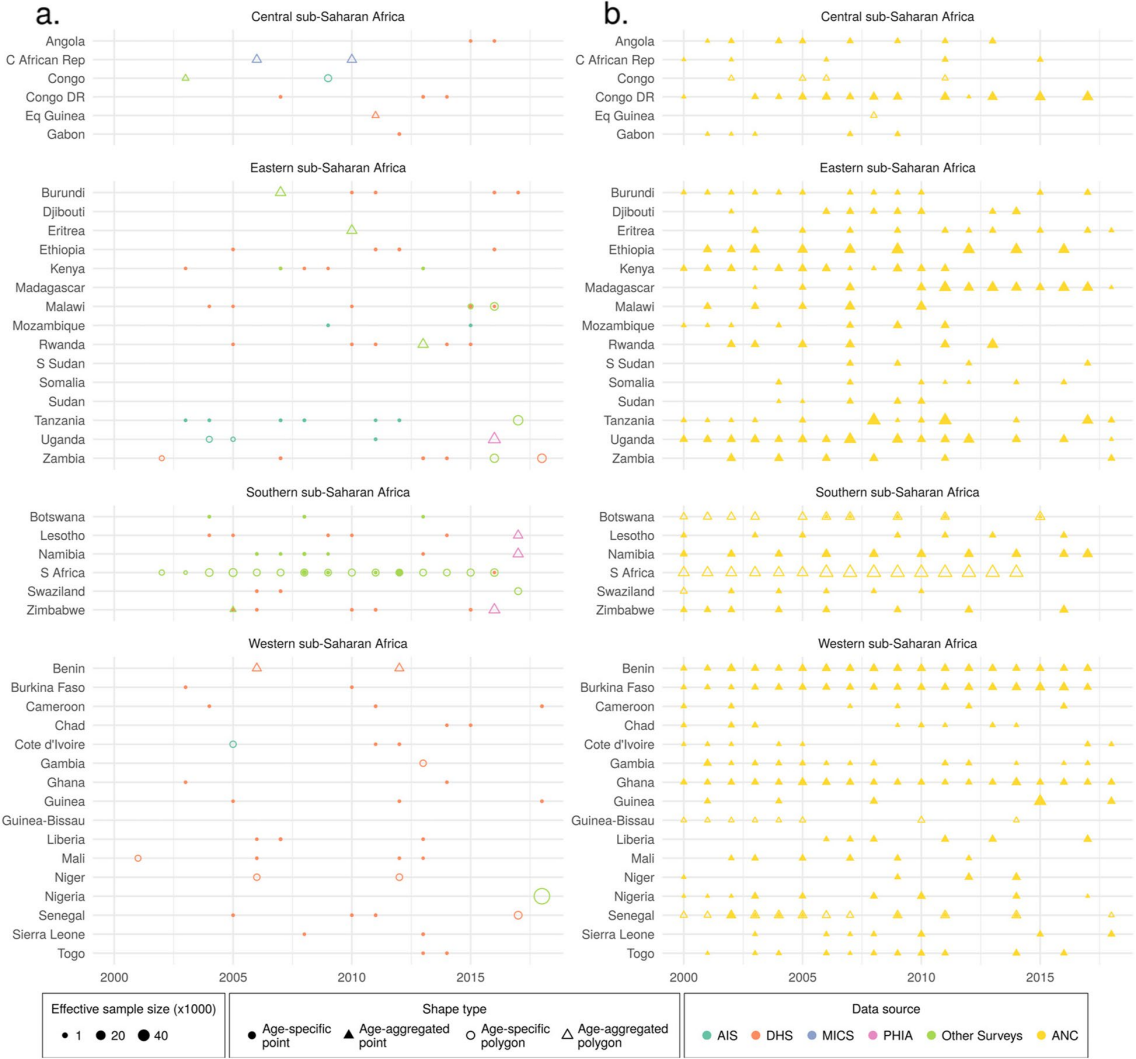
HIV prevalence data by region and country. **a** HIV seroprevalence survey data and **b** ANC sentinel surveillance data used in this analysis, by region and country. Color indicates the data source. AIS, AIDS Indicator Survey; DHS, Demographic and Health Survey; MICS, Multiple Indicator Cluster Survey; PHIA, Population-based HIV Impact Assessment Survey. Shape type indicates whether a data source is age-specific and has point (GPS) or polygon location information. Size indicates the relative effective sample size for each source. A full list of data sources with additional details about data type (such as survey microdata and survey reports) and geographical details are provided in Additional file 2: Tables S1-S5

**Fig. 2:**
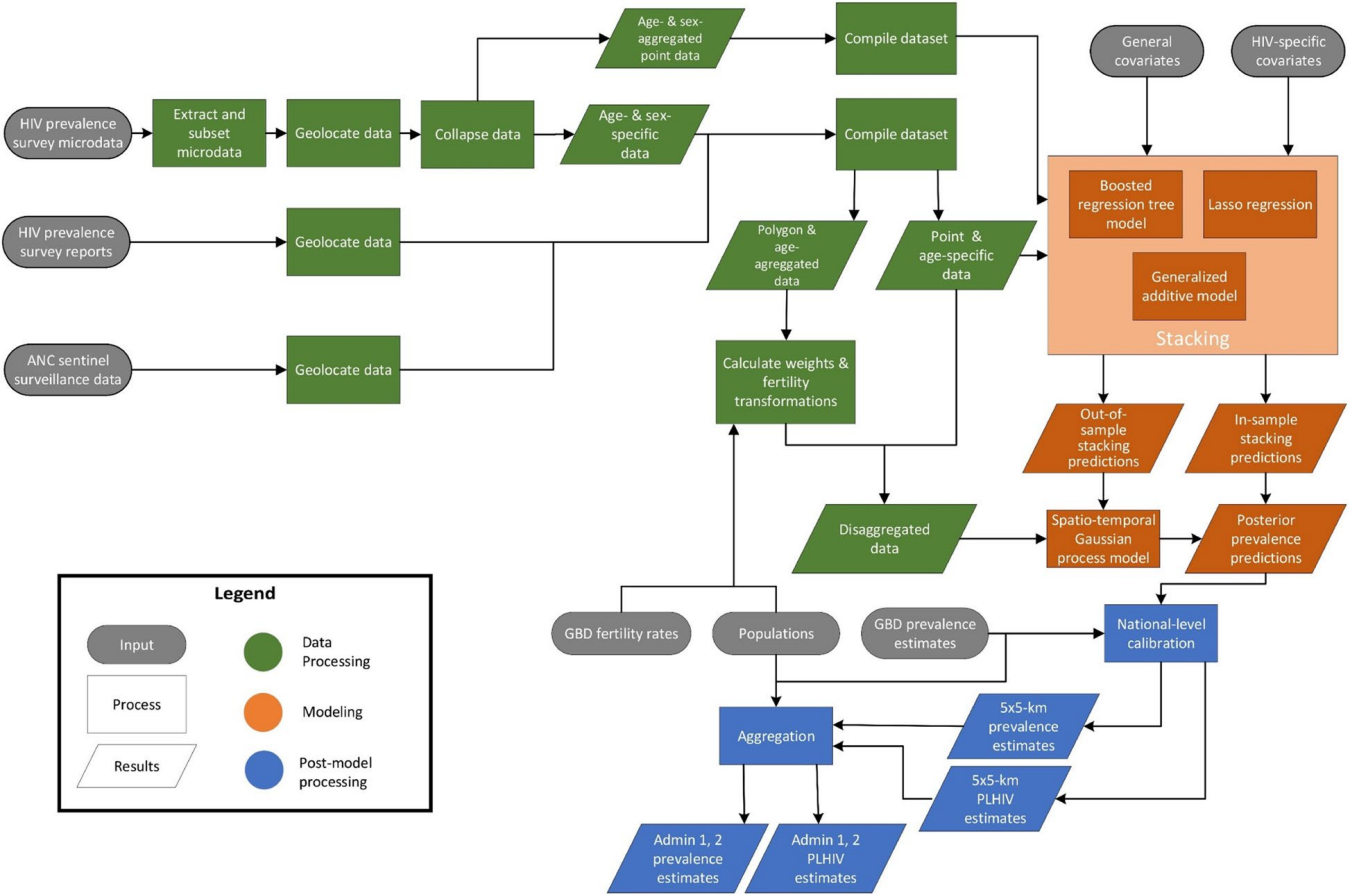
Analytical process overview. The process used to produce age- and sex-specific HIV prevalence estimates in sub-Saharan Africa involved three main parts. In the data-processing steps (green), data were identified, extracted, and prepared for use in the HIV prevalence model and in covariate models. In the modeling phase (orange), we used these data and covariates in a stacked generalization ensemble model and spatiotemporal Gaussian process model. In the post-processing phase (blue), we calibrated the prevalence estimation to match GBD 2019 estimates at the national level, aggregated prevalence estimates to the first- and second-level administrative subdivisions in each country, and calculated the number of people living with HIV (PLHIV)

The methodology used here largely parallels that previously used to map adult HIV prevalence in SSA [10], with the incorporation of modifications necessary to model by age and sex, and improvements related to the inclusion of spatially aggregated data and ANC data (Fig. 1). We used a 5 × 5-km grid for consistency with this previous analysis; to align with the resolution available for pre-existing covariates incorporated in this analysis; and for flexibility in aggregating these estimates to other levels of interest (e.g., first- and second-level administrative subdivisions, such as states or districts, respectively, or more aggregated age groups such as reproductive ages [commonly 15-49]) using grid-cell-level estimates of age- and sex-specific population from Worldpop [32]. These population estimates were also used to estimate the number of people living with HIV in each demographic group. All analyses were conducted in R version 3.6.1 [33]. Figure 2 provides an overview of the analytic process, described in more depth below. Additional details are available in Additional file 1.

### Data

#### HIV data

We compiled a geolocated dataset of 304,672 observations from 91 seroprevalence surveys from 37 countries and 10,351 observations from sentinel surveillance among antenatal care clinic attendees (ANC data) in 43 countries (Additional file 2: Tables S1-S2; Fig. 1). Data from seroprevalence surveys were originally in the form of survey microdata (that is, individual-level survey responses) or survey reports (Additional file 2: Table S1). For surveys with available microdata, we extracted variables related to age, sex, HIV blood test result, location, and year, as well as survey weights, where available. We excluded rows with missing information on any of these variables, and subset the data to ages 15–59 years. For data coded by gender rather than sex, we treated these data as if they were sex-specific rather than gender-specific. We recognize that sex and gender are not interchangeable: sex is a biological variable, while gender is a fluid social construct. In the absence of quality data, however, we could not disaggregate estimates by gender at this time. After subsetting by age, we collapsed the age-specific data into 5-year age bins (hereafter referred to as “ages”) by sex. We did this by calculating the weighted age- and sex-specific HIV prevalence at the finest spatial resolution available. Ideally, this was at the level of global positioning system (GPS) coordinates that represent the location of a survey cluster. In most surveys, GPS coordinates are randomly displaced (typically by 2–5 km depending on the setting and the survey series [34]) in order to protect respondent’s confidentiality. In instances where GPS coordinates were not available, the smallest areal unit (termed a “polygon”) possible was used instead. These typically represented an administrative subdivision. For surveys without microdata but for which estimates with some subnational resolution were provided in a report, we extracted these estimates with information about the sample size and location. GPS coordinates were not available for these reports, so these data were exclusively matched to polygons. In most reports, age ranges larger than 5 years were reported. Among these, we retained data reported for age ranges that corresponded exactly to one or more of the 5-year age bins used in this model; for example, we included surveys covering age ranges 15–49 years, or 15–24 years, but excluded those covering age ranges such as 18–24 years. For age-aggregated data, we retained information regarding the age range covered, to be used in our modeling process as described below. We also only included sex-specific data. For more information on excluded surveys see Additional file 2: Table S3.

Data that were spatially aggregated (i.e., polygon data) and/or age-aggregated required additional processing. Although we ultimately modeled HIV prevalence at the level of the observation, be it point or polygon, age-specific or age-aggregated, our modeling process initially specified HIV prevalence at the point-, time-, age-, and sex-specific level. Because of this, it was necessary that we disaggregate the age-aggregated and polygon survey data to be location- and age-specific. We did this by distributing polygon data to pixels proportional to population. Specifically, for each polygon, we generated points at the centroid of each 5 × 5-km pixel falling within that polygon and replicated that observation’s HIV prevalence and sample size at the location of each of those centroids. Age-aggregated point data were similarly disaggregated by replicating the HIV prevalence and sample size once for each year-age group covered in the overall age range. In the cases of age-aggregated polygon data, these two processes were combined. Next, each of the disaggregated, location- and age-specific rows of data associated with a given aggregated observation were assigned weights proportional to the age- and sex-specific population residing at that location for the given year, derived from WorldPop [32]. Weights per observation all summed to one. This process substantially increased the size of the dataset. To reduce the associated computational burden when fitting the model, in cases where at least one row within an observation was given a weight of less than half of one divided by the number of locations and/or ages in that observation, we successively dropped the lowest-weighted locations and/or ages until reaching a maximum of 1% of the observation’s weight dropped. Remaining locations and/or ages within that observation were then reweighted to maintain a total weight of one. Data that were not aggregated (i.e., age-specific point observations) were each assigned a weight of one.

ANC data were primarily derived from national HIV estimate files developed by national teams and compiled and shared via UNAIDS [35] and supplemented with data derived from sentinel surveillance country reports (Additional file 2: Table S2). We extracted information from these sources on HIV prevalence and sample size by site and year. Sites were geolocated to specific GPS coordinates where possible and otherwise to a polygon that represents an administrative subdivision. The ANC data available for this analysis were not age-specific. Because ANC data included only pregnant females, we assumed the age range of these data to be that of females with non-zero fertility rates in SSA according to GBD 2019 [36], that is, females ages 15–54 years. We disaggregated ANC data to the age and location level as we did for age-aggregated or polygon survey data. However, specific locations and ages were weighted by number of births rather than population size. The number of births for a given age and location was estimated as the product of the location-, age-, and sex-specific population, again derived from WorldPop [32], and the national fertility rate, derived from GBD 2019 estimates [36].

#### Covariates

This analysis included the same covariates as the previous analysis [10]. This included five pre-existing covariates: (1) travel time to the nearest settlement of more than 50,000 inhabitants; (2) total population; (3) night-time lights; (4) urbanicity; and (5) malaria incidence (Additional file 2: Table S4). In addition, eight covariates were constructed explicitly for this analysis owing to their known association with HIV prevalence and data availability: (1) prevalence of male circumcision (all forms); (2) prevalence of self-reported sexually transmitted infection (STI) symptoms; (3) prevalence of marriage or living with a partner as married; (4) prevalence of one’s current partner living elsewhere among females; (5) prevalence of condom use at last sexual encounter; (6) prevalence of reporting ever having had intercourse among young females; and (7) and (8) prevalence of multiple partners in the past year for males and for females, respectively. We updated the covariates constructed for this analysis to incorporate newly available data but utilized the original statistical methods (Additional file 1: Section 3.2; Additional file 2: Table S5; Additional file 3: Figs. S1-S8).

### Model and estimation

#### Covariate stacking

An ensemble covariate modeling approach (“stacking”) was implemented to capture possible nonlinear interactions among the covariates across space and time [37]. In this approach, three sub-models were fitted to the HIV survey data with the covariates as explanatory predictors: generalized additive models [38], boosted regression trees [39], and lasso regression [40]. Each sub-model was fitted using fivefold cross-validation to avoid overfitting, and the out-of-sample predictions from across the five folds were compiled into a single set of predictions that were used to fit the geostatistical model described below. In addition, each sub-model was also fitted to the full dataset to generate a complete set of in-sample predictions that were subsequently used when generating predictions from the geostatistical model (Additional file 3: Figs. S9-S11). Because the covariates used here were neither age-specific nor (for most) sex-specific, we fit these sub-models at that same age- and sex-aggregated level as the HIV-specific covariates, modeling HIV prevalence data aggregated across ages 15–49 and males and females. The age range 15–49 years was used in this case because of its more common usage in seroprevalence surveys compared to the 15–59 years range, allowing us to retain more data for the stacking model. Polygon data were excluded from stacking models due to their incongruity with the configurations needed for the different sub-models. The ANC data were also excluded due to known sampling biases, which are described in the Additional file 1: Section 4.2.

#### Geostatistical model

This model was fit in Template Model Builder (TMB) [41]. Owing to computational constraints, and to allow for regional differences in the relationships between covariates and HIV prevalence, as well as differences in the temporal, spatial, and demographic autocorrelation in HIV prevalence, separate models were fitted for four regions (Additional file 3: Fig. S12). We modeled HIV prevalence stratified by space, time, age, and sex using a generalized linear mixed-effects model. To simultaneously model point- and polygon-level observations, as well as both age-specific and age-aggregated observations, we specified the data likelihood at the observation level (*i*), which accommodated all of these. We modeled the number of HIV-positive individuals (*Y*_*i*_) among a sample (*N*_*i*_) for a given observation as a binomial variable:

$${Y}_i\sim \textrm{Binomial}\left({N}_i,{p}_i\right)$$

Logit-transformed prevalence was however first specified at the space, time, age, and sex-disaggregated level (*j*):

$${\displaystyle \begin{array}{c}\textrm{logit}\left({p}_j\right)={\beta}_0+{\boldsymbol{\beta}}_1{\boldsymbol{X}}_j+{Z}_{1,j}+{Z}_{2,j}+{Z}_{3,c\left[j\right]}\\ {}{Z}_{1,j}\sim \textrm{GP}\left(0,{\varSigma}_{1, space}\otimes {\varSigma}_{1, time}\right)\\ {}\begin{array}{c}{Z}_{2,j}\sim \textrm{GMRF}\left(0,{\varSigma}_{2, time}\otimes {\varSigma}_{2, age}\otimes {\varSigma}_{2, sex}\right)\\ {}{Z}_{3,c\left[j\right]}\sim \textrm{GMRF}\left(0,{\varSigma}_{3,c}\right)\end{array}\end{array}}$$

We specified logit-transformed prevalence at the disaggregated level (*p*_*j*_) as a linear combination of:

- A regional intercept (*β*_0_);
- Covariates and associated regression parameters (***β***_1_***X***_*j*_);
- Random effects correlated across space and time, (*Z*_1, *j*_);
- Random effects correlated across time, age, and sex, (*Z*_2, *j*_);
- Country-specific (*c*) random effects correlated across age, (*Z*_3, *c*[*j*]_).

The random effects capturing correlations between space, time, age, and sex included:

- *Z*_1, *j*_: a Gaussian process with mean 0 and a covariance matrix given by the Kronecker product of a spatial Matérn covariance function [42] (*Σ*_1, *space*_) and a temporal first-order autoregressive covariance function (*Σ*_1, *time*_);
- *Z*_2, *j*_: a Gaussian Markov Random Field with mean 0 and a covariance matrix given by the Kronecker product of first-order autoregressive covariance functions for time (*Σ*_2, *time*_), age (*Σ*_2, *age*_), and sex (*Σ*_2, *sex*_);
- *Z*_3, *c*[*j*]_: a Gaussian Markov Random Field with mean 0 and a covariance matrix given by country-specific first-order autoregressive covariance functions for age (*Σ*_3, *c*_).

We used the stochastic partial differential equation [43] approach to approximate the continuous spatiotemporal Gaussian random field (*Z*_1, *j*_). Sensitivity analyses were carried out to compare this model configuration to others with differing *p*_*j*_ specification configurations, as well as to several other model and data specifications, and are described in detail in the Additional file 1: Section 4.3, Additional file 3: Figs. S13-S15, and the “Discussion” section. We then specified observation-level (*i*) prevalence:

$${p}_i={\textrm{logit}}^{-1}\left(\textrm{logit}\left(\sum \left({p}_{transformed,j}\cdot {w}_j\right)\right)+\left({\beta}_2+{U}_{s\left[i\right]}\right)\cdot {I}_{ANC}+{\epsilon}_i\right)$$

*p*_*i*_ was calculated as the sum of disaggregated prevalence (*p*_*transformed*, *j*_) estimates multiplied by their respective population (or in the case of ANC data, birth) weights (*w*_*j*_), plus the incorporation of additional ANC-related transformations and bias corrections (*β*_2_, *U*_*s*[*i*]_, and *I*_*ANC*_ described below), and an observation-level uncorrelated error term (*ϵ*_*i*_):

$${\upepsilon}_i\sim \textrm{Normal}\left(0,{\sigma}_i^2\right)$$

In cases where data were already disaggregated spatially and by age, *w*_*j*_ = 1.

HIV prevalence as measured by sentinel surveillance of ANC clinic attendees is known to be biased as a measure of HIV prevalence in the general adult female population [44], because it only covers pregnant females who attend ANC, compared to all adult females [45, 46]. Additionally, fertility rates differ between HIV^+^ and HIV^-^ females, with the exact relationship varying by age [47], thereby impacting age-specific ANC clinic visitation rates. To address this, for ANC data we transformed prevalence among pregnant females based on the underlying prevalence among all females and the age-specific fertility-rate ratio (HIV^+^ fertility/HIV^-^ fertility). For ANC data,

$${p}_{transformed,j}=\frac{\left({p}_j\cdot {FRR}_j\right)}{\left({p}_j\cdot {FRR}_j\right)+1-{p}_j}$$

Fertility rate ratios (*FRR*_*j*_) were derived from GBD 2019 fertility estimates [36], taken at the national level except in cases where subnational estimates were available (in Ethiopia, Nigeria, and South Africa). For survey data,

$${p}_{transformed,j}={p}_j$$

To allow for additional ANC-related bias at the observation level (*i*), in instances where data in our model were derived from ANC sentinel surveillance (where *I*_*ANC*_ = 1 for ANC data, and *I*_*ANC*_ = 0 for all other data) our model incorporated a fixed term (*β*_2_) that captured overall mean bias in the ANC data, and a random effect (*U*_*s*[*i*]_) for a given ANC site *s* that captured spatial differences in the extent of this bias:

$${U}_{s\left[i\right]}\sim \textrm{Normal}\left(0,{\sigma}_{site\left[i\right]}^2\right)$$

Fitted model parameters are detailed in Additional file 2: Table S6. From each fitted model, we generated 1000 draws from the approximated joint posterior distribution of all model parameters and used these to construct 1000 draws of *p*_*j*_, setting *I*_*ANC*_ to 0. Fivefold cross-validation was used to assess model performance and to compare a number of alternative models (Additional file 3: Figs. S13-S15). We also compared the re-aggregated adult-level estimates from our final model to those from the results of an age- and sex-aggregated counterpart (Additional file 3: Fig. S16).

#### Post-estimation

To take advantage of the more structured modeling approach and additional national-level data used by GBD 2019 [2], we performed post hoc calibration of our estimates to the corresponding national-level GBD estimates. For each country, year, age bin, and sex in our analysis, we defined a “raking factor” equal to the ratio of the GBD estimate for this country-year-age-sex to the population-weighted posterior mean HIV prevalence in all corresponding grid cells (Additional file 3: Figs. S17-S18). These raking factors were then used to scale each draw of HIV prevalence for each grid cell within that GBD geography, year, age, and sex. Point estimates for each grid cell were calculated as the mean of the scaled draws, and 95% uncertainty intervals were calculated as the 2.5th and 97.5th percentiles of the scaled draws. Grid cells that crossed international borders within modeling regions were fractionally allocated to multiple countries in proportion to the covered area during this process. In cases where subnational (i.e., first administrative level) estimates were available from the GBD, that is, for Ethiopia, Nigeria and South Africa, we calibrated to those estimates rather than those at the national level. Uncertainty in GBD estimates was not accounted for in this calibration.

In addition to estimates of HIV prevalence on a 5 × 5-km grid, we constructed estimates of HIV prevalence for first- and second-level administrative subdivisions. We did this by calculating age- and sex-specific population-weighted averages of prevalence for all grid cells within a given area. This process was carried out for each of the 1000 posterior draws (after calibration to GBD), with final point estimates derived from the mean of these draws and uncertainty intervals from the 2.5th and 97.5th percentiles. Additionally, estimates of the number of people living with HIV for a given age and sex in each grid cell were derived by multiplying estimated prevalence in each grid cell by the corresponding population estimate from WorldPop [32], which was also calibrated to match GBD 2019 [36] (Additional file 1: Section 4.4; complete estimates of people living with HIV are available along with all prevalence estimates at (https://ghdx.healthdata.org/record/ihme-data/sub-saharan-africa-hiv-prevalence-geospatial-estimates-2000-2018)).

Although the model makes predictions for all locations covered by available covariates, all final model outputs for which land cover was classified as barren or sparsely vegetated according to European Space Agency Climate Change Initiative satellite data [48] and for which total population density was less than 10 individuals per 1 × 1-km in 2015 were masked for improved clarity when communicating with data specialists and policymakers. Maps were generated in R using the *ggplot2* [49] package version 3.3.0.

## Results

### Geographic variation

**Fig. 3:**
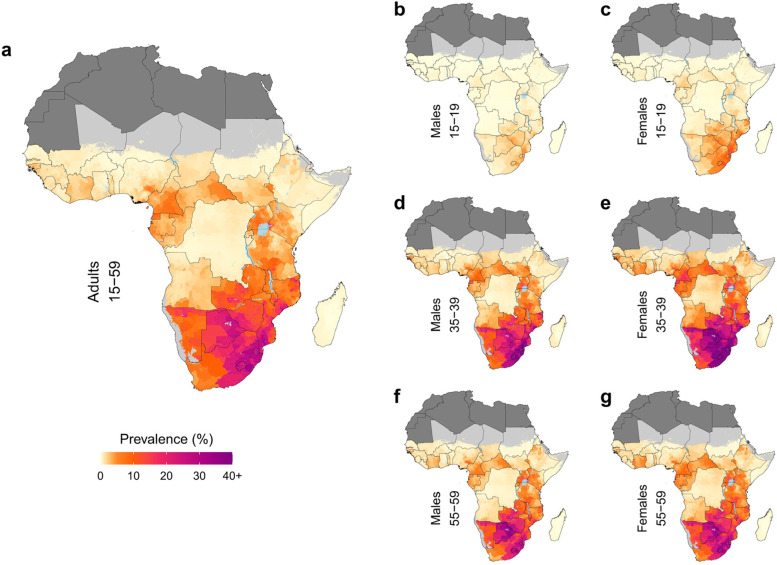
HIV prevalence in sub-Saharan Africa in 2018 at the second administrative level for a subset of modeled demographic groups from the lower, middle, and upper age ranges: **a** all adults, ages 15–59 years; **b** males and **c** females ages 15–19 years; **d** males and **e** females ages 35–39 years; and **f** males and **g** females ages 55–59 years. Maps reflect national boundaries, land cover, lakes, and population; areas with fewer than ten people per 1 × 1 km, and classified as barren or sparsely vegetated, are colored light gray. Countries colored in dark gray were not included in the analysis

**Fig. 4:**
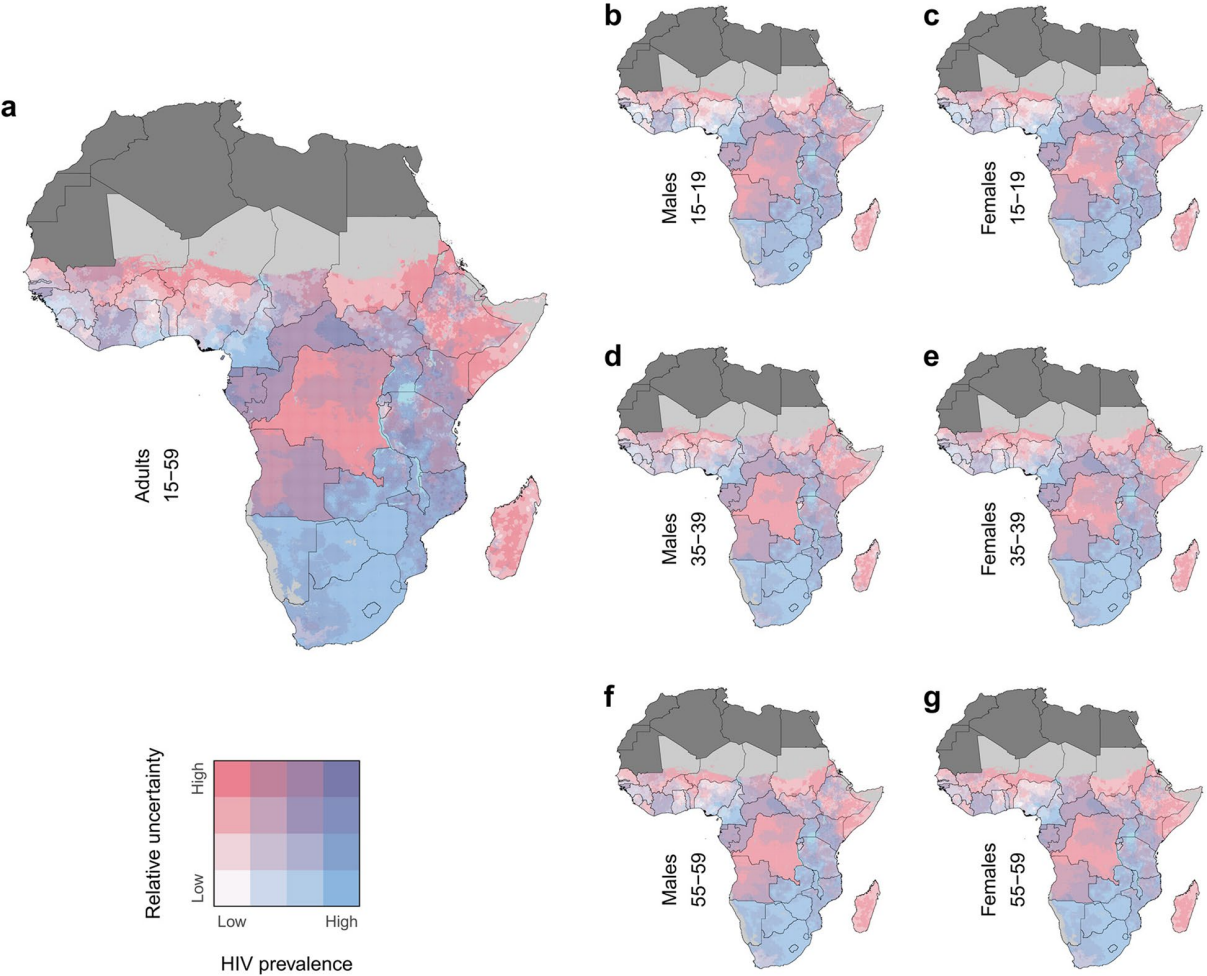
Relative uncertainty in HIV prevalence, 2018. Overlapping population-weighted quartiles of HIV prevalence (constructed separately for each demographic group) and relative 95% uncertainty in 2018 at the 5 × 5-km grid cell level for select demographic groups: **a** all adults, ages 15–59 years; **b** males and **c** females ages 15–19 years; **d** males and **e** females ages 35–39 years; and **f** males and **g** females ages 55–59 years. Relative uncertainty is defined as the ratio of the width of the 95% uncertainty interval to the mean estimate. Maps reflect national boundaries, land cover, lakes, and population; areas with fewer than ten people per 1 × 1 km, and classified as barren or sparsely vegetated, are colored light gray. Countries colored in dark gray were not included in the analysis

We found large differences in the spatial and demographic distribution of estimated HIV prevalence in SSA that were masked in demographically aggregated estimates (Figs. 3 and 4; Additional file 3: Figs. S19-S34). This was particularly striking among middle and older age groups. For example, in the year 2018, the maximum estimated HIV prevalence in any second-level administrative unit for adults ages 15–59 years was 35.4% in Umgungundlovu in the Kwazulu Natal province, South Africa (95% uncertainty interval (UI), 22.3–46.3%). However, estimated prevalence reached up to 59.4% [46.5–71.2%], almost 1.7 times higher, for females ages 35–39 years within that same location. Across all second-level administrative units, age groups, and sexes, females ages 35–39 in Nkilongo in Lubombo, Eswatini, had the highest estimated HIV prevalence in the year 2018, at 62.5% [50.1–74.5%].

Geographic variation within countries was also more dramatic in our demographically disaggregated results. Across SSA countries, the median absolute difference between second-level administrative units with the lowest and highest estimated prevalence within a given country in 2018 was 3.5 times greater when considered across ages and sexes, than when estimated for all adults combined (11.2 percentage points versus 3.2 percentage points). This difference in within-country prevalence range between demographically aggregated versus disaggregated estimates varied greatly between countries. For example, in Mozambique, this range across second-level administrative units was 30.1 percentage points [16.7–46.3] for combined adults and 56.9 percentage points [37.4–78.2] (or 1.9 times larger) for estimates across ages and sexes. In Lesotho, on the other hand, this range was 8.2 times larger for estimates across ages and sexes compared to adults combined (51.6 percentage points [40.1–63.5] versus 6.3 percentage points [1.4–11.5]). Overall, countries in Eastern SSA tended to see greater such discrepancies compared to other regions; here, the median absolute difference between second-level administrative units was 4.4 times greater when considered across ages and sexes than for all adults combined (14.0 versus 3.2 percentage points). For complete geographic variation comparisons within each country, including uncertainty estimates, see Additional file 4.

### Variation between males and females

**Fig. 5:**
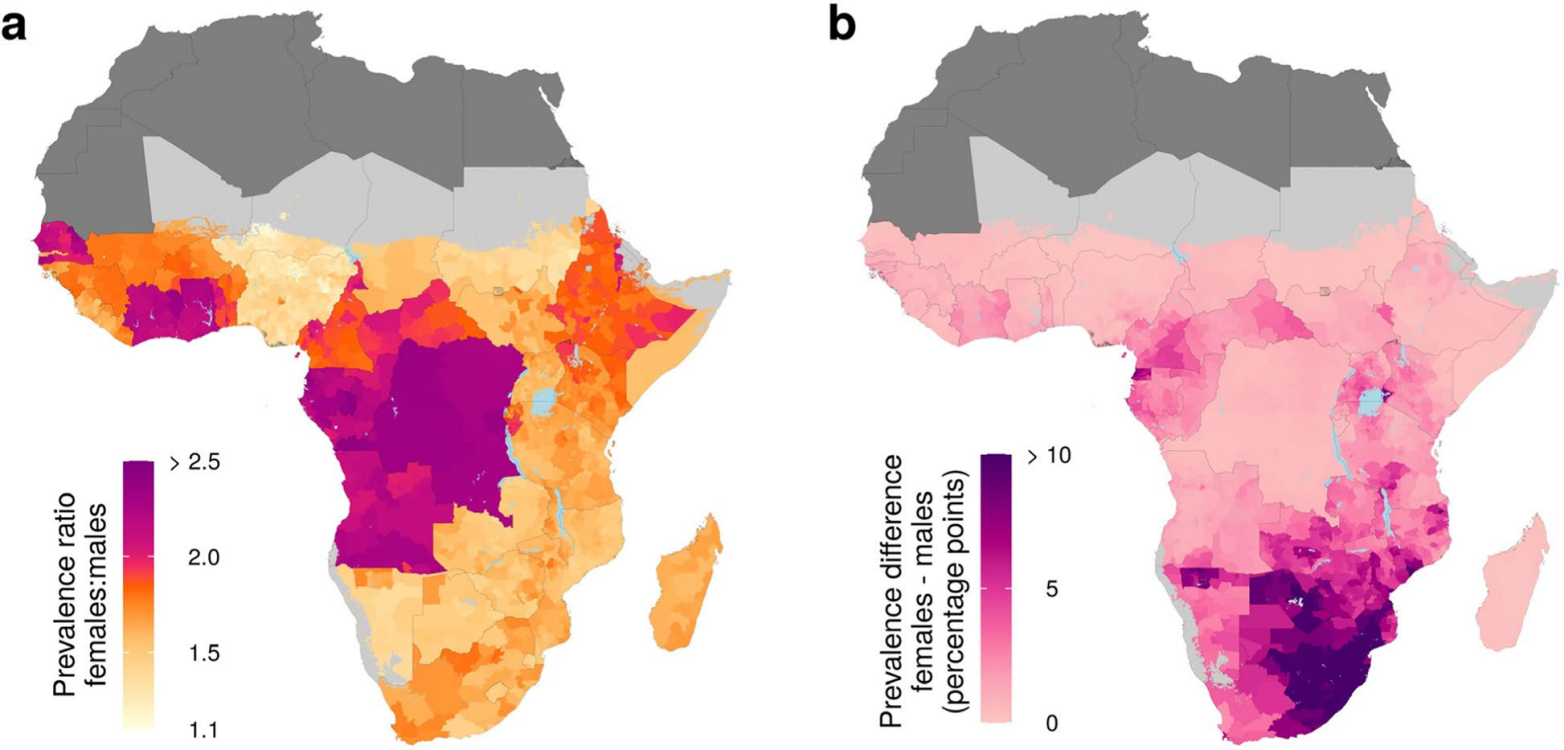
Differences in estimated prevalence between males and females ages 15–59 years at the second administrative level in 2018, calculated as **a** the ratio of estimated prevalence among females to prevalence among males and **b** the absolute difference in estimated prevalence between females and males. Maps reflect national boundaries, land cover, lakes, and population; areas with fewer than ten people per 1 × 1 km, and classified as barren or sparsely vegetated, are colored light gray. Countries colored in dark gray were not included in the analysis

Across SSA and across the years 2000–2018, estimated HIV prevalence was generally higher among females than males (Fig. 5). In 2018, for prevalence aggregated across ages 15–59 years, in no second-level administrative units was estimated prevalence higher among males compared to females. The absolute difference in estimated prevalence in 2018 between females and males reached a maximum of 15.0 percentage points (in Umkhanyakude, in KwaZulu-Natal, South Africa, with 36.3% [24.7–46.8%] estimated prevalence in females compared to 21.3% [13.1–28.7%] estimated prevalence in males), for a female to male prevalence ratio of 1.7 [1.5–1.9]. Countries in Central SSA, where overall prevalence was lower than in other SSA regions, tended to see the largest disparity between females and males in terms of relative differences. Estimated prevalence among females in Central SSA ranged up to a maximum of 2.7 [1.84–4.2] times greater than estimated prevalence in males in 2018 (in San Antonio de Palé, in Annobón, Equatorial Guinea, with 8.3% [2.1–21.4%] prevalence in females compared to 3.1% [0.8–8.1%] prevalence in males). Across Central SSA second-level administrative units, the median ratio between female and male estimated prevalence was 2.2, compared to the all-SSA median ratio of 1.6. The greatest absolute differences were seen in Eastern SSA, where the median absolute difference between female and male estimated prevalence was 1.9 percentage points in 2018, compared to the all-SSA median absolute difference of 0.9 percentage points. These differences between female and male prevalence in 2018 were less than those observed in the year 2000, when the median ratio between female and male estimated prevalence was 1.5, and the median absolute difference was 1.5 percentage points. We did not note substantial differences in within-country variations in prevalence between females and males in either 2000 or 2018 in any region. For complete comparisons between sexes by second-level administrative unit, including uncertainty estimates, see Additional file 4.

### Variation between age groups

**Fig. 6:**
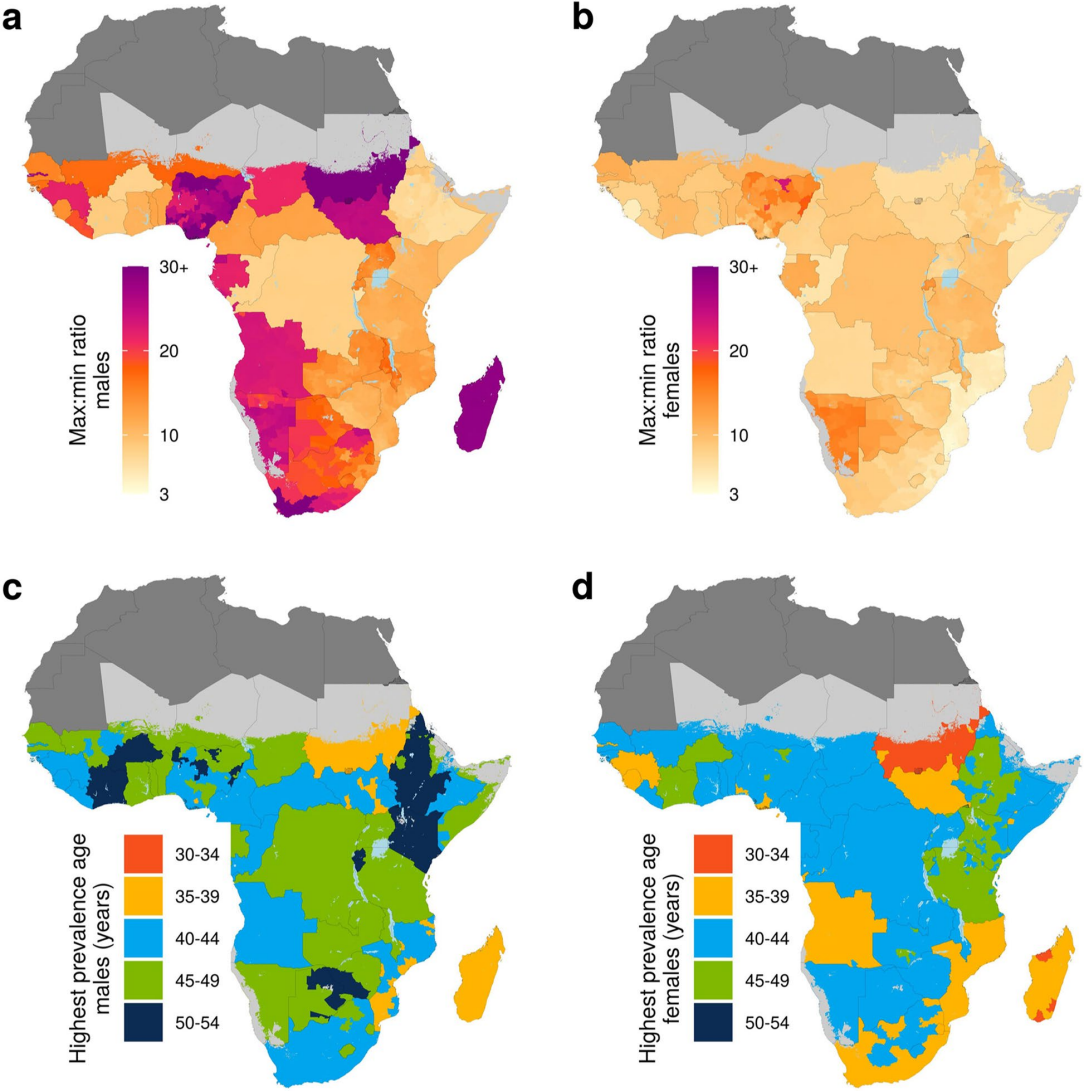
Differences in prevalence between age groups in the year 2018 at the second administrative level, calculated as the ratio of estimated prevalence between the age groups with highest and lowest prevalence, for **a** males **b** and females; and the age groups with highest prevalence for **c** males **d** and females in 2018. Maps reflect national boundaries, land cover, lakes, and population; areas with fewer than ten people per 1 × 1 km, and classified as barren or sparsely vegetated, are colored light gray. Countries colored in dark gray were not included in the analysis

Prevalence within second-level administrative units was also highly variable across age groups (Fig. 6), and relative variation in prevalence between age groups in 2018 tended to be higher in males. Comparing estimated prevalence across age groups within a given second-level administrative unit in 2018, the ratio between highest and lowest prevalence among age groups tended to be larger among males compared to females (median ratio across all SSA second-level administrative units of 14.4 for males, and 9.3 for females). For males, this ratio between highest and lowest estimated prevalence among age groups was smaller in Central SSA compared to other regions (median ratio of 8.3) and was largest in Western SSA (median ratio of 21.7). There was little regional difference for females. The sexes also differed in changes in this ratio between years, where it decreased over time for males (with a median ratio in 2000 of 52.7) but increased over time for females (median ratio in 2000 of 5.6). For complete age variation comparisons by second-level administrative unit, including uncertainty estimates, see Additional file 4.

Across SSA, the age group with the highest estimated prevalence in any given second-level administrative unit in 2018 was always between ages 35 and 54 years for males and between 30 and 49 years for females (Fig. 6). In 2018, males ages 45–49 years most commonly had the highest estimated prevalence across all age groups in a given second-level administrative unit, at 46.8% of second-level administrative units (1894 of 4043) from within 23 of 43 countries. Females ages 40–44 years had the highest estimated prevalence across age groups in 63.8% of second-level administrative units (2581 of 4043) in 31 of 43 countries. For both males and females, the age group with the highest estimated prevalence tended to vary more across Eastern SSA compared to other regions.

Within-country variation between second-level administrative units was relatively consistent across age groups. The ratio of maximum to minimum estimated prevalence among districts within each country was lowest for ages 35–39 years (median ratio of 4.3 across countries) and highest for ages 15–19 years (median ratio of 4.8 across countries) in 2018. Slightly larger differences were seen between age groups in Eastern and Southern SSA, with lower variation in middle-age groups and greater within-country variation in younger age groups. The maximum-to-minimum within-country prevalence ratio in Eastern SSA was lowest for adults ages 40–44 years (median ratio of 5.4 across Eastern SSA countries) and highest for adults ages 15–19 years (median ratio of 6.7 across Eastern SSA countries). These same age groups also represented the highest and lowest ratios in Southern SSA countries, with median values of 2.0 in adults ages 40–44 years and 2.8 in adults ages 15–19 years.

### Variation over time

**Fig. 7:**
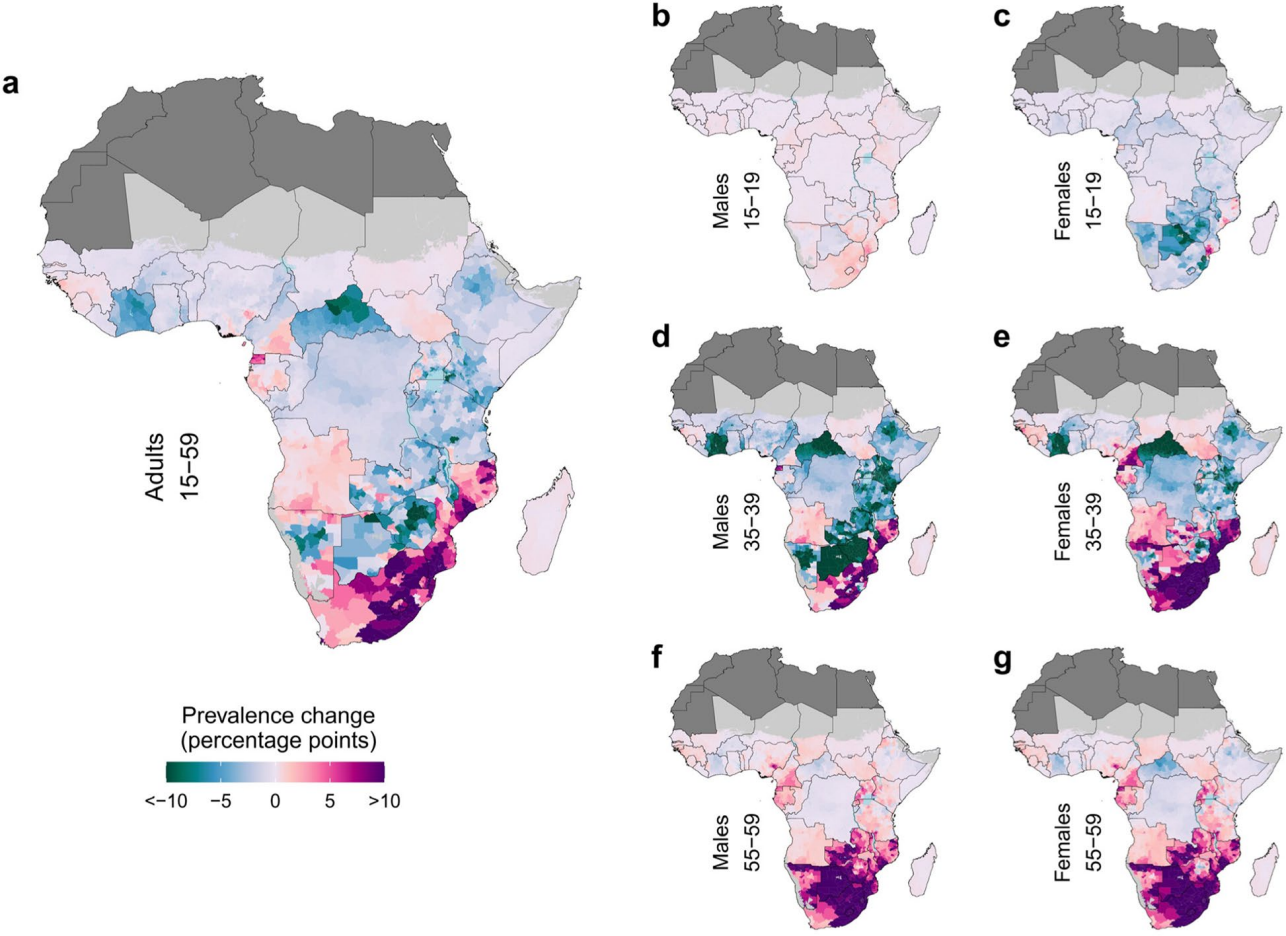
Change in HIV prevalence at the second administrative level between 2000 and 2018 for a subset of modeled demographic groups from the lower, middle, and upper age ranges: **a** all adults, ages 15–59 years; **b** males and **c** females ages 15–19 years; **d** males and **e** females ages 35–39 years; and **f** males and **g** females ages 55–59 years. Maps reflect national boundaries, land cover, lakes, and population; areas with fewer than ten people per 1 × 1 km, and classified as barren or sparsely vegetated, are colored light gray. Countries colored in dark gray were not included in the analysis

Estimated change in prevalence over time among all adults masked broad differences between specific age and sex groups (Fig. 7; Additional file 3: Figs. S35-S40). Large temporal changes were much more common when considering sexes and age groups, compared to all adults combined. Between the years 2000 and 2018, among all adults ages 15–59 years, estimated HIV prevalence increased by more than 5.0 percentage points in only 3.7% (151 out of 4043) of second-level administrative units across SSA and decreased by more than 5.0 percentage points in 7.9% (321 of 4043) of second-level administrative units. On the other hand, 37.7% (1523 of 4043) of second-level administrative units experienced an increase in estimated HIV prevalence greater than 5.0 percentage points in that timeframe in at least one sex and age group, and 70.9% (2867 of 4043) of second-level administrative units saw a decrease greater than 5.0 percentage points in at least one sex and age group.

The distribution of districts with large increases or decreases in prevalence over time also varied greatly by region. All regions saw a decrease of greater than 5.0 percentage points in estimated prevalence for at least one sex and age group in a majority of second-level administrative units between 2000 and 2018: 61.2% for Central SSA, (393 out of 642), 70.9% (1160 out if 1635) for Western SSA, 71.0% (1032 out of 1452) for Eastern SSA, and 90.1% (283 out of 314) for Southern SSA. However, Southern SSA also had a very high proportion of second-level administrative units seeing an increase of greater than 5.0 percentage points in that same time frame, at 92.0% (289 of 314), while only a minority of second-level administrative units saw similar increases in the other regions.

We found diverging overall trends between age groups over time, with greater decreases over time among younger age groups, and greater increases among older age groups. For example, for females ages 25–29 years, we found that estimated prevalence decreased by at least 1.0 percentage point in the year 2018 compared to 2000 in more than 73.3% of second-level administrative units in SSA (2965 of 4043) and increased by at least 1.0 percentage point in only 2.4% (99 of 4043) of all second-level administrative units. Conversely, among females ages 50–54 years, estimated prevalence decreased between 2000 and 2018 by at least 1.0 percentage point in just 11.8% (477 of 4043) of second-level administrative units but increased by at least 1.0 percentage point in 40.1% (1622 of 4043) of second-level administrative units. We found this trend to be similar across regions. For complete comparisons of prevalence over time for each second-level administrative unit, age, and sex, including uncertainty estimates, see Additional file 4.

## Discussion

The results of this study, the first to present age- and sex-specific HIV prevalence estimates across sub-Saharan Africa at local scales, emphasize the interactions of geographic and demographic differences in HIV prevalence, going beyond previous research focused on either aspect individually. Just as previous work demonstrated how much geographic variability is masked in national prevalence estimates [10], we show here that demographically aggregated estimates mask important variation in the age and sex distributions of HIV prevalence at a local level, which in turn provide much clearer insights into the evolution of the HIV epidemic in SSA.

Many intervention methods are commonly used in the fight against the HIV epidemic, and variation in their efficacy and implementation has likely contributed to the prevalence trends presented here. Cost-efficiency is a consistent priority and is generally maximized by using targeted, integrated interventions [50]. For example, HIV prevention via behavioral and biomedical interventions based on local prevalence rates, HIV testing, and treatment initiation may be priorities for some age groups [51], while long-term ART retention and comorbidity care may require more emphasis for others [52]. Barriers to access to care often differ between geographic and demographic groups, where in some cases barriers may be logistical (e.g., geographic isolation and programmatic fragmentation [53]) or social (e.g., lack of information, stigmatization, homophobia [54]), and require different intervention methods. Males and females are also often targeted using different points of contact. For example, HIV testing has been recommended for all females attending antenatal care clinics [55], whereas for males the provision of self-, home-based, and mobile testing compared to facility-based testing may be more useful for testing and subsequent uptake of care [56–58]. Effective targeting of these interventions requires local, demographically specific HIV burden information, such as provided in the estimates presented here. Countries may similarly use this burden information to prioritize subnational and demographically specific treatment needs. This resource may also be useful in program evaluation efforts and thus aid the development of more successfully tailored interventions.

Variation in the social determinants driving HIV incidence and mortality, and thus HIV prevalence, are also an important consideration when assessing inequalities in HIV prevalence between locations and demographic groups. While prevalence among females is consistently higher than prevalence among males, for example, these differences can be attributed to different exposure to risk factors (such as age at first sex between males and females, marital status) in different countries [59]. In addition to understanding local patterns in HIV prevalence, effective interventions also need to consider, if not focus directly on, locally important risk factors and determinants of HIV infection and mortality [60, 61].

Our estimates point to many local shifts in HIV prevalence over time. A multitude of factors can affect HIV prevalence trends at the local level over time, from local changes in prevention interventions to shifts in the overall demographics of an area, but one particularly important factor is local scale-up of ART [62, 63]. Increases in ART coverage and reduced treatment costs have repeatedly been associated with large demographic shifts among people living with HIV [64] due to its success in reducing HIV mortality, leading to greatly increasing numbers of people living with HIV over the age of 50 years; our results reflect this trend. Given evidence pointing to differences between younger and older ART patients in rates of CD4 cell count decline [65], immune reconstitution rates [66], and risk of associated non-communicable diseases [67, 68], among other health metrics [69], it is necessary that treatment plans for older patients be specifically tailored for their age group. Our results highlight those locations with large existing populations of people living with HIV for ages 50–59 years, and those seeing rapid growth of HIV prevalence in that demographic group. At the same time, the minimal change in estimated prevalence over time among the youngest age groups suggests that continued and even expanded efforts in HIV prevention for adolescents and young adults still need to be maintained as a priority across the continent.

Despite the significant progress made through this analysis in describing HIV burden in SSA, prevalence estimates mask complex and varied relationships between HIV incidence and mortality, as well as migration and seasonal mobility. It is difficult to determine, for example, if a dramatic decrease in HIV prevalence in an area is due to reduced incidence, increased mortality, or differences in the immigration and emigration rates of HIV^+^ and HIV^-^ individuals. Primary data for all three of these metrics are not widely available for SSA, adding additional complexity to the interpretation of our estimates. Importantly, no estimates of these indicators are consistently available at local scales for specific demographic groups. Furthermore, local data related to diagnosis, treatment, and viral suppression rates are also limited, despite these metrics lying at the heart of the UNAIDS 95-95-95 goals [4]. While very informative, difficulties can still arise in intervention decision-making built around HIV prevalence estimates alone, without understanding their underlying drivers. Improved surveillance of HIV prevalence, incidence, and mortality, combined with reliable population and migration estimates and information on local programs, are necessary to fully understand the complexities of the region’s HIV epidemic. Clearly, even with the development of more comprehensive burden information, any modeled estimates should only be used for intervention purposes in conjunction with local program knowledge.

### Methodological advantages and limitations

The methods used in this analysis build upon those previously used by Dwyer-Lindgren et al. to model adult HIV prevalence [10]. While this analysis does improve upon and have advantages over the previous methods in some ways, it faces some of the same, as well as some new limitations. As with the previous study, and as with all modeling studies, the quality of our estimates is highly dependent on the quality and coverage of our input data. Despite constructing a large database of HIV prevalence data, coverage gaps and small sample sizes in some locations can be associated with imprecision and/or large uncertainty intervals in some of our prevalence estimates (Additional file 3: Figs. S27-S34). Additionally, the location information associated with the data compiled for this analysis is subject to some error. In order to protect respondent confidentiality, most surveys that collect GPS coordinates perform some type of random displacement on those coordinates prior to releasing data for secondary analysis: for example, GPS coordinates for Demographic and Health Surveys (DHS) are displaced by up to 2 km for urban clusters, up to 5 km for most rural clusters, and up to 10 km in a random 1% of rural clusters [34]. Past research has found that displacement can degrade the predictive power of a geostatistical model, however this effect was found to be modest, and researchers concluded that relatively accurate mapping can be undertaken at a 5 × 5-km resolution even with GPS displacement [70].

The approximate integration method we use in this analysis better handles uncertainty estimation and easily accommodates not only polygon data but age-aggregated data as well, compared to the polygon resampling method that has been used elsewhere [10, 71, 72]. At the same time, given the large number of dimensions being modeled, as well as the high data input count produced by our data disaggregation technique, we found that current matrix packages, as well as our computational facilities, could not accommodate a Gaussian process that accounted for the covariance of a complete space-time-age-sex Kronecker product. We therefore focused on the interactions between space, time, age, and sex that we believed would be most relevant in terms of capturing important variability in these dimensions, within our computational abilities. Our modeling strategy also assumed no difference in the probability that an HIV^+^ versus an HIV^-^ pregnant woman would access antenatal care and therefore be included in ANC surveillance.

Due to limited data availability, we delineated estimates in this analysis using a male/female binary. We recognize that this approach does not allow for investigation of HIV prevalence among gender and sex diverse people, despite the disproportionate burden of HIV commonly seen among these populations [73]. Further, we recognize that many data sources do not provide the option to select a sex other than “male” or “female,” gender options beyond “man” or “woman,” and often conflate gender with sex. In the future, we hope that high-quality data on HIV prevalence for gender and sexual diverse people will be more widely available, so we can produce estimates beyond females and males.

We note that our results include unprecedentedly high prevalence estimates for certain population subsets. In most cases, we do not believe these estimates are implausible. For example, we estimated prevalence among middle- and older-aged females to be up to 59.2% [45.9–73.0%] in Umgungundlovu in KwaZulu-Natal, South Africa in 2018. Previous research has estimated prevalence for females adults of all ages combined in Umgungundlovu in 2017 to be 46.6% [43.8–49.5%] [74]. As we have shown that prevalence in middle- and older-aged females tended to be higher than all-ages prevalence, we believe our estimates for middle- and older-aged females during this time period in this location to be reasonable, especially with uncertainty intervals taken into consideration. In rare cases, however, our methods yielded estimates which we were unable to support through the literature. For example, for males ages 35–39 and 40–44 years in Nyatike in Migori, Kenya, we estimated prevalence in the year 2000 to be 77.8% [50.2–100.0%] and 78.7% [50.0–100.0%], respectively. It is unlikely true prevalence in that area and year was this high (though given the large uncertainty intervals associated with these values, it is probable that true prevalence does fall within those ranges). We note, however, that the high estimates in this area and surrounding second-level administrative units were predominantly associated with the earlier years in our time series—we believe the more recent estimates in Nyatike to be more realistic [75]. In these locations, decreases in prevalence over time may therefore also be overestimated. These instances were rare.

A combination of data limitations and model complexity ultimately led to large uncertainty intervals around our estimates. Given that our 95% coverage estimates in model validation were consistently higher than expected (Additional file 3: Figs. S14-S16), this indicates that these uncertainty intervals may be larger than appropriate. Wide uncertainty can limit the utility of our estimates in terms of informing HIV policies, and reducing this uncertainty through improved data coverage will be an important consideration in future iterations of this model. We were also unable to account for all sources of uncertainty such as uncertainty in the WorldPop estimates used in many stages of our modeling and estimation processes and uncertainty in covariates.

## Conclusions

HIV continues to impose enormous human and financial costs [3] on SSA, decades since its emergence. Financial and logistical disruptions and discontinuities due to the impacts of COVID-19, as well as changes in ART adherence, are likely to present new barriers [21, 76] to the UNAIDS 95-95-95 goals [4]. This analysis provides important insight into the nuances of HIV burden in SSA, offering information that is critical to the development of targeted interventions.

## Supplementary Information

**Additional file 1: Supplemental information.1.** Compliance with the Guidlines for Accurate and Transparent Health Estimates Reporting (GATHER). **2.** HIV data sources and data processing. **3. **Covariate and auxiliary data. **4.** Statistical model. **5.** References. 

**Additional file 2: Supplemental tables.Table S1.** HIV seroprevalence survey data. **Table S2.** ANC sentinel surveillance data. **Table S3.** HIV and covariates surveys excluded from this analysis. **Table S4.** Sources for pre-existing covariates. **Table S5.** HIV covariate survey data. **Table S6.** Fitted model parameters.

**Additional file 3: Supplemental figures.Figure S1.** Prevalence of male circumcision. **Figure S2.** Prevalence of signs and symptoms of sexually transmitted infections. **Figure S3.** Prevalence of marriage or living as married. **Figure S4.** Prevalence of partner living elsewhere among females. **Figure S5.** Prevalence of condom use during most recent sexual encounter. **Figure S6.** Prevalence of sexual activity among young females. **Figure S7.** Prevalence of multiple partners among males in the past year. **Figure S8.** Prevalence of multiple partners among females in the past year. **Figure S9.** HIV prevalence predictions from the boosted regression tree model. **Figure S10.** HIV prevalence predictions from the generalized additive model. **Figure S11.** HIV prevalence predictions from the lasso regression model. **Figure S12.** Modeling regions. **Figure S13.** Age- and sex-specific vs. adult prevalence modeling. **Figure S14.** Data sensitivity. **Figure S15.** Model specification validation. **Figure S16.** Modeled and re-aggregated adult prevalence comparison. **Figure S17.** HIV prevalence raking factors for males. **Figure S18.** HIV prevalence raking factors for females. **Figure S19.** Age-specific HIV prevalence in males, 2000. **Figure S20.** Age-specific HIV prevalence in females, 2000. **Figure S21.** Age-specific HIV prevalence in males, 2005. **Figure S22.** Age-specific HIV prevalence in females, 2005. **Figure S23.** Age-specific HIV prevalence in males, 2010. **Figure S24.** Age-specific HIV prevalence in females, 2010. **Figure S25.** Age-specific HIV prevalence in males, 2018. **Figure S26.** Age-specific HIV prevalence in females, 2018. **Figure S27.** Age-specific uncertainty interval range estimates in males, 2000. **Figure S28.** Age-specific uncertainty interval range estimates in females, 2000. **Figure S29.** Age-specific uncertainty interval range estimates in males, 2005. **Figure S30.** Age-specific uncertainty interval range estimates in females, 2005. **Figure S31.** Age-specific uncertainty interval range estimates in males, 2010. **Figure S32.** Age-specific uncertainty interval range estimates in females, 2010. **Figure S33.** Age-specific uncertainty interval range estimates in males, 2018. **Figure S34.** Age-specific uncertainty interval range estimates in females, 2018. **Figure S35.** Change in HIV prevalence in males, 2000-2005. **Figure S36.** Change in HIV prevalence in females, 2000-2005. **Figure S37.** Change in HIV prevalence in males, 2005-2010. **Figure S38.** Change in HIV prevalence in females, 2005-2010. **Figure S39.** Change in HIV prevalence in males, 2010-2018. **Figure S40.** Change in HIV prevalence in females, 2010-2018. **Figure S41.** Space mesh for geostatistical models.

**Additional file 4: Supplemental results.1.** README. **2.** Prevalence range across districts. **3.** Prevalence range between sexes. **4.** Prevalence range between ages. **5.** Age-specific district ranges.

## Data Availability

The findings of this study are supported by data available in public online repositories and data publicly available upon request of the data provider. Details regarding the data sources used and their availability can be found in Additional file 2: Supplemental Tables 1-5 and online via the Global Health Data Exchange (https://ghdx.healthdata.org/record/ihme-data/sub-saharan-africa-hiv-prevalence-geospatial-estimates-2000-2018). Estimates can also be further explored through the Global Health Data Exchange, as well as via our online visualization tool (http://vizhub.healthdata.org/lbd/hiv-prev-disagg). Administrative boundaries were modified from the Database for Global Administrative Areas (GADM) dataset [77]. Populations were retrieved from WorldPop [32]. This study complies with the Guidelines for Accurate and Transparent Health Estimates Reporting (GATHER) recommendations [31]. All maps and figures presented in this study are generated by the authors; no permissions are required for publication. All computer code is available online and can be found at (https://github.com/ihmeuw/lbd/tree/hiv_prev-africa-2020).

## Abbreviations

AIDS: Acquired immune deficiency syndrome
ANC: Antenatal care
ART: Antiretroviral therapy
GATHER: Guidelines for Accurate and Transparent Health Estimates Reporting
GBD: Global Burden of Disease
GPS: Global positioning system
HIV: Human immunodeficiency virus
SDGs: Sustainable Development Goals
SSA: Sub-Saharan Africa
TMB: Template Model Builder
UNAIDS: Joint United Nations Programme on HIV/AIDS
