# Supplementary material for: Mapping age- and sex-specific HIV prevalence in adults in sub-Saharan Africa, 2000–2018
Source: BMC Med. 2022 Dec 19;20:488. doi: 10.1186/s12916-022-02639-z (PMC9760541; doi:10.1186/s12916-022-02639-z)
Supplement: Supplementary file 1 — Additional file 1: Supplemental information.1. Compliance with the Guidlines for Accurate and Transparent Health Estimates Reporting (GATHER). 2. HIV data sources and data processing. 3. Covariate and auxiliary data. 4. Statistical model. 5. References. [file 12916_2022_2639_MOESM1_ESM.pdf]

Additional File 1: Supplemental Information

Mapping age- and sex-specific HIV prevalence in adults in sub-Saharan Africa, 2000–2018

Contents

1 Compliance with the Guidelines for Accurate and Transparent Health Estimates Reporting (GATHER) ..3
2 HIV data sources and data processing 6
2.1 Seroprevalence surveys 6
2.1.1 Data identification strategy 6
2.1.2 Data processing for microdata 6
2.1.3 Data processing for reports 7
2.2 Antenatal care (ANC) sentinel surveillance 7
2.2.1 Data sources 7
2.2.2 Data processing....8
2.3 Polygon and age-aggregated data processing 9
3 Covariate and auxiliary data 10
3.1 Pre-existing covariates....10
3.2 Covariates constructed for this analysis 11
3.2.1 Covariate selection criteria and definitions....11
3.2.2 Covariate data....13
3.2.3 Covariate modeling....16
3.3 Administrative boundaries 17
3.4 Gridded population....17
4 Statistical model....17
4.1 Covariate stacking....17
4.2 Geostatistical model 18
4.2.1 Model description....18
4.2.2 Model fitting and prediction....23
4.3 Model validation 25
4.3.1 Validation strategy....25
4.3.2 Sensitivity analyses 26

4.3.3 Comparisons to adult prevalence estimates ... 29
4.4 Post-estimation ... 29
4.4.1 Aggregation to first- and second-level administrative subdivisions ... 29
4.4.2 Calibration to Global Burden of Disease 2019 ... 30
4.4.3 Calculating people living with HIV (PLHIV) ... 31
5 References ... 31

1 Compliance with the Guidelines for Accurate and Transparent Health Estimates Reporting (GATHER)

Item #	Checklist item	Description of Compliance
Objectives and funding		
1	Define the indicator(s), populations (including age, sex, and geographic entities), and time period(s) for which estimates were made.	Precision public health and HIV section
2	List the funding sources for the work.	Acknowledgments section
Data Inputs		
For all data inputs from multiple sources that are synthesized as part of the study:		
3	Describe how the data were identified and how the data were accessed.	Methods; Additional File 1: Sections 2.1, 2.2, 3.1, 3.2
4	Specify the inclusion and exclusion criteria. Identify all ad-hoc exclusions.	Additional File 1: Sections 2.1, 2.2, 3.1, 3.2, Additional File 2: Table S3
5	Provide information on all included data sources and their main characteristics. For each data source used, report reference information or contact name/institution, population represented, data collection method, year(s) of data collection, sex and age range, diagnostic criteria or measurement method, and sample size, as relevant.	Additional File 2: Tables S1-2,4-5, https://ghdx.healthdata.org/record/ihme-data/sub-saharan-africa-hiv-prevalence-geospatial-estimates-2000-2018
6	Identify and describe any categories of input data that have potentially important biases (e.g., based on characteristics listed in item 5).	Methods
For data inputs that contribute to the analysis but were not synthesized as part of the study:		
7	Describe and give sources for any other data inputs.	Methods; Additional File 1: Sections 3.1, 3.3, 3.4;
For all data inputs:		
8	Provide all data inputs in a file format from which data can be	Available through https://ghdx.healthdata.org/rec

	efficiently extracted (e.g., a spreadsheet rather than a PDF), including all relevant meta-data listed in item 5. For any data inputs that cannot be shared because of ethical or legal reasons, such as third-party ownership, provide a contact name or the name of the institution that retains the right to the data.	ord/ihme-data/sub-saharan-africa-hiv-prevalence-geospatial-estimates-2000-2018
Data analysis		
9	Provide a conceptual overview of the data analysis method. A diagram may be helpful.	Methods; Figure 2
10	Provide a detailed description of all steps of the analysis, including mathematical formulae. This description should cover, as relevant, data cleaning, data pre-processing, data adjustments and weighting of data sources, and mathematical or statistical model(s).	Methods; Additional File 1: Sections 2-4
11	Describe how candidate models were evaluated and how the final model(s) were selected.	Additional File 1: Section 4.3
12	Provide the results of an evaluation of model performance, if done, as well as the results of any relevant sensitivity analysis.	Additional File 1: Section 4.3
13	Describe methods for calculating uncertainty of the estimates. State which sources of uncertainty were, and were not, accounted for in the uncertainty analysis.	Methods; Additional File 1: Sections 3.2, 4.4
14	State how analytic or statistical source code used to generate estimates can be accessed.	Available through https://github.com/ihmeuw/lbd/tree/hiv_prev-africa-2020
Results and Discussion		

15	Provide published estimates in a file format from which data can be efficiently extracted.	Available through https://ghdx.healthdata.org/record/ihme-data/sub-saharan-africa-hiv-prevalence-geospatial-estimates-2000-2018
16	Report a quantitative measure of the uncertainty of the estimates (e.g. uncertainty intervals).	Results; Figure 4; Additional File 3: Figs. S27-34
17	Interpret results in light of existing evidence. If updating a previous set of estimates, describe the reasons for changes in estimates.	Results; Methods
18	Discuss limitations of the estimates. Include a discussion of any modelling assumptions or data limitations that affect interpretation of the estimates.	Discussion; Methods

2 HIV data sources and data processing

2.1 Seroprevalence surveys

2.1.1 Data identification strategy

We identified HIV seroprevalence surveys in sub-Saharan Africa (SSA) through a review of all surveys in the Demographic and Health Survey (DHS), AIDS Indicator Survey (AIS), Multiple Indicator Cluster Survey (MICS) series, and other surveys listed in the Global Health Data Exchange[1]; surveys included in the national HIV estimates files from UNAIDS[2]; and surveys listed in the US Census Bureau HIV/AIDS Surveillance Database[3]. For a survey to be considered for this analysis, we required that the survey reported HIV blood test results, sampled from the general adult population, and contained geographic information more refined than country level. For surveys with no microdata available we used reports if they included sample size, or uncertainty intervals from which sample size could be derived. Our desired age range was 15–59 years, but we also included survey reports that recorded prevalence for age spans within that range. The surveys used in this analysis are listed in Additional File 2: Table S1 and visualized in Figure 1. We additionally considered data sources identified through literature review; however, because data from these sources predominantly did not match our inclusion criteria related to age distribution (see section 2.1.3 below), we elected to exclude all literature review data from this model. Other survey data exclusions are detailed in Additional File 2: Table S2.

2.1.2 Data processing for microdata

To prepare survey microdata for analysis, we first subset the data to the age range of interest, 15–59 years, and dropped any data that were not sex-specific. For data coded by gender rather than sex, we treated these data as if they were sex-specific rather than gender-specific. We then dropped rows for individuals explicitly listed as not tested or where the blood samples were marked as lost or rejected (insufficient sample volume, tip broken, etc.). Inconclusive and indeterminate test results were coded as a negative test result. After subsetting according to these conditions, we further dropped any microdata missing an HIV test result, survey weight, or geographic information or due to the GPS coordinates being located more than 10 km outside of the country border. Coordinates within 10 km of the country border were snapped to be approximately 1 km inside the nearest border of the specified country.

We then aggregated the individual-level microdata into sex-specific five-year age bins (15–19, 20–24, 25–29, 30–34, 35–39, 40–44, 45–49, 50–54, 55–59; hereafter termed ‘ages’) to the finest possible spatial resolution available, ideally a latitude and longitude pair representing the location of the survey cluster (point-level data). The interview date for each specific location was calculated as the median of the individual-level interview dates. Where point-level referencing was not available, we geolocated

survey microdata to the smallest geographical area (termed 'polygon') possible. Individual-level sample weights were used when calculating prevalence, and the effective sample size for each prevalence estimate was estimated via the Kish approximation[4], which accounts for differences in the underlying selection probability within a sample.

2.1.3 Data processing for reports

In instances where individual-level microdata were not available, we used summary reports, given that the estimates reported were similar in nature to what we would calculate from the microdata. We used the median months of the reported data collection periods as the interview dates to align with the extracted microdata. If sample sizes were not included in the report, we estimated them from the reported confidence intervals, assuming that a Normal approximation was used to generate 95% confidence intervals. In both instances, sample sizes were further adjusted by multiplying the median design effect (ratio of effective sample size to observed sample size) calculated in the microdata as described above. We only used reports with sex-specific estimates. Summary reports only provide estimates aggregated across age; we included only those that completely covered either some or all the 5-year age bins within the 15–59 year age range being modeled. Because of their incongruity with our methods for modeling age-aggregated data (detailed in Additional File 1: Section 2.3), we did not include reports extending below 15 years or above 59 years, or any reports incompletely covering any of our ages. For example, we included reports covering age ranges such as 15–59 years, or 15–49 years, but excluded reports covering age ranges such as 15–64 years, or 18–24 years.

2.2 Antenatal care (ANC) sentinel surveillance

2.2.1 Data sources

In addition to general population surveys, we used antenatal care (ANC) sentinel surveillance data, which measure HIV prevalence among pregnant females attending antenatal care clinics. Most of these raw data came from national Spectrum files that were developed by a country team of experts and compiled and shared by the UNAIDS secretariat[2]. These files include the HIV prevalence and sample size of ANC sentinel surveillance and routine testing for various sites and years. We only used the sentinel surveillance estimates for our analysis.

We supplemented this data with ANC sentinel surveillance country reports. In general, the reports contained the same information as the Spectrum files, but there was some additional information in the reports and some discrepancies compared to the Spectrum files. The additional information included additional sites, additional years for given sites, and more precise prevalence estimates. In instances

where there were discrepancies for a given site-year, we elected to use the source where HIV prevalence was closest to the average prevalence of surrounding years for the same site.

Four countries had a notably large number of discrepancies between the Spectrum files and the ANC reports. The Zambia Spectrum files recorded prevalence for the 15–39 years age range, while the reports recorded prevalence for the 15–44 years age range. In this case, we elected to use the Spectrum files because they had better data coverage in terms of number of site-years. There were also many discrepancies in Central African Republic, Côte d'Ivoire, and Zimbabwe; we were unable to identify a specific reason for these discrepancies and elected to use data from the Spectrum files only for these countries. We investigated the ANC reports to determine if site names in the Spectrum files represented hospitals, cities, or administrative subdivisions. We then used various mapping websites to find geographic information related to these sites. For hospitals and cities/towns that are less than 25 km^2 in area, we used a central GPS coordinate, and for administrative subdivisions we used a polygon of the area. Some hospital sites had a city or town name rather than a hospital name. In those instances, we searched for a hospital in the given city or town and used that hospital's GPS coordinates. If there were multiple hospitals in the area but they were less than 5 km apart, we used the GPS coordinates of the midpoint of the hospitals. If no hospitals were found in the area but the corresponding region was less than 25 km^2 in area, we used the central GPS coordinate. Sites that could not be geolocated because none of these conditions were met were excluded from further analyses.

2.2.2 Data processing

To prepare the ANC data for analysis, we compiled the HIV prevalence and sample size data from the Spectrum files and the ANC reports, and the site geographic information – either GPS coordinates or polygons for administrative subdivisions – into one dataset. After thoroughly inspecting the data, we decided to exclude the following data from our analysis:

- Hospital-level sites were dropped from Congo in 2011 (23 site-years) and Guinea-Bissau in 2003, 2005, 2010, and 2014 (10 site-years) because the data aggregated by administrative subdivisions had better temporal coverage.
- We dropped administrative subdivisions that were masked by a different level of administrative subdivisions (8 site-years), defaulting to the level that would give better temporal coverage.
- We determined that 181 site-years were outliers based on inspection of site-level time trends and undue influence on model results, and these were dropped from the analysis.

- Data from sites that could not be geolocated were also dropped (96 sites). Additionally, in the Spectrum files, data from 12 site-years labeled as sentinel surveillance we suspect are actually routine testing, and we excluded these from the analysis. In some cases, 'default' sample sizes were reported for all sites and certain years in a given country (typically $N = 300$). In cases where measured sample sizes were available for these affected sites in two or more other years, we replaced the 'placeholder' sample size with the site-specific median from across measured years. In cases where reported sample sizes were not available for other years, or where median values clearly conflicted with site-specific trends in sample size over time, the 'placeholder' sample size was retained. In the end, we adjusted sample sizes in this way for select data in five countries, five years, and 57 sites, equating to 11 country-years and 146 site-years in total.

The ANC data included in this analysis are listed in Additional File 2: Table S2 and visualized in Figure 1.

2.3 Polygon and age-aggregated data processing

To incorporate observations geolocated to the polygon level as well as age-aggregated observations into our model, we disaggregated these data to mimic point and/or age-specific data. Specifically, we disaggregated each of these given observations to be location- and/or age-specific. For each polygon, we generated points at the centroid of each pixel falling within that polygon and replicated that observation's HIV prevalence and sample size at the location of each centroid. Age-aggregated data were similarly disaggregated by replicating HIV prevalence and sample size once for each age covered in the given age-aggregated observation's age range. In the cases of age-aggregated polygon data, these two processes were combined. Next, each of the disaggregated, location- and age-specific rows of data associated with a given aggregated observation were assigned weights (w_j) proportional to the age- and sex-specific population at that location for the given year, derived from WorldPop[5]. For ANC data, ages and locations within an ANC observation were weighted by births rather than population. The number of births for a given age and location was calculated as the product of the location-, age-, and sex-specific population again derived from WorldPop[5], and the national fertility rate, derived from GBD 2019 estimates[6]. Weights per observation all summed to one. Age-specific point observations were each assigned a weight of one.

To reduce the computational burden imposed by this method in terms of the large number of locations and ages generated, in cases where for at least one location and/or age (j) within an observation,

$$w_j < \frac{1}{2} \cdot 1/\max(j),$$

we successively dropped the lowest-weighted locations and/or ages in that observation, until a maximum of 1% of the observation's weight was dropped. Remaining locations and/or ages within that observation were then reweighted to maintain a total observation weight of one. Age-specific point observations were each given a weight of one. This ultimately allowed us to retain ≥99% of our observation weight of while removing 42.2% of pixel-ages, greatly mitigating the computational burden on this model.

3 Covariate and auxiliary data

3.1 Pre-existing covariates

Mirroring the previously published adult HIV prevalence model[7], this analysis included five pre-existing covariates: travel time to the nearest settlement of more than 50,000 inhabitants, total population, night-time lights, urbanicity, and malaria incidence. These variables were selected from among available gridded datasets for SSA because they are factors, or proxies for factors, that previous literature has identified to be associated (not necessarily causally) with HIV prevalence. The first four variables were included as measures or proxies for connectedness and urbanicity, as HIV historically spread through SSA along travel routes[8, 9] and is typically found to be higher in more urban compared to more rural locations. Malaria incidence was selected based on prior evidence relating higher malaria incidence rates to higher prevalence of HIV at the population level[10, 11]. Sources for these data are given in Additional File 2: Table S4. These covariates underwent spatial and temporal processing in preparation for their inclusion in analysis.

Spatial processing involved resampling the input covariate raster to align the spatial resolution of the covariate to the 5 x 5-km resolution used in modeling. For covariates that were originally at a finer resolution, we resampled the raster by taking the neighborhood average (travel time to the nearest settlement of more than 50,000 inhabitants, night-time lights, and urbanicity) or sum (total population) of the finer covariate raster to produce one at a 5 x 5-km resolution. Malaria incidence was natively at a 5 x 5-km resolution and thus did not require additional spatial processing.

Temporal processing was required in instances where the original temporal resolution of the covariate was anything other than annual. To resolve from a coarser time period to an annual time period, we filled the intervening years with the value from the nearest neighboring year (urbanicity) or using an exponential growth rate model (total population). Night-time lights and malaria incidence were provided at a one-year temporal resolution and did not require interpolation. As travel time to the nearest settlement of more than 50,000 inhabitants was available only for a single representative year

(2015), this covariate was set to be unchanged over time. After interpolation, night-time lights and urbanicity were still missing the most recent years of the 2000–2018 analysis period, and in these instances, we filled out the end of the time-series carrying forward the most recent year without modification.

3.2 Covariates constructed for this analysis

3.2.1 Covariate selection criteria and definitions

In addition to the five pre-existing covariates, we constructed eight additional covariates for this analysis that were updated from the previously published adult HIV prevalence model[7]. Numerous studies have been conducted in SSA on risk and protective factors for HIV infection, and these factors commonly include sexual behavior and factors that are thought to influence the transmission of HIV during sexual intercourse[12]. Potential covariates were informed by past literature and required to have a demonstrated association with HIV prevalence, though not necessarily a causal relationship. Furthermore, our selection of covariates depended on having adequate data coverage from data sources that could be readily extracted. In total, eight covariates were constructed:

- Prevalence of male circumcision, including medical or traditional circumcision ('male circumcision');
- Prevalence of self-reported STI symptoms (genital discharge and/or genital ulcer/sore) in the last 12 months ('STI symptoms');
- Prevalence of marriage or living with a partner as married ('in union');
- Prevalence of one's current partner living elsewhere among females ('partner away');
- Prevalence of condom use at last sexual encounter within the last 12 months ('condom last time');
- Prevalence of sexual activity among young females ('had intercourse');
- Prevalence of males reporting multiple sexual partners within the last year ('multiple partners in year');
- Prevalence of females reporting multiple sexual partners within the last year ('multiple partners in year').

The notion that male circumcision has a protective effect against acquiring HIV was first proposed in 1986, and since then more than 30 cross-sectional studies have found the prevalence of HIV to be significantly higher in uncircumcised males, as well as numerous prospective studies that have shown a protective effect ranging from 48% to 88%[13]. In 2005, following the interruption of a randomized,

controlled trial of male circumcision in South Africa that showed a 60% protective effect of circumcision, WHO and UN agencies first acknowledged evidence of male circumcision's protective effect[14]. Following these declarations, voluntary medical male circumcision clinics (VMMC) emerged as an HIV prevention strategy in 15 countries in Eastern and Southern Africa with high HIV prevalence and low levels of male circumcision[15]. Given male circumcision's linkage to HIV in the scientific literature, many surveys record self-reported circumcision status. The modeling of male circumcision estimates in this study closely mirrors the methods recently published by Cork *et al*[16]. Here, we extend the analysis to include estimates for the year 2018, as well as additional countries included in this study but not in the previous work.

Coinfection of HIV with viral and bacterial sexually transmitted infections (STIs), most notably herpes simplex virus type 2, is a well-studied mechanistic factor associated with higher risk of HIV acquisition[17]. STIs are thought to have been especially important risk factors during the early stages of the epidemic when infections were concentrated in high-risk groups, though researchers have since argued STIs are also critical in advanced stages[18]. Due to the association between STI prevalence, sexual behavior, and HIV, most survey series detail the self-reported presence of STI symptoms, facilitating its inclusion as an HIV covariate in this analysis.

Marital status represents a structural factor that, while distal to HIV exposure, has been associated with the number and type of sexual partners, as well as with HIV status[19, 20]. It has been postulated that the relationship between an individual's marital status and the number of sexual relationships regulates the protective effect of marriage on the risk of HIV infection[21]. Marital status is a readily available indicator in household surveys more generally.

The frequency with which a partner has slept away from home during the past year is an indicator of the mobility of male partners, and studies have found that mobility confers an increased risk for HIV[22]. Part of the rapid spread of HIV in SSA has been attributed to occupations that consist of geographical mobility, especially truck drivers, who are identified as high-risk for acquiring and spreading HIV[23]. Many surveys ask females if their partner has lived away from home in the past year, and we use these responses as a proxy for occupational mobility.

Condom use is a sexual behavior factor that is protective against acquiring HIV. Condoms are often presented as the most effective HIV prevention method of sexual transmission of the disease[24]. Though it is difficult to measure accurately how often condoms are used in sexual encounters, most

surveys report on the use of condoms in last sexual intercourse, a readily available proxy for overall condom use.

An early age at sexual debut may be associated with the number of lifetime sexual partners, which is considered a key risk factor for contracting HIV[21]. Furthermore, early age at sexual debut has been shown to be associated with numerous other risk factors for HIV acquisition, such as STI prevalence and decreased condom use[25]. For young females, the initiation of sexual activity is the first important determinant of potential viral exposure, and delayed sexual debut has been associated with decreased risk of HIV acquisition[26]. Given these relationships between HIV and age of sexual debut, and the relative ease of acquiring self-reported sexual status, we constructed an indicator for whether young (ages 15–24) females have had intercourse.

An individual's number of sexual partners correlates with HIV risk, and past studies have found a relationship between the number of sexual partners and HIV prevalence[27]. The number of sexual partners is thought to have been an especially important factor in the early stages of an epidemic, though past research has determined it remains a key risk factor in advanced stages[18]. Surveys often ask males and females their number of partners in the past year, and we used these responses to construct a proxy for multiple concurrent sexual relationships. Separate covariates were constructed for males and females given the well-documented discrepancy in the number of partners reported by males as compared to females[28].

3.2.2 Covariate data

3.2.2.1 Covariate data identification strategy

We reviewed major survey series (Demographic and Health Surveys [DHS]; Multiple Indicator Cluster Surveys [MICS]; AIDS Indicator Surveys [AIS]; Malaria Indicator Surveys [MIS]; Performance, Monitoring, and Accountability Surveys [PMA]; Reproductive Health Surveys [RHS]; and Living Standards Measurement Surveys [LSMS]) to identify surveys in SSA that contained relevant variables. We supplemented this initial list of surveys with country-specific surveys identified in the Global Health Data Exchange[1] and with a cross-check of all surveys extracted for HIV prevalence. We included surveys that contain variables related to one or more of the covariate indicators (including any time restrictions inherent to the indicator definition) and contained geographic information at a subnational level.

For all indicators except for 'had intercourse,' we required a survey to sample the general adult (ages 15–49) population. This age range was chosen for the covariates primarily due to data availability. For 'had intercourse', a survey only had to sample the general young female (ages 15–24) population to be

included. Because covariates were not modeled to be age-specific, more discerning age range requirements were not required of the surveys used in these models.

Because of variations we identified in the way these questions were asked across surveys, we tracked the skip logic and question format for all surveys including STI symptoms and/or the sexual activity indicators. This helped us identify surveys for which the question format was so substantively different from others as to require special handling or exclusion (e.g., questions asked without a time restriction for indicators that require a response from the last 12 months). We excluded select surveys because of these irreconcilable question variations, incomplete sampling (e.g., a specific age range or subpopulation), or untrustworthy or outlier data (as determined by the survey administrator or by inspection). The surveys used for these covariates are listed in Additional File 2: Table S5.

3.2.2.2 Covariate data processing for microdata

To prepare the survey microdata for analysis, we first constructed final indicators from the raw variables included in the survey data:

- For 'STI symptoms,' we constructed a symptoms indicator that was true if a respondent reported either genital discharge or a genital sore/ulcer in the last 12 months, missing if either individual symptom was missing, and false if both symptoms were reported in the negative.
- For 'in union,' we constructed an indicator that was true for all respondents who reported being either currently married or living with a partner, false for any other marital status response, and missing if the marital status response was missing.
- For 'multiple partners in year', we used the reported number of sexual partners within the last 12 months to construct a binary indicator that was true for any respondent reporting two or more partners and false for any respondent with 0 or 1 partners (including respondents who had never had intercourse).
- The other indicators were extracted from the survey microdata in their final form and required no additional construction.

For each indicator, we subset the data to the desired age range (15–24 years for 'had intercourse', 15–49 years for all other indicators). For 'STI symptoms' we additionally restricted the sample to respondents who reported having had intercourse, while for 'partner away' we additionally restricted the sample to respondents currently 'in union'. We dropped any rows with missing responses or sample weights. For indicators where we model males and females together ('STI symptoms,' 'in union,' 'condom last time'), we dropped any surveys that did not interview both males and females. Any

observations missing geographic information or with inconsistent geographic information (i.e., points more than 10 km from the nearest specified country border) were also dropped.

Finally, we aggregated the weighted individual-level microdata for each indicator to the finest possible spatial resolution available. We did not collapse or model covariate data according to specific age-bins due to data limitations. As in Dwyer-Lindgren *et al.*[7], data for the covariate 'multiple partners per year,' was collapsed separately for males and females. 'Male circumcision' and 'prevalence of sexual activity among young females' included data exclusively for males or females, respectively, but for all other covariates, data were not collapsed to be sex-specific. Data were geolocated to latitude and longitude at the survey cluster level wherever possible, and to the smallest possible polygon available otherwise. As with the HIV prevalence data, we calculated the effective sample size for each spatial aggregation using the Kish approximation[4].

3.2.2.3 Covariate data processing for reports

For 'male circumcision,' we also included summary reports for surveys where individual-level microdata were not available. We followed the same methods for report data processing as reported in Cork *et al*[16]. We chose not to include summary reports for other covariates. For 'STI symptoms,' the estimates included in reports used a different construction of the variable than that which we built from the microdata, making the reports incompatible with the microdata. For the sexual activity indicators, we decided against summary report extraction due to the significant number of surveys we were able to extract at the microdata level and the scarcity of reports for most of these indicators.

3.2.2.4 Covariate data processing for polygons

As with HIV prevalence data, wherever possible, covariate data were matched to a specific latitude and longitude, and otherwise to the smallest areal unit (polygon) possible. The statistical model we employed for covariate modeling required point-referenced data, so data matched to polygons were resampled to generate pseudo-point data based on the underlying population distribution within the polygon. The methods for the resampling are consistent with those previously used in the geospatial modeling of many indicators, including adult HIV prevalence[7] and under-5 mortality[29]. Specifically, for each polygon-level observation, we randomly sampled 10,000 locations among grid cells in the given polygon with probability proportional to grid cell population. Grid cells were defined to be contained within the polygon if their centroid fell within the geographic boundary. We performed k-means clustering (with k set to 1 per 40 grid cells) on the sampled points to generate a reduced set of locations to be used in modeling based on the k-means cluster centroids. Weights were assigned to each pseudo-point proportional to the number of sampled points contained in each of the k-means clusters, i.e., the

number of sampled points divided by 10,000. Each pseudo-point generated by this process was assigned the HIV prevalence observed for the polygon as a whole, and a sample size equal to the sample size for the polygon as a whole multiplied by the weight derived for each point.

3.2.3 Covariate modeling

Each of these covariates was estimated using a simplified version of the modeling framework used for HIV prevalence as described in Additional File 1: Section 4.2, closely mirroring the framework previously used to model adult HIV prevalence[7]. Notable differences from the age- and sex-specific HIV prevalence model reported in this paper included:

- No covariates were included in the covariate geospatial models;
- No corrections for data derived from ANC sentinel surveillance were included (as no such data were used in these models);
- Covariate prevalence was modeled entirely at the disaggregated level (i.e., space- and time-specific). This was possible for covariate models because prevalence was specified at the age-aggregated level, and polygon data were resampled into pseudo-points;
- Because the covariate models did not include age or sex dimensions, only the spatiotemporal Gaussian process term was included;
- An unstructured error term (or 'nugget effect') for location s and year t was included;
- A fixed effect on time was included. This was particularly important for 'male circumcision' for capturing the growing emphasis on voluntary medical male circumcision as an intervention for HIV prevention[16]. For other covariates, this effect captured general regional time trends;
- Covariate models were fit in R-INLA[30]. Modeling in R-INLA was possible for the covariate models due to their more simplistic specifications relative to the age- and sex-specific HIV prevalence model.

Therefore, these models were specified as follows:

$$Y_j \sim Binomial(N_j, p_j)$$

$$logit(p_j) = \beta_0 + \boldsymbol{\beta}_1 \boldsymbol{t} + \gamma_{c[l]} + Z_j + \epsilon_j$$

$$\gamma_{c[l]} \sim Normal(0, \sigma 2_{country})$$

$$Z_j \sim GP(0, \Sigma_{space} \otimes \Sigma_{time})$$

$$\epsilon_j \sim Normal(0, \sigma^2_{nugget})$$

where:

- N_j is the number of individuals sampled and Y_j is the number of individuals who tested positive, or answered affirmatively among those sampled for the given covariate, for a given location and year (j);
- p_j is the underlying prevalence for the given covariate for a given location and year j;
- β_0 is an intercept;
- $\boldsymbol{\beta}_1\boldsymbol{t}$ is a fixed effect for a given year $\boldsymbol{t}$;
- $\gamma_{c[l]}$ is a country-level random effect for country c containing location l;
- Z_i is a spatially and temporally correlated random effect for a given location and year j;
- ϵ_i is an independently distributed random effect for a given location and year j.

All priors and hyper-priors were otherwise the same as those used for the same respective terms in the previously published adult HIV prevalence model[7]. Maps of each constructed covariate in 2000, 2005, 2010, and 2018 are displayed in Additional File 3: Figs. S1-8.

3.3 Administrative boundaries

For this analysis we used shape files from the Database of Global Administrative Areas (GADM)[31] to define country boundaries and first- and second-level administrative subdivisions. We manually updated known discrepancies.

3.4 Gridded population

The gridded population data used for this analysis were obtained from WorldPop[5]. Because WorldPop provides data at a 1 x 1-km spatial resolution at five-year intervals, we processed these data as described in Additional File 1: Section 3.1 to aggregate to a 5 x 5-km spatial resolution and interpolate to annual time periods. When we use population as a covariate, we use total population. In all other instances (as described in Additional File 1: Sections 2.3 and 4.4) we use age- and sex-specific population.

4 Statistical model

4.1 Covariate stacking

Stacked generalization/regression, or stacking, is an ensemble modeling method that combines multiple prediction methods to increase predictive validity relative to a single modeling approach. This ensemble modeling method relies on a variety of sub-models that are then combined by a secondary learner to produce a meta-model that fuses multiple algorithmic methods to capture nonlinear effects and

complex interactions[32]. Our implementation of stacking largely follows the approach described by Bhatt and colleagues[33] and which was previously implemented for modeling adult HIV prevalence[7].

Because the HIV-specific covariates were modeled at the age- and (largely) sex-aggregated level, we fit the stacker models at that same level, using HIV prevalence data aggregated across ages 15–49 years and both sexes. The age range 15–49 years was used in this case because of its predominant use in seroprevalence surveys compared to the 15–59 years range, allowing us to retain more data for use in stacking purposes. Polygon data were excluded from stacking models due to their incongruity with the configurations needed for the different sub-models. The ANC data were also excluded due to known sampling biases, which are described in the Additional File 1: Section 4.2.

We fit three sub-models – a generalized additive model, boosted regression trees, and lasso regression – to the HIV survey data with the five pre-existing and eight constructed covariates as well as calendar year included as explanatory variables. We selected these three sub-models based on ease of implementation through existing software packages, the fundamental differences in their approaches, and a proven track record of predictive accuracy[33]. Sub-models were fit in R using the mgcv[34], xgboost[35], glmnet[36], and caret[37] packages.

Each sub-model was fit using five-fold cross-validation to avoid overfitting, and hyper-parameter fitting was done to maximize predictive power. For each sub-model, we produced two sets of predictions: out-of-sample and in-sample. Out-of-sample predictions for each model were generated by compiling the predictions from the five holdouts from each cross-validation fold, and in-sample predictions were generated by re-fitting the sub-models using all available data. The out-of-sample sub-model predictions were used as explanatory covariates when fitting the geostatistical model described below, and the in-sample predictions were used when generating predictions from the geostatistical model in order to maximize data use. In both instances, the logit-transformation of the predictions was used to put these predictions on the same scale as the linear predictors in the geostatistical model. Maps of in-sample predictions from each stacker are presented in Additional File 3: Figs. S9-11.

4.2 Geostatistical model

4.2.1 Model description

We modeled HIV prevalence using a generalized linear mixed effects model discretized by space, time, age, and sex. To simultaneously model our point and polygon observations, and our age-specific and age-aggregated observations, we modeled prevalence at the observation level (i). However, prevalence was first specified at the space, time, age-, and sex-disaggregated level (j):

$$Y_i \sim \text{Binomial}(N_i, p_i)$$

$$\text{logit}(p_j) = \beta_0 + \boldsymbol{\beta}_1 \boldsymbol{X}_j + Z_{1,j} + Z_{2,j} + Z_{3,c[j]}$$

$$Z_{1,j} \sim \text{GP}(0, \Sigma_{space} \otimes \Sigma_{1,time})$$

$$Z_{2,j} \sim \text{GMRF}(0, \Sigma_{2,time} \otimes \Sigma_{2,age} \otimes \Sigma_{2,sex})$$

$$Z_{3,c[j]} \sim \text{GMRF}(0, \Sigma_{3,c})$$

where:

- N_i and Y_i are the number of individuals sampled and the number of individuals who are HIV$^+$ among those sampled, respectively, at the observation level (i);
- p_i is the underlying HIV prevalence at the observation level i;
- p_j is the underlying HIV prevalence at the fully disaggregated (i.e., location, year, age, and sex-specific; j) level;
- β_0 is an intercept;
- $\boldsymbol{X}_j$ is a vector of logit-transformed stacked covariates at the disaggregated level j, and $\boldsymbol{\beta}_1$ is the corresponding vector of regression coefficients;
- $Z_{1,j}$ random effects correlated across space and time;
- $Z_{2,j}$ is a random effect correlated across time, age, and sex;
- $Z_{3,c[j]}$ is a country-specific (c) random effect correlated across age.

Descriptively, we modeled the number of HIV-positive individuals (Y_i) among a sample (N_i) for a given observation i as a binomial variable. The model first specified logit-transformed prevalence at the disaggregated level (p_j) as a linear combination of a regional intercept (β_0), age- and sex-specific covariate effects ($\boldsymbol{\beta}_1 \boldsymbol{X}_j$), and random effects correlated across space, time, age, and sex ($Z_{1,j}, Z_{2,j}, Z_{3,c[j]}$). The intercept captures the overall mean level of HIV prevalence, while the covariate effects capture the spatial and temporal variation in HIV prevalence that can be described as a function of spatial and temporal variation in the included covariates. The random effects correlated across space, time, age, and sex capture additional variation by location (within and between countries), time, age, and sex that varies smoothly over these dimensions.

We then applied age-specific transformations related to fertility to p_j (described below), calculated as:

$$p_{transformed,j} = \frac{(p_j \cdot FRR_j)}{(p_j \cdot FRR_j) + 1 - p_j}$$

where:

- $p_{transformed,j}$ is the underlying HIV prevalence at the disaggregated level j, transformed to account for age-specific differences in fertility within observation-level data derived from antenatal care clinic sentinel surveillance. For all other survey data, $p_{transformed,j} = p_j$;
- And FRR_j is the fertility rate ratio between HIV$^+$ and HIV$^-$ females at the disaggregated level j, used to correct for age-specific differences within observation-level (i.e., in this case, age-aggregated) data derived from data derived from antenatal care clinic sentinel surveillance. For all other survey data, FRR_j = 1;

Finally, prevalence at the observation level (p_i) was then specified as:

$$p_i = \text{logit}^{-1}\left(\text{logit}\left(\sum\left(p_{transformed,j} \cdot w_j\right)\right) + \left(\beta_2 + U_{s[i]}\right) \cdot I_{ANC} + \epsilon_i\right)$$

$$U_{s[i]} \sim \text{Normal}\left(0, \sigma_{site}^2\right)$$

$$\epsilon_i \sim \text{Normal}(0, \sigma_i^2)$$

where:

- w_j is the weight applied to data at the disaggregated level. For point and age-specific data, w_j = 1;
- I_{ANC} is an indicator variable that is 1 for data derived from antenatal care clinic sentinel surveillance and 0 otherwise;
- β_2 is a fixed offset for observation-level data derived from antenatal care clinic sentinel surveillance;
- $U_{i[s]}$ is a site-level random effect for data derived from antenatal care clinic sentinel surveillance for observation i containing ANC site s;
- and (ϵ_i) is an observation-level error term.

Technically our polygon and age-aggregated data would follow a convolution of a mixture of binomial distributions. However, for computational efficiency we instead implement here a binomial approximation where for a given observation i:

$$Y_i \sim \text{Binomial}(N_i, p_i)$$

$$p_i = \sum_j w_j p(x_j) / \sum_j w_j$$

where we take w_j to be the population density proportion at pixel-age j (i.e., location and age x_j) for the polygon and/or age range for observation i, and $\sum_j w_j = 1$. We expected increased variance in our estimates given this modeling framework compared to a model with equal data coverage that used only point and age-specific data; however, given the limited availability of point and age-specific data, sensitivity analyses (see Additional File 1: Section 4.3 and Additional File 3: Figs. S13-15) demonstrate the larger benefit to our model in terms of reducing bias and error provided by the inclusion of aggregated data. We chose this method for including aggregated data rather than the polygon resampling method previously used to model adult HIV prevalence[7] among other indicators because polygon resampling is less robust[38], isn't able to account for variation in the spatial covariates or spatial field within polygon data sources, and uses an ad-hoc method for down-weighting the sample size of the resampled points. Also, the new method enabled us to disaggregate data not only over space but also by age, and allowed us to account for ANC-related bias at both age-aggregated and age-disaggregated levels.

HIV prevalence as measured by sentinel surveillance of antenatal care (ANC) clinics is known to be biased as a measure of HIV prevalence in the general adult population because it captures pregnant females who attend ANC only, as compared to all adult females[39, 40]. This bias may be either positive or negative: the fact that all pregnant females are sexually active tends to elevate their risk of having acquired HIV prevalence compared to the general female population (some of whom are not sexually active), while HIV-related sub-fertility tends to reduce the prevalence of HIV^+ females among the population of pregnant females[41, 42]. Additionally, HIV-related sub-fertility tends to vary across ages[43]; however, ANC data reported at the age-aggregated level does not account for these differences. Further, we do not expect the sampling bias within age- and spatially aggregated ANC observations to correspond with underlying populations, as we do for survey data. Nevertheless, ANC data have better temporal and spatial coverage in many countries than survey data alone (Figure 1). We therefore incorporated ANC data to capitalize on this additional data coverage, but also attempted to correct for the known biases in multiple ways.

First, to account for age-specific differences in the fertility rate ratio of HIV^+ and HIV^- females, we corrected prevalence estimates from ANC clinics at the disaggregated level according to age-specific fertility rate ratios, calculated according to age-specific and HIV-status-specific fertility estimates from

GBD 2019[6]. Fertility rate ratios were calculated at the national level, except for in Ethiopia, Kenya, Nigeria, and South Africa, where estimates were available at the first administrative level.

Second, because we expect sampling prevalence for ANC data disaggregated over space and age to vary according to age- and location-specific ANC clinic visitation rates, rather than according to the distribution of the underlying population, we calculated the w_j values for disaggregated ANC data to reflect this. Specifically, we used the number of births in a given year, location, and age as a proxy for ANC visitation rate, and weighted disaggregated ANC data accordingly. Births were calculated by multiplying the local population of females in the given year and age (based on local estimates from WorldPop[5]) by the national fertility rate for that year and age (based on national-level estimates from GBD 2019[6]).

Third, we accounted for ANC-related bias at the observation level. In instances where data in our model were derived from ANC sentinel surveillance (I_{ANC} = 1), our model allows for this bias via a fixed term (β_2) that captures the overall mean bias, and a site-specific random effect ($U_{i[l]}$) that captures local differences in the extent of this bias. This approach is conceptually like previously described approaches for spatial modeling using non-randomized (and therefore potentially biased) data and randomized survey data[44, 45]. Although the bias associated with ANC sentinel surveillance may also vary over time in addition to varying spatially, we felt there was insufficient data to estimate both spatial and temporal variation in this bias, and so the bias associated with ANC sentinel surveillance was assumed to be time-invariant over the period of this analysis.

The spatially and temporally correlated random effect (Z_{1j}) was modeled as a Gaussian process with mean 0 and a covariance matrix given by the Kronecker product of a spatial Matérn covariance function (Σ_{space}) and a temporal first-order autoregressive (AR1) covariance function (Σ_{time}). The Matérn covariance function is given by:

$$\Sigma_{space} = \sigma^2 \frac{2^{1-v}}{\Gamma(v)} \cdot (\kappa D)^v \cdot K_v(\kappa D)$$

In this analysis v (the smoothness parameter) was fixed at 1. A penalized complexity (PC) prior was used for the Matérn covariance function and specified via two hyper-parameters: the spatial range, ρ_s (where $\rho_s = \sqrt{8v}/\kappa$ and is equal to the distance at which correlation is approximately 0.1; the subscript s for space is used as to not confuse with the other correlation parameters, below), and marginal standard deviation, σ. PC priors shrink towards a more simplistic base model – in this case, one where the

marginal variance is 0 and the spatial range is infinite – and are specified via setting the tail probabilities on each hyper-parameter[46, 47]. We followed the guidance provided by Fugulstad et al., who recommend selecting priors that satisfy $P(\sigma > \sigma_0) = 0.05$ and $P(\rho_s > \rho_{s_0}) = 0.05$, where σ_0 is between 2.5 to 40 times the expected true marginal standard deviation and ρ_{s_0} is between 1⁄10 to 1⁄2.5 of the expected true range[48]. Specifically, we set:

$$\sigma_0 = 5;\ P(\sigma > \sigma_0) = 0.05$$

$$\rho_{s_0} = 0.01\ radians;\ P(\rho_s > \rho_{s_0}) = 0.05$$

Separate σ parameters were specified for each Z_j term included in the model; each was assigned the same prior as above. Individual σ parameters were also included for the observation-level error term (ϵ_i) and the ANC random effect ($U_{s[i]}$), with respective priors set as:

$$\sigma = 3;\ P(\sigma > \sigma_0) = 0.05$$

Additionally, for all Z_j terms included in the model, the AR1 covariance function is associated with different parameters accounting for correlations in time, age, and sex—ρ_t, ρ_z, and ρ_x, respectively. Unique ρ parameters were identified in each of their respective appearances in the model. For example, because an AR1 temporal covariance function was incorporated into the covariance matrices for $Z_{1,j}$, and $Z_{2,j}$, we fit two separate ρ_t parameters ($\rho_{1,2,t}$). We nevertheless used the same following hyper-prior for all ρ_t, ρ_z, and ρ_x parameters, which corresponds to a prior mean of 0.76 with a 95% range of -0.17 to 0.97:

$$\log\left(\frac{1+\rho}{1-\rho}\right) \sim \text{Normal}(2, 1.2^2)$$

Finally, priors for fixed effects were set as:

$$\beta_0 \sim \text{Normal}(0, 3^2)$$

$$\beta_1 \sim \text{Normal}(0, 3^2)$$

$$\beta_2 \sim \text{Normal}(0, 3^2)$$

4.2.2 Model fitting and prediction

This model was fit in Template Model Builder (TMB)[49], package in R version 3.6.1. We used the stochastic partial differential equations (SPDE) approach[50] to approximate the continuous spatiotemporal Gaussian random field ($Z_{1,j}$). We constructed a finite elements mesh for the SPDE approximation to the Gaussian process regression using a simplified polygon boundary (Additional File 3:

Fig. S41). We used a spatial mesh that was constructed on the S^2 domain which allowed distance to be calculated along a sphere instead of using Euclidean distance between latitude and longitude coordinates. We set the inner mesh triangle minimum edge length to 35 km, the maximum triangle length to 500 km, with the mesh extending 500 km past the region's boundary. We used maximum a posteriori (MAP) inference, using a maximum likelihood estimation with an augmented optimization objective (log-likelihood function) which incorporated prior distributions for all model parameters. Estimated model parameters are listed in Additional File 2: Table S6.

Due to computational constraints and to allow for regional differences in the relationship between covariates and HIV prevalence as well as the strength of auto-correlation across space, time, age, and sex in HIV prevalence, separate models were fit for four regions (Additional File 3: Fig. S12). Specifically, we used the regional classifications for SSA from the Global Burden of Disease (GBD) study[51] which group countries by location and epidemiological profile. We made small modifications to this classification, grouping Sudan as part of the Eastern SSA region rather than the North Africa and the Middle East region. We also dropped Cape Verde, Comoros, São Tomé and Príncipe, and Mauritania from these modeling regions due to data missingness.

After fitting each model, we generated 1,000 draws of all model parameters from the approximated joint posterior distribution using a multivariate-normal approximation. For each draw s of the model parameters, we constructed a draw of

$$p_j^{(s)} = \operatorname{logit}^{-1}\left(\beta_0^{(s)} + \beta_1^{(s)} X_j + Z_{1,j}^{(s)} + Z_{2,j}^{(s)} + Z_{3,c[j]}^{(s)}\right)$$

I_{ANC} is set to 0 for the purposes of generating estimates, so draws of β_2 and U_i are not incorporated when generating draws of p_j. Additional processing of the output from the multivariate-normal approximation is required for the spatial-temporal random effect ($Z_{1,j}^{(s)}$) prior to constructing $p_j^{(s)}$ according to the equation above. Specifically, for $Z_{1,j}^{(s)}$, draws are generated initially only at vertices of the finite element mesh, so we project from this mesh to each pixel-year combination desired for prediction, i.e., the centroid of each grid cell on a 5 × 5-km grid as well as all years from 2000 to 2018. At the end of this process, we have 1,000 draws of $p_j^{(s)}$ for each grid cell and year combination.

4.3 Model validation

4.3.1 Validation strategy

We used five-fold out-of-sample cross-validation in order to assess the performance of the modeling framework described above with respect to predicting HIV prevalence. We first split all location- and age-specific data into five groups using spatial and temporal stratification[52]. Temporal folds were created by stratifying across years such that each fold contains approximately 1/5 of the data for each year. Spatial folds were constructed we used a modified quadtree algorithm to spatially aggregate data points. This algorithm recursively partitions two-dimensional space, alternating between horizontal and vertical splits on the weighted data sample size medians. The depth of recursive partitioning is constrained by the target sample size within a partition and the minimum number of clusters or pseudo-clusters allowed within each spatial partition. The minimum sample size was set according to data availability in each region—the minimum sample size was set at 425 for Central SSA and Southern SSA, and 500 for Eastern SSA and Western SSA. These partitions were then allocated to one of five folds for cross validation. This resulted in five groups that are approximately equal in terms of the total effective sample size. We then fit the model described above five times, excluding each of the five holdout data groups in turn. All ANC data were included in all models and were not used to assess model performance given the known biases in these data. Due to difficulties in comparing age-aggregated and polygon data to age- and location-specific results, polygon and age-aggregated survey data were excluded from use in assessing model performance and were therefore used in all models.

After fitting the model five times, the data withheld from each model were matched with predictions from that model, and then these data-prediction pairs were compiled across all five models, resulting in a complete dataset of out-of-sample predictions corresponding to all location- and age-specific data included in the analysis. HIV prevalence estimates based on single survey clusters are generally quite noisy due to very small sample sizes and are consequently insufficient as a 'gold standard' for evaluating the model predictions[29]. To address this issue, we aggregated both the observed data and the corresponding age- and sex-specific out-of-sample predictions within countries and within first- and second-level administrative subdivisions, by calculating a weighted mean of each using the effective sample sizes as the weights. Then, across all data-estimate pairs, we calculated two summary measures: the mean error (ME, a measure of bias) and the root-mean square error (RMSE, a measure of total variance).

In addition, for each data-estimate pair, we constructed 95% prediction intervals from the 2.5th and 97.5th percentiles of 1,000 draws from a binomial distribution corresponding to each of the 1,000

posterior draws of HIV prevalence with p equal to HIV prevalence in a given posterior draw and N equal to the effective sample size for the data point. We then calculated coverage as the percentage of data-estimate pairs where the data point was contained within this 95% prediction interval. Finally, to complement the out-of-sample predictive validity metrics, we calculated in-sample predictive validity metrics using the same process but matching each data point to predictions from a model fit using all data.

4.3.2 Sensitivity analyses

We used this validation strategy to assess model performance of the final model compared models of adult prevalence, as well as a number of alternatives related to data inclusion and model specification[53].

4.3.2.1 Adult prevalence sensitivity

We assessed the performance of our age- and sex-specific model compared to an adult-level HIV prevalence model, that is, one for combined sexes and ages 15–49 years. In these comparisons, we validated the results of the age and sex model not only at the age- and sex-disaggregated level, but also for estimates re-aggregated to the adult level (see Additional File 1: Section 4.3.3). The adult prevalence model we tested mirrored the age- and sex-specific model as closely as possible; all survey microdata and reports for ages 15–49 years were included, as well as all ANC data. All parameters from the age- and sex-specific model were retained in the adult prevalence model, except those that pertained to age and sex correlations (i.e., $Z_{2,j}$ and $Z_{3,[c]j}$). To replace the country-level variation provided in the age- and sex-specific model by the country-specific age correlation term ($Z_{3,c[j]}$), we instead included a country-level random effect, $\gamma_{[c]j}$. Logit-transformed disaggregated prevalence $\text{logit}(p_j)$ was therefore specified as:

$$\text{logit}(p_j) = \beta_0 + \beta_1 X_j + Z_{1,j} + \gamma_{[c]j}$$

$$Z_{1,j} \sim \text{GP}(0, \Sigma_{space} \otimes \Sigma_{1,time})$$

Observation-level adult prevalence (p_i) was calculated using the same equation from age- and sex-specific prevalence estimation, differing only in that the transformation related to age-specific fertility-rate ratios (FRR) was not applied.

To assess our decision to employ novel methods for including polygon data in our model rather than the previously utilized polygon resampling technique[7, 54], we also compare our results to those of an adult prevalence model built using polygon resampling. We elected to test polygon resampling in an

age-aggregated model due to the age- and sex-specific model's heavy reliance on age-aggregated data, which is processed in effectively the same manner as the polygon data. We therefore avoid this conflict by testing resampling in the adult prevalence model. In total this resulted in the comparison of four models and corresponding sets of results:

1. The final age- and sex-specific model, with age- and sex-specific results;
2. The final age- and sex-specific model, results re-aggregated to the adult level;
3. Results for an adult prevalence model, employing the novel polygon processing system as in the final model;
4. Results for an adult prevalence model, employing the previously published polygon resampling system.

Comparisons of adult prevalence when modeled versus re-aggregated can be seen in Additional File 3: Fig. S16. The results of this sensitivity analyses can be found in Additional File 3: Fig. S13. The re-aggregated adult estimates were outperformed by the modeled adult estimates in some respects, but not in others. For example, our mean error calculations were much closer to zero (indicating less bias) for modeled adult prevalence compared to re-aggregated estimates. This may ultimately be a product of our process for re-aggregating age- and sex-specific estimates—these calculations are heavily influenced by local population structure. We also calculated consistent overestimations for 95% coverage for the age- and sex-specific model, indicating some overestimation of our uncertainty intervals compared to modeled adult prevalence. Meanwhile RMSE tended to be substantially lower for the re-aggregated estimates (indicating lower variance). The in- vs. out-of-sample results also tended to be more similar within the re-aggregated estimates compared to other models, although this also varied by region. Some necessary differences in data and model configuration likely contributed to these differences. Further investigation of the influences on these differences will be an important future direction in this line of research.

4.3.2.2 Data sensitivity

To assess the contribution of our different data sources, we tested additional models with the following subsets of the data:

1. Survey data only (no ANC data);
2. Point and age-specific data only (no polygon or age-aggregated data).

The results of this sensitivity analyses can be found in Additional File 3: Fig. S14. We found the performance of these models using smaller data subsets to be very region-specific. For example, when ANC data were excluded, mean error in Eastern and Southern SSA tended to be closer to zero (i.e., less biased) compared to when these data were included. When all polygon and age-aggregated data were excluded, Eastern SSA was still less biased, but in this case out-of-sample Southern SSA performed worse than when all data were included. Central and Western SSA, on the other hand, performed dramatically worse in terms of mean error when ANC as well as all polygon and age-aggregated data were excluded. Survey data were severely limited in Central SSA in particular, so it is not surprising that estimates in this region were highly dependent on ANC data. These results were similar for our other validation metrics. Given that Eastern and Southern SSA have relatively better spatial and temporal survey data coverage (Figure 1), it is expected that these regions would be more robust to the loss of ANC and other aggregated data. It is clear that while these unconventional data sources provide tremendous insight in the absence of better survey data coverage, more work is needed to reduce bias associated with their inclusion.

4.3.2.3 Statistical configuration sensitivity

To assess our final chosen statistical configuration, we assess the utility of each term included in the model by testing models excluding individual parameters. This resulted in six additional models:

1. No interaction between the space and time correlation terms;
2. No interaction between the time, age, and sex correlation terms;
3. No interactions whatsoever between the space, time, age, and sex correlation terms;
4. No country-specific age correlation term;
5. No observation-level error term;
6. No stackers.

In cases where interactions between terms were removed, the individual terms were retained if not included elsewhere in the model. For example, for the model where the interaction between space and time correlations was removed, and additional “space-only” correlation term was included, but because the time correlation was still accounted for in the time-age-sex interaction, no additional time correlation was included. The results of this sensitivity analyses can be found in Additional File 3: Fig. S15. We note that in a number of respects, our final chosen model did not out-perform those excluding some of our chosen parameters (Additional File 3: Fig. S15). For example, the out-of-sample RSME values for our final model in many cases were higher than those for other tested models. We believe

this may be partially driven by the fact that our validation analyses are conducted exclusively for our point and age-specific data. Given the heavy reliance of this model on polygon and age-aggregated data, we believe that these sensitivity analyses provide an incomplete assessment of our model performance. In in-sample testing, our final model did outperform other models with regards to RMSE, though we acknowledge this does not speak to our ability to predict to sparsely sampled location-years. We also found that in inclusion of some terms, such as the country-specific age effect, $Z_{3,c[j]}$ and the observation-level error term helped to reduce bias and smooth trends at the national level, which may not be reflected in these validation metrics. With additional data and computing power, it is probable that this model would benefit from additional and more complex interactions. However, given the resources currently available to us, we are confident that our final model represents the best possible option at this time.

4.3.3 Comparisons to adult prevalence estimates

As this age- and sex-specific HIV prevalence model serves as a follow-up to a previously described analysis of adult (ages 15–49 years) HIV prevalence[7], it was important that we compare the estimates from this model to one mirroring its predecessor. To make this comparison effectively, it was necessary that we re-aggregate our age- and sex-specific results to the 'adult' level. We therefore calculated HIV prevalence for adults ages 15–49 years by summing our final age- and sex-specific PLHIV estimates across males and females age groups 15–49 years, for each grid cell and year, and dividing those by cell- and year-specific population estimates summed across the same age groups. Both PLHIV and population estimates were derived during the post-estimation process, described below in Additional File 1: Section 4.4. In select grid cells where the population was estimated to be zero, prevalence was weighted by the second administrative-level population age and sex structure. For a description of the calculation of second administrative-level estimates, see Additional File 1: Section 4.4. For sensitivity analyses, re-aggregated estimates were compared to location-specific survey microdata collapsed across all adults ages 15–49 years, the same data used to validate modeled adult prevalence. For a comparison of these results re-aggregated across sexes and age groups to HIV prevalence estimates modeled across adults, see Additional File 3: Figs. S13 and S16.

4.4 Post-estimation

4.4.1 Aggregation to first- and second-level administrative subdivisions

In addition to estimates of HIV prevalence on a grid, we also constructed estimates of HIV prevalence for first- and second-level administrative subdivisions. These estimates were derived by calculating population-weighted averages of HIV prevalence for each grid cell or fractional grid cell within a given

first- or second-level administrative subdivision for a given age, sex, and year. Grid cell fractions were assigned at the second-level administrative subdivision shape to determine what fraction of the area of each grid cell fell within each administrative unit. Since all second-level subdivisions nest within first-level subdivisions, which in turn nest within countries, this strategy assigned the cell fractions to an administrative area at each level of the administrative hierarchy. We assumed that population density within each cell was uniform, and for cells that were split across multiple subdivisions, allocated the WorldPop population estimate in proportion to area. This process was carried out separately for each modeling region, so cells that cross international borders that are also regional borders were allocated in their entirety to the country that contained the centroid of the grid cell. This was carried out for each of the 1,000 posterior draws at the grid cell level, generating 1,000 posterior draws for each administrative subdivision. Final estimates and uncertainty intervals for each subdivision at each level of the administrative hierarchy were derived from the mean, 2.5th percentile, and 97.5th percentile of these draws, respectively.

4.4.2 Calibration to Global Burden of Disease 2019

To take advantage of the more epidemiologically structured modeling approach and additional national-level data used by GBD 2019, we performed post-hoc calibration of our estimates to the GBD estimates[43]. Using the assignment of cells and cell fractions to the administrative hierarchy described above, we first scaled the grid cell-level WorldPop estimates[5] to match the corresponding GBD population estimates[6] for each country, year, age, and sex. To do so, for each country, year, age, and sex, we defined a population raking factor as the ratio of the GBD population estimate to the sum of the WorldPop population estimates for all cells and fractional cells within the country, and then multiplied the WorldPop population estimates for all cells and fractional cells within the country by this raking factor.

We then similarly adjusted our HIV prevalence estimates. Specifically, for each country, year, age, and sex, we defined a prevalence ‘raking factor’ as the ratio of the GBD prevalence estimate to the population-weighted mean of estimates for all cells and fractional cells within the country, and then multiplied each HIV prevalence draw for all cells and fractional cells within the country by this raking factor. At this point, the prevalence estimates for cells that had been fractionally allocated to multiple countries were recombined by calculating a weighted average, with weights determined by the relative area of each fraction. Final calibrated estimates for each grid cell were calculated as the mean of the scaled draws, and 95% uncertainty intervals were calculated as the 2.5th and 97.5th percentiles of the

scaled draws. The impact of this calibration procedure is depicted in Additional File 3: Figs. S17 and S18, which compares the pre-calibration estimates to the post-calibration estimates.

4.4.3 Calculating people living with HIV (PLHIV)

We estimated the number of people living with HIV (PLHIV) in each grid cell, year, age and sex by combining estimated population and HIV prevalence after calibration to GBD 2019 estimates as described above. Specifically, for each cell and fractional cell, we multiplied the estimated population by each of the 1,000 prevalence draws to generate 1,000 draws of PLHIV. Fractional cells were then recombined by summing PLHIV for each draw within each cell. Final point estimates and uncertainty intervals for PLHIV were calculated as the mean, 2.5th percentile, and 97.5th percentile of these draws, respectively.

5 References

1. Global Health Data Exchange | GHDx. Available at: http://ghdx.healthdata.org/. (Accessed: 16th June 2020)

2. UNAIDS. National HIV estimates file. 2019. https://www.unaids.org/en/dataanalysis/datatools/spectrum-epp.

3. The United States Census Bureau. HIV/AIDS Database. 2019. https://www.census.gov/programs-surveys/international-programs/about/hiv.html. Accessed 16 Jun 2020.

4. Wiegand H. Kish, L.: Survey Sampling. John Wiley & Sons, Inc., New York, London 1965, IX + 643 S., 31 Abb., 56 Tab., Preis 83 s. Biom Z. 1968;10:88–9.

5. WorldPop. WorldPop Dataset. 2020. http://www.worldpop.org.uk/data/get_data/.

6. GBD 2019 Demographics Collaborators. Global age-sex-specific fertility, mortality, healthy life expectancy (HALE), and population estimates in 204 countries and territories, 1950–2019: a comprehensive demographic analysis for the Global Burden of Disease Study 2019. Lancet. 2020;396:1160–203.

7. Dwyer-Lindgren L, Cork MA, Sligar A, Steuben KM, Wilson KF, Provost NR, et al. Mapping HIV prevalence in sub-Saharan Africa between 2000 and 2017. Nature. 2019;570:189–93.

8. Tatem AJ, Hemelaar J, Gray RR, Salemi M. Spatial accessibility and the spread of HIV-1 subtypes and recombinants. AIDS. 2012;26:2351–60.

9. Gray RR, Tatem AJ, Lamers S, Hou W, Laeyendecker O, Serwadda D, et al. Spatial phylodynamics of HIV-1 epidemic emergence in east Africa. AIDS Lond Engl. 2009;23:F9–17.

10. Abu-Raddad LJ, Patnaik P, Kublin JG. Dual Infection with HIV and Malaria Fuels the Spread of Both Diseases in Sub-Saharan Africa. Science. 2006;314:1603–6.

11. Cuadros DF, Branscum AJ, Crowley PH. HIV–malaria co-infection: effects of malaria on the prevalence of HIV in East sub-Saharan Africa. Int J Epidemiol. 2011;40:931–9.

12. Auvert B, Buvé A, Ferry B, Caraël M, Morison L, Lagarde E, et al. Ecological and individual level analysis of risk factors for HIV infection in four urban populations in sub-Saharan Africa with different levels of HIV infection. AIDS. 2001;15:S15.

13. Bailey RC, Moses S, Parker CB, Agot K, Maclean I, Krieger JN, et al. Male circumcision for HIV prevention in young men in Kisumu, Kenya: a randomised controlled trial. Lancet Lond Engl. 2007;369:643–56.

14. World Health Organization. WHO | UNAIDS statement on South African trial findings regarding male circumcision and HIV. 2005. https://www.who.int/mediacentre/news/releases/2005/pr32/en/.

15. Sgaier SK, Reed JB, Thomas A, Njeuhmeli E. Achieving the HIV prevention impact of voluntary medical male circumcision: lessons and challenges for managing programs. PLOS Med. 2014;11:e1001641.

16. Cork MA, Wilson KF, Perkins S, Collison ML, Deshpande A, Eaton JW, et al. Mapping male circumcision for HIV prevention efforts in sub-Saharan Africa. BMC Med. 2020;18:189.

17. Freeman EE, Weiss HA, Glynn JR, Cross PL, Whitworth JA, Hayes RJ. Herpes simplex virus 2 infection increases HIV acquisition in men and women: systematic review and meta-analysis of longitudinal studies. AIDS. 2006;20:73–83.

18. Chen L, Jha P, Stirling B, Sgaier SK, Daid T, Kaul R, et al. Sexual Risk Factors for HIV Infection in Early and Advanced HIV Epidemics in Sub-Saharan Africa: Systematic Overview of 68 Epidemiological Studies. PLoS ONE. 2007;2:e1001.

19. Glynn JR, Caraël M, Auvert B, Kahindo M, Chege J, Musonda R, et al. Why do young women have a much higher prevalence of HIV than young men? A study in Kisumu, Kenya and Ndola, Zambia. AIDS. 2001;15:S51.

20. Johnson K, Way A. Risk Factors for HIV Infection in a National Adult Population: Evidence From the 2003 Kenya Demographic and Health Survey. JAIDS J Acquir Immune Defic Syndr. 2006;42:627–36.

21. Pettifor AE, van der Straten A, Dunbar MS, Shiboski SC, Padian NS. Early age of first sex: a risk factor for HIV infection among women in Zimbabwe. AIDS Lond Engl. 2004;18:1435–42.

22. Coffee MP, Garnett GP, Mlilo M, Voeten HACM, Chandiwana S, Gregson S. Patterns of Movement and Risk of HIV Infection in Rural Zimbabwe. J Infect Dis. 2005;191 Supplement_1:S159–67.

23. Bwayo J, Plummer F, Omari M, Mutere A, Moses S, Ndinya-Achola J, et al. Human Immunodeficiency Virus Infection in Long-Distance Truck Drivers in East Africa. Arch Intern Med. 1994;154:1391–6.

24. Davis KR, Weller SC. The effectiveness of condoms in reducing heterosexual transmission of HIV. Fam Plann Perspect. 1999;31:272–9.

25. Duncan ME, Peutherer JF, Simmonds P, Young H, Tibaux G, Pelzer A, et al. First coitus before menarche and risk of sexually transmitted disease. The Lancet. 1990;335:338–40.

26. Stöckl H, Kalra N, Jacobi J, Watts C. Is Early Sexual Debut a Risk Factor for HIV Infection Among Women in Sub-Saharan Africa? A Systematic Review. Am J Reprod Immunol. 2013;69:27–40.

27. Carswell JW, Lloyd G, Howells J. Prevalence of HIV-1 in east African lorry drivers. AIDS. 1989;3:759–62.

28. Brown NR, Sinclair RC. Estimating number of lifetime sexual partners: Men and women do it differently. J Sex Res. 1999;36:292–7.

29. Golding N, Burstein R, Longbottom J, Browne AJ, Fullman N, Osgood-Zimmerman A, et al. Mapping under-5 and neonatal mortality in Africa, 2000–15: a baseline analysis for the Sustainable Development Goals. The Lancet. 2017;390:2171–82.

30. Rue H, Martino S, Chopin N. Approximate Bayesian inference for latent Gaussian models by using integrated nested Laplace approximations. J R Stat Soc Ser B Stat Methodol. 2009;71:319–92.

31. GADM database of global administrative areas. 2018. https://gadm.org/ (accessed May 6, 2018). Accessed 6 May 2018.

32. Breiman L. Stacked regressions. Mach Learn. 1996;24:49–64.

33. Bhatt S, Cameron E, Flaxman SR, Weiss DJ, Smith DL, Gething PW. Improved prediction accuracy for disease risk mapping using Gaussian process stacked generalization. J R Soc Interface. 2017;14.

34. Wood SN. Generalized additive models: an introduction with R, second edition. CRC Press; 2017.

35. Chen T, He T. xgboost: eXtreme gradient boosting. :4.

36. Friedman JH, Hastie T, Tibshirani R. Regularization paths for generalized linear models via coordinate descent. J Stat Softw. 2010;33:1–22.

37. Kuhn M, Wing J, Weston S, Williams A, Keefer C, Engelhardt A, et al. caret: classification and regression training. 2020.

38. Marquez N, Wakefield J. Harmonizing child mortality data at disparate geographic Levels. ArXiv200200089 Stat. 2020.

39. Gouws E, Mishra V, Fowler TB. Comparison of adult HIV prevalence from national population-based surveys and antenatal clinic surveillance in countries with generalised epidemics: implications for calibrating surveillance data. Sex Transm Infect. 2008;84 Suppl 1:i17–23.

40. Marsh K, Mahy M, Salomon JA, Hogan DR. Assessing and adjusting for differences between HIV prevalence estimates derived from national population-based surveys and antenatal care surveillance, with applications for Spectrum 2013. AIDS Lond Engl. 2014;28:S497–505.

41. Kongnyuy EJ, Wiysonge CS. Association between fertility and HIV status: what implications for HIV estimates? BMC Public Health. 2008;8:309.

35. Zaba, B. & Gregson, S. Measuring the impact of HIV on fertility in Africa. AIDS12 Suppl 1, S41-50 (1998).

43. GBD 2019 Diseases and Injuries Collaborators. Global burden of 369 diseases and injuries in 204 countries and territories, 1990–2019: a systematic analysis for the Global Burden of Disease Study 2019. Lancet. 2020;396:1204–22.

44. Giorgi E, Sesay SSS, Terlouw DJ, Diggle PJ. Combining data from multiple spatially referenced prevalence surveys using generalized linear geostatistical models. J R Stat Soc Ser A Stat Soc. 2015;178:445–64.

45. Diggle PJ, Giorgi E. Model-Based Geostatistics for Prevalence Mapping in Low-Resource Settings. J Am Stat Assoc. 2016;111:1096–120.

46. Franco-Villoria M, Ventrucci M, Rue H. A unified view on Bayesian varying coefficient models. ArXiv180602084 Stat. 2019.

47. Simpson D, Rue H, Riebler A, Martins TG, Sørbye SH. Penalising Model Component Complexity: A Principled, Practical Approach to Constructing Priors. Stat Sci. 2017;32:1–28.

48. Fuglstad G-A, Simpson D, Lindgren F, Rue H. Constructing Priors that Penalize the Complexity of Gaussian Random Fields. J Am Stat Assoc. 2019;114:445–52.

49. Kristensen K, Nielsen A, Berg CW, Skaug H, Bell BM. TMB: Automatic Differentiation and Laplace Approximation. J Stat Softw. 2016;70:1–21.

50. Lindgren F, Rue H, Lindström J. An explicit link between Gaussian fields and Gaussian Markov random fields: the stochastic partial differential equation approach. J R Stat Soc Ser B Stat Methodol. 2011;73:423–98.

51. Murray CJ, Ezzati M, Flaxman AD, Lim S, Lozano R, Michaud C, et al. GBD 2010: design, definitions, and metrics. The Lancet. 2012;380:2063–6.

52. Roberts DR, Bahn V, Ciuti S, Boyce MS, Elith J, Guillera-Arroita G, et al. Cross-validation strategies for data with temporal, spatial, hierarchical, or phylogenetic structure. Ecography. 2017;40:913–29.

53. Waller LA. Estimate suggests many infant deaths in sub-Saharan Africa attributable to air pollution. Nature. 2018;559:188–9.

54. Burstein R, Henry NJ, Collison ML, Marczak LB, Sligar A, Watson S, et al. Mapping 123 million neonatal, infant and child deaths between 2000 and 2017. Nature. 2019;574:353–8.
