# Supplementary material for: Mapping age- and sex-specific HIV prevalence in adults in sub-Saharan Africa, 2000–2018
Source: BMC Med. 2022 Dec 19;20:488. doi: 10.1186/s12916-022-02639-z (PMC9760541; doi:10.1186/s12916-022-02639-z)
Supplement: Supplementary file 2 — Additional file 2: Supplemental tables.Table S1. HIV seroprevalence survey data. Table S2. ANC sentinel surveillance data. Table S3. HIV and covariates surveys excluded from this analysis. Table S4. Sources for pre-existing covariates. Table S5. HIV covariate survey data. Table S6. Fitted model parameters. [file 12916_2022_2639_MOESM2_ESM.pdf]

Additional File 2: Supplemental Tables

Mapping age- and sex-specific HIV prevalence in adults in sub-Saharan Africa, 2000–2018

Contents

Table S1: HIV seroprevalence survey data (2000–2018) 2
Table S2: ANC sentinel surveillance data (2000–2018) 16
Table S3: HIV and covariates surveys excluded from this analysis 29
Table S4: Sources for pre-existing covariates 39
Table S5: HIV covariate survey data (2000–2018) 40
Table S6: Fitted model parameters 140

Table S1: HIV seroprevalence survey data (2000–2018)

Country	Years	Name	Type	Ages	Sexes	Age Detail	Geographic Detail	Citation	NID*
Angola	2015-16	Angola Demographic and Health Survey 2015-2016	Survey microdata	15-54	Males & females	Disaggregated	GPS	ICF International, Ministry of Health (Angola), National Institute of Statistics (Angola), United Nations Children's Fund (UNICEF). Angola Demographic and Health Survey 2015-2016. Fairfax, United States of America: ICF International, 2017.	218555
Benin	2006	Benin Demographic and Health Survey 2006	Survey report	15-49	Males & females	Aggregated	Admin 1	Macro International, Inc, National Institute of Statistics and Economic Analysis (INSAE) (Benin), National Program Against AIDS (PNLS) (Benin). Benin Demographic and Health Survey 2006. Fairfax, United States of America: ICF International.	18959
Benin	2012	Benin Demographic and Health Survey 2011-2012	Survey report	15-49	Males & females	Aggregated	Admin 1	ICF International, National Institute of Statistics and Economic Analysis (INSAE) (Benin), National Program Against AIDS (PNLS) (Benin). Benin Demographic and Health Survey 2011-2012. Fairfax, United States of America: ICF International, 2014.	79839
Botswana	2004	Botswana AIDS Impact Survey 2004	Survey microdata	15-59	Males & females	Disaggregated	GPS	Central Statistics Office (Botswana). Botswana AIDS Impact Survey 2004. Gaborone, Botswana: Statistics Botswana.	22114
Botswana	2008	Botswana AIDS Impact Survey 2008	Survey microdata	15-59	Males & females	Disaggregated	GPS	Central Statistics Office (Botswana), National AIDS Coordinating Agency (Botswana). Botswana AIDS Impact Survey 2008. Gaborone, Botswana: Statistics Botswana, 2015.	22116
Botswana	2013	Botswana AIDS Impact Survey 2013	Survey microdata	15-59	Males & females	Disaggregated	GPS	Ministry of Health (Botswana), National AIDS Coordinating Agency (Botswana), Statistics Botswana. Botswana AIDS Impact Survey 2013. Gaborone, Botswana: Statistics Botswana, 2015.	134753
Burkina Faso	2003	Burkina Faso Demographic and Health Survey 2003	Survey microdata	15-59	Males & females	Disaggregated	GPS	Macro International, Inc, National Institute of Statistics and Demography (Burkina Faso). Burkina Faso Demographic and Health Survey 2003. Fairfax, United States of America: ICF International.	19088
Burkina Faso	2010	Burkina Faso Demographic and	Survey microdata	15-59	Males & females	Disaggregated	GPS	ICF Macro, Ministry of Health (Burkina Faso), National Institute of Statistics and Demography (Burkina Faso).	19133

Country	Years	Name	Type	Ages	Sexes	Age Detail	Geographic Detail	Citation	NID*
		Health Survey 2010-2011						Burkina Faso Demographic and Health Survey 2010-2011. Fairfax, United States of America: ICF International.	
Burundi	2010-11	Burundi Demographic and Health Survey 2010-2011	Survey microdata	15-59	Males & females	Disaggregated	GPS	Burundi Institute of Statistics and Economic Studies, ICF International, Ministry of Public Health and the Fight Against AIDS (Burundi). Burundi Demographic and Health Survey 2010-2011. Fairfax, United States of America: ICF International, 2012.	30431
Burundi	2016-17	Burundi Demographic and Health Survey 2016-2017	Survey microdata	15-59	Males & females	Disaggregated	GPS	Burundi Institute of Statistics and Economic Studies, ICF International, Ministry of Public Health and the Fight Against AIDS (Burundi). Burundi Demographic and Health Survey 2016-2017. Fairfax, United States of America: ICF International, 2018.	286766
Burundi	2007	Burundi Combined HIV/AIDS/STI Behavioral Surveillance and HIV/AIDS Seroprevalence Survey 2007	Survey report	15-49	Males & females	Aggregated	Admin 1	National Council for the Fight Against AIDS (CNLS) (Burundi). Burundi Combined HIV/AIDS/STI Behavioral Surveillance and HIV/AIDS Seroprevalence Survey 2007.	335814
Cameroon	2004	Cameroon Demographic and Health Survey 2004	Survey microdata	15-59	Males & females	Disaggregated	GPS	Macro International, Inc, National Institute of Statistics (Cameroon). Cameroon Demographic and Health Survey 2004. Fairfax, United States of America: ICF International.	19211
Cameroon	2011	Cameroon Demographic and Health Survey 2011	Survey microdata	15-59	Males & females	Disaggregated	GPS	ICF International, Ministry of Economy, Planning and Regional Development (Cameroon), Ministry of Public Health (Cameroon), National Institute of Statistics (Cameroon), Pasteur Center of Cameroon. Cameroon Demographic and Health Survey 2011. Fairfax, United States of America: ICF International.	19274
Cameroon	2018-19	Cameroon Demographic and Health Survey 2018-2019	Survey microdata	15-59	Males & females	Disaggregated	GPS	ICF International, Ministry of Public Health (Cameroon), National Institute of Statistics (Cameroon). Cameroon Demographic and Health Survey 2018-2019. Fairfax, United States of America: ICF International.	413167

Country	Years	Name	Type	Ages	Sexes	Age Detail	Geographic Detail	Citation	NID*
Central African Republic	2006	Central African Republic Multiple Indicator Cluster Survey 2006	Survey report	15-49	Males & females	Aggregated	Admin 1	United Nations Children's Fund (UNICEF). Central African Republic Multiple Indicator Cluster Survey 2006. New York, United States: United Nations Children's Fund (UNICEF).	2223
Central African Republic	2010	Central African Republic Multiple Indicator Cluster Survey 2010-2011	Survey report	15-49	Males & females	Aggregated	Admin 1	Central African Institute of Statistics, Economic and Social Studies (ICASEES) (Central African Republic), ICF International. Central African Republic Multiple Indicator Cluster Survey 2010-2011. Fairfax, United States of America: ICF International, 2013.	82832
Chad	2014-15	Chad Demographic and Health Survey 2014-2015	Survey microdata	15-59	Males & females	Disaggregated	GPS	ICF International, National Institute for Statistics, Economic and Demographic Studies (INSEED) (Chad). Chad Demographic and Health Survey 2014-2015. Fairfax, United States of America: ICF International, 2016.	157025
Congo	2009	Congo AIDS Indicator Survey 2009	Survey microdata	15-49	Males & females	Disaggregated	Admin 1	ICF Macro, National Center for Statistics and Economic Studies (Congo, Rep.). Congo AIDS Indicator Survey 2009. Fairfax, United States of America: ICF International.	3133
Congo	2003	HIV and AIDS Surveillance Database, Version July 2017	Survey report	15-49	Males & females	Aggregated	Admin 1	United States Census Bureau. HIV and AIDS Surveillance Database, Version July 2017. Washington, D.C., United States: United States Census Bureau, 2017.	313360
Côte d'Ivoire	2011-12	Côte d'Ivoire Demographic and Health Survey 2011-2012	Survey microdata	15-59	Males & females	Disaggregated	GPS	ICF International, Ministry of the Fight Against AIDS (Côte d'Ivoire), National Institute of Statistics (Côte d'Ivoire). Côte d'Ivoire Demographic and Health Survey 2011-2012. Fairfax, United States of America: ICF International.	18533
Côte d'Ivoire	2005	Côte d'Ivoire AIDS Indicator Survey 2005	Survey microdata	15-49	Males & females	Disaggregated	Admin >1	CDC Retro-CI, Ministry of the Fight Against AIDS (Côte d'Ivoire), National Institute of Statistics (Côte d'Ivoire), ORC Macro. Côte d'Ivoire AIDS Indicator Survey 2005. Fairfax, United States of America: ICF International.	56148
Democratic Republic of the Congo	2007	Democratic Republic of the Congo Demographic and Health Survey 2007	Survey microdata	15-59	Males & females	Disaggregated	GPS	Macro International, Inc, Ministry of Planning (Congo, DR). Democratic Republic of the Congo Demographic and Health Survey 2007. Fairfax, United States of America: ICF International.	19381

Country	Years	Name	Type	Ages	Sexes	Age Detail	Geographic Detail	Citation	NID*
Democratic Republic of the Congo	2013-14	Democratic Republic of the Congo Demographic and Health Survey 2013-2014	Survey microdata	15-59	Males & females	Disaggregated	GPS	ICF International, Ministry of Planning and Monitoring Implementation of the Revolution of Modernity (Congo, DR), Ministry of Public Health (Congo, DR), National Institute of Statistics (Congo, DR). Democratic Republic of the Congo Demographic and Health Survey 2013-2014. Fairfax, United States of America: ICF International, 2014.	76878
Equatorial Guinea	2011	Equatorial Guinea Demographic and Health Survey 2011	Survey report	15-49	Males & females	Aggregated	Admin 1	ICF International, Ministry of Health and Social Welfare (Equatorial Guinea), Ministry of Planning, Economic Development and Public Investment (Equatorial Guinea). Equatorial Guinea Demographic and Health Survey 2011. Fairfax, United States of America: ICF International, 2012.	76884
Eritrea	2010	Eritrea Population and Health Survey 2010	Survey report	15-49	Males & females	Aggregated	Admin 1	Kenya Medical Research Institute (KEMRI), National Statistics Office (Eritrea), The Fafo Research Foundation. Eritrea Population and Health Survey 2010.	249999
Eswatini	2006-07	Swaziland Demographic and Health Survey 2006-2007	Survey microdata	15-59	Males & females	Disaggregated	GPS	Central Statistical Office (Swaziland), Macro International, Inc. Swaziland Demographic and Health Survey 2006-2007. Fairfax, United States of America: ICF International.	20829
Eswatini	2017	Swaziland HIV Incidence Measurement Survey 2 2016-2017	Survey microdata	15-59	Males & females	Disaggregated	Admin 1	Government of the Kingdom of Eswatini. Swaziland HIV Incidence Measurement Survey 2 (SHIMS2) 2016-2017. Final Report. Mbabane: Government of the Kingdom of Eswatini; April 2019.	415531
Ethiopia	2005	Ethiopia Demographic and Health Survey 2005	Survey microdata	15-59	Males & females	Disaggregated	GPS	Macro International, Inc, Population and Housing Census Commissions Office (PHCCO). Ethiopia Demographic and Health Survey 2005. Fairfax, United States of America: ICF International.	19557
Ethiopia	2011-12	Ethiopia Demographic and Health Survey 2010-2011	Survey microdata	15-59	Males & females	Disaggregated	GPS	Central Statistical Agency (Ethiopia), ICF Macro, Ministry of Health (Ethiopia). Ethiopia Demographic and Health Survey 2010-2011. Fairfax, United States of America: ICF International.	21301
Ethiopia	2016	Ethiopia Demographic and	Survey microdata	15-59	Males & females	Disaggregated	GPS	Central Statistical Agency (Ethiopia), ICF International. Ethiopia Demographic and Health Survey 2016. Fairfax, United States of America: ICF International, 2017.	218568

Country	Years	Name	Type	Ages	Sexes	Age Detail	Geographic Detail	Citation	NID*
		Health Survey 2016							
Gabon	2012	Gabon Demographic and Health Survey 2012	Survey microdata	15-59	Males & females	Disaggregated	GPS	General Directorate of Statistics (Gabon), ICF International, Ministry of Economy, Employment and Sustainable Development (Gabon), Ministry of Health (Gabon). Gabon Demographic and Health Survey 2012. Fairfax, United States of America: ICF International, 2013.	76706
Gambia	2013	Gambia Demographic and Health Survey 2013	Survey microdata	15-59	Males & females	Disaggregated	Admin 2	Gambia Bureau of Statistics (GBOS), ICF International, Ministry of Health and Social Welfare (The Gambia). Gambia Demographic and Health Survey 2013. Fairfax, United States of America: ICF International, 2015.	77384
Ghana	2003	Ghana Demographic and Health Survey 2003	Survey microdata	15-59	Males & females	Disaggregated	GPS	Ghana Statistical Service, Macro International, Inc. Ghana Demographic and Health Survey 2003. Fairfax, United States of America: ICF International.	19627
Ghana	2014	Ghana Demographic and Health Survey 2014	Survey microdata	15-59	Males & females	Disaggregated	GPS	Ghana Health Service, Ghana Statistical Service, ICF International. Ghana Demographic and Health Survey 2014. Fairfax, United States of America: ICF International, 2016.	157027
Guinea	2005	Guinea Demographic and Health Survey 2005	Survey microdata	15-59	Males & females	Disaggregated	GPS	Macro International, Inc, National Statistics Directorate (Guinea). Guinea Demographic and Health Survey 2005. Fairfax, United States of America: ICF International.	19683
Guinea	2012	Guinea Demographic and Health Survey 2012	Survey microdata	15-59	Males & females	Disaggregated	GPS	ICF Macro, Ministry of Health and Public Hygiene (Guinea), National Institute of Statistics (Guinea). Guinea Demographic and Health Survey 2012. Fairfax, United States of America: ICF International.	69761
Guinea	2018	Guinea Demographic and Health Survey 2018	Survey microdata	15-59	Males & females	Disaggregated	GPS	Ministry of Health (Guinea), Ministry of Planning and Economic Development (Guinea), National Institute of Statistics (Guinea). Guinea Demographic and Health Survey 2018. Fairfax, United States of America: ICF International, 2019.	396957
Kenya	2003	Kenya Demographic and	Survey microdata	15-54	Males & females	Disaggregated	GPS	Centers for Disease Control and Prevention (CDC), Central Bureau of Statistics (Kenya), Macro International, Inc,	20145

Country	Years	Name	Type	Ages	Sexes	Age Detail	Geographic Detail	Citation	NID*
		Health Survey 2003						Ministry of Health (Kenya), National Council for Population and Development (Kenya). Kenya Demographic and Health Survey 2003. Fairfax, United States of America: ICF International.	
Kenya	2008-09	Kenya Demographic and Health Survey 2008-2009	Survey microdata	15-54	Males & females	Disaggregated	GPS	ICF Macro, Kenya Medical Research Institute (KEMRI), Kenya National Bureau of Statistics, Ministry of Public Health and Sanitation (Kenya), National AIDS and STI Control Programme (NASCOP) (Kenya), National Aids Control Council (NACC), National Coordinating Agency for Population and Development (Kenya). Kenya Demographic and Health Survey 2008-2009. Fairfax, United States of America: ICF International.	21365
Kenya	2007	Kenya AIDS Indicator Survey 2007	Survey microdata	15-59	Males & females	Disaggregated	GPS	Centers for Disease Control and Prevention (CDC), Kenya Medical Research Institute (KEMRI), Kenya National Bureau of Statistics, Ministry of Public Health and Sanitation (Kenya), National AIDS Control Council (Kenya), National AIDS and STI Control Programme (NASCOP) (Kenya), National Coordinating Agency for Population and Development (Kenya), National Public Health Laboratory Services, Ministry of Public Health and Sanitation (Kenya), United States Agency for International Development (USAID). Kenya AIDS Indicator Survey 2007. Nairobi, Kenya: Kenya National Bureau of Statistics.	133219
Kenya	2013	Kenya AIDS Indicator Survey 2012-2013	Survey microdata	15-59	Males & females	Disaggregated	GPS	Kenya National Bureau of Statistics, Ministry of Devolution and Planning (Kenya), Ministry of Health (Kenya), National AIDS and STI Control Programme (NASCOP) (Kenya). Kenya AIDS Indicator Survey 2012-2013. Nairobi, Kenya: Kenya National Bureau of Statistics.	133304
Lesotho	2004-05	Lesotho Demographic and Health Survey 2004-2005	Survey microdata	15-59	Males & females	Disaggregated	GPS	Bureau of Statistics (Lesotho), Macro International, Inc, Ministry of Health and Social Welfare (Lesotho). Lesotho Demographic and Health Survey 2004-2005. Fairfax, United States of America: ICF International.	20167
Lesotho	2009-10	Lesotho Demographic and	Survey microdata	15-59	Males & females	Disaggregated	GPS	ICF Macro, Ministry of Health and Social Welfare (Lesotho). Lesotho Demographic and Health Survey 2009-2010. Fairfax, United States of America: ICF International.	21382

Country	Years	Name	Type	Ages	Sexes	Age Detail	Geographic Detail	Citation	NID*
		Health Survey 2009-2010							
Lesotho	2014	Lesotho Demographic and Health Survey 2014	Survey microdata	15-59	Males & females	Disaggregated	GPS	ICF International, Ministry of Health and Social Welfare (Lesotho). Lesotho Demographic and Health Survey 2014. Fairfax, United States of America: ICF International.	157058
Lesotho	2017	Lesotho Population-based HIV Impact Assessment 2016-2017	Survey report	15-49	Males & females	Aggregated	Admin 1	Ministry of Health, Lesotho, Centers for Disease Control and Prevention (CDC), and ICAP at Columbia University. Lesotho Population-based HIV Impact Assessment (LePHIA) 2016-2017: Final Report. Maseru, Lesotho, Atlanta, Georgia, and New York, New York, USA: Ministry of Health, CDC, and ICAP, September 2019.	327582
Liberia	2006-07	Liberia Demographic and Health Survey 2006-2007	Survey microdata	15-49	Males & females	Disaggregated	GPS	Liberia Institute for Statistics and Geo-information Services (LISGIS), Macro International, Inc. Liberia Demographic and Health Survey 2006-2007. Fairfax, United States of America: ICF International.	20191
Liberia	2013	Liberia Demographic and Health Survey 2013	Survey microdata	15-49	Males & females	Disaggregated	GPS	ICF International, Liberia Institute for Statistics and Geo-information Services (LISGIS), National AIDS and STI Control Program (NACP), Ministry of Health and Social Welfare (Liberia). Liberia Demographic and Health Survey 2013. Fairfax, United States of America: ICF International.	77385
Malawi	2004-05	Malawi Demographic and Health Survey 2004-2005	Survey microdata	15-54	Males & females	Disaggregated	GPS	Macro International, Inc, National Statistical Office of Malawi. Malawi Demographic and Health Survey 2004-2005. Fairfax, United States of America: ICF International.	20263
Malawi	2010	Malawi Demographic and Health Survey 2010	Survey microdata	15-54	Males & females	Disaggregated	GPS	ICF Macro, National Statistical Office of Malawi. Malawi Demographic and Health Survey 2010. Fairfax, United States of America: ICF International.	21393
Malawi	2015-16	Malawi Demographic and Health Survey 2015-2016	Survey microdata	15-54	Males & females	Disaggregated	GPS	Emory University and Centers for Disease Control & Prevention Collaboration, ICF International, Ministry of Health (Malawi), National Statistical Office of Malawi. Malawi Demographic and Health Survey 2015-2016. Fairfax, United States of America: ICF International, 2017.	218581

Country	Years	Name	Type	Ages	Sexes	Age Detail	Geographic Detail	Citation	NID*
Malawi	2015-16	Malawi Population-based HIV Impact Assessment 2015-2016	Survey microdata	15-59	Males & females	Disaggregated	Admin >1	Ministry of Health, Malawi, Centers for Disease Control and Prevention (CDC), and ICAP at Columbia University. Malawi Population-based HIV Impact Assessment (MPHIA) 2015-16: First Report. Lilongwe, Malawi, Atlanta, Georgia and New York, New York, USA: Ministry of Health, CDC and ICAP. December 2016.	287629
Mali	2006	Mali Demographic and Health Survey 2006	Survey microdata	15-59	Males & females	Disaggregated	GPS	Macro International, Inc, Ministry of Health (Mali), National Directorate of Statistics and Informatics (DNSI) (Mali). Mali Demographic and Health Survey 2006. Fairfax, United States of America: ICF International.	20274
Mali	2001	Mali Demographic and Health Survey 2001	Survey microdata	15-59	Males & females	Disaggregated	Admin 1, GPS	Macro International, Inc, National Directorate of Statistics and Informatics (DNSI) (Mali), Planning and Statistics Unit, Ministry of Health (Mali). Mali Demographic and Health Survey 2001. Fairfax, United States of America: ICF International.	20315
Mali	2012-13	Mali Demographic and Health Survey 2012-2013	Survey microdata	15-59	Males & females	Disaggregated	GPS	ICF International, INFO-STAT (Mali), Ministry of Health (Mali), National Institute of Statistics (INSTAT) (Mali), Planning and Statistics Unit, Ministry of Health (Mali). Mali Demographic and Health Survey 2012-2013. Fairfax, United States of America: ICF International, 2014.	77388
Mozambique	2009	Mozambique AIDS Indicator Survey 2009	Survey microdata	15-59	Males & females	Disaggregated	GPS	ICF Macro, Ministry of Health (Mozambique), National Institute of Statistics (INE) (Mozambique). Mozambique AIDS Indicator Survey 2009. Fairfax, United States of America: ICF International, 2010.	8906
Mozambique	2015	Mozambique AIDS Indicator Survey 2015	Survey microdata	15-59	Males & females	Disaggregated	GPS	Centers for Disease Control and Prevention (CDC), ICF International, Ministry of Health (Mozambique), National Institute of Health (Mozambique), National Institute of Statistics (INE) (Mozambique). Mozambique AIDS Indicator Survey 2015. Fairfax, United States of America: ICF International, 2018.	157060
Namibia	2013	Namibia Demographic and Health Survey 2013	Survey microdata	15-59	Males & females	Disaggregated	GPS	ICF International, Ministry of Health and Social Services (Namibia), Namibia Institute of Pathology, Namibia Statistics Agency. Namibia Demographic and Health Survey 2013. Fairfax, United States of America: ICF International.	150382

Country	Years	Name	Type	Ages	Sexes	Age Detail	Geographic Detail	Citation	NID*
Namibia	2017	Namibia Population-based HIV Impact Assessment 2017	Survey report	15-49	Males & females	Aggregated	Admin 1	Ministry of Health and Social Services (MoHSS), Namibia. Namibia Population-based HIV Impact Assessment (NAMPHIA) 2017: Final Report. Windhoek: MoHSS, Namibia; November, 2019.	359089
Namibia	2006-09	Namibia - Khomas Windhoek Okambilimbili HIV Endline Survey 2009	Survey microdata	15-59	Males & females	Disaggregated	GPS	Aulagnier M, Janssens W, De Beer I, van Rooy G, Gaeb E, Hesp C, van der Gaag J, Rinke de Wit TF. Namibia - Khomas Windhoek Okambilimbili HIV Endline Survey 2009.	425256
Niger	2006	Niger Demographic and Health Survey 2006	Survey microdata	15-59	Males & females	Disaggregated	Admin 1	Department of Statistics and National Accounts (Niger), Macro International, Inc. Niger Demographic and Health Survey 2006. Fairfax, United States of America: ICF International.	20499
Niger	2012	Niger Demographic and Health Survey 2012	Survey microdata	15-59	Males & females	Disaggregated	Admin 1	ICF International, Ministry of Public Health (Niger), National Institute of Statistics (Niger). Niger Demographic and Health Survey 2012. Fairfax, United States of America: ICF International.	74393
Nigeria	2018	Nigeria HIV/AIDS Indicator and Impact Survey 2018	Survey report	15-59	Males & females	Disaggregated	Admin 1	Federal Ministry of Health (Nigeria), Government of Nigeria, National Agency for the Control of AIDS (NACA) (Nigeria), University of Maryland. Nigeria HIV/AIDS Indicator and Impact Survey 2018.	399235
Rwanda	2005	Rwanda Demographic and Health Survey 2005	Survey microdata	15-59	Males & females	Disaggregated	GPS	Macro International, Inc, National Institute of Statistics of Rwanda. Rwanda Demographic and Health Survey 2005. Fairfax, United States of America: ICF International.	20740
Rwanda	2010-11	Rwanda Demographic and Health Survey 2010-2011	Survey microdata	15-59	Males & females	Disaggregated	GPS	ICF Macro, Ministry of Health (Rwanda), National Institute of Statistics of Rwanda. Rwanda Demographic and Health Survey 2010-2011. Fairfax, United States of America: ICF International.	56040
Rwanda	2014-15	Rwanda Demographic and Health Survey 2014-2015	Survey microdata	15-59	Males & females	Disaggregated	GPS	ICF International, Ministry of Health (Rwanda), National Institute of Statistics of Rwanda. Rwanda Demographic and Health Survey 2014-2015. Fairfax, United States of America: ICF International, 2016.	157063

Country	Years	Name	Type	Ages	Sexes	Age Detail	Geographic Detail	Citation	NID*
Rwanda	2013	Rwanda AIDS Indicator and HIV Incidence Survey 2013	Survey report	15-59	Males & females	Aggregated	Admin 1	Ministry of Health (Rwanda), Rwanda Biomedical Center (RBC). Rwanda AIDS Indicator and HIV Incidence Survey 2013.	343466
Senegal	2005	Senegal Demographic and Health Survey 2005	Survey microdata	15-59	Males & females	Disaggregated	GPS	Ministry of Health and Prevention (Senegal), Research Center for Human Development (Senegal). Senegal Demographic and Health Survey 2005. Fairfax, United States of America: ICF International.	26855
Senegal	2010-11	Senegal Demographic and Health Survey 2010-2011	Survey microdata	15-59	Males & females	Disaggregated	GPS	Center for Research in Human Development (CRDH), Cheikh Anta Diop University, Hospital Aristide Le Dantec, ICF Macro, National Agency of Statistics and Demography (Senegal). Senegal Demographic and Health Survey 2010-2011. Fairfax, United States of America: ICF International.	56063
Senegal	2017	Senegal Continuous Demographic and Health Survey 2017	Survey microdata	15-59	Males & females	Disaggregated	Admin 1	ICF International, Ministry of Health and Social Action (Senegal), National Agency of Statistics and Demography (Senegal), Unit for the Fight Against Malnutrition (Senegal). Senegal Continuous Demographic and Health Survey 2017. Fairfax, United States of America: ICF International, 2018.	353526
Sierra Leone	2008	Sierra Leone Demographic and Health Survey 2008	Survey microdata	15-59	Males & females	Disaggregated	GPS	Macro International, Inc, Statistics Sierra Leone. Sierra Leone Demographic and Health Survey 2008. Fairfax, United States of America: ICF International.	21258
Sierra Leone	2013	Sierra Leone Demographic and Health Survey 2013	Survey microdata	15-59	Males & females	Disaggregated	GPS	ICF International, Ministry of Health and Sanitation (Sierra Leone), Statistics Sierra Leone. Sierra Leone Demographic and Health Survey 2013. Fairfax, United States of America: ICF International, 2014.	131467
South Africa	2003-16	South Africa - ACDIS Health and Demographic Surveillance System	Survey microdata	15-59	Males & females	Disaggregated	Admin <1	Africa Centre for Population Studies and Reproductive Health, INDEPTH, South African Medical Research Council, University of Kwazulu-Natal. South Africa - ACDIS Health and Demographic Surveillance System.	11780
South Africa	2002	South Africa HIV/AIDS Behavioral Risks,	Survey microdata	15-59	Males & females	Disaggregated	Admin 3	Center for AIDS Development, Research and Evaluation (CADRE) (South Africa), Department of Social Development (South Africa), Family Health International, Geospace	12102

Country	Years	Name	Type	Ages	Sexes	Age Detail	Geographic Detail	Citation	NID*
		Sero-Status, and Mass Media Impact Survey 2002						International (South Africa), Human Sciences Research Council, Joint United Nations Program on HIV/AIDS (UNAIDS), Medical University of Southern Africa (MEDUNSA), National Agency for AIDS Research (ANRS) (France), National Health Laboratory Service (NHLS) (South Africa), South African Medical Research Council, University of Natal, Wits Health Consortium. South Africa HIV/AIDS Behavioral Risks, Sero-Status, and Mass Media Impact Survey 2002. Pretoria, South Africa: Human Sciences Research Council, 2011.	
South Africa	2016	South Africa Demographic and Health Survey 2016	Survey microdata	15-59	Males & females	Disaggregated	GPS	Department of Health (South Africa), ICF International, South African Medical Research Council, Statistics South Africa. South Africa Demographic and Health Survey 2016. Fairfax, United States of America: ICF International, 2019.	157064
South Africa	2008-09	South Africa National HIV Prevalence, Incidence, Behavior and Communication Survey 2008-2009	Survey microdata	15-59	Males & females	Disaggregated	Admin 5, GPS	Center for AIDS Development, Research and Evaluation (CADRE) (South Africa), Geospace International (South Africa), Global Clinical and Viral Laboratory (South Africa), Human Sciences Research Council, Maphume Research Services, National Institute for Communicable Diseases (South Africa), South African Medical Research Council. South Africa National HIV Prevalence, Incidence, Behavior and Communication Survey 2008-2009. Pretoria, South Africa: Human Sciences Research Council, 2014.	228102
South Africa	2005	South Africa National HIV Prevalence, Incidence, Behavior and Communication Survey 2004-2005	Survey microdata	15-59	Males & females	Disaggregated	Admin 3	Center for AIDS Development, Research and Evaluation (CADRE) (South Africa), Centers for Disease Control and Prevention (CDC), Global Clinical and Viral Laboratory (South Africa), Human Sciences Research Council, Maphume Research Services, National Institute for Communicable Diseases (South Africa). South Africa National HIV Prevalence, Incidence, Behavior and Communication Survey 2004-2005. Pretoria, South Africa: Human Sciences Research Council, 2011.	313074
South Africa	2011-12	South Africa National HIV Prevalence,	Survey microdata	15-59	Males & females	Disaggregated	Admin 5, GPS	Centers for Disease Control and Prevention (CDC), Global Clinical and Viral Laboratory (South Africa), Human Sciences Research Council, National Institute for	313076

Country	Years	Name	Type	Ages	Sexes	Age Detail	Geographic Detail	Citation	NID*
		Incidence, and Behavior Survey 2011-2012						Communicable Diseases (South Africa), South African Medical Research Council, University of Cape Town. South Africa National HIV Prevalence, Incidence, and Behavior Survey 2011-2012. Pretoria, South Africa: Human Sciences Research Council, 2016.	
Tanzania	2003-04	Tanzania AIDS Indicator Survey 2003-2004	Survey microdata	15-49	Males & females	Disaggregated	GPS	National Bureau of Statistics (Tanzania), ORC Macro, Tanzania Commission for AIDS (TACAIDS). Tanzania AIDS Indicator Survey 2003-2004. Fairfax, United States of America: ICF International.	12630
Tanzania	2007-08	Tanzania HIV/AIDS and Malaria Indicator Survey 2007-2008	Survey microdata	15-49	Males & females	Disaggregated	GPS	Macro International, Inc, National Bureau of Statistics (Tanzania), Office of the Chief Government Statistician (OCGS) (Zanzibar), Tanzania Commission for AIDS (TACAIDS), Zanzibar AIDS Commission (ZAC). Tanzania HIV/AIDS and Malaria Indicator Survey 2007-2008. Fairfax, United States of America: ICF International.	12644
Tanzania	2011-12	Tanzania AIDS Indicator Survey 2011-2012	Survey microdata	15-49	Males & females	Disaggregated	GPS	ICF International, National Bureau of Statistics (Tanzania), Office of the Chief Government Statistician (OCGS) (Zanzibar), Tanzania Commission for AIDS (TACAIDS), Zanzibar AIDS Commission (ZAC). Tanzania AIDS Indicator Survey 2011-2012. Fairfax, United States of America: ICF International, 2013.	77395
Tanzania	2017	Tanzania HIV Impact Survey 2016-2017	Survey microdata	15-59	Males & females	Disaggregated	Admin 1	Tanzania Commission for AIDS (TACAIDS), Zanzibar AIDS Commission (ZAC). Tanzania HIV Impact Survey(THIS) 2016-2017: Final Report. Dar es Salaam, Tanzania. December 2018.	327591
Togo	2013-14	Togo Demographic and Health Survey 2013-2014	Survey microdata	15-59	Males & females	Disaggregated	GPS	Directorate General of Statistics and National Accounts (Togo), ICF International, Ministry of Health (Togo), Ministry of Planning, Development and Zoning (Togo). Togo Demographic and Health Survey 2013-2014. Fairfax, United States of America: ICF International, 2015.	77515
Uganda	2004-05	Uganda AIDS Indicator Survey 2004-2005	Survey microdata	15-59	Males & females	Disaggregated	Admin 1	Division of Reproductive Health, Centers for Disease Control and Prevention (CDC), Ministry of Health (Uganda). Uganda AIDS Indicator Survey 2004-2005.	13084

Country	Years	Name	Type	Ages	Sexes	Age Detail	Geographic Detail	Citation	NID*
Uganda	2011	Uganda AIDS Indicator Survey 2011	Survey microdata	15-59	Males & females	Disaggregated	GPS	Centers for Disease Control and Prevention (CDC), ICF Macro, Ministry of Health (Uganda), Uganda Bureau of Statistics, Uganda Virus Research Institute. Uganda AIDS Indicator Survey 2011. Fairfax, United States of America: ICF International.	55973
Uganda	2016	Uganda Population-based HIV Impact Assessment 2016-2017	Survey report	15-49	Males & females	Aggregated	Admin 1	Ministry of Health, Uganda. Uganda Population-based HIV Impact Assessment (UPHIA) 2016-2017: Final Report. Kampala: Ministry of Health; July, 2019.	327593
Zambia	2002	Zambia Demographic and Health Survey 2001-2002	Survey microdata	15-59	Males & females	Disaggregated	Admin 1	Central Board of Health (Zambia), Central Statistical Office (Zambia), Macro International, Inc. Zambia Demographic and Health Survey 2001-2002. Fairfax, United States of America: ICF International.	21102
Zambia	2007	Zambia Demographic and Health Survey 2007	Survey microdata	15-59	Males & females	Disaggregated	GPS	Central Statistical Office (Zambia), Macro International, Inc. Zambia Demographic and Health Survey 2007. Fairfax, United States of America: ICF International.	21117
Zambia	2013-14	Zambia Demographic and Health Survey 2013-2014	Survey microdata	15-59	Males & females	Disaggregated	GPS	Central Statistical Office (Zambia), ICF International, Ministry of Health (Zambia), Tropical Diseases Research Centre, University Teaching Hospital (Zambia), University of Zambia. Zambia Demographic and Health Survey 2013-2014. Fairfax, United States of America: ICF International.	77516
Zambia	2016	Zambia Population-based HIV Impact Assessment 2016	Survey microdata	15-59	Males & females	Disaggregated	Admin 2	Ministry of Health, Zambia, Centers for Disease Control and Prevention (CDC), and ICAP at Columbia University. Zambia Population-based HIV Impact Assessment (ZAMPHIA) 2015-16: First Report. Lusaka, Zambia, Atlanta, Georgia and New York, New York, USA: Ministry of Health, CDC and ICAP. December 2016.	287630
Zambia	2018	Zambia Demographic and Health Survey 2018-2019	Survey microdata	15-59	Males & females	Disaggregated	Admin 1	Central Statistical Office (Zambia), ICF International, Ministry of Health (Zambia), University Teaching Hospital (Zambia), University of Zambia. Zambia Demographic and Health Survey 2018-2019. Fairfax, United States of America: ICF International, 2020.	411301

Country	Years	Name	Type	Ages	Sexes	Age Detail	Geographic Detail	Citation	NID*
Zimbabwe	2005-06	Zimbabwe Demographic and Health Survey 2005-2006	Survey microdata	15-54	Males & females	Disaggregated	GPS	Central Statistical Office (Zimbabwe), Macro International, Inc. Zimbabwe Demographic and Health Survey 2005-2006. Fairfax, United States of America: ICF International.	21163
Zimbabwe	2010-11	Zimbabwe Demographic and Health Survey 2010-2011	Survey microdata	15-54	Males & females	Disaggregated	GPS	ICF Macro, Zimbabwe National Statistics Agency. Zimbabwe Demographic and Health Survey 2010-2011. Calverton, United States of America: ICF Macro, 2012.	55992
Zimbabwe	2015	Zimbabwe Demographic and Health Survey 2015	Survey microdata	15-54	Males & females	Disaggregated	GPS	ICF International, National Microbiology Reference Laboratory, Harare Central Hospital (NMRL) (Zimbabwe), Zimbabwe National Statistics Agency. Zimbabwe Demographic and Health Survey 2015. Fairfax, United States of America: ICF International, 2016.	157066
Zimbabwe	2016	Zimbabwe Population-based HIV Impact Assessment 2015-2016	Survey report	15-49	Males & females	Aggregated	Admin 1	Ministry of Health and Child Care (MOHCC), Zimbabwe, Centers for Disease Control and Prevention (CDC), and ICAP at Columbia University. Zimbabwe Population-based HIV Impact Assessment (ZIMPHIA) 2015-16: First Report. Harare, Zimbabwe, Atlanta, Georgia and New York, New York, USA: Ministry of Health, CDC and ICAP. December 2016.	287631
Zimbabwe	2005	Zimbabwe - Chimanimani Behavioral Risks and HIV Serostatus Survey 2005	Survey report	15-24	Males & females	Aggregated	Admin 2	Biomedical Research and Training Institute (Zimbabwe), Human Sciences Research Council, National Institute of Health Research, Ministry of Health and Child Welfare (NIHR) (Zimbabwe), Zimbabwe Central Statistics Office. Zimbabwe - Chimanimani Behavioral Risks and HIV Serostatus Survey 2005.	333662

*NID = Data source unique identifier in the Global Health Data Exchange (http://ghdx.healthdata.org/). Additional information about each data sources is available via the GHDx, including information about the data provider and links to where the data can be accessed or requested (where available). NIDs can be entered in the search bar to retrieve the record for a particular source.

Table S2: ANC sentinel surveillance data (2000–2018)

Country	Years	Source	Geographic Detail	Citation	NID*
Angola	2001-02	HIV Prevalence Among Women Attending Antenatal Care by Province 1985-2002	GPS	Angola Prevalence of HIV, Hepatitis B, and Syphilis, and HIV Prevalence Among Women Attending Antenatal Care, Personal Communication with K. Stanecki and D. Serrano. [Unpublished].	347096
Angola	2002	Presentation of HIV Surveillance Information and CAP Survey	GPS	Serrano D, National AIDS Control Program, Ministry of Health (PNLS) (Angola). Presentation of HIV Surveillance Information and CAP Survey.	347109
Angola	2004-05, 2007, 2009, 2011, 2013	UNAIDS Spectrum - National HIV Estimates 2019	GPS	Joint United Nations Program on HIV/AIDS (UNAIDS). UNAIDS Spectrum - National HIV Estimates 2019. Geneva, Switzerland: Joint United Nations Program on HIV/AIDS (UNAIDS), 2018.	415978
Benin	2000-17	UNAIDS Spectrum - National HIV Estimates 2019	GPS	Joint United Nations Program on HIV/AIDS (UNAIDS). UNAIDS Spectrum - National HIV Estimates 2019. Geneva, Switzerland: Joint United Nations Program on HIV/AIDS (UNAIDS), 2018.	415978
Botswana	2000-03, 2005-07, 2009, 2011, 2015	UNAIDS Spectrum - National HIV Estimates 2019	Admin 2, GPS	Joint United Nations Program on HIV/AIDS (UNAIDS). UNAIDS Spectrum - National HIV Estimates 2019. Geneva, Switzerland: Joint United Nations Program on HIV/AIDS (UNAIDS), 2018.	415978
Botswana	2001-03	Botswana HIV Sentinel Survey 2003	Admin 2	Botswana AIDS/STD Unit, Botswana Harvard AIDS Institute Partnership (BHP), Centers for Disease Control and Prevention (CDC), Government of Botswana, National AIDS Coordinating Agency (Botswana), World Health Organization (WHO). Botswana HIV Sentinel Survey 2003.	347264
Botswana	2000	Botswana HIV Sentinel Survey 2000	Admin 2	Botswana AIDS/STD Unit, Centers for Disease Control and Prevention (CDC), Government of Botswana, National AIDS Coordinating Agency (Botswana), World Health Organization (WHO). Botswana HIV Sentinel Survey 2000.	347350
Botswana	2005	Botswana HIV Sentinel Survey 2005	Admin 2	Botswana Harvard AIDS Institute Partnership (BHP), Centers for Disease Control and Prevention (CDC), Department of AIDS Prevention and Care, Ministry of Health (Botswana), Joint United Nations Program on HIV/AIDS (UNAIDS), Ministry of Local Government (Botswana), National AIDS	347307

Country	Years	Source	Geographic Detail	Citation	NID*
				Coordinating Agency (Botswana), National Health Laboratory (NHL) (Botswana), World Health Organization (WHO). Botswana HIV Sentinel Survey 2005.	
Botswana	2007	Botswana HIV ANC Sentinel Survey 2007	Admin 2	African Comprehensive HIV/AIDS Partnerships (ACHAP), Botswana Harvard AIDS Institute Partnership (BHP), Centers for Disease Control and Prevention (CDC), Central Statistics Office (Botswana), Department of AIDS Prevention and Care, Ministry of Health (Botswana), Department of Local Government Service Management (DLGSM) (Botswana), Government of Botswana, Health Statistics Unit, Ministry of Health (Botswana), Joint United Nations Program on HIV/AIDS (UNAIDS), Ministry of Local Government (Botswana), National AIDS Coordinating Agency (Botswana), National Health Laboratory (NHL) (Botswana), World Health Organization (WHO). Botswana HIV ANC Sentinel Survey 2007.	347145
Burkina Faso	2000-17	UNAIDS Spectrum - National HIV Estimates 2019	GPS	Joint United Nations Program on HIV/AIDS (UNAIDS). UNAIDS Spectrum - National HIV Estimates 2019. Geneva, Switzerland: Joint United Nations Program on HIV/AIDS (UNAIDS), 2018.	415978
Burundi	2000-10, 2015, 2017	UNAIDS Spectrum - National HIV Estimates 2019	GPS	Joint United Nations Program on HIV/AIDS (UNAIDS). UNAIDS Spectrum - National HIV Estimates 2019. Geneva, Switzerland: Joint United Nations Program on HIV/AIDS (UNAIDS), 2018.	415978
Burundi	2002	Burundi Annual HIV/AIDS/STIs Epidemiological Surveillance Report 2002	GPS	Ministry of Public Health (Burundi), National AIDS and STDs Program (Burundi). Burundi Annual HIV/AIDS/STIs Epidemiological Surveillance Report 2002. Burundi: National AIDS and STDs Program (Burundi), 2002.	352105
Cameroon	2000, 2002, 2007, 2009, 2012, 2016	UNAIDS Spectrum - National HIV Estimates 2019	GPS	Joint United Nations Program on HIV/AIDS (UNAIDS). UNAIDS Spectrum - National HIV Estimates 2019. Geneva, Switzerland: Joint United Nations Program on HIV/AIDS (UNAIDS), 2018.	415978
Cameroon	2002	Cameroon National HIV Serosurvey 2002	GPS	Ministry of Public Health (Cameroon), National AIDS Control Committee (Cameroon). Cameroon National HIV Serosurvey 2002.	352128

Country	Years	Source	Geographic Detail	Citation	NID*
Cameroon	2016	UNAIDS Spectrum - National HIV Estimates 2017	GPS	Joint United Nations Program on HIV/AIDS (UNAIDS). UNAIDS Spectrum - National HIV Estimates 2017. Geneva, Switzerland: Joint United Nations Program on HIV/AIDS (UNAIDS), 2017.	317607
Central African Republic	2000, 2002, 2006, 2011, 2015	UNAIDS Spectrum - National HIV Estimates 2019	GPS	Joint United Nations Program on HIV/AIDS (UNAIDS). UNAIDS Spectrum - National HIV Estimates 2019. Geneva, Switzerland: Joint United Nations Program on HIV/AIDS (UNAIDS), 2018.	415978
Chad	2002-03, 2009-11, 2013-14	UNAIDS Spectrum - National HIV Estimates 2019	GPS	Joint United Nations Program on HIV/AIDS (UNAIDS). UNAIDS Spectrum - National HIV Estimates 2019. Geneva, Switzerland: Joint United Nations Program on HIV/AIDS (UNAIDS), 2018.	415978
Chad	2000, 2002-03	UNAIDS Spectrum - National HIV Estimates 2016	GPS	Joint United Nations Program on HIV/AIDS (UNAIDS). UNAIDS Spectrum - National HIV Estimates 2016. Geneva, Switzerland: Joint United Nations Program on HIV/AIDS (UNAIDS).	306517
Chad	2000	Chad HIV Prevalence Among Women in ANC Per Sentinel Site 2000	GPS	Ministry of Public Health (Chad). Chad HIV Prevalence Among Women in ANC Per Sentinel Site 2000.	356793
Chad	2002	Chad Annual Report of Epidemiology Service Activities 2002, Epidemiological Surveillance of Second Generation HIV	GPS	Ministry of Public Health (Chad), National AIDS/STI Program (PNLS) (Chad). Chad Annual Report of Epidemiology Service Activities 2002, Epidemiological Surveillance of Second Generation HIV. N'Djamena, Chad: Ministry of Public Health (Chad), 2003.	354940
Chad	2002-03, 2011, 2013-14	UNAIDS Spectrum - National HIV Estimates 2018	GPS	Joint United Nations Program on HIV/AIDS (UNAIDS). UNAIDS Spectrum - National HIV Estimates 2018. Geneva, Switzerland: Joint United Nations Program on HIV/AIDS (UNAIDS), 2018.	365412
Congo	2002, 2005-06, 2011	UNAIDS Spectrum - National HIV Estimates 2019	Admin 1, Admin 2	Joint United Nations Program on HIV/AIDS (UNAIDS). UNAIDS Spectrum - National HIV Estimates 2019. Geneva, Switzerland: Joint United Nations Program on HIV/AIDS (UNAIDS), 2018.	415978
Côte d'Ivoire	2000-02, 2004-05, 2008, 2017	UNAIDS Spectrum - National HIV Estimates 2018	GPS	Joint United Nations Program on HIV/AIDS (UNAIDS). UNAIDS Spectrum - National HIV Estimates 2018. Geneva, Switzerland: Joint United Nations Program on HIV/AIDS (UNAIDS), 2018.	365412

Country	Years	Source	Geographic Detail	Citation	NID*
Côte d'Ivoire	2000-02, 2004-05, 2008, 2017-18	UNAIDS Spectrum - National HIV Estimates 2019	GPS	Joint United Nations Program on HIV/AIDS (UNAIDS). UNAIDS Spectrum - National HIV Estimates 2019. Geneva, Switzerland: Joint United Nations Program on HIV/AIDS (UNAIDS), 2018.	415978
Côte d'Ivoire	2001	UNAIDS Spectrum - National HIV Estimates 2017	GPS	Joint United Nations Program on HIV/AIDS (UNAIDS). UNAIDS Spectrum - National HIV Estimates 2017. Geneva, Switzerland: Joint United Nations Program on HIV/AIDS (UNAIDS), 2017.	317607
Côte d'Ivoire	2005, 2008	UNAIDS Spectrum - National HIV Estimates 2016	GPS	Joint United Nations Program on HIV/AIDS (UNAIDS). UNAIDS Spectrum - National HIV Estimates 2016. Geneva, Switzerland: Joint United Nations Program on HIV/AIDS (UNAIDS).	306517
Democratic Republic of the Congo	2000, 2003-09, 2011-13, 2015, 2017	UNAIDS Spectrum - National HIV Estimates 2019	GPS	Joint United Nations Program on HIV/AIDS (UNAIDS). UNAIDS Spectrum - National HIV Estimates 2019. Geneva, Switzerland: Joint United Nations Program on HIV/AIDS (UNAIDS), 2018.	415978
Djibouti	2002, 2006-10, 2013-14	UNAIDS Spectrum - National HIV Estimates 2019	GPS	Joint United Nations Program on HIV/AIDS (UNAIDS). UNAIDS Spectrum - National HIV Estimates 2019. Geneva, Switzerland: Joint United Nations Program on HIV/AIDS (UNAIDS), 2018.	415978
Equatorial Guinea	2008	Equatorial Guinea HIV Seroprevalence Survey 2008	Admin 1	Castroverde Laboratory (Equatorial Guinea), Institute of Health Carlos III (Spain), Ministry of Science and Innovation (Spain), Reference Center for Endemic Control (CRCE) (Equatorial Guinea), Spanish Agency for International Development Cooperation (AECID). Equatorial Guinea HIV Seroprevalence Survey 2008.	355263
Eritrea	2003, 2005, 2007, 2009, 2011-13, 2015, 2017	UNAIDS Spectrum - National HIV Estimates 2019	GPS	Joint United Nations Program on HIV/AIDS (UNAIDS). UNAIDS Spectrum - National HIV Estimates 2019. Geneva, Switzerland: Joint United Nations Program on HIV/AIDS (UNAIDS), 2018.	415978
Eritrea	2003	Eritrea HIV Sentinel Surveillance Survey 2003	GPS	Ministry of Health (Eritrea). Eritrea HIV Sentinel Surveillance Survey 2003.	355283
Eritrea	2003	Eritrea HIV Prevalence by Health Unit, Site, and Zone 1999-2003	GPS	Ministry of Health (Eritrea). Eritrea HIV Prevalence by Health Unit, Site, and Zone 1999-2003.	355296

Country	Years	Source	Geographic Detail	Citation	NID*
Eritrea	2003, 2005, 2007	Eritrea HIV and Syphilis Sentinel Surveillance Survey in ANC Attendee Women 2007	GPS	Ministry of Health (Eritrea). Eritrea HIV and Syphilis Sentinel Surveillance Survey in ANC Attendee Women 2007.	143203
Eritrea	2003, 2005, 2007, 2009, 2011, 2013, 2015, 2018	UNAIDS Spectrum - National HIV Estimates 2018	GPS	Joint United Nations Program on HIV/AIDS (UNAIDS). UNAIDS Spectrum - National HIV Estimates 2018. Geneva, Switzerland: Joint United Nations Program on HIV/AIDS (UNAIDS), 2018.	365412
Eswatini	2000, 2002, 2004, 2006, 2008, 2010	UNAIDS Spectrum - National HIV Estimates 2018	Admin 1, GPS	Joint United Nations Program on HIV/AIDS (UNAIDS). UNAIDS Spectrum - National HIV Estimates 2018. Geneva, Switzerland: Joint United Nations Program on HIV/AIDS (UNAIDS), 2018.	365412
Ethiopia	2001-03, 2005, 2007, 2009, 2012, 2014, 2016	UNAIDS Spectrum - National HIV Estimates 2018	GPS	Joint United Nations Program on HIV/AIDS (UNAIDS). UNAIDS Spectrum - National HIV Estimates 2018. Geneva, Switzerland: Joint United Nations Program on HIV/AIDS (UNAIDS), 2018.	365412
Ethiopia	2001-03	AIDS in Ethiopia: Fifth Report	GPS	Centers for Disease Control and Prevention (CDC), Ministry of Health (Ethiopia). AIDS in Ethiopia: Fifth Report. Addis Ababa, Ethiopia: Ministry of Health (Ethiopia), 2004.	316592
Ethiopia	2005	AIDS in Ethiopia: Sixth Report	GPS	Federal HIV/AIDS Prevention and Control Office (Ethiopia), Ministry of Health (Ethiopia), President's Emergency Plan for AIDS Relief (PEPFAR). AIDS in Ethiopia: Sixth Report. Addis Ababa, Ethiopia: Ministry of Health (Ethiopia).	143235
Ethiopia	2007, 2009	Ethiopia Antenatal Care Sentinel HIV Surveillance 2009	GPS	Centers for Disease Control and Prevention (CDC), Ethiopian Health and Nutrition Research Center (EHNRI). Ethiopia Antenatal Care Sentinel HIV Surveillance 2009.	355394
Gabon	2001-03, 2007, 2009	UNAIDS Spectrum - National HIV Estimates 2019	GPS	Joint United Nations Program on HIV/AIDS (UNAIDS). UNAIDS Spectrum - National HIV Estimates 2019. Geneva, Switzerland: Joint United Nations Program on HIV/AIDS (UNAIDS), 2018.	415978
Gabon	2001	Gabon HIV/AIDS Incidence, Prevalence, and Mortality 2001-2003	GPS	National AIDS Control Program (Gabon). Gabon HIV/AIDS Incidence, Prevalence, and Mortality 2001-2003. 2003.	355441
Gambia	2001-08, 2011-12, 2014, 2016-17	UNAIDS Spectrum - National HIV Estimates 2019	GPS	Joint United Nations Program on HIV/AIDS (UNAIDS). UNAIDS Spectrum - National HIV Estimates 2019. Geneva, Switzerland: Joint United Nations Program on HIV/AIDS (UNAIDS), 2018.	415978

Country	Years	Source	Geographic Detail	Citation	NID*
Ghana	2000-18	UNAIDS Spectrum - National HIV Estimates 2019	GPS	Joint United Nations Program on HIV/AIDS (UNAIDS). UNAIDS Spectrum - National HIV Estimates 2019. Geneva, Switzerland: Joint United Nations Program on HIV/AIDS (UNAIDS), 2018.	415978
Ghana	2000	Ghana HIV Sentinel Survey 2000	GPS	Ministry of Health (Ghana), National AIDS Control Program (NACP) (Ghana), National Public Health and Reference Laboratory (NHPRL) (Ghana). Ghana HIV Sentinel Survey 2000.	355496
Ghana	2001	Ghana HIV Sentinel Survey 2001	GPS	Ministry of Health (Ghana), National AIDS Control Program (NACP) (Ghana), National Public Health and Reference Laboratory (NHPRL) (Ghana). Ghana HIV Sentinel Survey 2001.	355494
Ghana	2003	Ghana HIV Sentinel Survey 2003	GPS	Ghana Health Service, National AIDS Control Program (NACP) (Ghana), National Public Health and Reference Laboratory (NHPRL) (Ghana), Noguchi Memorial Institute for Medical Research (NMIMR) (Ghana). Ghana HIV Sentinel Survey 2003.	355475
Ghana	2005	Ghana HIV Sentinel Survey 2005	GPS	Ghana Health Service, National AIDS Control Program (NACP) (Ghana), National Public Health and Reference Laboratory (NHPRL) (Ghana), Noguchi Memorial Institute for Medical Research (NMIMR) (Ghana). Ghana HIV Sentinel Survey 2005.	355473
Ghana	2006	Ghana HIV Sentinel Survey 2006	GPS	Ghana Health Service, National AIDS Control Program (NACP) (Ghana), National Public Health and Reference Laboratory (NHPRL) (Ghana), Noguchi Memorial Institute for Medical Research (NMIMR) (Ghana). Ghana HIV Sentinel Survey 2006.	355458
Guinea	2001, 2004, 2008, 2015, 2018	UNAIDS Spectrum - National HIV Estimates 2019	GPS	Joint United Nations Program on HIV/AIDS (UNAIDS). UNAIDS Spectrum - National HIV Estimates 2019. Geneva, Switzerland: Joint United Nations Program on HIV/AIDS (UNAIDS), 2018.	415978
Guinea	2015	UNAIDS Spectrum - National HIV Estimates 2016	GPS	Joint United Nations Program on HIV/AIDS (UNAIDS). UNAIDS Spectrum - National HIV Estimates 2016. Geneva, Switzerland: Joint United Nations Program on HIV/AIDS (UNAIDS).	306517

Country	Years	Source	Geographic Detail	Citation	NID*
Guinea-Bissau	2000-05, 2010, 2014	UNAIDS Spectrum - National HIV Estimates 2019	Admin 2	Joint United Nations Program on HIV/AIDS (UNAIDS). UNAIDS Spectrum - National HIV Estimates 2019. Geneva, Switzerland: Joint United Nations Program on HIV/AIDS (UNAIDS), 2018.	415978
Kenya	2000-11	UNAIDS Spectrum - National HIV Estimates 2018	GPS	Joint United Nations Program on HIV/AIDS (UNAIDS). UNAIDS Spectrum - National HIV Estimates 2018. Geneva, Switzerland: Joint United Nations Program on HIV/AIDS (UNAIDS), 2018.	365412
Kenya	2010	Kenya ANC HIV Sentinel Survey 2010	GPS	Kenya Medical Research Institute (KEMRI), National AIDS and STI Control Programme (NASCOP) (Kenya), National HIV Reference Laboratory (NHRL) (Kenya), National Public Health Laboratory Services, Ministry of Public Health and Sanitation (Kenya), World Health Organization (WHO). Kenya ANC HIV Sentinel Survey 2010.	355605
Kenya	2011	Kenya ANC HIV Sentinel Survey 2011	GPS	Kenya Medical Research Institute (KEMRI), National AIDS and STI Control Programme (NASCOP) (Kenya), National HIV Reference Laboratory (NHRL) (Kenya), National Public Health Laboratory Services, Ministry of Public Health and Sanitation (Kenya), World Health Organization (WHO). Kenya ANC HIV Sentinel Survey 2011.	355607
Lesotho	2000, 2003, 2005, 2009, 2011, 2013, 2016, 2018	UNAIDS Spectrum - National HIV Estimates 2019	GPS	Joint United Nations Program on HIV/AIDS (UNAIDS). UNAIDS Spectrum - National HIV Estimates 2019. Geneva, Switzerland: Joint United Nations Program on HIV/AIDS (UNAIDS), 2018.	415978
Liberia	2006-08, 2011, 2013, 2017	UNAIDS Spectrum - National HIV Estimates 2019	GPS	Joint United Nations Program on HIV/AIDS (UNAIDS). UNAIDS Spectrum - National HIV Estimates 2019. Geneva, Switzerland: Joint United Nations Program on HIV/AIDS (UNAIDS), 2018.	415978
Liberia	2007-08	Liberia HIV Sentinel Survey 2008	GPS	Global Fund to Fight Aids Tuberculosis and Malaria (GFATM), National AIDS and STI Control Program (NACP), Ministry of Health and Social Welfare (Liberia), National Public Health Reference Laboratory (Liberia), United Nations Development Programme (UNDP), World Health Organization (WHO). Liberia HIV Sentinel Survey 2008.	355669

Country	Years	Source	Geographic Detail	Citation	NID*
Madagascar	2003	Madagascar Towards Knowledge of its Epidemic: Analysis of the Epidemiological Profile and Determinants of the HIV Infection	GPS	Executive Secretariat of the National Committee for the Fight against HIV/AIDS (SE CNLS) (Madagascar), Joint United Nations Program on HIV/AIDS (UNAIDS), United States Agency for International Development (USAID), World Bank, World Health Organization (WHO). Madagascar Towards Knowledge of its Epidemic: Analysis of the Epidemiological Profile and Determinants of the HIV Infection. Antananarivo, Madagascar: Executive Secretariat of the National Committee for the Fight against HIV/AIDS (SE CNLS) (Madagascar), 2008.	355675
Madagascar	2005	Madagascar Biological Surveillance Survey 2005	GPS	Centers for Disease Control and Prevention (CDC), French Cooperation, Joint United Nations Program on HIV/AIDS (UNAIDS), Ministry of Health and Family Planning (Madagascar), National Institute of Public and Community Health (INSPC) (Madagascar), Pasteur Institute of Madagascar (IPM), United Nations Children's Fund (UNICEF), United States Agency for International Development (USAID), World Health Organization (WHO). Madagascar Biological Surveillance Survey 2005.	355671
Madagascar	2005, 2007, 2010-18	UNAIDS Spectrum - National HIV Estimates 2019	GPS	Joint United Nations Program on HIV/AIDS (UNAIDS). UNAIDS Spectrum - National HIV Estimates 2019. Geneva, Switzerland: Joint United Nations Program on HIV/AIDS (UNAIDS), 2018.	415978
Malawi	2003	Malawi HIV and Syphilis Sentinel Surveillance of Antenatal Clinic Attendees 2003	GPS	Ministry of Health (Malawi), National AIDS Commission (Malawi). Malawi HIV and Syphilis Sentinel Surveillance of Antenatal Clinic Attendees 2003.	157140
Malawi	2001, 2003, 2005, 2007, 2010	UNAIDS Spectrum - National HIV Estimates 2019	GPS	Joint United Nations Program on HIV/AIDS (UNAIDS). UNAIDS Spectrum - National HIV Estimates 2019. Geneva, Switzerland: Joint United Nations Program on HIV/AIDS (UNAIDS), 2018.	415978
Malawi	2001	HIV/AIDS in Malawi: 2003 Estimates and Implications	GPS	Ministry of Health and Population (Malawi), National AIDS Commission (Malawi). HIV/AIDS in Malawi: 2003 Estimates and Implications. Lilongwe, Malawi: National AIDS Commission (Malawi).	355677

Country	Years	Source	Geographic Detail	Citation	NID*
Malawi	2007	Malawi HIV and Syphilis Sero-Survey of Antenatal Clinic Attendees 2007	GPS	Ministry of Health (Malawi), National AIDS Commission (Malawi). Malawi HIV and Syphilis Sero-Survey of Antenatal Clinic Attendees 2007.	157132
Mali	2002-03, 2005, 2007, 2009, 2012	UNAIDS Spectrum - National HIV Estimates 2019	GPS	Joint United Nations Program on HIV/AIDS (UNAIDS). UNAIDS Spectrum - National HIV Estimates 2019. Geneva, Switzerland: Joint United Nations Program on HIV/AIDS (UNAIDS), 2018.	415978
Mali	2007	Mali HIV and Syphilis Sentinel Surveillance Survey 2007	GPS	Centers for Disease Control and Prevention (CDC), INFO-STAT (Mali), Ministry of Health (Mali), National Institute of Public Health Research (INRSP) (Mali), Sectoral Committee for the Fight Against AIDS (CSLS) (Mali). Mali HIV and Syphilis Sentinel Surveillance Survey 2007.	355931
Mozambique	2000-02, 2004, 2007, 2009, 2011	UNAIDS Spectrum - National HIV Estimates 2019	GPS	Joint United Nations Program on HIV/AIDS (UNAIDS). UNAIDS Spectrum - National HIV Estimates 2019. Geneva, Switzerland: Joint United Nations Program on HIV/AIDS (UNAIDS), 2018.	415978
Namibia	2000, 2002, 2004, 2006, 2008, 2010, 2012, 2014, 2016-17	UNAIDS Spectrum - National HIV Estimates 2019	Admin 2, GPS	Joint United Nations Program on HIV/AIDS (UNAIDS). UNAIDS Spectrum - National HIV Estimates 2019. Geneva, Switzerland: Joint United Nations Program on HIV/AIDS (UNAIDS), 2018.	415978
Namibia	2000, 2002, 2004, 2006, 2008, 2010, 2012, 2014, 2016	UNAIDS Spectrum - National HIV Estimates 2017	GPS, Admin 2	Joint United Nations Program on HIV/AIDS (UNAIDS). UNAIDS Spectrum - National HIV Estimates 2017. Geneva, Switzerland: Joint United Nations Program on HIV/AIDS (UNAIDS), 2017.	317607
Namibia	2000	Namibia Epidemiological Report on HIV/AIDS for the Year 2000	Admin 2	National AIDS Coordination Program (Namibia). Namibia Epidemiological Report on HIV/AIDS for the Year 2000. Namibia: National AIDS Coordination Program (Namibia), 2001.	356159
Namibia	2002	Namibia National HIV Sentinel Survey 2002	Admin 2, GPS	Ministry of Health and Social Services (Namibia), Namibia Institute of Pathology, National AIDS Coordination Program (Namibia). Namibia National HIV Sentinel Survey 2002.	356248
Namibia	2014, 2016	UNAIDS Spectrum - National HIV Estimates 2018	GPS, Admin 2	Joint United Nations Program on HIV/AIDS (UNAIDS). UNAIDS Spectrum - National HIV Estimates 2018. Geneva,	365412

Country	Years	Source	Geographic Detail	Citation	NID*
				Switzerland: Joint United Nations Program on HIV/AIDS (UNAIDS), 2018.	
Niger	2000, 2009, 2012, 2014	UNAIDS Spectrum - National HIV Estimates 2019	GPS	Joint United Nations Program on HIV/AIDS (UNAIDS). UNAIDS Spectrum - National HIV Estimates 2019. Geneva, Switzerland: Joint United Nations Program on HIV/AIDS (UNAIDS), 2018.	415978
Niger	2000	Niger HIV Epidemiological Surveillance Report 2001	GPS	National Program for the Fight Against AIDS/STDs (PNLS/MST) (Niger). Niger HIV Epidemiological Surveillance Report 2001.	356296
Nigeria	2000-03, 2005, 2008, 2010, 2014, 2017	UNAIDS Spectrum - National HIV Estimates 2019	GPS	Joint United Nations Program on HIV/AIDS (UNAIDS). UNAIDS Spectrum - National HIV Estimates 2019. Geneva, Switzerland: Joint United Nations Program on HIV/AIDS (UNAIDS), 2018.	415978
Nigeria	2003	Nigeria National HIV Seroprevalence Sentinel Survey 2003	GPS	Federal Ministry of Health (Nigeria). Nigeria National HIV Seroprevalence Sentinel Survey 2003.	152886
Nigeria	2003, 2005, 2008, 2010, 2014	UNAIDS Spectrum - National HIV Estimates 2018	GPS	Joint United Nations Program on HIV/AIDS (UNAIDS). UNAIDS Spectrum - National HIV Estimates 2018. Geneva, Switzerland: Joint United Nations Program on HIV/AIDS (UNAIDS), 2018.	365412
Nigeria	2005	Nigeria National HIV/Syphilis Seroprevalence Sentinel Survey 2005	GPS	Federal Ministry of Health (Nigeria). Nigeria National HIV/Syphilis Seroprevalence Sentinel Survey 2005.	138348
Nigeria	2005, 2008, 2010	Nigeria National HIV Seroprevalence Sentinel Survey 2010	GPS	Federal Ministry of Health (Nigeria). Nigeria National HIV Seroprevalence Sentinel Survey 2010.	152887
Rwanda	2002-03, 2005, 2007, 2011, 2013	UNAIDS Spectrum - National HIV Estimates 2019	GPS	Joint United Nations Program on HIV/AIDS (UNAIDS). UNAIDS Spectrum - National HIV Estimates 2019. Geneva, Switzerland: Joint United Nations Program on HIV/AIDS (UNAIDS), 2018.	415978
Rwanda	2002-03, 2005, 2007, 2011, 2013	UNAIDS Spectrum - National HIV Estimates 2016	GPS	Joint United Nations Program on HIV/AIDS (UNAIDS). UNAIDS Spectrum - National HIV Estimates 2016. Geneva, Switzerland: Joint United Nations Program on HIV/AIDS (UNAIDS).	306517
Rwanda	2002	Rwanda HIV Sentinel Surveillance Survey 2002	GPS	Centers for Disease Control and Prevention (CDC), IMPACT Rwanda, Ministry of Health (Rwanda). Rwanda HIV Sentinel Surveillance Survey 2002.	356349

Country	Years	Source	Geographic Detail	Citation	NID*
Senegal	2000-01	Senegal HIV Prevalence in Men and Pregnant Women by Region 1989-2002	Admin 1, GPS	Ministry of Health and Prevention (Senegal). Senegal HIV Prevalence in Men and Pregnant Women by Region 1989-2002.	356379
Senegal	2000-07, 2009, 2011, 2014, 2018	UNAIDS Spectrum - National HIV Estimates 2019	Admin 1, GPS	Joint United Nations Program on HIV/AIDS (UNAIDS). UNAIDS Spectrum - National HIV Estimates 2019. Geneva, Switzerland: Joint United Nations Program on HIV/AIDS (UNAIDS), 2018.	415978
Senegal	2002	Senegal Sero-Epidemiological Bulletin on HIV Surveillance No. 10, July 2003	Admin 1, GPS	Ministry of Health and Prevention (Senegal), National Council for the Fight Against AIDS (Senegal). Senegal Sero-Epidemiological Bulletin on HIV Surveillance No. 10, July 2003. Dakar, Senegal: National Council for the Fight Against AIDS (Senegal), 2003.	356380
Senegal	2005-07	Senegal Epidemiological Bulletin on Sentinel Surveillance of HIV and Syphilis in Pregnant Women No. 13, November 2008	Admin 1	Ministry of Health and Prevention (Senegal), National Council for the Fight Against AIDS (Senegal). Senegal Epidemiological Bulletin on Sentinel Surveillance of HIV and Syphilis in Pregnant Women No. 13, November 2008. Dakar, Senegal: National Council for the Fight Against AIDS (Senegal), 2008.	356395
Sierra Leone	2003, 2006-08, 2010, 2018	UNAIDS Spectrum - National HIV Estimates 2019	GPS	Joint United Nations Program on HIV/AIDS (UNAIDS). UNAIDS Spectrum - National HIV Estimates 2019. Geneva, Switzerland: Joint United Nations Program on HIV/AIDS (UNAIDS), 2018.	415978
Sierra Leone	2015	UNAIDS Spectrum - National HIV Estimates 2016	GPS	Joint United Nations Program on HIV/AIDS (UNAIDS). UNAIDS Spectrum - National HIV Estimates 2016. Geneva, Switzerland: Joint United Nations Program on HIV/AIDS (UNAIDS).	306517
Somalia	2004, 2007, 2010-12, 2014, 2016	UNAIDS Spectrum - National HIV Estimates 2019	GPS	Joint United Nations Program on HIV/AIDS (UNAIDS). UNAIDS Spectrum - National HIV Estimates 2019. Geneva, Switzerland: Joint United Nations Program on HIV/AIDS (UNAIDS), 2018.	415978
South Africa	2000-14	UNAIDS Spectrum - National HIV Estimates 2017	Admin 1, Admin 2, Admin 3	Joint United Nations Program on HIV/AIDS (UNAIDS). UNAIDS Spectrum - National HIV Estimates 2017. Geneva, Switzerland: Joint United Nations Program on HIV/AIDS (UNAIDS), 2017.	317607

Country	Years	Source	Geographic Detail	Citation	NID*
South Africa	2006-08	South Africa National Antenatal Sentinel HIV and Syphilis Prevalence Survey 2008	Admin 2, Admin 3	Department of Health (South Africa). South Africa National Antenatal Sentinel HIV and Syphilis Prevalence Survey 2008.	356401
South Sudan	2007, 2009, 2012	UNAIDS Spectrum - National HIV Estimates 2016	GPS	Joint United Nations Program on HIV/AIDS (UNAIDS). UNAIDS Spectrum - National HIV Estimates 2016. Geneva, Switzerland: Joint United Nations Program on HIV/AIDS (UNAIDS).	306517
South Sudan	2007, 2009, 2012, 2017	UNAIDS Spectrum - National HIV Estimates 2019	GPS	Joint United Nations Program on HIV/AIDS (UNAIDS). UNAIDS Spectrum - National HIV Estimates 2019. Geneva, Switzerland: Joint United Nations Program on HIV/AIDS (UNAIDS), 2018.	415978
Sudan	2004-05, 2007, 2009-10	UNAIDS Spectrum - National HIV Estimates 2019	GPS	Joint United Nations Program on HIV/AIDS (UNAIDS). UNAIDS Spectrum - National HIV Estimates 2019. Geneva, Switzerland: Joint United Nations Program on HIV/AIDS (UNAIDS), 2018.	415978
Uganda	2000-05, 2007, 2009-12, 2014, 2016, 2018	UNAIDS Spectrum - National HIV Estimates 2019	GPS	Joint United Nations Program on HIV/AIDS (UNAIDS). UNAIDS Spectrum - National HIV Estimates 2019. Geneva, Switzerland: Joint United Nations Program on HIV/AIDS (UNAIDS), 2018.	415978
Uganda	2000-01, 2003-07, 2009	Uganda HIV/AIDS Epidemiological Surveillance Report 2010	GPS	Centers for Disease Control and Prevention (CDC), Medical Research Council (Uganda), Ministry of Health (Uganda), National Institute of Health (Italy), Rakai Health Sciences Program (Uganda), STD/AIDS Control Program, Ministry of Health (Uganda), Uganda Virus Research Institute. Uganda HIV/AIDS Epidemiological Surveillance Report 2010. Uganda: STD/AIDS Control Program, Ministry of Health (Uganda), 2010.	321955
Uganda	2005-07	Uganda HIV/AIDS Epidemiological Surveillance Report 2005-2007	GPS	Centers for Disease Control and Prevention (CDC), Joint United Nations Program on HIV/AIDS (UNAIDS), Medical Research Council (Uganda), Ministry of Health (Uganda), National Institute of Health (Italy), Rakai Health Sciences Program (Uganda), STD/AIDS Control Program, Ministry of Health (Uganda), Uganda AIDS Commission, Uganda Virus Research Institute, United States Agency for International	356783

Country	Years	Source	Geographic Detail	Citation	NID*
				Development (USAID), World Health Organization (WHO). Uganda HIV/AIDS Epidemiological Surveillance Report 2005-2007. Uganda: STD/AIDS Control Program, Ministry of Health (Uganda), 2009.	
Tanzania	2000-03, 2005, 2008, 2010-11, 2014, 2017-18	UNAIDS Spectrum - National HIV Estimates 2019	GPS	Joint United Nations Program on HIV/AIDS (UNAIDS). UNAIDS Spectrum - National HIV Estimates 2019. Geneva, Switzerland: Joint United Nations Program on HIV/AIDS (UNAIDS), 2018.	415978
Tanzania	2000, 2005, 2008-09, 2011, 2017	UNAIDS Spectrum - National HIV Estimates 2018	GPS	Joint United Nations Program on HIV/AIDS (UNAIDS). UNAIDS Spectrum - National HIV Estimates 2018. Geneva, Switzerland: Joint United Nations Program on HIV/AIDS (UNAIDS), 2018.	365412
Tanzania	2000-01, 2003, 2005, 2008, 2011	UNAIDS Spectrum - National HIV Estimates 2017	GPS	Joint United Nations Program on HIV/AIDS (UNAIDS). UNAIDS Spectrum - National HIV Estimates 2017. Geneva, Switzerland: Joint United Nations Program on HIV/AIDS (UNAIDS), 2017.	317607
Togo	2001, 2003-04, 2006-11, 2014, 2016	UNAIDS Spectrum - National HIV Estimates 2019	GPS	Joint United Nations Program on HIV/AIDS (UNAIDS). UNAIDS Spectrum - National HIV Estimates 2019. Geneva, Switzerland: Joint United Nations Program on HIV/AIDS (UNAIDS), 2018.	415978
Togo	2011	Togo National HIV and Syphilis Sentinel Surveillance Survey 2011-2012	GPS	Directorate General of Health (DGS) (Togo), Joint United Nations Program on HIV/AIDS (UNAIDS), National Program for the Fight Against HIV/AIDS (PNLS) (Togo), World Health Organization (WHO). Togo National HIV and Syphilis Sentinel Surveillance Survey 2011-2012.	356774
Zambia	2002, 2004, 2006, 2008, 2011, 2018	UNAIDS Spectrum - National HIV Estimates 2019	GPS	Joint United Nations Program on HIV/AIDS (UNAIDS). UNAIDS Spectrum - National HIV Estimates 2019. Geneva, Switzerland: Joint United Nations Program on HIV/AIDS (UNAIDS), 2018.	415978
Zimbabwe	2000-02, 2004, 2006, 2009, 2012, 2016	UNAIDS Spectrum - National HIV Estimates 2019	GPS	Joint United Nations Program on HIV/AIDS (UNAIDS). UNAIDS Spectrum - National HIV Estimates 2019. Geneva, Switzerland: Joint United Nations Program on HIV/AIDS (UNAIDS), 2018.	415978

Table S3: HIV and covariates surveys excluded from this analysis

Country	Year(s)	Name	Citation	Excluded from	Rationale
Burundi	2002	Burundi National HIV Infection Seroprevalence Survey 2001	Training Center for Medical and Infectious Diseases (CEFORMI) (Burundi). Burundi National HIV Infection Seroprevalence Survey 2001.	HIV Prevalence	This survey report did not include sex and age disaggregated data.
Cameroon	2017	Cameroon Population-based HIV Impact Assessment 2017-2018	Ministry of Health, Cameroon, Centers for Disease Control and Prevention (CDC), and ICAP at Columbia University. Cameroon Population-based HIV Impact Assessment (CAMPHIA) 2017-18: Summary Sheet. Yaoundé, Cameroon, Atlanta, Georgia and New York, New York, USA: Ministry of Health, CDC and ICAP. July 2018.	HIV Prevalence	This survey report did not include sex and age disaggregated data.
Chad	2005	Chad National HIV/AIDS Seroprevalence Survey 2005	Conseil Santé (France), National Institute for Statistics, Economic and Demographic Studies (INSEED) (Chad). Chad National HIV/AIDS Seroprevalence Survey 2005.	HIV Prevalence	This survey report did not include sex and age disaggregated data.
Côte d'Ivoire	2017	Côte d'Ivoire Population-based HIV Impact Assessment 2017-2018	Ministry of Health and Public Hygiene, Cote d'Ivoire, Centers for Disease Control and Prevention (CDC), and ICAP at Columbia Universito. Cote d'Ivoire Population-based HIV Impact Assessment (CIPHIA) 2017-18: Summary Sheet. Abidjan, Cote d'Ivoire, Atlanta, Georgia and New York, New York, USA: Ministry of Health, CDC and ICAP. July 2018.	HIV Prevalence	This survey report did not include sex and age disaggregated data.

Country	Year(s)	Name	Citation	Excluded from	Rationale
Eswatini	2011	Swaziland HIV Incidence Measurement Survey (SHIMS): Descriptive Data Tables, August 2014	Centers for Disease Control and Prevention (CDC), ICAP, Columbia University Mailman School of Public Health, Ministry of Health (Swaziland). Swaziland HIV Incidence Measurement Survey (SHIMS): Descriptive Data Tables, August 2014. New York, New York: ICAP, Columbia University Mailman School of Public Health, 2014.	HIV Prevalence	This survey report did not include sex and age disaggregated data.
Ethiopia	2017-2018	Ethiopia Population-based HIV Impact Assessment 2017-2018	Centers for Disease Control and Prevention (CDC), Central Statistical Agency (Ethiopia), Ethiopian Public Health Institute (EPHI), Government of Ethiopia, ICAP, Columbia University Mailman School of Public Health, Ministry of Health (Ethiopia). Ethiopia Population-Based HIV Impact Assessment 2017-2018.	HIV Prevalence	This survey report only covered urban areas.
Guinea-Bissau	2010	Guinea-Bissau HIV Prevalence Survey 2010	Institute of Hygiene and Tropical Medicine, Nova University of Lisbon (Portugal), National Institute of Public Health (INASA) (Guinea-Bissau). Guinea-Bissau HIV Prevalence Survey 2010.	HIV Prevalence	This survey report did not include sex and age disaggregated data.

Country	Year(s)	Name	Citation	Excluded from	Rationale
Nigeria	2018	Nigeria HIV/AIDS Indicator and Impact Survey 2018	Federal Ministry of Health (Nigeria), Government of Nigeria, National Agency for the Control of AIDS (NACA) (Nigeria), University of Maryland. Nigeria HIV/AIDS Indicator and Impact Survey 2018.	HIV Prevalence	This survey report did not include sex and age disaggregated data at a granular level.
Nigeria	2007, 2012	Nigeria National HIV/AIDS and Reproductive Health Survey	Federal Ministry of Health (Nigeria), National Agency for the Control of AIDS (NACA) (Nigeria), National Bureau of Statistics (Nigeria), National Population Commission (NPC), Nigerian Institute of Medical Research, Society for Family Health (Nigeria), University College Hospital, Ibadan. Nigeria National HIV/AIDS and Reproductive Health Survey 2007, 2012.	HIV Prevalence	These surveys provided low data quality and data validation issues.
Sierra Leone	2002	Sierra Leone HIV/AIDS Seroprevalence and Behavioral Risk Factor Survey 2002	Centers for Disease Control and Prevention (CDC), Government of Sierra Leone, World Bank. Sierra Leone HIV/AIDS Seroprevalence and Behavioral Risk Factor Survey 2002.	HIV Prevalence	This survey report did not include sex and age disaggregated data. .
Sierra Leone	2005	Sierra Leone National Population-based HIV Seroprevalence Survey 2005	Ministry of Health and Sanitation (Sierra Leone), National HIV/AIDS Secretariat (NAS) (Sierra Leone), Nimba Research and Consulting (NiRC) (Ghana), Statistics Sierra Leone. Sierra Leone National Population-Based HIV Seroprevalence Survey 2005.	HIV Prevalence	This survey report did not include sex and age disaggregated data.

Country	Year(s)	Name	Citation	Excluded from	Rationale
South Africa	2017	South Africa National HIV Prevalence, Incidence, Behavior and Communication Survey 2017	Centers for Disease Control and Prevention (CDC), Human Sciences Research Council, National Institute for Communicable Diseases (South Africa), South African Medical Research Council. South Africa National HIV Prevalence, Incidence, Behavior and Communication Survey 2017.	HIV Prevalence	This survey report did not include sex and age disaggregated data.
All countries (Burkina Faso, Chad, Comoros, Congo, Côte d'Ivoire, Ethiopia, Ghana, Kenya, Malawi, Mali, Mauritania, Namibia, Senegal, South Africa, Swaziland, Zambia, Zimbabwe)	All years (2002-04)	World Health Surveys (WHS)	World Health Organization (WHO). Geneva, Switzerland: World Health Organization (WHO), 2005.	All covariates	Geographic patterns of cluster locations in available microdata are inconsistent with reported maps. Additionally, estimates were considered outliers compared to other surveys in the same countries.
Benin	2009	Benin Household Living Conditions Survey	Benin Household Living Conditions Survey 2009.	'in union'	Estimates for 'in union' were implausibly high compared to estimates from other surveys (2006 and 2011 DHS).
Botswana	2001 and 2004	Botswana AIDS Impact Surveys	Central Statistics Office (Botswana), Ministry of Health (Botswana). Botswana AIDS Impact Survey 2001. Gaborone, Botswana: Statistics Botswana.	'partner away'	These surveys did not ask the 'partner away' question for respondents who had previously indicated they were living with a partner

Country	Year(s)	Name	Citation	Excluded from	Rationale
			Central Statistics Office (Botswana). Botswana AIDS Impact Survey 2004. Gaborone, Botswana: Statistics Botswana.		and survey estimates were implausibly low compared to estimates from the same survey series in later years (2008 and 2013).
Burkina Faso	2005	Burkina Faso CWIQ Survey	National Institute of Statistics and Demography (Burkina Faso), World Bank. Burkina Faso Core Welfare Indicators Questionnaire Survey 2005. Ouagadougou, Burkina Faso: National Institute of Statistics and Demography (Burkina Faso)	'in union'	Implausibly low estimates as compared to DHS 2005
Burundi	2005	Multiple Indicator Cluster Survey (MICS)	United Nations Children's Fund (UNICEF), Burundi Institute of Statistics and Economic Studies, United Nations Population Fund (UNFPA). Burundi Multiple Indicator Cluster Survey 2005. New York, United States: United Nations Children's Fund (UNICEF)	'in union'	Implausibly low estimates as compared to other surveys
Cameroon	2006	Multiple Indicator Cluster Survey (MICS)	United Nations Children's Fund (UNICEF), National Institute of Statistics (Cameroon). Cameroon Multiple Indicator Cluster Survey 2006. New York, United States: United Nations Children's Fund (UNICEF).	'had intercourse'	Estimates for 'had intercourse' were implausibly lower than estimates from other surveys (2004 DHS and 2011 DHS) and, in comparison with published reports for this survey, it appears missing responses were marked as false values for this

Country	Year(s)	Name	Citation	Excluded from	Rationale
					indicator in reports with no explanation.
Central African Republic	2000	Multiple Indicator Cluster Survey (MICS)	Division of Statistics and Economic Studies (Central African Republic), Ministry of Economy, Planning and International Cooperation (Central African Republic), United Nations Children's Fund (UNICEF). Central African Republic Multiple Indicator Cluster Survey 2000. New York, United States: United Nations Children's Fund (UNICEF)	'in union'	Implausibly low estimates as compared to other surveys in later years.
Democratic Republic of the Congo	2013-14, 2014, 2015, 2015-16, and 2016	Performance, Monitoring, and Accountability Survey (PMA)	Performance Monitoring and Accountability 2020 (PMA2020) Project, University of Kinshasa School of Public Health, Tulane University School of Public Health and Tropical Medicine. 2013, 2014, 2015, 2016. Democratic Republic of the Congo. Baltimore, MD: PMA2020, Bill & Melinda Gates Institute for Population and Reproductive Health, Johns Hopkins Bloomberg School of Public Health.	'in union'	Estimates for 'in union' were implausibly high compared to estimates from other surveys (2001 and 2010 MICS, 2007 and 2013-2014 DHS).
Ghana	2007-08	Multiple Indicator Cluster Survey (MICS)	Ghana Statistical Service, Ministry of Health (Ghana), United Nations Children's Fund (UNICEF). Ghana District Multiple Indicator Cluster Survey 2007-2008.	'had intercourse'	Survey data had zero explicit 'never had intercourse' responses, so survey estimates are implausibly low compared to estimates from other surveys (2003, 2008,

Country	Year(s)	Name	Citation	Excluded from	Rationale
					and 2014 DHS and 2006 MICS).
Kenya	2005-2006	Kenya Integrated HH Budget Survey	Kenya National Bureau of Statistics. Kenya Integrated Household Budget Survey Tabular Data 2005-2006. Nairobi, Kenya: Kenya National Bureau of Statistics.	'in union'	Implausibly low estimates as compared to other surveys.
Kenya	2015	STEPS Noncommunicable Disease Risk Factors Survey	Kenya National Bureau of Statistics, Ministry of Health (Kenya), World Health Organization (WHO). Kenya STEPS Noncommunicable Disease Risk Factors Survey 2015.	'in union'	Estimates for 'in union' were implausibly high compared to estimates from other surveys (2008 DHS and 2014 and 2015 PMA).
Malawi	2013	Malawi Integrated HH Survey	National Statistical Office of Malawi. Malawi Integrated Household Survey 2013 (IHS3 - Year: 2). Dataset downloaded from [http://go.worldbank.org/NOXNI9YDS0] on [September 15, 2015]	'in union'	Implausibly low estimates as compared to other surveys in neighbouring years.
Mali	2014-15	Living Standards Measurement Study – Integrated Survey on Agriculture (LSMS – ISA)	Ministry of Rural Development (Mali), National Institute of Statistics (INSTAT) (Mali), World Bank. Mali Agricultural Integrated Economic Survey 2014-2015. Washington DC, United States: World Bank.	'in union'	Estimates for 'in union' were implausibly low compared to other surveys (2012 DHS, 2015 MICS, and 2015 MIS).
Namibia	2009-2010	Namibia Household Income and Expenditure Survey	Namibia Statistics Agency. Namibia Household Income and Expenditure Survey 2009-2010. Windhoek, Namibia: Namibia Statistics Agency.	'in union'	Estimates for 'in union' were implausibly low compared to other surveys (2006 and 2013 DHS).

Country	Year(s)	Name	Citation	Excluded from	Rationale
Nigeria	2007 and 2012	National HIV/AIDS and Reproductive Health Survey	Federal Ministry of Health (Nigeria), National Bureau of Statistics (Nigeria), National Population Commission (NPC), Society for Family Health (Nigeria), University College Hospital, Ibadan. Nigeria National HIV/AIDS and Reproductive Health Surveys 2007 and 2012.	'male circumcision', 'sti symptoms', 'had intercourse', 'multiple partners-men', multiple partners – women'	Estimates for 'male circumcision' were implausibly lower than estimates from other survey series (2008 DHS, 2013 DHS, and 2016-2017 MICS).
Nigeria	2008-10	Living Standards Survey	National Bureau of Statistics (Nigeria). Nigeria Living Standards Survey 2008-2010. Abuja, Nigeria: National Bureau of Statistics (Nigeria).	'male circumcision'	Estimates for 'male circumcision' were implausibly lower than estimates from other survey series (2008 DHS, 2013 DHS, and 2016-2017 MICS).
Nigeria	2012-2013	Nigeria General HH Survey	National Bureau of Statistics (Nigeria). Nigeria General Household Survey 2012-2013. Washington DC, United States: World Bank	'in union'	Implausibly low estimates as compared to other surveys in neighbouring years.
South Africa	All years	International Social Science Survey Programme (ISSP)	ISSP Research Group (2009): International Social Survey Programme: Leisure Time and Sports - ISSP 2007. GESIS Data Archive, Cologne. ZA4850 Data file version 2.0.0, doi:10.4231/1.10079.<br><br>ISSP Research Group (2008): International Social Survey Programme: Role of Government IV - ISSP 2006. GESIS Data Archive, Cologne. ZA4700	'in union'	Estimates for 'in union' were consistently low for this survey series compared to all other surveys in country.

Country	Year(s)	Name	Citation	Excluded from	Rationale
			Data file Version 1.0.0, doi:10.4232/1.4700.<br><br>ISSP Research Group (2012): International Social Survey Programme: Religion III - ISSP 2008. GESIS Data Archive, Cologne. ZA4950 Data file Version 2.2.0, doi:10.4232/1.11334.<br><br>ISSP Research Group (2017): International Social Survey Programme: Social Inequality IV - ISSP 2009. GESIS Data Archive, Cologne. ZA5400 Data file Version 4.0.0, doi:10.4232/1.12777.<br><br>ISSP Research Group (2012): International Social Survey Programme: Environment III - ISSP 2010. GESIS Data Archive, Cologne. ZA5500 Data file Version 2.0.0, doi:10.4232/1.11418.<br><br>ISSP Research Group (2016): International Social Survey Programme: Family and Changing Gender Roles IV - ISSP 2012. GESIS Data Archive, Cologne. ZA5900 Data file Version 4.0.0, doi:10.4232/1.12661.<br><br>ISSP Research Group (2012): International Social Survey Programme: Citizenship - ISSP 2004. GESIS Data Archive, Cologne. ZA3950 Data file Version 1.3.0, doi:10.4232/1.11372.		

Country	Year(s)	Name	Citation	Excluded from	Rationale
			ISSP Research Group (2013): International Social Survey Programme: Work Orientation III - ISSP 2005. GESIS Data Archive, Cologne. ZA4350 Data file Version 2.0.0, doi:10.4232/1.11648.<br><br>ISSP Research Group (2015): International Social Survey Programme: National Identity III - ISSP 2013. GESIS Data Archive, Cologne. ZA5950 Data file Version 2.0.0, doi:10.4232/1.12312.<br><br>ISSP Research Group (2016): International Social Survey Programme: Citizenship II - ISSP 2014. GESIS Data Archive, Cologne. ZA6670 Data file Version 2.0.0, doi:10.4232/1.12590.		
Uganda	2009-10	Uganda Living Standards Measurement Survey – Integrated Survey on Agriculture (LSMS – ISA)	Uganda Bureau of Statistics. Uganda Living Standards Measurement Survey - Integrated Survey on Agriculture 2009-2010. Washington DC, United States: World Bank.	'in union'	Estimates for 'in union' were implausibly low compared to other surveys (2006 and 2011 DHS, 2011 AIS).

Table S4: Sources for pre-existing covariates

Covariate	Temporal resolution	Source	Reference
Travel time to nearest settlement >50,000 inhabitants	Static	Malaria Atlas Project, Big Data Institute, Nuffield Department of Medicine, University of Oxford	Weiss, D. J. et al. A global map of travel time to cities to assess inequalities in accessibility in 2015. Nature 533, 333-336 (2018).
Night-time lights	Annual, 2000-13	NOAA DMSP	P Savory et al. Intercalibration and Gaussian Process Modeling of Nighttime Lights Imagery for Measuring Urbanization Trends in Africa 2000–2013. Remote Sens. 9 (2017).
Urbanicity	Annual, 2000-16	European Commission/ GHS	Pesaresi, M. et al. Operating procedure for the production of the Global Human Settlement Layer from Landsat data of the epochs 1975, 1990, 2000, and 2014. Publications Office of the European Union (2016).
Malaria incidence	Annual, 2000-17	Malaria Atlas Project	Bhatt, S. et al. The effect of malaria control on Plasmodium falciparum in Africa between 2000 and 2015. Nature 526, 207–211 (2015).
Population	Annual, 2000-18	WorldPop	Lloyd, C. T. et al. Global spatio-temporally harmonised datasets for producing high-resolution gridded population distribution datasets, Big Earth Data, DOI: 10.1080/20964471.2019.1625151 (2019).<br><br>World Pop. Get data. Available at: http://www.worldpop.org.uk/data/get_data/. (Accessed: 3rd March 2020)

23 Table S5: HIV covariate survey data (2000–2018)

Country	Years	Name	Type	Geographic Detail	Male Circumcision	STI Symptoms	Had Intercourse	Partner Away	Condom Last Time	Multiple Partners in Year (Males	Multiple Partners in Year (Females)	In Union	Citation	NID
Angola	2001	Angola Multiple Indicator Cluster Survey 2001	Survey microdata	Admin 1								X	National Institute of Statistics (Angola), United Nations Children's Fund (UNICEF). Angola Multiple Indicator Cluster Survey 2001. New York, United States of America: United Nations Children's Fund (UNICEF).	687
Angola	2006	Angola Core Welfare Indicators Questionnaire Survey 2005	Survey microdata	Admin 1								X	National Institute of Statistics (Angola). Angola Core Welfare Indicators Questionnaire Survey 2005. Luanda, Angola: National Institute of Statistics (Angola).	151566
Angola	2011	Angola Core Welfare Indicators Questionnaire Survey 2011	Survey microdata	Admin 1								X	Ministry of Planning and Territorial Development (Angola), National Institute of Statistics (Angola). Angola Core Welfare Indicators Questionnaire Survey 2011. Luanda, Angola: National Institute of Statistics (Angola).	151568

Country	Years	Name	Type	Geographic Detail	Male Circumcision	STI Symptoms	Had Intercourse	Partner Away	Condom Last Time	Multiple Partners in Year (Males	Multiple Partners in Year (Females)	In Union	Citation	NID
Angola	2015-16	Angola Demographic and Health Survey 2015-2016	Survey microdata	GPS	X	X	X	X	X	X	X	X	ICF International, Ministry of Health (Angola), National Institute of Statistics (Angola), United Nations Children's Fund (UNICEF). Angola Demographic and Health Survey 2015-2016. Fairfax, United States of America: ICF International, 2017.	218555
Benin	2001	Benin Demographic and Health Survey 2001	Survey microdata	GPS			X	X			X		National Institute of Statistics and Economic Analysis (INSAE) (Benin), ORC Macro. Benin Demographic and Health Survey 2001. Fairfax, United States of America: ICF International.	18950
Benin	2006	Benin Demographic and Health Survey 2006	Survey microdata	Admin 1	X	X	X	X	X	X		X	Macro International, Inc, National Institute of Statistics and Economic Analysis (INSAE) (Benin), National Program Against AIDS (PNLS) (Benin). Benin Demographic and Health Survey 2006. Fairfax, United States of America: ICF International.	18959

Country	Years	Name	Type	Geographic Detail	Male Circumcision	STI Symptoms	Had Intercourse	Partner Away	Condom Last Time	Multiple Partners in Year (Males	Multiple Partners in Year (Females)	In Union	Citation	NID
Benin	2011-12	Benin Demographic and Health Survey 2011-2012	Survey microdata	GPS	X	X	X	X	X	X	X	X	ICF International, National Institute of Statistics and Economic Analysis (INSAE) (Benin), National Program Against AIDS (PNLS) (Benin). Benin Demographic and Health Survey 2011-2012. Fairfax, United States of America: ICF International, 2014.	79839
Benin	2012	Benin Integrated Modular Survey on Household Living Conditions 2011-2012	Survey microdata	Admin 1								X	Danish International Development Agency (DANIDA), German Society for International Cooperation (GIZ), National Institute of Statistics and Economic Analysis (INSAE) (Benin). Benin Integrated Modular Survey on Household Living Conditions 2011-2012. Cotonou, Benin: National Institute of Statistics and Economic Analysis (INSAE) (Benin).	151805
Benin	2014	Benin Multiple Indicator Cluster Survey 2014	Survey microdata	Admin 1	X		X		X	X	X	X	National Institute of Statistics and Economic Analysis (INSAE) (Benin), United Nations Children's Fund (UNICEF). Benin Multiple Indicator Cluster Survey 2014. New York, United States of America: United Nations Children's Fund (UNICEF), 2017.	206075

Country	Years	Name	Type	Geographic Detail	Male Circumcision	STI Symptoms	Had Intercourse	Partner Away	Condom Last Time	Multiple Partners in Year (Males	Multiple Partners in Year (Females)	In Union	Citation	NID
Benin	2017-18	Benin Demographic and Health Survey 2017-2018	Survey microdata	GPS	X	X	X	X	X	X	X	X	Hubert Koutoukou Maga National University Hospital Center (CNHU-HKM)(Benin), ICF International, National Institute of Statistics and Economic Analysis (INSAE) (Benin), National Malaria Control Program, Ministry of Health (Benin), Permanent Secretariat of the Food Council and Nutrition (SP-CAN)(Benin). Benin Demographic and Health Survey 2017-2018. Fairfax, United States of America: ICF International, 2018.	218565
Botswana	2000	Botswana Multiple Indicator Cluster Survey 2000	Survey microdata	Admin 1								X	Central Statistics Office (Botswana), United Nations Children's Fund (UNICEF). Botswana Multiple Indicator Cluster Survey 2000. New York, United States of America: United Nations Children's Fund (UNICEF), 2015.	1404
Botswana	2001	Botswana AIDS Impact Survey 2001	Survey microdata	Admin 2, Admin <1	X	X	X		X			X	Central Statistics Office (Botswana), Ministry of Health (Botswana). Botswana AIDS Impact Survey 2001. Gaborone, Botswana: Statistics Botswana.	22112

Country	Years	Name	Type	Geographic Detail	Male Circumcision	STI Symptoms	Had Intercourse	Partner Away	Condom Last Time	Multiple Partners in Year (Males	Multiple Partners in Year (Females)	In Union	Citation	NID
Botswana	2004	Botswana AIDS Impact Survey 2004	Survey microdata	GPS	X	X	X		X	X	X	X	Central Statistics Office (Botswana). Botswana AIDS Impact Survey 2004. Gaborone, Botswana: Statistics Botswana.	22114
Botswana	2007-08	Botswana Family Health Survey 2007-2008	Survey microdata	Admin 3, GPS			X					X	Central Statistics Office (Botswana). Botswana Family Health Survey 2007-2008. Gaborone, Botswana: Central Statistics Office (Botswana), 2009.	22125
Botswana	2008	Botswana AIDS Impact Survey 2008	Survey microdata	GPS	X	X	X	X	X	X	X	X	Central Statistics Office (Botswana), National AIDS Coordinating Agency (Botswana). Botswana AIDS Impact Survey 2008. Gaborone, Botswana: Statistics Botswana, 2015.	22116

Country	Years	Name	Type	Geographic Detail	Male Circumcision	STI Symptoms	Had Intercourse	Partner Away	Condom Last Time	Multiple Partners in Year (Males	Multiple Partners in Year (Females)	In Union	Citation	NID
Botswana	2010	Botswana Core Welfare Indicators Survey 2009-2010	Survey microdata	Admin 3, GPS								X	Central Statistics Office (Botswana). Botswana Core Welfare Indicators Survey 2009-2010.	151536
Botswana	2013	Botswana AIDS Impact Survey 2013	Survey microdata	GPS	X	X	X		X	X	X	X	Ministry of Health (Botswana), National AIDS Coordinating Agency (Botswana), Statistics Botswana. Botswana AIDS Impact Survey 2013. Gaborone, Botswana: Statistics Botswana, 2015.	134753
Burkina Faso	2003	Burkina Faso Core Welfare Indicators Questionnaire Survey 2003	Survey microdata	Admin 3, GPS								X	National Institute of Statistics and Demography (Burkina Faso), World Bank. Burkina Faso Core Welfare Indicators Questionnaire Survey 2003. Ouagadougou, Burkina Faso: National Institute of Statistics and Demography (Burkina Faso).	1855

Country	Years	Name	Type	Geographic Detail	Male Circumcision	STI Symptoms	Had Intercourse	Partner Away	Condom Last Time	Multiple Partners in Year (Males	Multiple Partners in Year (Females)	In Union	Citation	NID
Burkina Faso	2003	Burkina Faso Demographic and Health Survey 2003	Survey microdata	GPS	X	X	X	X	X	X	X	X	Macro International, Inc, National Institute of Statistics and Demography (Burkina Faso). Burkina Faso Demographic and Health Survey 2003. Fairfax, United States of America: ICF International.	19088
Burkina Faso	2006	Burkina Faso Multiple Indicator Cluster Survey 2006	Survey microdata	GPS			X				X		National Institute of Statistics and Demography (Burkina Faso), United Nations Children's Fund (UNICEF). Burkina Faso Multiple Indicator Cluster Survey 2006. New York, United States of America: United Nations Children's Fund (UNICEF).	1927
Burkina Faso	2007	Burkina Faso Core Welfare Indicators Questionnaire Survey 2007	Survey microdata	Admin 3, GPS								X	National Institute of Statistics and Demography (INSD). Burkina Faso Core Welfare Indicators Questionnaire Survey 2007. Ouagadougou, Burkina Faso: National Institute of Statistics and Demography (INSD), 2008.	18499

Country	Years	Name	Type	Geographic Detail	Male Circumcision	STI Symptoms	Had Intercourse	Partner Away	Condom Last Time	Multiple Partners in Year (Males	Multiple Partners in Year (Females)	In Union	Citation	NID
Burkina Faso	2010	Burkina Faso Demographic and Health Survey 2010-2011	Survey microdata	GPS	X	X	X	X	X	X	X	X	ICF Macro, Ministry of Health (Burkina Faso), National Institute of Statistics and Demography (Burkina Faso). Burkina Faso Demographic and Health Survey 2010-2011. Fairfax, United States of America: ICF International.	19133
Burkina Faso	2014	Burkina Faso Malaria Indicator Survey 2014	Survey microdata	GPS								X	ICF International, National Center for Research and Training on Malaria (CNRFP) (Burkina Faso), National Institute of Statistics and Demography (Burkina Faso), National Program for the Fight Against Malaria (PNLP) (Burkina Faso). Burkina Faso Malaria Indicator Survey 2014. Fairfax, United States of America: ICF International, 2015.	188785
Burkina Faso	2014	Burkina Faso Performance Monitoring and Accountability 2020 Survey, Round 1 2014	Survey microdata	Admin 1			X	X				X	Institut Supérieur des Sciences de la Population, Université Joseph Ki-Zerbo, Ouagadougou, Burkina Faso, and The Bill & Melinda Gates Institute for Population and Reproductive Health at The Johns Hopkins Bloomberg School of Public Health. Performance Monitoring and Accountability 2020 (PMA2020) Survey Round 1, PMA2014/Burkina Faso-R1. 2014. Ouagadougou, Burkina Faso and Baltimore, Maryland, USA.	257044

Country	Years	Name	Type	Geographic Detail	Male Circumcision	STI Symptoms	Had Intercourse	Partner Away	Condom Last Time	Multiple Partners in Year (Males	Multiple Partners in Year (Females)	In Union	Citation	NID
Burkina Faso	2015	Burkina Faso Performance Monitoring and Accountability 2020 Survey, Round 2 2015	Survey microdata	Admin 1			X	X				X	Institut Supérieur des Sciences de la Population, Université Joseph Ki-Zerbo, Ouagadougou, Burkina Faso, and The Bill & Melinda Gates Institute for Population and Reproductive Health at The Johns Hopkins Bloomberg School of Public Health. Performance Monitoring and Accountability 2020 (PMA2020) Survey Round 2, PMA2015/Burkina Faso-R2. 2015. Ouagadougou, Burkina Faso and Baltimore, Maryland, USA.	257045
Burkina Faso	2016	Burkina Faso Performance Monitoring and Accountability 2020 Survey, Round 3 2016	Survey microdata	Admin 1			X	X				X	Institut Supérieur des Sciences de la Population, Université Joseph Ki-Zerbo, Ouagadougou, Burkina Faso, and The Bill & Melinda Gates Institute for Population and Reproductive Health at The Johns Hopkins Bloomberg School of Public Health. Performance Monitoring and Accountability 2020 (PMA2020) Survey Round 3, PMA2016/Burkina Faso-R3. 2016. Ouagadougou, Burkina Faso and Baltimore, Maryland, USA.	285993
Burkina Faso	2016	Burkina Faso Performance Monitoring and Accountability 2020 Survey, Round 4 2016-2017	Survey microdata	Admin 1			X	X				X	Institut Supérieur des Sciences de la Population, Université Joseph Ki-Zerbo, Ouagadougou, Burkina Faso, and The Bill & Melinda Gates Institute for Population and Reproductive Health at The Johns Hopkins Bloomberg School of Public Health. Performance Monitoring and Accountability 2020 (PMA2020) Survey Round 4, PMA2016/Burkina Faso-R4. 2016. Ouagadougou, Burkina Faso and Baltimore, Maryland, USA.	307751

Country	Years	Name	Type	Geographic Detail	Male Circumcision	STI Symptoms	Had Intercourse	Partner Away	Condom Last Time	Multiple Partners in Year (Males	Multiple Partners in Year (Females)	In Union	Citation	NID
Burkina Faso	2018	Burkina Faso Performance Monitoring and Accountability 2020 Survey, Round 6 2018-2019	Survey microdata	Admin 1			X					X	Institut Supérieur des Sciences de la Population, Université Joseph Ki-Zerbo, Ouagadougou, Burkina Faso, and The Bill & Melinda Gates Institute for Population and Reproductive Health at The Johns Hopkins Bloomberg School of Public Health. Performance Monitoring and Accountability 2020 (PMA2020) Survey Round 6, PMA2018/Burkina Faso-R6. 2018. Ouagadougou, Burkina Faso and Baltimore, Maryland, USA.	407829
Burundi	2000	Burundi Multiple Indicator Cluster Survey 2000	Survey microdata	Admin 1								X	Burundi Institute of Statistics and Economic Studies, United Nations Children's Fund (UNICEF). Burundi Multiple Indicator Cluster Survey 2000. New York, United States: United Nations Children's Fund (UNICEF).	1994
Burundi	2005	Burundi Multiple Indicator Cluster Survey 2005	Survey microdata	Admin 3			X		X	X			United Nations Children's Fund (UNICEF), Burundi Institute of Statistics and Economic Studies, United Nations Population Fund (UNFPA). Burundi Multiple Indicator Cluster Survey 2005. New York, United States: United Nations Children's Fund (UNICEF).	1981

Country	Years	Name	Type	Geographic Detail	Male Circumcision	STI Symptoms	Had Intercourse	Partner Away	Condom Last Time	Multiple Partners in Year (Males	Multiple Partners in Year (Females)	In Union	Citation	NID
Burundi	2010-11	Burundi Demographic and Health Survey 2010-2011	Survey microdata	GPS	X	X	X		X	X	X	X	Burundi Institute of Statistics and Economic Studies, ICF International, Ministry of Public Health and the Fight Against AIDS (Burundi). Burundi Demographic and Health Survey 2010-2011. Fairfax, United States of America: ICF International, 2012.	30431
Burundi	2016-17	Burundi Demographic and Health Survey 2016-2017	Survey microdata	GPS	X	X	X		X	X	X	X	Burundi Institute of Statistics and Economic Studies, ICF International, Ministry of Public Health and the Fight Against AIDS (Burundi). Burundi Demographic and Health Survey 2016-2017. Fairfax, United States of America: ICF International, 2018.	286766
Cameroon	2000	Cameroon Multiple Indicator Cluster Survey 2000	Survey microdata	Admin 1								X	Directorate of Statistics and National Accounts, Ministry of Economics and Finance (Cameroon), United Nations Children's Fund (UNICEF). Cameroon Multiple Indicator Cluster Survey 2000. New York, United States of America: United Nations Children's Fund (UNICEF).	2053

Country	Years	Name	Type	Geographic Detail	Male Circumcision	STI Symptoms	Had Intercourse	Partner Away	Condom Last Time	Multiple Partners in Year (Males	Multiple Partners in Year (Females)	In Union	Citation	NID
Cameroon	2001	Cameroon Household Survey 2001	Survey microdata	Admin 1, Admin 7								X	National Institute of Statistics (Cameroon), Directorate of Statistics and National Accounts, Ministry of Economics and Finance (Cameroon), AFRISTAT. Cameroon Household Survey 2001. Yaounde, Cameroon: National Institute of Statistics (Cameroon).	2039
Cameroon	2004	Cameroon Demographic and Health Survey 2004	Survey microdata	GPS	X	X	X	X	X	X	X	X	Macro International, Inc, National Institute of Statistics (Cameroon). Cameroon Demographic and Health Survey 2004. Fairfax, United States of America: ICF International.	19211
Cameroon	2006	Cameroon Multiple Indicator Cluster Survey 2006	Survey microdata	Admin 1, Admin 3							X	X	United Nations Children's Fund (UNICEF), National Institute of Statistics (Cameroon). Cameroon Multiple Indicator Cluster Survey 2006. New York, United States: United Nations Children's Fund (UNICEF).	2063

Country	Years	Name	Type	Geographic Detail	Male Circumcision	STI Symptoms	Had Intercourse	Partner Away	Condom Last Time	Multiple Partners in Year (Males	Multiple Partners in Year (Females)	In Union	Citation	NID
Cameroon	2011	Cameroon Demographic and Health Survey 2011	Survey microdata	GPS	X	X	X	X	X	X	X	X	ICF International, Ministry of Economy, Planning and Regional Development (Cameroon), Ministry of Public Health (Cameroon), National Institute of Statistics (Cameroon), Pasteur Center of Cameroon. Cameroon Demographic and Health Survey 2011. Fairfax, United States of America: ICF International.	19274
Cameroon	2014	Cameroon Multiple Indicator Cluster Survey 2014	Survey microdata	Admin 1, Admin 3			X		X	X	X	X	Ministry of Public Health (Cameroon), National Institute of Statistics (Cameroon), United Nations Children's Fund (UNICEF). Cameroon Multiple Indicator Cluster Survey 2014. New York, United States of America: United Nations Children's Fund (UNICEF), 2017.	244455
Central African Republic	2006	Central African Republic Multiple Indicator Cluster Survey 2006	Survey microdata	Admin 1	X		X		X	X	X	X	United Nations Children's Fund (UNICEF). Central African Republic Multiple Indicator Cluster Survey 2006. New York, United States: United Nations Children's Fund (UNICEF).	2223

Country	Years	Name	Type	Geographic Detail	Male Circumcision	STI Symptoms	Had Intercourse	Partner Away	Condom Last Time	Multiple Partners in Year (Males	Multiple Partners in Year (Females)	In Union	Citation	NID
Central African Republic	2010	Central African Republic Multiple Indicator Cluster Survey 2010-2011	Survey microdata	Admin 1			X		X	X	X	X	Central African Institute of Statistics, Economic and Social Studies (ICASEES) (Central African Republic), ICF International. Central African Republic Multiple Indicator Cluster Survey 2010-2011. Fairfax, United States of America: ICF International, 2013.	82832
Chad	2000	Chad Multiple Indicator Cluster Survey 2000	Survey microdata	Admin 1								X	United Nations Children's Fund (UNICEF), Census Bureau (Chad), National Institute of Statistical, Economic and Demographic Studies (Chad). Chad Multiple Indicator Cluster Survey 2000. New York, United States: United Nations Children's Fund (UNICEF).	2244
Chad	2004	Chad Demographic and Health Survey 2004	Survey microdata	Admin 1	X	X	X	X	X	X	X	X	Macro International, Inc, National Institute for Statistics, Economic and Demographic Studies (INSEED) (Chad). Chad Demographic and Health Survey 2004. Fairfax, United States of America: ICF International.	19315

Country	Years	Name	Type	Geographic Detail	Male Circumcision	STI Symptoms	Had Intercourse	Partner Away	Condom Last Time	Multiple Partners in Year (Males	Multiple Partners in Year (Females)	In Union	Citation	NID
Chad	2010	Chad Multiple Indicator Cluster Survey 2010	Survey microdata	Admin 2			X				X		Ministry of Planning, Economy, and International Cooperation (Chad), National Institute for Statistics, Economic and Demographic Studies (INSEED) (Chad), United Nations Children's Fund (UNICEF). Chad Multiple Indicator Cluster Survey 2010. New York, United States of America: United Nations Children's Fund (UNICEF), 2014.	76701
Chad	2014-15	Chad Demographic and Health Survey 2014-2015	Survey microdata	GPS	X	X	X	X	X	X	X	X	ICF International, National Institute for Statistics, Economic and Demographic Studies (INSEED) (Chad). Chad Demographic and Health Survey 2014-2015. Fairfax, United States of America: ICF International, 2016.	157025
Comoros	2000	Comoros Multiple Indicator Cluster Survey 2000	Survey microdata	Admin 1								X	United Nations Development Programme (UNDP), United Nations Children's Fund (UNICEF). Comoros Multiple Indicator Cluster Survey 2000. New York, United States: United Nations Children's Fund (UNICEF).	3114

Country	Years	Name	Type	Geographic Detail	Male Circumcision	STI Symptoms	Had Intercourse	Partner Away	Condom Last Time	Multiple Partners in Year (Males	Multiple Partners in Year (Females)	In Union	Citation	NID
Comoros	2012	Comoros Demographic and Health Survey 2012-2013	Survey microdata	GPS	X	X	X	X	X	X	X	X	General Directorate of Statistics and Forecasting (Comoros), ICF International. Comoros Demographic and Health Survey 2012-2013. Fairfax, United States of America: ICF International.	76850
Congo	2005	Congo Demographic and Health Survey 2005	Survey microdata	Admin 1		X	X	X	X	X	X	X	Macro International, Inc, National Center for Statistics and Economic Studies (Congo, Rep.). Congo Demographic and Health Survey 2005. Fairfax, United States of America: ICF International.	19391
Congo	2009	Congo AIDS Indicator Survey 2009	Survey microdata	Admin 1	X	X	X	X	X	X	X	X	ICF Macro, National Center for Statistics and Economic Studies (Congo, Rep.). Congo AIDS Indicator Survey 2009. Fairfax, United States of America: ICF International.	3133

Country	Years	Name	Type	Geographic Detail	Male Circumcision	STI Symptoms	Had Intercourse	Partner Away	Condom Last Time	Multiple Partners in Year (Males	Multiple Partners in Year (Females)	In Union	Citation	NID
Congo	2011	Congo Demographic and Health Survey 2011-2012	Survey microdata	Admin 1	X	X	X	X	X	X	X	X	ICF International, Ministry of Health (Congo, Rep.), National Center for Statistics and Economic Studies (Congo, Rep.). Congo Demographic and Health Survey 2011-2012. Fairfax, United States of America: ICF International.	56151
Congo	2014-15	Congo Multiple Indicator Cluster Survey 2014-2015	Survey microdata	Admin 1	X								National Institute of Statistics (INS) (Congo, Rep.), United Nations Children's Fund (UNICEF). Congo Multiple Indicator Cluster Survey 2014-2015. New York, United States of America: United Nations Children's Fund (UNICEF), 2018.	234733
Côte d'Ivoire	2000	Côte d'Ivoire Multiple Indicator Cluster Survey 2000	Survey microdata	Admin >1								X	National School for Statistics and Economics Applied (ENSEA), United Nations Children's Fund (UNICEF), United Nations Educational, Scientific and Cultural Organization (UNESCO). Côte d'Ivoire Multiple Indicator Cluster Survey 2000. New York, United States of America: United Nations Children's Fund (UNICEF).	26444

Country	Years	Name	Type	Geographic Detail	Male Circumcision	STI Symptoms	Had Intercourse	Partner Away	Condom Last Time	Multiple Partners in Year (Males	Multiple Partners in Year (Females)	In Union	Citation	NID
Côte d'Ivoire	2005	Côte d'Ivoire AIDS Indicator Survey 2005	Survey microdata	Admin >1	X	X	X	X	X	X	X	X	CDC Retro-CI, Ministry of the Fight Against AIDS (Côte d'Ivoire), National Institute of Statistics (Côte d'Ivoire), ORC Macro. Côte d'Ivoire AIDS Indicator Survey 2005. Fairfax, United States of America: ICF International.	56148
Côte d'Ivoire	2011-12	Côte d'Ivoire Demographic and Health Survey 2011-2012	Survey microdata	GPS	X	X	X	X	X	X	X	X	ICF International, Ministry of the Fight Against AIDS (Côte d'Ivoire), National Institute of Statistics (Côte d'Ivoire). Côte d'Ivoire Demographic and Health Survey 2011-2012. Fairfax, United States of America: ICF International.	18533
Côte d'Ivoire	2016	Cote d'Ivoire Multiple Indicator Cluster Survey 2016	Survey microdata	Admin >1			X		X	X	X	X	National Institute of Statistics (Côte d'Ivoire), United Nations Children's Fund (UNICEF). Cote d'Ivoire Multiple Indicator Cluster Survey 2016. New York, United States of America: United Nations Children's Fund (UNICEF), 2018.	218611

Country	Years	Name	Type	Geographic Detail	Male Circumcision	STI Symptoms	Had Intercourse	Partner Away	Condom Last Time	Multiple Partners in Year (Males	Multiple Partners in Year (Females)	In Union	Citation	NID
Côte d'Ivoire	2018	Cote d'Ivoire Performance Monitoring and Accountability 2020 Survey, Round 2 2018	Survey microdata	Admin 2			X					X	Institut National de la Statistique de la Côte d'Ivoire (INS-Côte d'Ivoire), La Direction de Coordination du Programme National de Santé de la Mère et de l'Enfant (DC-PNSME), and The Bill & Melinda Gates Institute for Population and Reproductive Health at The Johns Hopkins Bloomberg School of Public Health. Performance Monitoring and Accountability 2020 (PMA2020) Survey round 2, PMA2018/Cote d'Ivoire-R2. 2018. Abidjan, Cote d'Ivoire and Baltimore, Maryland, USA.	398717
Democratic Republic of the Congo	2001	Democratic Republic of the Congo Multiple Indicator Cluster Survey 2001	Survey microdata	Admin 1			X					X	Ministry of Planning and Reconstruction (Congo, DR), United Nations Children's Fund (UNICEF). Congo, DR Multiple Indicator Cluster Survey 2001. New York, United States: United Nations Children's Fund (UNICEF).	3161
Democratic Republic of the Congo	2007	Democratic Republic of the Congo Demographic and Health Survey 2007	Survey microdata	GPS	X	X	X	X	X	X	X	X	Macro International, Inc, Ministry of Planning (Congo, DR). Democratic Republic of the Congo Demographic and Health Survey 2007. Fairfax, United States of America: ICF International.	19381

Country	Years	Name	Type	Geographic Detail	Male Circumcision	STI Symptoms	Had Intercourse	Partner Away	Condom Last Time	Multiple Partners in Year (Males	Multiple Partners in Year (Females)	In Union	Citation	NID
Democratic Republic of the Congo	2010	Democratic Republic of the Congo Multiple Indicator Cluster Survey 2010	Survey microdata	GPS			X				X		National Statistical Institute (Congo, DR), Ministry of Planning (Congo, DR), United Nations Children's Fund (UNICEF). Congo, DR Multiple Indicator Cluster Survey 2010. New York, United States: United Nations Children's Fund (UNICEF).	26998
Democratic Republic of the Congo	2013	Democratic Republic of the Congo - Kinshasa Performance Monitoring and Accountability 2020 Survey, Round 1 2013-2014	Survey microdata	Admin 1			X	X					Tulane University School of Public Health, University of Kinshasa School of Public Health and The Bill & Melinda Gates Institute for Population and Reproductive Health at The Johns Hopkins Bloomberg School of Public Health. Performance Monitoring and Accountability 2020 (PMA2020) Survey round 1, PMA2013/DRC-R1 (Kinshasa). 2013. DRC and Baltimore, Maryland, USA.	257822
Democratic Republic of the Congo	2013-14	Democratic Republic of the Congo Demographic and Health Survey 2013-2014	Survey microdata	GPS		X	X	X	X	X	X	X	ICF International, Ministry of Planning and Monitoring Implementation of the Revolution of Modernity (Congo, DR), Ministry of Public Health (Congo, DR), National Institute of Statistics (Congo, DR). Democratic Republic of the Congo Demographic and Health Survey 2013-2014. Fairfax, United States of America: ICF International, 2014.	76878

Country	Years	Name	Type	Geographic Detail	Male Circumcision	STI Symptoms	Had Intercourse	Partner Away	Condom Last Time	Multiple Partners in Year (Males	Multiple Partners in Year (Females)	In Union	Citation	NID
Democratic Republic of the Congo	2014	Democratic Republic of the Congo - Kinshasa Performance Monitoring and Accountability 2020 Survey, Round 2 2014	Survey microdata	GPS			X	X					Tulane University School of Public Health, University of Kinshasa School of Public Health and The Bill & Melinda Gates Institute for Population and Reproductive Health at The Johns Hopkins Bloomberg School of Public Health. Performance Monitoring and Accountability 2020 (PMA2020) Survey round 2, PMA2014/DRC-R2 (Kinshasa). 2014. Kinshasa, DRC and Baltimore, Maryland, USA.	257823
Democratic Republic of the Congo	2015	Democratic Republic of the Congo - Kinshasa Performance Monitoring and Accountability 2020 Survey, Round 3 2015	Survey microdata	GPS			X	X					Tulane University School of Public Health, University of Kinshasa School of Public Health and The Bill & Melinda Gates Institute for Population and Reproductive Health at The Johns Hopkins Bloomberg School of Public Health. Performance Monitoring and Accountability 2020 (PMA2020) Survey round 3, PMA2015/DRC-R3 (Kinshasa). 2015. Kinshasa, DRC and Baltimore, Maryland, USA.	257826
Democratic Republic of the Congo	2015	Democratic Republic of the Congo - Kinshasa and Kongo Central Performance Monitoring and Accountability 2020 Survey, Round 4 2015-2016	Survey microdata	GPS			X	X					Tulane University School of Public Health, University of Kinshasa School of Public Health and The Bill & Melinda Gates Institute for Population and Reproductive Health at The Johns Hopkins Bloomberg School of Public Health. Performance Monitoring and Accountability 2020 (PMA2020) Survey round 4, PMA2015/DRC-R4 (Kinshasa & Kongo Central). 2015. Kinshasa, DRC and Baltimore, Maryland, USA.	286019

Country	Years	Name	Type	Geographic Detail	Male Circumcision	STI Symptoms	Had Intercourse	Partner Away	Condom Last Time	Multiple Partners in Year (Males	Multiple Partners in Year (Females)	In Union	Citation	NID
Democratic Republic of the Congo	2016	Democratic Republic of the Congo - Kinshasa and Kongo Central Performance Monitoring and Accountability 2020 Survey, Round 5 2016	Survey microdata	GPS			X	X					Tulane University School of Public Health, University of Kinshasa School of Public Health and The Bill & Melinda Gates Institute for Population and Reproductive Health at The Johns Hopkins Bloomberg School of Public Health. Performance Monitoring and Accountability 2020 (PMA2020) Survey round 5, PMA2016/DRC-R5 (Kinshasa & Kongo Central). 2016. Kinshasa, DRC and Baltimore, Maryland, USA.	286054
Equatorial Guinea	2000	Equatorial Guinea Multiple Indicator Cluster Survey 2000	Survey microdata	Admin 1								X	Ministry of Planning, Economic Development and Public Investment (Equatorial Guinea), United Nations Children's Fund (UNICEF). Equatorial Guinea Multiple Indicator Cluster Survey 2000. New York, United States of America: United Nations Children's Fund (UNICEF).	3655
Eritrea	2002	Eritrea Demographic and Health Survey 2002	Survey microdata	Admin 1			X	X				X	Macro International, Inc, National Statistics and Evaluation Office (Eritrea). Eritrea Demographic and Health Survey 2002. Fairfax, United States of America: ICF International.	19539

Country	Years	Name	Type	Geographic Detail	Male Circumcision	STI Symptoms	Had Intercourse	Partner Away	Condom Last Time	Multiple Partners in Year (Males	Multiple Partners in Year (Females)	In Union	Citation	NID
Eritrea	2010	Eritrea Population and Health Survey 2010	Survey report	Admin 1	X								Kenya Medical Research Institute (KEMRI), National Statistics Office (Eritrea), The Fafo Research Foundation. Eritrea Population and Health Survey 2010.	249999
Eswatini	2000	Swaziland Multiple Indicator Cluster Survey 2000	Survey microdata	Admin 1								X	Central Statistical Office (Swaziland), United Nations Children's Fund (UNICEF). Swaziland Multiple Indicator Cluster Survey 2000. New York, United States of America: United Nations Children's Fund (UNICEF).	12320
Eswatini	2006-07	Swaziland Demographic and Health Survey 2006-2007	Survey microdata	GPS	X	X	X	X	X	X	X	X	Central Statistical Office (Swaziland), Macro International, Inc. Swaziland Demographic and Health Survey 2006-2007. Fairfax, United States of America: ICF International.	20829

Country	Years	Name	Type	Geographic Detail	Male Circumcision	STI Symptoms	Had Intercourse	Partner Away	Condom Last Time	Multiple Partners in Year (Males	Multiple Partners in Year (Females)	In Union	Citation	NID
Eswatini	2010	Swaziland Multiple Indicator Cluster Survey 2010	Survey microdata	Admin 1	X	X	X		X	X	X	X	Central Statistical Office (Swaziland), United Nations Children's Fund (UNICEF). Swaziland Multiple Indicator Cluster Survey 2010. New York, United States of America: United Nations Children's Fund (UNICEF).	30325
Eswatini	2014	Swaziland Multiple Indicator Cluster Survey 2014	Survey microdata	Admin 1	X		X		X	X	X	X	Central Statistical Office (Swaziland), United Nations Children's Fund (UNICEF), United Nations Educational, Scientific and Cultural Organization (UNESCO), United Nations Population Fund (UNFPA). Swaziland Multiple Indicator Cluster Survey 2014. New York, United States of America: United Nations Children's Fund (UNICEF), 2016.	200707
Eswatini	2017	Swaziland HIV Incidence Measurement Survey 2 2016-2017	Survey microdata	Admin 1	X		X		X	X	X	X	Centers for Disease Control and Prevention (CDC), Central Statistical Office (Swaziland), Environmental Health Department, Ministry of Health (Swaziland), Health Promotion Unit (Swaziland), ICAP, Columbia University Mailman School of Public Health, Ministry of Health (Swaziland), National Emergency Response Council on HIV and AIDS (NERCHA) (Swaziland), Swaziland Health Laboratory Services, Ministry of Health (SHLS), Swaziland Health Research (SHR), Swaziland National AIDS Programme (SNAP). Swaziland HIV Incidence Measurement Survey 2 2016-2017.	415531

Country	Years	Name	Type	Geographic Detail	Male Circumcision	STI Symptoms	Had Intercourse	Partner Away	Condom Last Time	Multiple Partners in Year (Males	Multiple Partners in Year (Females)	In Union	Citation	NID
Ethiopia	2000	Ethiopia Demographic and Health Survey 2000	Survey microdata	GPS			X	X	X	X	X	X	Central Statistical Agency (Ethiopia), ORC Macro. Ethiopia Demographic and Health Survey 2000. Calverton, United States of America: ORC Macro, 2001.	19571
Ethiopia	2004	Ethiopia Rural Socioeconomic Survey 2011-2012	Survey microdata	GPS								X	Central Statistical Agency (Ethiopia), World Bank. Ethiopia Rural Socioeconomic Survey 2011-2012. Washington DC, United States of America: World Bank.	93848
Ethiopia	2005	Ethiopia Demographic and Health Survey 2005	Survey microdata	GPS	X	X	X	X	X	X	X	X	Macro International, Inc, Population and Housing Census Commissions Office (PHCCO). Ethiopia Demographic and Health Survey 2005. Fairfax, United States of America: ICF International.	19557

Country	Years	Name	Type	Geographic Detail	Male Circumcision	STI Symptoms	Had Intercourse	Partner Away	Condom Last Time	Multiple Partners in Year (Males	Multiple Partners in Year (Females)	In Union	Citation	NID
Ethiopia	2006-07	Ethiopia Socioeconomic Survey 2013-2014	Survey microdata	GPS								X	Central Statistical Agency (Ethiopia), World Bank. Ethiopia Socioeconomic Survey 2013-2014. Washington DC, United States of America: World Bank, 2015.	235215
Ethiopia	2008	Ethiopia Socioeconomic Survey 2015-2016	Survey microdata	GPS								X	Central Statistical Agency (Ethiopia), World Bank. Ethiopia Socioeconomic Survey 2015-2016. Washington DC, United States of America: World Bank, 2015.	286657
Ethiopia	2010-11	Ethiopia Demographic and Health Survey 2010-2011	Survey microdata	GPS	X	X	X	X	X	X	X	X	Central Statistical Agency (Ethiopia), ICF Macro, Ministry of Health (Ethiopia). Ethiopia Demographic and Health Survey 2010-2011. Fairfax, United States of America: ICF International.	21301

Country	Years	Name	Type	Geographic Detail	Male Circumcision	STI Symptoms	Had Intercourse	Partner Away	Condom Last Time	Multiple Partners in Year (Males	Multiple Partners in Year (Females)	In Union	Citation	NID
Ethiopia	2014	Ethiopia Performance Monitoring and Accountability 2020 Survey, Round 1 2014	Survey microdata	Admin 1			X	X				X	Addis Ababa University School of Public Health and The Bill & Melinda Gates Institute for Population and Reproductive Health at The Johns Hopkins Bloomberg School of Public Health. Performance Monitoring and Accountability 2020 (PMA2020) Survey round 1, PMA2014/Ethiopia-R1. 2014. Ethiopia and Baltimore, Maryland, USA.	153503
Ethiopia	2014	Ethiopia Performance Monitoring and Accountability 2020 Survey, Round 2 2014	Survey microdata	Admin 1			X	X				X	Addis Ababa University School of Public Health and The Bill & Melinda Gates Institute for Population and Reproductive Health at The Johns Hopkins Bloomberg School of Public Health. Performance Monitoring and Accountability 2020 (PMA2020) Survey round 2, PMA2014/Ethiopia-R2. 2014. Ethiopia and Baltimore, Maryland, USA.	256175
Ethiopia	2015	Ethiopia Performance Monitoring and Accountability 2020 Survey, Round 3 2015	Survey microdata	Admin 1			X	X				X	Addis Ababa University School of Public Health and The Bill & Melinda Gates Institute for Population and Reproductive Health at The Johns Hopkins Bloomberg School of Public Health. Performance Monitoring and Accountability 2020 (PMA2020) Survey round 3, PMA2015/Ethiopia-R3. 2015. Ethiopia and Baltimore, Maryland, USA.	256176

Country	Years	Name	Type	Geographic Detail	Male Circumcision	STI Symptoms	Had Intercourse	Partner Away	Condom Last Time	Multiple Partners in Year (Males	Multiple Partners in Year (Females)	In Union	Citation	NID
Ethiopia	2016	Ethiopia Demographic and Health Survey 2016	Survey microdata	GPS	X	X	X	X	X	X	X	X	Central Statistical Agency (Ethiopia), ICF International. Ethiopia Demographic and Health Survey 2016. Fairfax, United States of America: ICF International, 2017.	218568
Ethiopia	2016	Ethiopia Performance Monitoring and Accountability 2020 Survey, Round 4 2016	Survey microdata	Admin 1			X	X				X	Addis Ababa University School of Public Health and The Bill & Melinda Gates Institute for Population and Reproductive Health at The Johns Hopkins Bloomberg School of Public Health. Performance Monitoring and Accountability 2020 (PMA2020) Survey round 4, PMA2016/Ethiopia-R4. 2016. Ethiopia and Baltimore, Maryland, USA.PMA2020) Survey round 3, PMA2016/Burkina Faso-R3. 2016. Ouagadougou, Burkina Faso and Baltimore, Maryland, USA.	285891
Ethiopia	2017	Ethiopia Performance Monitoring and Accountability 2020 Survey, Round 5 2017	Survey microdata	Admin 1			X	X				X	Addis Ababa University School of Public Health and The Bill & Melinda Gates Institute for Population and Reproductive Health at The Johns Hopkins Bloomberg School of Public Health. Performance Monitoring and Accountability 2020 (PMA2020) Survey round 5, PMA2017/Ethiopia-R5. 2017. Ethiopia and Baltimore, Maryland, USA.	347050

Country	Years	Name	Type	Geographic Detail	Male Circumcision	STI Symptoms	Had Intercourse	Partner Away	Condom Last Time	Multiple Partners in Year (Males	Multiple Partners in Year (Females)	In Union	Citation	NID
Ethiopia	2018	Ethiopia Performance Monitoring and Accountability 2020 Survey, Round 6 2018	Survey microdata	GPS			X					X	Addis Ababa University School of Public Health and The Bill & Melinda Gates Institute for Population and Reproductive Health at The Johns Hopkins Bloomberg School of Public Health. Performance Monitoring and Accountability 2020 (PMA2020) Survey round 6, PMA2018/Ethiopia-R6. 2018. Ethiopia and Baltimore, Maryland, USA.	398720
Gabon	2000	Gabon Demographic and Health Survey 2000-2001	Survey microdata	Admin 2			X	X	X			X	General Directorate of Statistics and Economic Studies (Gabon), Macro International, Inc. Gabon Demographic and Health Survey 2000-2001. Fairfax, United States of America: ICF International.	19579
Gabon	2012	Gabon Demographic and Health Survey 2012	Survey microdata	GPS	X	X	X	X	X	X	X	X	General Directorate of Statistics (Gabon), ICF International, Ministry of Economy, Employment and Sustainable Development (Gabon), Ministry of Health (Gabon). Gabon Demographic and Health Survey 2012. Fairfax, United States of America: ICF International, 2013.	76706

Country	Years	Name	Type	Geographic Detail	Male Circumcision	STI Symptoms	Had Intercourse	Partner Away	Condom Last Time	Multiple Partners in Year (Males	Multiple Partners in Year (Females)	In Union	Citation	NID
Gambia	2000	Gambia Multiple Indicator Cluster Survey 2000	Survey microdata	Admin 1								X	Central Statistics Department (Gambia), United Nations Children's Fund (UNICEF). Gambia Multiple Indicator Cluster Survey 2000. New York, United States: United Nations Children's Fund (UNICEF).	3922
Gambia	2010	Gambia Multiple Indicator Cluster Survey 2010	Survey microdata	Admin 1			X				X		Gambia Bureau of Statistics (GBOS), United Nations Children's Fund (UNICEF). Gambia Multiple Indicator Cluster Survey 2010. New York, United States of America: United Nations Children's Fund (UNICEF), 2018.	91506
Gambia	2013	Gambia Demographic and Health Survey 2013	Survey microdata	Admin 2		X	X	X	X	X	X	X	Gambia Bureau of Statistics (GBOS), ICF International, Ministry of Health and Social Welfare (The Gambia). Gambia Demographic and Health Survey 2013. Fairfax, United States of America: ICF International, 2015.	77384

Country	Years	Name	Type	Geographic Detail	Male Circumcision	STI Symptoms	Had Intercourse	Partner Away	Condom Last Time	Multiple Partners in Year (Males	Multiple Partners in Year (Females)	In Union	Citation	NID
Gambia	2018	Gambia Multiple Indicator Cluster Survey 2018	Survey microdata	Admin 1			X		X			X	Gambia Bureau of Statistics (GBOS), United Nations Children's Fund (UNICEF). Gambia Multiple Indicator Cluster Survey 2018. New York, United States of America: United Nations Children's Fund (UNICEF), 2019.	424884
Ghana	2003	Ghana Demographic and Health Survey 2003	Survey microdata	GPS	X	X	X	X	X	X	X	X	Ghana Statistical Service, Macro International, Inc. Ghana Demographic and Health Survey 2003. Fairfax, United States of America: ICF International.	19627
Ghana	2006	Ghana Multiple Indicator Cluster Survey 2006	Survey microdata	Admin 1			X		X	X	X	X	Ministry of Health (MOH) (Ghana), Ghana Statistical Service and United Nations Children's Fund (UNICEF). Ghana Multiple Indicator Cluster Survey 2006. New York, United States: United Nations Children's Fund (UNICEF).	4694

Country	Years	Name	Type	Geographic Detail	Male Circumcision	STI Symptoms	Had Intercourse	Partner Away	Condom Last Time	Multiple Partners in Year (Males	Multiple Partners in Year (Females)	In Union	Citation	NID
Ghana	2008	Ghana Demographic and Health Survey 2008	Survey microdata	GPS	X	X	X	X	X	X	X	X	Ghana Statistical Service, Macro International, Inc, Ministry of Health (Ghana). Ghana Demographic and Health Survey 2008. Fairfax, United States of America: ICF International.	21188
Ghana	2010	Ghana - Accra Multiple Indicator Cluster Survey 2010-2011	Survey microdata	GPS			X		X	X	X	X	Institute of Statistical, Social and Economic Research, University of Ghana, United Nations Children's Fund (UNICEF). Ghana - Accra Multiple Indicator Cluster Survey 2010-2011. New York, United States of America: United Nations Children's Fund (UNICEF), 2014.	56241
Ghana	2011	Ghana Multiple Indicator Cluster Survey 2011	Survey microdata	GPS			X		X	X	X	X	Centers for Disease Control and Prevention (CDC), Ghana Statistical Service, Government of Japan, ICF Macro, Ministry of Health (Ghana), Navrongo Health Research Centre, United Nations Children's Fund (UNICEF), United Nations Population Fund (UNFPA), United States Agency for International Development (USAID). Ghana Multiple Indicator Cluster Survey 2011. New York, United States of America: United Nations Children's Fund (UNICEF), 2013.	63993

Country	Years	Name	Type	Geographic Detail	Male Circumcision	STI Symptoms	Had Intercourse	Partner Away	Condom Last Time	Multiple Partners in Year (Males	Multiple Partners in Year (Females)	In Union	Citation	NID
Ghana	2013	Ghana Living Standards Measurement Survey 2012-2013	Survey microdata	Admin 1								X	Ghana Statistical Service, World Bank. Ghana Living Standards Measurement Survey 2012-2013. Accra, Ghana: Ghana Statistical Service.	165101
Ghana	2013	Ghana Performance Monitoring and Accountability 2020 Survey, Round 1 2013	Survey microdata	Admin 1			X	X				X	Kwame Nkrumah University of Science & Technology School of Medicine and The Bill & Melinda Gates Institute for Population and Reproductive Health at The Johns Hopkins Bloomberg School of Public Health. Performance Monitoring and Accountability 2020 (PMA2020) Survey round 1, PMA2013/Ghana-R1. 2013. Ghana and Baltimore, Maryland, USA.	197904
Ghana	2014	Ghana Demographic and Health Survey 2014	Survey microdata	GPS	X	X	X	X	X	X	X	X	Ghana Health Service, Ghana Statistical Service, ICF International. Ghana Demographic and Health Survey 2014. Fairfax, United States of America: ICF International, 2016.	157027

Country	Years	Name	Type	Geographic Detail	Male Circumcision	STI Symptoms	Had Intercourse	Partner Away	Condom Last Time	Multiple Partners in Year (Males	Multiple Partners in Year (Females)	In Union	Citation	NID
Ghana	2014	Ghana Performance Monitoring and Accountability 2020 Survey, Round 2 2014	Survey microdata	Admin 1			X	X				X	Kwame Nkrumah University of Science & Technology School of Medicine and The Bill & Melinda Gates Institute for Population and Reproductive Health at The Johns Hopkins Bloomberg School of Public Health. Performance Monitoring and Accountability 2020 (PMA2020) Survey round 2, PMA2014/Ghana-R2. 2014. Ghana and Baltimore, Maryland, USA.	256241
Ghana	2014	Ghana Performance Monitoring and Accountability 2020 Survey, Round 3 2014	Survey microdata	Admin 1			X	X				X	Kwame Nkrumah University of Science & Technology School of Medicine and The Bill & Melinda Gates Institute for Population and Reproductive Health at The Johns Hopkins Bloomberg School of Public Health. Performance Monitoring and Accountability 2020 (PMA2020) Survey round 3, PMA2014/Ghana-R3. 2014. Ghana and Baltimore, Maryland, USA.	256243
Ghana	2015	Ghana Performance Monitoring and Accountability 2020 Survey, Round 4 2015	Survey microdata	Admin 1			X	X				X	Bill and Melinda Gates Institute for Population and Reproductive Health, Johns Hopkins Bloomberg School of Public Health, Ghana Health Service, Ghana Statistical Service, Kwame Nkrumah University of Science and Technology (KNUST), University for Development Studies (Ghana). Ghana Performance Monitoring and Accountability 2020 Survey, Round 4 2015. Baltimore, United States of America: Bill and Melinda Gates Institute for Population and Reproductive Health, Johns Hopkins Bloomberg School of Public Health.	256244

Country	Years	Name	Type	Geographic Detail	Male Circumcision	STI Symptoms	Had Intercourse	Partner Away	Condom Last Time	Multiple Partners in Year (Males	Multiple Partners in Year (Females)	In Union	Citation	NID
Ghana	2016	Ghana Performance Monitoring and Accountability 2020 Survey, Round 5 2016	Survey microdata	Admin 1			X	X				X	Kwame Nkrumah University of Science & Technology School of Medicine and The Bill & Melinda Gates Institute for Population and Reproductive Health at The Johns Hopkins Bloomberg School of Public Health. Performance Monitoring and Accountability 2020 (PMA2020) Survey round 5, PMA2016/Ghana-R5. 2016. Ghana and Baltimore, Maryland, USA.	286146
Ghana	2017	Ghana Special Demographic and Health Survey 2017	Survey microdata	GPS				X					Ghana Health Service, Ghana Statistical Service, ICF International. Ghana Special Demographic and Health Survey 2017. Fairfax, United States of America: ICF International, 2018.	218572
Guinea	2005	Guinea Demographic and Health Survey 2005	Survey microdata	GPS	X	X	X	X	X	X	X	X	Macro International, Inc, National Statistics Directorate (Guinea). Guinea Demographic and Health Survey 2005. Fairfax, United States of America: ICF International.	19683

Country	Years	Name	Type	Geographic Detail	Male Circumcision	STI Symptoms	Had Intercourse	Partner Away	Condom Last Time	Multiple Partners in Year (Males	Multiple Partners in Year (Females)	In Union	Citation	NID
Guinea	2012	Guinea Demographic and Health Survey 2012	Survey microdata	GPS		X	X	X	X	X	X	X	ICF Macro, Ministry of Health and Public Hygiene (Guinea), National Institute of Statistics (Guinea). Guinea Demographic and Health Survey 2012. Fairfax, United States of America: ICF International.	69761
Guinea	2016	Guinea Multiple Indicator Cluster Survey 2016	Survey microdata	Admin 1			X				X		National Institute of Public Health (NPHI) (Guinea), National Institute of Statistics (Guinea), National Malaria Control Program (Guinea), United Nations Children's Fund (UNICEF). Guinea Multiple Indicator Cluster Survey 2016. New York, United States of America: United Nations Children's Fund (UNICEF), 2018.	303458
Guinea	2018	Guinea Demographic and Health Survey 2018	Survey microdata	GPS		X	X	X	X	X	X	X	Ministry of Health (Guinea), Ministry of Planning and Economic Development (Guinea), National Institute of Statistics (Guinea). Guinea Demographic and Health Survey 2018. Fairfax, United States of America: ICF International, 2019.	396957

Country	Years	Name	Type	Geographic Detail	Male Circumcision	STI Symptoms	Had Intercourse	Partner Away	Condom Last Time	Multiple Partners in Year (Males	Multiple Partners in Year (Females)	In Union	Citation	NID
Guinea-Bissau	2006	Guinea-Bissau Multiple Indicator Cluster Survey 2006	Survey microdata	Admin 1			X				X		United Nations Children's Fund (UNICEF), Government of Guinea-Bissau. Guinea-Bissau Multiple Indicator Cluster Survey 2006. New York, United States: United Nations Children's Fund (UNICEF).	4818
Guinea-Bissau	2014	Guinea-Bissau Multiple Indicator Cluster Survey 2014	Survey microdata	Admin 1	X		X		X	X	X	X	National Statistics Institute (Guinea-Bissau), United Nations Children's Fund (UNICEF). Guinea-Bissau Multiple Indicator Cluster Survey 2014. New York, United States of America: United Nations Children's Fund (UNICEF), 2016.	174049
Kenya	2000	Kenya Multiple Indicator Cluster Survey 2000	Survey microdata	GPS								X	Central Bureau of Statistics (Kenya), United Nations Children's Fund (UNICEF). Kenya Multiple Indicator Cluster Survey 2000. New York, United States: United Nations Children's Fund (UNICEF).	7387

Country	Years	Name	Type	Geographic Detail	Male Circumcision	STI Symptoms	Had Intercourse	Partner Away	Condom Last Time	Multiple Partners in Year (Males	Multiple Partners in Year (Females)	In Union	Citation	NID
Kenya	2003	Kenya Demographic and Health Survey 2003	Survey microdata	GPS	X	X	X	X	X	X	X	X	Centers for Disease Control and Prevention (CDC), Central Bureau of Statistics (Kenya), Macro International, Inc, Ministry of Health (Kenya), National Council for Population and Development (Kenya). Kenya Demographic and Health Survey 2003. Fairfax, United States of America: ICF International.	20145
Kenya	2007	Kenya AIDS Indicator Survey 2007	Survey microdata	GPS	X		X	X	X	X	X	X	Centers for Disease Control and Prevention (CDC), Kenya Medical Research Institute (KEMRI), Kenya National Bureau of Statistics, Ministry of Public Health and Sanitation (Kenya), National AIDS Control Council (Kenya), National AIDS and STI Control Programme (NASCOP) (Kenya), National Coordinating Agency for Population and Development (Kenya), National Public Health Laboratory Services, Ministry of Public Health and Sanitation (Kenya), United States Agency for International Development (USAID). Kenya AIDS Indicator Survey 2007. Nairobi, Kenya: Kenya National Bureau of Statistics.	133219
Kenya	2007	Kenya Household Health Expenditure and Utilization Survey 2007	Survey microdata	Admin 2								X	Abt Associates Inc., Kenya National Bureau of Statistics, Ministry of Health (Kenya). Kenya Household Health Expenditure and Utilization Survey 2007. Nairobi, Kenya: Kenya National Bureau of Statistics.	157635

Country	Years	Name	Type	Geographic Detail	Male Circumcision	STI Symptoms	Had Intercourse	Partner Away	Condom Last Time	Multiple Partners in Year (Males	Multiple Partners in Year (Females)	In Union	Citation	NID
Kenya	2008-09	Kenya Demographic and Health Survey 2008-2009	Survey microdata	GPS	X	X	X	X	X	X	X	X	ICF Macro, Kenya Medical Research Institute (KEMRI), Kenya National Bureau of Statistics, Ministry of Public Health and Sanitation (Kenya), National AIDS and STI Control Programme (NASCOP) (Kenya), National Aids Control Council (NACC), National Coordinating Agency for Population and Development (Kenya). Kenya Demographic and Health Survey 2008-2009. Fairfax, United States of America: ICF International.	21365
Kenya	2009	Kenya - Coast Multiple Indicator Cluster Survey 2009	Survey microdata	Admin 3			X				X		Kenya National Bureau of Statistics, United Nations Children's Fund (UNICEF). Kenya - Coast Multiple Indicator Cluster Survey 2009. New York, United States of America: United Nations Children's Fund (UNICEF), 2014.	56420
Kenya	2011	Kenya - Nyanza Province Multiple Indicator Cluster Survey 2011	Survey microdata	GPS			X				X		Kenya National Bureau of Statistics, United Nations Children's Fund (UNICEF). Kenya - Nyanza Province Multiple Indicator Cluster Survey 2011. Nairobi, Kenya: Kenya National Bureau of Statistics.	135416

Country	Years	Name	Type	Geographic Detail	Male Circumcision	STI Symptoms	Had Intercourse	Partner Away	Condom Last Time	Multiple Partners in Year (Males	Multiple Partners in Year (Females)	In Union	Citation	NID
Kenya	2013	Kenya AIDS Indicator Survey 2012-2013	Survey microdata	GPS	X	X	X	X		X	X	X	Kenya National Bureau of Statistics, Ministry of Devolution and Planning (Kenya), Ministry of Health (Kenya), National AIDS and STI Control Programme (NASCOP) (Kenya). Kenya AIDS Indicator Survey 2012-2013. Nairobi, Kenya: Kenya National Bureau of Statistics.	133304
Kenya	2013-14	Kenya - Bungoma County Multiple Indicator Survey 2013-2014	Survey microdata	GPS			X						Kenya National Bureau of Statistics, Population Studies and Research Institute, University of Nairobi (Kenya), United Nations Children's Fund (UNICEF). Kenya - Bungoma County Multiple Indicator Survey 2013-2014. New York, United States of America: United Nations Children's Fund (UNICEF), 2015.	203654
Kenya	2013-14	Kenya - Kakamega County Multiple Indicator Survey 2013-2014	Survey microdata	GPS			X				X		Kenya National Bureau of Statistics, Population Studies and Research Institute, University of Nairobi (Kenya), United Nations Children's Fund (UNICEF). Kenya - Kakamega County Multiple Indicator Survey 2013-2014. New York, United States of America: United Nations Children's Fund (UNICEF), 2015.	203663

Country	Years	Name	Type	Geographic Detail	Male Circumcision	STI Symptoms	Had Intercourse	Partner Away	Condom Last Time	Multiple Partners in Year (Males	Multiple Partners in Year (Females)	In Union	Citation	NID
Kenya	2013-14	Kenya - Turkana County Multiple Indicator Survey 2013-2014	Survey microdata	GPS			X				X		Kenya National Bureau of Statistics, Population Studies and Research Institute, University of Nairobi (Kenya), United Nations Children's Fund (UNICEF). Kenya - Turkana County Multiple Indicator Survey 2013-2014. New York, United States of America: United Nations Children's Fund (UNICEF), 2015.	203664
Kenya	2014	Kenya Demographic and Health Survey 2014	Survey microdata	GPS	X	X	X	X	X	X	X	X	ICF International, Kenya Medical Research Institute (KEMRI), Kenya National Bureau of Statistics, Ministry of Health (Kenya), National AIDS Control Council (Kenya), National Council for Population and Development (Kenya). Kenya Demographic and Health Survey 2014. Fairfax, United States of America: ICF International.	157057
Kenya	2014	Kenya Performance Monitoring and Accountability 2020 Survey, Round 1 2014	Survey microdata	Admin 2			X	X				X	International Centre for Reproductive Health Kenya (ICRHK) and The Bill & Melinda Gates Institute for Population and Reproductive Health at The Johns Hopkins Bloomberg School of Public Health. Performance Monitoring and Accountability 2020 (PMA2020) Survey round 1, PMA2014/Kenya-R1. 2014. Kenya and Baltimore, Maryland, USA.	197910

Country	Years	Name	Type	Geographic Detail	Male Circumcision	STI Symptoms	Had Intercourse	Partner Away	Condom Last Time	Multiple Partners in Year (Males	Multiple Partners in Year (Females)	In Union	Citation	NID
Kenya	2014	Kenya Performance Monitoring and Accountability 2020 Survey, Round 2 2014	Survey microdata	Admin 2			X	X				X	Bill and Melinda Gates Institute for Population and Reproductive Health, Johns Hopkins Bloomberg School of Public Health, International Center for Reproductive Health (Kenya), Kenya National Bureau of Statistics, Ministry of Health (Kenya), National Council for Population and Development (Kenya). Kenya Performance Monitoring and Accountability 2020 Survey, Round 2 2014. Baltimore, United States of America: Johns Hopkins Bloomberg School of Public Health.	256338
Kenya	2015	Kenya Performance Monitoring and Accountability 2020 Survey, Round 3 2015	Survey microdata	Admin 2			X	X				X	International Centre for Reproductive Health Kenya (ICRHK) and The Bill & Melinda Gates Institute for Population and Reproductive Health at The Johns Hopkins Bloomberg School of Public Health. Performance Monitoring and Accountability 2020 (PMA2020) Survey round 3, PMA2015/Kenya-R3. 2015. Kenya and Baltimore, Maryland, USA.	256365
Kenya	2015	Kenya Performance Monitoring and Accountability 2020 Survey, Round 4 2015	Survey microdata	Admin 2			X	X				X	International Centre for Reproductive Health Kenya (ICRHK) and The Bill & Melinda Gates Institute for Population and Reproductive Health at The Johns Hopkins Bloomberg School of Public Health. Performance Monitoring and Accountability 2020 (PMA2020) Survey round 4, PMA2015/Kenya-R4. 2015. Kenya and Baltimore, Maryland, USA.	256366

Country	Years	Name	Type	Geographic Detail	Male Circumcision	STI Symptoms	Had Intercourse	Partner Away	Condom Last Time	Multiple Partners in Year (Males	Multiple Partners in Year (Females)	In Union	Citation	NID
Kenya	2016	Kenya Performance Monitoring and Accountability 2020 Survey, Round 5 2016	Survey microdata	Admin 2			X	X				X	International Centre for Reproductive Health Kenya (ICRHK) and The Bill & Melinda Gates Institute for Population and Reproductive Health at The Johns Hopkins Bloomberg School of Public Health. Performance Monitoring and Accountability 2020 (PMA2020) Survey round 5, PMA2016/Kenya-R5. 2016. Kenya and Baltimore, Maryland, USA.	347047
Kenya	2017	Kenya Performance Monitoring and Accountability 2020 Survey, Round 6 2017	Survey microdata	Admin 1			X						International Centre for Reproductive Health Kenya (ICRHK) and The Bill & Melinda Gates Institute for Population and Reproductive Health at The Johns Hopkins Bloomberg School of Public Health. Performance Monitoring and Accountability 2020 (PMA2020) Survey round 5, PMA2016/Kenya-R5. 2016. Kenya and Baltimore, Maryland, USA.	374949
Kenya	2018	Kenya Performance Monitoring and Accountability 2020 Survey, Round 7 2018	Survey microdata	Admin 1			X					X	International Centre for Reproductive Health Kenya (ICRHK) and The Bill & Melinda Gates Institute for Population and Reproductive Health at The Johns Hopkins Bloomberg School of Public Health. Performance Monitoring and Accountability 2020 (PMA2020) Survey Round 7, PMA2018/Kenya-R7. 2018. Kenya and Baltimore, Maryland, USA.	407837

Country	Years	Name	Type	Geographic Detail	Male Circumcision	STI Symptoms	Had Intercourse	Partner Away	Condom Last Time	Multiple Partners in Year (Males	Multiple Partners in Year (Females)	In Union	Citation	NID
Lesotho	2000	Lesotho Multiple Indicator Cluster Survey 2000	Survey microdata	Admin 1								X	Bureau of Statistics (Lesotho), United Nations Children's Fund (UNICEF). Lesotho Multiple Indicator Cluster Survey 2000. New York, United States of America: United Nations Children's Fund (UNICEF).	7721
Lesotho	2004-05	Lesotho Demographic and Health Survey 2004-2005	Survey microdata	GPS	X	X	X	X		X	X	X	Bureau of Statistics (Lesotho), Macro International, Inc, Ministry of Health and Social Welfare (Lesotho). Lesotho Demographic and Health Survey 2004-2005. Fairfax, United States of America: ICF International.	20167
Lesotho	2009-10	Lesotho Demographic and Health Survey 2009-2010	Survey microdata	GPS	X	X	X	X	X	X	X	X	ICF Macro, Ministry of Health and Social Welfare (Lesotho). Lesotho Demographic and Health Survey 2009-2010. Fairfax, United States of America: ICF International.	21382

Country	Years	Name	Type	Geographic Detail	Male Circumcision	STI Symptoms	Had Intercourse	Partner Away	Condom Last Time	Multiple Partners in Year (Males	Multiple Partners in Year (Females)	In Union	Citation	NID
Lesotho	2014	Lesotho Demographic and Health Survey 2014	Survey microdata	GPS	X	X	X	X	X	X	X	X	ICF International, Ministry of Health and Social Welfare (Lesotho). Lesotho Demographic and Health Survey 2014. Fairfax, United States of America: ICF International.	157058
Lesotho	2018	Lesotho Multiple Indicator Cluster Survey 2018	Survey microdata	Admin 1								X	Bureau of Statistics (Lesotho), United Nations Children's Fund (UNICEF). Lesotho Multiple Indicator Cluster Survey 2018. New York, United States of America: United Nations Children's Fund (UNICEF), 2019.	427778
Liberia	2006-07	Liberia Demographic and Health Survey 2006-2007	Survey microdata	GPS	X	X	X	X	X	X	X	X	Liberia Institute for Statistics and Geo-information Services (LISGIS), Macro International, Inc. Liberia Demographic and Health Survey 2006-2007. Fairfax, United States of America: ICF International.	20191

Country	Years	Name	Type	Geographic Detail	Male Circumcision	STI Symptoms	Had Intercourse	Partner Away	Condom Last Time	Multiple Partners in Year (Males	Multiple Partners in Year (Females)	In Union	Citation	NID
Liberia	2013	Liberia Demographic and Health Survey 2013	Survey microdata	GPS	X	X	X	X	X	X	X	X	ICF International, Liberia Institute for Statistics and Geo-information Services (LISGIS), National AIDS and STI Control Program (NACP), Ministry of Health and Social Welfare (Liberia). Liberia Demographic and Health Survey 2013. Fairfax, United States of America: ICF International.	77385
Madagascar	2000	Madagascar Multiple Indicator Cluster Survey 2000	Survey microdata	Admin 1			X						National Institute of Statistics (Madagascar), United Nations Children's Fund (UNICEF). Madagascar Multiple Indicator Cluster Survey 2000. New York, United States of America: United Nations Children's Fund (UNICEF).	27020
Madagascar	2003-04	Madagascar Demographic and Health Survey 2003-2004	Survey microdata	Admin 2	X	X	X	X	X	X	X	X	Macro International, Inc, National Institute of Statistics (Madagascar). Madagascar Demographic and Health Survey 2003-2004. Fairfax, United States of America: ICF International.	20223

Country	Years	Name	Type	Geographic Detail	Male Circumcision	STI Symptoms	Had Intercourse	Partner Away	Condom Last Time	Multiple Partners in Year (Males	Multiple Partners in Year (Females)	In Union	Citation	NID
Madagascar	2008-09	Madagascar Demographic and Health Survey 2008-2009	Survey microdata	GPS	X	X	X	X	X	X	X	X	ICF Macro, National Institute of Statistics (Madagascar). Madagascar Demographic and Health Survey 2008-2009. Fairfax, United States of America: ICF International.	21409
Madagascar	2012	Madagascar - South Multiple Indicator Cluster Survey 2012	Survey microdata	GPS			X				X		National Institute of Statistics (Madagascar), United Nations Children's Fund (UNICEF). Madagascar - South Multiple Indicator Cluster Survey 2012. New York, United States of America: United Nations Children's Fund (UNICEF), 2015.	125594
Madagascar	2018	Madagascar Multiple Indicator Cluster Survey 2018	Survey microdata	GPS			X		X			X	National Institute of Statistics (Madagascar), United Nations Children's Fund (UNICEF). Madagascar Multiple Indicator Cluster Survey 2018. 2019.	399853

Country	Years	Name	Type	Geographic Detail	Male Circumcision	STI Symptoms	Had Intercourse	Partner Away	Condom Last Time	Multiple Partners in Year (Males	Multiple Partners in Year (Females)	In Union	Citation	NID
Malawi	2000	Malawi Demographic and Health Survey 2000	Survey microdata	GPS		X	X	X	X	X	X	X	Macro International, Inc, National Statistical Office of Malawi. Malawi Demographic and Health Survey 2000. Fairfax, United States of America: ICF International.	20252
Malawi	2004-05	Malawi Demographic and Health Survey 2004-2005	Survey microdata	GPS	X	X	X	X	X	X	X	X	Macro International, Inc, National Statistical Office of Malawi. Malawi Demographic and Health Survey 2004-2005. Fairfax, United States of America: ICF International.	20263
Malawi	2006	Malawi Multiple Indicator Cluster Survey 2006	Survey microdata	Admin 2			X		X	X	X	X	United Nations Children's Fund (UNICEF), National Statistics Office (Malawi). Malawi Multiple Indicator Cluster Survey 2006. New York, United States: United Nations Children's Fund (UNICEF).	7919

Country	Years	Name	Type	Geographic Detail	Male Circumcision	STI Symptoms	Had Intercourse	Partner Away	Condom Last Time	Multiple Partners in Year (Males	Multiple Partners in Year (Females)	In Union	Citation	NID
Malawi	2010	Malawi Demographic and Health Survey 2010	Survey microdata	GPS	X	X	X	X	X	X	X	X	ICF Macro, National Statistical Office of Malawi. Malawi Demographic and Health Survey 2010. Fairfax, United States of America: ICF International.	21393
Malawi	2013-14	Malawi Multiple Indicator Cluster Survey 2013-2014	Survey microdata	Admin 2	X		X		X	X	X	X	National Statistical Office of Malawi, United Nations Children's Fund (UNICEF). Malawi Multiple Indicator Cluster Survey 2013-2014. New York, United States of America: United Nations Children's Fund (UNICEF), 2015.	161662
Malawi	2015-16	Malawi Demographic and Health Survey 2015-2016	Survey microdata	GPS	X	X	X	X	X	X	X	X	Emory University and Centers for Disease Control & Prevention Collaboration, ICF International, Ministry of Health (Malawi), National Statistical Office of Malawi. Malawi Demographic and Health Survey 2015-2016. Fairfax, United States of America: ICF International, 2017.	218581

Country	Years	Name	Type	Geographic Detail	Male Circumcision	STI Symptoms	Had Intercourse	Partner Away	Condom Last Time	Multiple Partners in Year (Males	Multiple Partners in Year (Females)	In Union	Citation	NID
Malawi	2015-16	Malawi Population-based HIV Impact Assessment 2015-2016	Survey microdata	Admin >1	X	X	X	X	X	X	X	X	Ministry of Health, Malawi, Centers for Disease Control and Prevention (CDC), and ICAP at Columbia University. Malawi Population-based HIV Impact Assessment (MPHIA) 2015-16: First Report. Lilongwe, Malawi, Atlanta, Georgia and New York, New York, USA: Ministry of Health, CDC and ICAP. December 2016.	287629
Malawi	2016-17	Malawi Integrated Household Survey 2016-2017	Survey microdata	Admin 2								X	National Statistical Office. Malawi - Fourth Integrated Household Survey 2016-2017, Ref. MWI_2016_IHS-IV_v02_M. Dataset downloaded from [http://microdata.worldbank.org/index.php/catalog/2936] on [December 18, 2017].	327852
Mali	2001	Mali Demographic and Health Survey 2001	Survey microdata	GPS		X	X	X	X	X	X	X	Macro International, Inc, National Directorate of Statistics and Informatics (DNSI) (Mali), Planning and Statistics Unit, Ministry of Health (Mali). Mali Demographic and Health Survey 2001. Fairfax, United States of America: ICF International.	20315

Country	Years	Name	Type	Geographic Detail	Male Circumcision	STI Symptoms	Had Intercourse	Partner Away	Condom Last Time	Multiple Partners in Year (Males	Multiple Partners in Year (Females)	In Union	Citation	NID
Mali	2006	Mali Demographic and Health Survey 2006	Survey microdata	GPS	X	X	X	X	X	X	X	X	Macro International, Inc, Ministry of Health (Mali), National Directorate of Statistics and Informatics (DNSI) (Mali). Mali Demographic and Health Survey 2006. Fairfax, United States of America: ICF International.	20274
Mali	2009-10	Mali Multiple Indicator Cluster Survey 2009-2010	Survey microdata	Admin 2			X				X		Ministry of Health (Mali), National Institute of Statistics (INSTAT) (Mali), United Nations Children's Fund (UNICEF). Mali Multiple Indicator Cluster Survey 2009-2010. New York, United States of America: United Nations Children's Fund (UNICEF), 2017.	270627
Mali	2012-13	Mali Demographic and Health Survey 2012-2013	Survey microdata	GPS	X	X	X	X	X	X	X	X	ICF International, INFO-STAT (Mali), Ministry of Health (Mali), National Institute of Statistics (INSTAT) (Mali), Planning and Statistics Unit, Ministry of Health (Mali). Mali Demographic and Health Survey 2012-2013. Fairfax, United States of America: ICF International, 2014.	77388

Country	Years	Name	Type	Geographic Detail	Male Circumcision	STI Symptoms	Had Intercourse	Partner Away	Condom Last Time	Multiple Partners in Year (Males	Multiple Partners in Year (Females)	In Union	Citation	NID
Mali	2015	Mali Malaria Indicator Survey 2015	Survey microdata	GPS								X	ICF International, INFO-STAT (Mali), Ministry of Health and Public Hygiene (Mali), National Institute of Public Health Research (INRSP) (Mali), National Institute of Statistics (INSTAT) (Mali), National Program for the Fight Against Malaria (Mali). Mali Malaria Indicator Survey 2015. Fairfax, United States of America: ICF International, 2016.	218587
Mali	2015	Mali Multiple Indicator Cluster Survey 2015	Survey microdata	Admin 1			X		X	X	X	X	Ministry of Health (Mali), Ministry of Planning (Mali), National Institute of Statistics (INSTAT) (Mali), United Nations Children's Fund (UNICEF). Mali Multiple Indicator Cluster Survey 2015. New York, United States of America: United Nations Children's Fund (UNICEF), 2017.	248224
Mali	2018	Mali Demographic and Health Survey 2018	Survey microdata	GPS	X	X	X	X	X	X	X	X	ICF International, National Institute of Statistics (INSTAT) (Mali). Mali Demographic and Health Survey 2018. Fairfax, United States of America: ICF International, 2019.	398033

Country	Years	Name	Type	Geographic Detail	Male Circumcision	STI Symptoms	Had Intercourse	Partner Away	Condom Last Time	Multiple Partners in Year (Males	Multiple Partners in Year (Females)	In Union	Citation	NID
Mauritania	2000-01	Mauritania Demographic and Health Survey 2000-2001	Survey microdata	Admin 1			X	X		X		X	Macro International, Inc, National Office of Statistics (Mauritania). Mauritania Demographic and Health Survey 2000-2001. Fairfax, United States of America: ICF International.	20322
Mauritania	2011	Mauritania Multiple Indicator Cluster Survey 2011	Survey microdata	Admin 3								X	National Office of Statistics (Mauritania), United Nations Children's Fund (UNICEF). Mauritania Multiple Indicator Cluster Survey 2011. New York, United States of America: United Nations Children's Fund (UNICEF), 2015.	152783
Mauritania	2015	Mauritania Multiple Indicator Cluster Survey 2015	Survey microdata	Admin 1								X	National Office of Statistics (Mauritania), United Nations Children's Fund (UNICEF). Mauritania Multiple Indicator Cluster Survey 2015. New York, United States of America: United Nations Children's Fund (UNICEF), 2018.	267343

Country	Years	Name	Type	Geographic Detail	Male Circumcision	STI Symptoms	Had Intercourse	Partner Away	Condom Last Time	Multiple Partners in Year (Males	Multiple Partners in Year (Females)	In Union	Citation	NID
Mozambique	2001	Mozambique Young Adult Reproductive Health Survey 2001	Survey microdata	Admin 1			X						Mozambique National Institute of Statistics, Division of Reproductive Health-Centers for Disease Control and Prevention (CDC). (2003) Mozambique Young Adult Reproductive Health Survey 2001. Atlanta, United States: Centers for Disease Control and Prevention (CDC).	27519
Mozambique	2003	Mozambique Demographic and Health Survey 2003-2004	Survey microdata	Admin 1	X	X	X	X	X	X	X	X	Macro International, Inc, National Institute of Statistics (INE) (Mozambique). Mozambique Demographic and Health Survey 2003-2004. Fairfax, United States of America: ICF International.	20394
Mozambique	2008	Mozambique Multiple Indicator Cluster Survey 2008-2009	Survey microdata	Admin 5, GPS			X						United Nations Children's Fund (UNICEF), National Statistics Institute (Mozambique). Mozambique Multiple Indicator Cluster Survey 2008-2009. New York, United States: United Nations Children's Fund (UNICEF).	27031

Country	Years	Name	Type	Geographic Detail	Male Circumcision	STI Symptoms	Had Intercourse	Partner Away	Condom Last Time	Multiple Partners in Year (Males	Multiple Partners in Year (Females)	In Union	Citation	NID
Mozambique	2009	Mozambique AIDS Indicator Survey 2009	Survey microdata	GPS	X	X	X	X	X	X	X	X	ICF Macro, Ministry of Health (Mozambique), National Institute of Statistics (INE) (Mozambique). Mozambique AIDS Indicator Survey 2009. Fairfax, United States of America: ICF International, 2010.	8906
Mozambique	2011	Mozambique Demographic and Health Survey 2011	Survey microdata	GPS	X	X	X	X	X	X	X	X	ICF Macro, Manhica Health Research Center (CISM), Ministry of Health (Mozambique), National Institute of Statistics (INE) (Mozambique). Mozambique Demographic and Health Survey 2011. Fairfax, United States of America: ICF International.	55975
Mozambique	2015	Mozambique AIDS Indicator Survey 2015	Survey microdata	GPS	X	X	X	X	X	X	X	X	Centers for Disease Control and Prevention (CDC), ICF International, Ministry of Health (Mozambique), National Institute of Health (Mozambique), National Institute of Statistics (INE) (Mozambique). Mozambique AIDS Indicator Survey 2015. Fairfax, United States of America: ICF International, 2018.	157060

Country	Years	Name	Type	Geographic Detail	Male Circumcision	STI Symptoms	Had Intercourse	Partner Away	Condom Last Time	Multiple Partners in Year (Males	Multiple Partners in Year (Females)	In Union	Citation	NID
Namibia	2000	Namibia Demographic and Health Survey 2000	Survey microdata	GPS			X	X	X	X	X	X	Macro International, Inc, Ministry of Health and Social Services (Namibia), National Planning Commission (Namibia). Namibia Demographic and Health Survey 2000. Fairfax, United States of America: ICF International.	20417
Namibia	2006-07	Namibia Demographic and Health Survey 2006-2007	Survey microdata	GPS	X	X	X	X	X	X	X	X	Macro International, Inc, Ministry of Health and Social Services (Namibia). Namibia Demographic and Health Survey 2006-2007. Fairfax, United States of America: ICF International.	20428
Namibia	2013	Namibia Demographic and Health Survey 2013	Survey microdata	GPS	X	X	X	X	X	X	X	X	ICF International, Ministry of Health and Social Services (Namibia), Namibia Institute of Pathology, Namibia Statistics Agency. Namibia Demographic and Health Survey 2013. Fairfax, United States of America: ICF International.	150382

Country	Years	Name	Type	Geographic Detail	Male Circumcision	STI Symptoms	Had Intercourse	Partner Away	Condom Last Time	Multiple Partners in Year (Males	Multiple Partners in Year (Females)	In Union	Citation	NID
Niger	2000	Niger Multiple Indicator Cluster Survey 2000	Survey microdata	Admin 1								X	Government of Niger, Macro International, Inc, United Nations Children's Fund (UNICEF). Niger Multiple Indicator Cluster Survey 2000. New York, United States of America: United Nations Children's Fund (UNICEF).	9439
Niger	2006	Niger Demographic and Health Survey 2006	Survey microdata	Admin 1	X	X	X	X	X	X	X	X	Department of Statistics and National Accounts (Niger), Macro International, Inc. Niger Demographic and Health Survey 2006. Fairfax, United States of America: ICF International.	20499
Niger	2012	Niger Demographic and Health Survey 2012	Survey microdata	Admin 1		X	X	X	X	X	X	X	ICF International, Ministry of Public Health (Niger), National Institute of Statistics (Niger). Niger Demographic and Health Survey 2012. Fairfax, United States of America: ICF International.	74393

Country	Years	Name	Type	Geographic Detail	Male Circumcision	STI Symptoms	Had Intercourse	Partner Away	Condom Last Time	Multiple Partners in Year (Males	Multiple Partners in Year (Females)	In Union	Citation	NID
Niger	2015	Niger - Niamey Performance Monitoring and Accountability 2020 Survey, Round 1 2015	Survey microdata	Admin 1			X	X				X	Niger/Niamey Institut National de la Statistique (National Institute of Statistics) and The Bill & Melinda Gates Institute for Population and Reproductive Health at The Johns Hopkins Bloomberg School of Public Health. Performance Monitoring and Accountability 2020 (PMA2020) Survey round 1, PMA2015/Niger-R1 (Niamey). 2015. Niamey, Niger and Baltimore, Maryland, USA.	256177
Niger	2016	Niger Performance Monitoring and Accountability 2020 Survey, Round 2 2016	Survey microdata	Admin 1			X	X				X	Niger/Niamey Institut National de la Statistique (National Institute of Statistics) and The Bill & Melinda Gates Institute for Population and Reproductive Health at The Johns Hopkins Bloomberg School of Public Health. Performance Monitoring and Accountability 2020 (PMA2020) Survey round 2, PMA2016/Niger-R2 (National). 2016. Niamey, Niger and Baltimore, Maryland, USA.	286052
Niger	2018	Niger - Niamey Performance Monitoring and Accountability 2020 Survey, Round 5 2018	Survey microdata	GPS			X					X	Niger/Niamey Institut National de la Statistique (National Institute of Statistics) and The Bill & Melinda Gates Institute for Population and Reproductive Health at The Johns Hopkins Bloomberg School of Public Health. Performance Monitoring and Accountability 2020 (PMA2020) Survey round 5, PMA2018/Niger-R5 (Niamey). 2018. Niamey, Niger and Baltimore, Maryland, USA.	398719

Country	Years	Name	Type	Geographic Detail	Male Circumcision	STI Symptoms	Had Intercourse	Partner Away	Condom Last Time	Multiple Partners in Year (Males	Multiple Partners in Year (Females)	In Union	Citation	NID
Nigeria	2003	Nigeria Demographic and Health Survey 2003	Survey microdata	GPS		X	X	X	X	X	X	X	Department for International Development (DFiD) (United Kingdom), National Population Commission of Nigeria, ORC Macro, United Nations Children's Fund (UNICEF), United Nations Population Fund (UNFPA). Nigeria Demographic and Health Survey 2003. Fairfax, United States of America: ICF International.	20567
Nigeria	2004	Nigeria Living Standards Survey 2003-2004	Survey microdata	Admin 1								X	Federal Office of Statistics (Nigeria). Nigeria Living Standards Survey 2003-2004.	25006
Nigeria	2006	Nigeria Core Welfare Indicators Questionnaire Survey 2006	Survey microdata	Admin 1	X							X	National Bureau of Statistics (Nigeria). Nigeria Core Welfare Indicators Questionnaire Survey 2006. Abuja, Nigeria: National Bureau of Statistics (Nigeria).	9522

Country	Years	Name	Type	Geographic Detail	Male Circumcision	STI Symptoms	Had Intercourse	Partner Away	Condom Last Time	Multiple Partners in Year (Males	Multiple Partners in Year (Females)	In Union	Citation	NID
Nigeria	2007	Nigeria Multiple Indicator Cluster Survey 2007	Survey microdata	Admin 1			X				X		United Nations Children's Fund (UNICEF), National Bureau of Statistics (Nigeria). Nigeria Multiple Indicator Cluster Survey 2007. New York, United States: United Nations Children's Fund (UNICEF).	9516
Nigeria	2007	Nigeria National HIV/AIDS and Reproductive Health Survey 2007	Survey microdata	Admin 1		X	X			X	X	X	Federal Ministry of Health (Nigeria), National Agency for the Control of AIDS (NACA) (Nigeria), National Bureau of Statistics (Nigeria), National Population Commission (NPC), Nigerian Institute of Medical Research, Society for Family Health (Nigeria), University College Hospital, Ibadan. Nigeria National HIV/AIDS and Reproductive Health Survey 2007.	325046
Nigeria	2008	Nigeria Demographic and Health Survey 2008	Survey microdata	GPS	X	X	X	X	X	X	X	X	Macro International, Inc, National Population Commission of Nigeria. Nigeria Demographic and Health Survey 2008. Fairfax, United States of America: ICF International, 2009.	21433

Country	Years	Name	Type	Geographic Detail	Male Circumcision	STI Symptoms	Had Intercourse	Partner Away	Condom Last Time	Multiple Partners in Year (Males	Multiple Partners in Year (Females)	In Union	Citation	NID
Nigeria	2008	Nigeria General Household Survey 2008 - IPUMS	Survey microdata	Admin 1								X	National Bureau of Statistics (Nigeria), Minnesota Population Center. Nigeria General Household Survey 2008 from the Integrated Public Use Microdata Series, International: [Machine-readable database]. Minneapolis: University of Minnesota.	151313
Nigeria	2009	Nigeria General Household Survey 2009 - IPUMS	Survey microdata	Admin 1								X	National Bureau of Statistics (Nigeria), Minnesota Population Center. Nigeria General Household Survey 2009 from the Integrated Public Use Microdata Series, International: [Machine-readable database]. Minneapolis: University of Minnesota.	151314
Nigeria	2009	Nigeria Living Standards Survey 2008-2010	Survey microdata	Admin 1, Admin 2								X	National Bureau of Statistics (Nigeria). Nigeria Living Standards Survey 2008-2010. Abuja, Nigeria: National Bureau of Statistics (Nigeria).	151719

Country	Years	Name	Type	Geographic Detail	Male Circumcision	STI Symptoms	Had Intercourse	Partner Away	Condom Last Time	Multiple Partners in Year (Males	Multiple Partners in Year (Females)	In Union	Citation	NID
Nigeria	2010	Nigeria General Household Survey 2010 - IPUMS	Survey microdata	Admin 2								X	National Bureau of Statistics (Nigeria), Minnesota Population Center. Nigeria General Household Survey 2010 from the Integrated Public Use Microdata Series, International: [Machine-readable database]. Minneapolis: University of Minnesota.	151315
Nigeria	2011	Nigeria General Household Survey 2010-2011 - IPUMS	Survey microdata	Admin 1								X	National Bureau of Statistics (Nigeria), Minnesota Population Center. Nigeria General Household Survey 2010-2011 from the Integrated Public Use Microdata Series, International: [Machine-readable database]. Minneapolis: University of Minnesota.	151317
Nigeria	2011	Nigeria Multiple Indicator Cluster Survey 2011	Survey microdata	Admin 1			X				X		National Bureau of Statistics (Nigeria), United Nations Children's Fund (UNICEF). Nigeria Multiple Indicator Cluster Survey 2011. New York, United States of America: United Nations Children's Fund (UNICEF), 2013.	76703

Country	Years	Name	Type	Geographic Detail	Male Circumcision	STI Symptoms	Had Intercourse	Partner Away	Condom Last Time	Multiple Partners in Year (Males	Multiple Partners in Year (Females)	In Union	Citation	NID
Nigeria	2012	Nigeria National HIV/AIDS and Reproductive Health Survey 2012	Survey microdata	Admin 2		X	X			X	X	X	Expanded Social Marketing Project in Nigeria (ESMPIN), Federal Ministry of Health (Nigeria), Joint United Nations Program on HIV/AIDS (UNAIDS), National Population Commission (NPC), Society for Family Health (Nigeria), University College Hospital, Ibadan, World Health Organization (WHO). Nigeria National HIV/AIDS and Reproductive Health Survey 2012.	324443
Nigeria	2013	Nigeria Demographic and Health Survey 2013	Survey microdata	GPS	X	X	X	X	X	X	X	X	ICF International, National Population Commission of Nigeria. Nigeria Demographic and Health Survey 2013. Fairfax, United States of America: ICF International.	77390
Nigeria	2014	Nigeria - Kaduna and Lagos Performance Monitoring and Accountability 2020 Survey, Round 1 2014	Survey microdata	Admin 1			X	X				X	Centre for Population and Reproductive Health (CPRH), University of Ibadan; Centre for Research, Evaluation Resources and Development (CRERD); Population and Reproductive Health Program (PRHP), Obafemi Awolowo University (OAU); Bayero University Kano (BUK); and The Bill & Melinda Gates Institute for Population and Reproductive Health at The Johns Hopkins Bloomberg School of Public Health. Performance Monitoring and Accountability 2020 (PMA2020) Survey round 1, PMA2014/Nigeria-R1 (Kaduna & Lagos). 2014. Nigeria and Baltimore, Maryland, USA.	256263

Country	Years	Name	Type	Geographic Detail	Male Circumcision	STI Symptoms	Had Intercourse	Partner Away	Condom Last Time	Multiple Partners in Year (Males	Multiple Partners in Year (Females)	In Union	Citation	NID
Nigeria	2015	Nigeria - Kaduna and Lagos Performance Monitoring and Accountability 2020 Survey, Round 2 2015	Survey microdata	Admin 1			X	X				X	Centre for Research, Evaluation Resources and Development (CRERD), Bayero University Kano (BUK), and The Bill & Melinda Gates Institute for Population and Reproductive Health at The Johns Hopkins Bloomberg School of Public Health. Performance Monitoring and Accountability 2020 (PMA2020) Survey round 2, PMA2015/Nigeria-R2 (Kaduna & Lagos). 2015. Nigeria and Baltimore, Maryland, USA	256268
Nigeria	2016	Nigeria Performance Monitoring and Accountability 2020 Survey, Round 3 2016	Survey microdata	Admin 1			X					X	Centre for Research, Evaluation Resources and Development (CRERD), Bayero University Kano (BUK), and The Bill & Melinda Gates Institute for Population and Reproductive Health at The Johns Hopkins Bloomberg School of Public Health. Performance Monitoring and Accountability 2020 (PMA2020) Survey round 3, PMA2016/Nigeria-R3 (National). 2016. Nigeria and Baltimore, Maryland, USA.	286022
Nigeria	2016-17	Nigeria Multiple Indicator Cluster Survey with National Immunization Coverage Survey Supplement 2016-2017	Survey microdata	GPS	X		X		X	X	X	X	National Agency for the Control of AIDS (Nigeria), National Bureau of Statistics (Nigeria), National Primary Health Care Development Agency (NPHCDA) (Nigeria), United Nations Children's Fund (UNICEF). Nigeria Multiple Indicator Cluster Survey with National Immunization Coverage Survey Supplement 2016-2017. New York, United States of America: United Nations Children's Fund (UNICEF), 2018.	218613

Country	Years	Name	Type	Geographic Detail	Male Circumcision	STI Symptoms	Had Intercourse	Partner Away	Condom Last Time	Multiple Partners in Year (Males	Multiple Partners in Year (Females)	In Union	Citation	NID
Nigeria	2018	Nigeria Demographic and Health Survey 2018	Survey microdata	GPS	X	X	X	X	X	X	X	X	Federal Ministry of Health (Nigeria), ICF International, National Population Commission (NPC). Nigeria Demographic and Health Survey 2018. Fairfax, United States of America: ICF International, 2019.	408484
Nigeria	2018	Nigeria Performance Monitoring and Accountability 2020 Survey, Round 5 2018	Survey microdata	Admin 1			X					X	Centre for Research, Evaluation Resources and Development (CRERD), Bayero University Kano (BUK), and The Bill & Melinda Gates Institute for Population and Reproductive Health at The Johns Hopkins Bloomberg School of Public Health. Performance Monitoring and Accountability 2020 (PMA2020) Survey round 5, PMA2018/Nigeria-R5 (National). 2018. Nigeria and Baltimore, Maryland, USA.	394679
Rwanda	2000	Rwanda Demographic and Health Survey 2000	Survey microdata	Admin 1		X	X		X	X	X	X	Macro International, Inc, National Office of Population (Rwanda). Rwanda Demographic and Health Survey 2000. Fairfax, United States of America: ICF International.	20722

Country	Years	Name	Type	Geographic Detail	Male Circumcision	STI Symptoms	Had Intercourse	Partner Away	Condom Last Time	Multiple Partners in Year (Males	Multiple Partners in Year (Females)	In Union	Citation	NID
Rwanda	2000	Rwanda Multiple Indicator Cluster Survey 2000	Survey microdata	Admin 1								X	Department of Statistics (Rwanda), United Nations Children's Fund (UNICEF). Rwanda Multiple Indicator Cluster Survey 2000. New York, United States: United Nations Children's Fund (UNICEF).	26930
Rwanda	2005	Rwanda Demographic and Health Survey 2005	Survey microdata	GPS	X	X	X		X	X	X	X	Macro International, Inc, National Institute of Statistics of Rwanda. Rwanda Demographic and Health Survey 2005. Fairfax, United States of America: ICF International.	20740
Rwanda	2007-08	Rwanda Interim Demographic and Health Survey 2007-2008	Survey microdata	GPS	X							X	Macro International, Inc, Ministry of Health (Rwanda), National Institute of Statistics of Rwanda. Rwanda Interim Demographic and Health Survey 2007-2008. Fairfax, United States of America: ICF International.	21222

Country	Years	Name	Type	Geographic Detail	Male Circumcision	STI Symptoms	Had Intercourse	Partner Away	Condom Last Time	Multiple Partners in Year (Males	Multiple Partners in Year (Females)	In Union	Citation	NID
Rwanda	2010-11	Rwanda Demographic and Health Survey 2010-2011	Survey microdata	GPS	X	X	X		X	X	X	X	ICF Macro, Ministry of Health (Rwanda), National Institute of Statistics of Rwanda. Rwanda Demographic and Health Survey 2010-2011. Fairfax, United States of America: ICF International.	56040
Rwanda	2011	Rwanda Integrated Household Living Conditions Survey 2010-2011	Survey microdata	Admin 1								X	National Institute of Statistics of Rwanda. Rwanda Integrated Household Living Conditions Survey 2010-2011. Kigali, Rwanda: National Institute of Statistics of Rwanda.	151437
Rwanda	2011	Rwanda Special Demographic and Health Survey 2011	Survey microdata	Admin 1								X	ICF International, Joint United Nations Program on HIV/AIDS (UNAIDS), National Institute of Statistics of Rwanda, Rwanda Biomedical Center/Institute of HIV/AIDS, Disease Control and Prevention Department, School of Public Health, University of Rwanda. Rwanda Special Demographic and Health Survey 2011. Fairfax, United States of America: ICF International, 2012.	56426

Country	Years	Name	Type	Geographic Detail	Male Circumcision	STI Symptoms	Had Intercourse	Partner Away	Condom Last Time	Multiple Partners in Year (Males	Multiple Partners in Year (Females)	In Union	Citation	NID
Rwanda	2014-15	Rwanda Demographic and Health Survey 2014-2015	Survey microdata	GPS	X	X	X		X	X	X	X	ICF International, Ministry of Health (Rwanda), National Institute of Statistics of Rwanda. Rwanda Demographic and Health Survey 2014-2015. Fairfax, United States of America: ICF International, 2016.	157063
Rwanda	2017	Rwanda Malaria Indicator Survey 2017	Survey microdata	Admin 2								X	ICF International, Ministry of Health (Rwanda), Rwanda Biomedical Center (RBC). Rwanda Malaria Indicator Survey 2017. Fairfax, United States of America: ICF International, 2018.	350836
Sao Tome and Principe	2000	Sao Tome and Principe Multiple Indicator Cluster Survey 2000	Survey microdata	Admin 1								X	National Institute of Statistics (Sao Tome and Principe), United Nations Children's Fund (UNICEF). Sao Tome and Principe Multiple Indicator Cluster Survey 2000. New York, United States of America: United Nations Children's Fund (UNICEF).	27055

Country	Years	Name	Type	Geographic Detail	Male Circumcision	STI Symptoms	Had Intercourse	Partner Away	Condom Last Time	Multiple Partners in Year (Males	Multiple Partners in Year (Females)	In Union	Citation	NID
Sao Tome and Principe	2008	Sao Tome and Principe Demographic and Health Survey 2008-2009	Survey microdata	Admin 2		X	X	X	X	X	X	X	ICF Macro, Ministry of Health (Sao Tome and Principe), National Institute of Statistics (Sao Tome and Principe). Sao Tome and Principe Demographic and Health Survey 2008-2009. Fairfax, United States of America: ICF International.	26866
Sao Tome and Principe	2014	Sao Tome and Principe Multiple Indicator Cluster Survey 2014	Survey microdata	Admin 2	X		X		X	X	X	X	Global Fund to Fight Aids Tuberculosis and Malaria (GFATM), ICF International, National Center for Endemic Diseases (CNE) (Sao Tome and Principe), National Institute of Statistics (Sao Tome and Principe), United Nations Children's Fund (UNICEF), United Nations Development Programme (UNDP). Sao Tome and Principe Multiple Indicator Cluster Survey 2014. New York, United States of America: United Nations Children's Fund (UNICEF), 2016.	214640
Senegal	2000	Senegal Multiple Indicator Cluster Survey 2000	Survey microdata	Admin 1								X	Directorate of Forecasting and Statistics, Ministry of the Economy, Finance and Planning (Senegal), Ministry of Economy and Finance (Senegal), United Nations Children's Fund (UNICEF). Senegal Multiple Indicator Cluster Survey 2000. New York, United States of America: United Nations Children's Fund (UNICEF).	27044

Country	Years	Name	Type	Geographic Detail	Male Circumcision	STI Symptoms	Had Intercourse	Partner Away	Condom Last Time	Multiple Partners in Year (Males	Multiple Partners in Year (Females)	In Union	Citation	NID
Senegal	2005	Senegal Demographic and Health Survey 2005	Survey microdata	GPS	X	X	X	X	X	X	X	X	Ministry of Health and Prevention (Senegal), Research Center for Human Development (Senegal). Senegal Demographic and Health Survey 2005. Fairfax, United States of America: ICF International.	26855
Senegal	2010-11	Senegal Demographic and Health Survey 2010-2011	Survey microdata	GPS		X	X	X	X	X	X	X	Center for Research in Human Development (CRDH), Cheikh Anta Diop University, Hospital Aristide Le Dantec, ICF Macro, National Agency of Statistics and Demography (Senegal). Senegal Demographic and Health Survey 2010-2011. Fairfax, United States of America: ICF International.	56063
Senegal	2012-13	Senegal Continuous Demographic and Health Survey 2012-2013	Survey microdata	GPS			X	X			X	X	ICF International, Ministry of Health and Social Action (Senegal), National Agency of Statistics and Demography (Senegal). Senegal Continuous Demographic and Health Survey 2012-2013. Fairfax, United States of America: ICF International.	111432

Country	Years	Name	Type	Geographic Detail	Male Circumcision	STI Symptoms	Had Intercourse	Partner Away	Condom Last Time	Multiple Partners in Year (Males	Multiple Partners in Year (Females)	In Union	Citation	NID
Senegal	2014	Senegal Continuous Demographic and Health Survey 2014	Survey microdata	GPS		X	X	X	X	X	X	X	Cheikh Anta Diop University, ICF International, National Agency of Statistics and Demography (Senegal). Senegal Continuous Demographic and Health Survey 2014. Fairfax, United States of America: ICF International.	191270
Senegal	2015	Senegal - Dakar Urban Multiple Indicator Cluster Survey 2015-2016	Survey microdata	Admin 2			X		X	X	X	X	National Agency of Statistics and Demography (Senegal), United Nations Children's Fund (UNICEF). Senegal - Dakar Urban Multiple Indicator Cluster Survey 2015-2016. New York, United States of America: United Nations Children's Fund (UNICEF), 2018.	287639
Senegal	2015	Senegal Continuous Demographic and Health Survey 2015	Survey microdata	GPS		X	X	X	X	X	X	X	Cheikh Anta Diop University, ICF International, National Agency of Statistics and Demography (Senegal). Senegal Continuous Demographic and Health Survey 2015. Fairfax, United States of America: ICF International, 2016.	218592

Country	Years	Name	Type	Geographic Detail	Male Circumcision	STI Symptoms	Had Intercourse	Partner Away	Condom Last Time	Multiple Partners in Year (Males	Multiple Partners in Year (Females)	In Union	Citation	NID
Senegal	2015	Senegal Global Adult Tobacco Survey 2015	Survey microdata	Admin 1								X	Centers for Disease Control and Prevention (CDC), Johns Hopkins Bloomberg School of Public Health, Ministry of Health and Social Action (Senegal), National Agency of Statistics and Demography (Senegal), Research Triangle Institute, Inc. (RTI), World Health Organization (WHO). Senegal Global Adult Tobacco Survey 2015.	237375
Senegal	2016	Senegal Continuous Demographic and Health Survey 2016	Survey microdata	GPS		X	X	X	X	X	X	X	ICF International, Ministry of Health and Social Action (Senegal), National Agency of Statistics and Demography (Senegal). Senegal Continuous Demographic and Health Survey 2016. Fairfax, United States of America: ICF International, 2017.	286772
Senegal	2017	Senegal Continuous Demographic and Health Survey 2017	Survey microdata	Admin 1		X	X	X	X	X	X	X	ICF International, Ministry of Health and Social Action (Senegal), National Agency of Statistics and Demography (Senegal), Unit for the Fight Against Malnutrition (Senegal). Senegal Continuous Demographic and Health Survey 2017. Fairfax, United States of America: ICF International, 2018.	353526

Country	Years	Name	Type	Geographic Detail	Male Circumcision	STI Symptoms	Had Intercourse	Partner Away	Condom Last Time	Multiple Partners in Year (Males	Multiple Partners in Year (Females)	In Union	Citation	NID
Sierra Leone	2000	Sierra Leone Multiple Indicator Cluster Survey 2000	Survey microdata	Admin 1								X	Central Statistics Office (Sierra Leone), United Nations Children's Fund (UNICEF). Sierra Leone Multiple Indicator Cluster Survey 2000. New York, United States of America: United Nations Children's Fund (UNICEF).	11639
Sierra Leone	2005	Sierra Leone Multiple Indicator Cluster Survey 2005	Survey microdata	Admin 2			X				X		United Nations Children's Fund (UNICEF), Statistics Sierra Leone. Sierra Leone Multiple Indicator Cluster Survey 2005. New York, United States: United Nations Children's Fund (UNICEF).	11649
Sierra Leone	2008	Sierra Leone Demographic and Health Survey 2008	Survey microdata	GPS	X	X	X	X	X	X	X	X	Macro International, Inc, Statistics Sierra Leone. Sierra Leone Demographic and Health Survey 2008. Fairfax, United States of America: ICF International.	21258

Country	Years	Name	Type	Geographic Detail	Male Circumcision	STI Symptoms	Had Intercourse	Partner Away	Condom Last Time	Multiple Partners in Year (Males	Multiple Partners in Year (Females)	In Union	Citation	NID
Sierra Leone	2010	Sierra Leone Multiple Indicator Cluster Survey 2010	Survey microdata	Admin 2			X				X		Statistics Sierra Leone, United Nations Children's Fund (UNICEF). Sierra Leone Multiple Indicator Cluster Survey 2010. New York, United States of America: United Nations Children's Fund (UNICEF).	76700
Sierra Leone	2013	Sierra Leone Demographic and Health Survey 2013	Survey microdata	GPS	X	X	X	X	X	X	X	X	ICF International, Ministry of Health and Sanitation (Sierra Leone), Statistics Sierra Leone. Sierra Leone Demographic and Health Survey 2013. Fairfax, United States of America: ICF International, 2014.	131467
Sierra Leone	2014	Sierra Leone Labor Force Survey 2014	Survey microdata	Admin 2								X	German Society for International Cooperation (GIZ), International Labour Organization (ILO), Statistics Sierra Leone, World Bank. Sierra Leone Labor Force Survey 2014.	286094

Country	Years	Name	Type	Geographic Detail	Male Circumcision	STI Symptoms	Had Intercourse	Partner Away	Condom Last Time	Multiple Partners in Year (Males	Multiple Partners in Year (Females)	In Union	Citation	NID
South Africa	2002	South Africa - Agincourt Integrated Family Survey 2002	Survey microdata	Admin 4								X	Case, A. 2003. Agincourt Integrated Family Survey 2002. [dataset] Version 1. Cape Town: DataFirst [distributor].	135825
South Africa	2002	South Africa General Household Survey 2002	Survey microdata	Admin 1								X	Statistics South Africa. South Africa General Household Survey 2002. Pretoria, South Africa: Statistics South Africa.	115481
South Africa	2002	South Africa HIV/AIDS Behavioral Risks, Sero-Status, and Mass Media Impact Survey 2002	Survey microdata	Admin 3	X		X		X	X	X	X	Center for AIDS Development, Research and Evaluation (CADRE) (South Africa), Department of Social Development (South Africa), Family Health International, Geospace International (South Africa), Human Sciences Research Council, Joint United Nations Program on HIV/AIDS (UNAIDS), Medical University of Southern Africa (MEDUNSA), National Agency for AIDS Research (ANRS) (France), National Health Laboratory Service (NHLS) (South Africa), South African Medical Research Council, University of Natal, Wits Health Consortium. South Africa HIV/AIDS Behavioral Risks, Sero-Status, and Mass Media Impact Survey 2002. Pretoria, South Africa: Human Sciences Research Council, 2011.	12102

Country	Years	Name	Type	Geographic Detail	Male Circumcision	STI Symptoms	Had Intercourse	Partner Away	Condom Last Time	Multiple Partners in Year (Males	Multiple Partners in Year (Females)	In Union	Citation	NID
South Africa	2003	South Africa General Household Survey 2003	Survey microdata	Admin 1								X	Statistics South Africa. South Africa General Household Survey 2003. Pretoria, South Africa: Statistics South Africa.	11787
South Africa	2004	South Africa General Household Survey 2004	Survey microdata	Admin 1								X	Statistics South Africa. South Africa General Household Survey 2004. Pretoria, South Africa: Statistics South Africa.	11788
South Africa	2005	South Africa General Household Survey 2005	Survey microdata	Admin 1								X	Statistics South Africa. South Africa General Household Survey 2005. Pretoria, South Africa: Statistics South Africa.	11789

Country	Years	Name	Type	Geographic Detail	Male Circumcision	STI Symptoms	Had Intercourse	Partner Away	Condom Last Time	Multiple Partners in Year (Males	Multiple Partners in Year (Females)	In Union	Citation	NID
South Africa	2005	South Africa National HIV Prevalence, Incidence, Behavior and Communication Survey 2004-2005	Survey microdata	Admin 3			X		X	X	X	X	Center for AIDS Development, Research and Evaluation (CADRE) (South Africa), Centers for Disease Control and Prevention (CDC), Global Clinical and Viral Laboratory (South Africa), Human Sciences Research Council, Maphume Research Services, National Institute for Communicable Diseases (South Africa). South Africa National HIV Prevalence, Incidence, Behavior and Communication Survey 2004-2005. Pretoria, South Africa: Human Sciences Research Council, 2011.	313074
South Africa	2006	South Africa General Household Survey 2006	Survey microdata	Admin 1								X	Statistics South Africa. South Africa General Household Survey 2006. Pretoria, South Africa: Statistics South Africa.	115486
South Africa	2007	South Africa General Household Survey 2007	Survey microdata	Admin 1								X	Statistics South Africa. South Africa General Household Survey 2007. Cape Town, South Africa: DataFirst.	11790

Country	Years	Name	Type	Geographic Detail	Male Circumcision	STI Symptoms	Had Intercourse	Partner Away	Condom Last Time	Multiple Partners in Year (Males	Multiple Partners in Year (Females)	In Union	Citation	NID
South Africa	2008	South Africa General Household Survey 2008	Survey microdata	Admin 1								X	Statistics South Africa. South Africa General Household Survey 2008. Pretoria, South Africa: Statistics South Africa.	115488
South Africa	2008-09	South Africa National HIV Prevalence, Incidence, Behavior and Communication Survey 2008-2009	Survey microdata	Admin 5, GPS	X	X	X		X	X	X	X	Center for AIDS Development, Research and Evaluation (CADRE) (South Africa), Geospace International (South Africa), Global Clinical and Viral Laboratory (South Africa), Human Sciences Research Council, Maphume Research Services, National Institute for Communicable Diseases (South Africa), South African Medical Research Council. South Africa National HIV Prevalence, Incidence, Behavior and Communication Survey 2008-2009. Pretoria, South Africa: Human Sciences Research Council, 2014.	228102
South Africa	2009	South Africa General Household Survey 2009	Survey microdata	Admin 1								X	Statistics South Africa. South Africa General Household Survey 2009. Pretoria, South Africa: Statistics South Africa.	115489

Country	Years	Name	Type	Geographic Detail	Male Circumcision	STI Symptoms	Had Intercourse	Partner Away	Condom Last Time	Multiple Partners in Year (Males	Multiple Partners in Year (Females)	In Union	Citation	NID
South Africa	2009	South Africa Living Conditions Survey 2008-2009	Survey microdata	Admin 1								X	Statistics South Africa. South Africa Living Conditions Survey 2008-2009. Pretoria, South Africa: Statistics South Africa.	152021
South Africa	2010-11	South Africa National Income Dynamics Study - Wave 2 2010-2011	Survey microdata	Admin 1, Admin 2,								X	Southern Africa Labour and Development Research Unit. National Income Dynamics Study 2010-2011, Wave 2. Version 1.0. Cape Town: Southern Africa Labour and Development Research Unit [producer], 2012. Cape Town: DataFirst [distributor], 2013.	133731
South Africa	2011-12	South Africa National HIV Prevalence, Incidence, and Behavior Survey 2011-2012	Survey microdata	Admin 5, GPS	X		X		X	X	X	X	Centers for Disease Control and Prevention (CDC), Global Clinical and Viral Laboratory (South Africa), Human Sciences Research Council, National Institute for Communicable Diseases (South Africa), South African Medical Research Council, University of Cape Town. South Africa National HIV Prevalence, Incidence, and Behavior Survey 2011-2012. Pretoria, South Africa: Human Sciences Research Council, 2016.	313076

Country	Years	Name	Type	Geographic Detail	Male Circumcision	STI Symptoms	Had Intercourse	Partner Away	Condom Last Time	Multiple Partners in Year (Males	Multiple Partners in Year (Females)	In Union	Citation	NID
South Africa	2013	South Africa General Household Survey 2013	Survey microdata	Admin 1								X	Statistics South Africa. General Household Survey 2013 [dataset]. Version 1. Pretoria. Statistics South Africa [producer], 2014. Cape Town. DataFirst [distributor], 2014.10.25828/a704-wm31	238483
South Africa	2014	South Africa General Household Survey 2014	Survey microdata	Admin 1								X	Statistics South Africa. South Africa General Household Survey 2014. Cape Town, South Africa: DataFirst, 2015.	238485
South Africa	2015	South Africa Living Conditions Survey 2014-2015	Survey microdata	Admin 1								X	Statistics South Africa. South Africa Living Conditions Survey 2014-2015. Pretoria, South Africa: Statistics South Africa.	317239

Country	Years	Name	Type	Geographic Detail	Male Circumcision	STI Symptoms	Had Intercourse	Partner Away	Condom Last Time	Multiple Partners in Year (Males	Multiple Partners in Year (Females)	In Union	Citation	NID
South Africa	2016	South Africa Community Survey 2016	Survey microdata	Admin 3								X	Statistics South Africa. South Africa Community Survey 2016. Pretoria, South Africa: Statistics South Africa, 2016.	280803
South Africa	2016	South Africa Demographic and Health Survey 2016	Survey microdata	GPS	X	X	X	X	X	X	X	X	Department of Health (South Africa), ICF International, South African Medical Research Council, Statistics South Africa. South Africa Demographic and Health Survey 2016. Fairfax, United States of America: ICF International, 2019.	157064
South Africa	2016	South Africa General Household Survey 2016	Survey microdata	Admin 1								X	Statistics South Africa. South Africa General Household Survey 2016. Cape Town, South Africa: DataFirst, 2015.	317089

Country	Years	Name	Type	Geographic Detail	Male Circumcision	STI Symptoms	Had Intercourse	Partner Away	Condom Last Time	Multiple Partners in Year (Males	Multiple Partners in Year (Females)	In Union	Citation	NID
South Sudan	2010	Sudan - South Multiple Indicator Cluster Survey 2010	Survey microdata	Admin 1			X				X	X	Central Bureau of Statistics (Sudan), Federal Ministry of Health (Sudan), Government of Sudan, Ministry of Health (South Sudan), Southern Sudan Centre for Census, Statistics and Evaluation. Sudan - South Multiple Indicator Cluster Survey 2010. New York, United States of America: United Nations Children's Fund (UNICEF), 2015.	32189
Sudan	2000	Sudan Multiple Indicator Cluster Survey 2000	Survey microdata	Admin 1								X	Central Bureau of Statistics (Sudan), Federal Ministry of Health (Sudan), United Nations Children's Fund (UNICEF). Sudan Multiple Indicator Cluster Survey 2000. New York, United States of America: United Nations Children's Fund (UNICEF).	12243
Sudan	2010	Sudan - North Multiple Indicator Cluster Survey 2010	Survey microdata	Admin 1								X	Central Bureau of Statistics (Sudan), Ministry of Health (South Sudan). Sudan - North Multiple Indicator Cluster Survey 2010. New York, United States of America: United Nations Children's Fund (UNICEF), 2015.	153643

Country	Years	Name	Type	Geographic Detail	Male Circumcision	STI Symptoms	Had Intercourse	Partner Away	Condom Last Time	Multiple Partners in Year (Males	Multiple Partners in Year (Females)	In Union	Citation	NID
Togo	2000	Togo Multiple Indicator Cluster Survey 2000	Survey microdata	Admin 1, Admin 2								X	United Nations Children's Fund (UNICEF). Togo Multiple Indicator Cluster Survey 2000. New York, United States of America: United Nations Children's Fund (UNICEF).	12886
Togo	2006	Togo Multiple Indicator Cluster Survey 2006	Survey microdata	Admin 1			X				X		Directorate General of Statistics and National Accounting (Togo), United Nations Children's Fund (UNICEF). Togo Multiple Indicator Cluster Survey 2006. New York, United States: United Nations Children's Fund (UNICEF).	12896
Togo	2010	Togo Multiple Indicator Cluster Survey 2010	Survey microdata	Admin 1			X		X	X	X	X	Directorate General of Statistics and National Accounting (Togo), United Nations Children's Fund (UNICEF). Togo Multiple Indicator Cluster Survey 2010. New York, United States: United Nations Children's Fund (UNICEF).	40021

Country	Years	Name	Type	Geographic Detail	Male Circumcision	STI Symptoms	Had Intercourse	Partner Away	Condom Last Time	Multiple Partners in Year (Males	Multiple Partners in Year (Females)	In Union	Citation	NID
Togo	2013-14	Togo Demographic and Health Survey 2013-2014	Survey microdata	GPS	X	X	X	X	X	X	X	X	Directorate General of Statistics and National Accounts (Togo), ICF International, Ministry of Health (Togo), Ministry of Planning, Development and Zoning (Togo). Togo Demographic and Health Survey 2013-2014. Fairfax, United States of America: ICF International, 2015.	77515
Togo	2017	Togo Malaria Indicator Survey 2017	Survey microdata	GPS								X	ICF International, National Institute of Hygiene, Ministry of Health (Togo), National Institute of Statistics and Economic and Demographic Studies (INSEED) (Togo), National Program to Fight Malaria, Ministry of Health (Togo). Togo Malaria Indicator Survey 2017. Fairfax, United States of America: ICF International, 2018.	359318
Uganda	2000-01	Uganda Demographic and Health Survey 2000-2001	Survey microdata	GPS		X	X	X	X	X	X	X	Macro International, Inc, Uganda Bureau of Statistics. Uganda Demographic and Health Survey 2000-2001. Fairfax, United States of America: ICF International.	20993

Country	Years	Name	Type	Geographic Detail	Male Circumcision	STI Symptoms	Had Intercourse	Partner Away	Condom Last Time	Multiple Partners in Year (Males	Multiple Partners in Year (Females)	In Union	Citation	NID
Uganda	2004	Uganda National Service Delivery Survey 2004	Survey microdata	Admin 1								X	Ministry of Public Service (Uganda), Uganda Bureau of Statistics. Uganda National Service Delivery Survey 2004. OpenMicroData.	13114
Uganda	2004-05	Uganda AIDS Indicator Survey 2004-2005	Survey microdata	Admin 1	X	X	X			X	X	X	Division of Reproductive Health, Centers for Disease Control and Prevention (CDC), Ministry of Health (Uganda). Uganda AIDS Indicator Survey 2004-2005.	13084
Uganda	2006	Uganda Demographic and Health Survey 2006	Survey microdata	GPS	X	X	X	X	X	X	X	X	Macro International, Inc, Uganda Bureau of Statistics. Uganda Demographic and Health Survey 2006. Fairfax, United States of America: ICF International.	21014

Country	Years	Name	Type	Geographic Detail	Male Circumcision	STI Symptoms	Had Intercourse	Partner Away	Condom Last Time	Multiple Partners in Year (Males	Multiple Partners in Year (Females)	In Union	Citation	NID
Uganda	2008	Uganda National Service Delivery Survey 2008	Survey microdata	Admin 1								X	Ministry of Public Service (Uganda), Uganda Bureau of Statistics. Uganda National Service Delivery Survey 2008.	93320
Uganda	2011	Uganda AIDS Indicator Survey 2011	Survey microdata	GPS	X	X	X	X	X	X	X	X	Centers for Disease Control and Prevention (CDC), ICF Macro, Ministry of Health (Uganda), Uganda Bureau of Statistics, Uganda Virus Research Institute. Uganda AIDS Indicator Survey 2011. Fairfax, United States of America: ICF International.	55973
Uganda	2011	Uganda Demographic and Health Survey 2011	Survey microdata	GPS	X	X	X	X	X	X	X	X	ICF Macro, Uganda Bureau of Statistics. Uganda Demographic and Health Survey 2011. Fairfax, United States of America: ICF International.	56021

Country	Years	Name	Type	Geographic Detail	Male Circumcision	STI Symptoms	Had Intercourse	Partner Away	Condom Last Time	Multiple Partners in Year (Males	Multiple Partners in Year (Females)	In Union	Citation	NID
Uganda	2013	Uganda National Household Survey 2012-2013	Survey microdata	Admin 1								X	Uganda Bureau of Statistics. Uganda National Household Survey 2012-2013. Kampala, Uganda: Uganda Bureau of Statistics.	165406
Uganda	2014	Uganda Performance Monitoring and Accountability 2020 Survey, Round 1 2014	Survey microdata	Admin 1			X	X				X	Makerere University, School of Public Health at the College of Health Sciences and The Bill & Melinda Gates Institute for Population and Reproductive Health at The Johns Hopkins Bloomberg School of Public Health. Performance Monitoring and Accountability 2020 (PMA2020) Survey round 1, PMA2014/Uganda-R1. 2014. Uganda and Baltimore, Maryland, USA.	256199
Uganda	2015	Uganda Performance Monitoring and Accountability 2020 Survey, Round 2 2015	Survey microdata	Admin 1			X	X				X	Makerere University, School of Public Health at the College of Health Sciences and The Bill & Melinda Gates Institute for Population and Reproductive Health at The Johns Hopkins Bloomberg School of Public Health. Performance Monitoring and Accountability 2020 (PMA2020) Survey round 2, PMA2015/Uganda-R2. 2015. Uganda and Baltimore, Maryland, USA.	256200

Country	Years	Name	Type	Geographic Detail	Male Circumcision	STI Symptoms	Had Intercourse	Partner Away	Condom Last Time	Multiple Partners in Year (Males	Multiple Partners in Year (Females)	In Union	Citation	NID
Uganda	2015	Uganda Performance Monitoring and Accountability 2020 Survey, Round 3 2015	Survey microdata	Admin 1			X	X				X	Makerere University, School of Public Health at the College of Health Sciences and The Bill & Melinda Gates Institute for Population and Reproductive Health at The Johns Hopkins Bloomberg School of Public Health. Performance Monitoring and Accountability 2020 (PMA2020) Survey round 3, PMA2015/Uganda-R3. 2015. Uganda and Baltimore, Maryland, USA.	256201
Uganda	2016	Uganda Demographic and Health Survey 2016	Survey microdata	GPS	X	X	X	X	X	X	X	X	ICF International, Uganda Bureau of Statistics. Uganda Demographic and Health Survey 2016. Fairfax, United States of America: ICF International, 2018.	286780
Uganda	2016	Uganda Performance Monitoring and Accountability 2020 Survey, Round 4 2016	Survey microdata	Admin 1			X	X				X	Makerere University, School of Public Health at the College of Health Sciences and The Bill & Melinda Gates Institute for Population and Reproductive Health at The Johns Hopkins Bloomberg School of Public Health. Performance Monitoring and Accountability 2020 (PMA2020) Survey round 4, PMA2016/Uganda-R4. 2016. Uganda and Baltimore, Maryland, USA.	285893

Country	Years	Name	Type	Geographic Detail	Male Circumcision	STI Symptoms	Had Intercourse	Partner Away	Condom Last Time	Multiple Partners in Year (Males	Multiple Partners in Year (Females)	In Union	Citation	NID
Uganda	2016	Uganda Population-based HIV Impact Assessment 2016-2017	Survey report	Admin 1	X								Centers for Disease Control and Prevention (CDC), ICAP, Columbia University Mailman School of Public Health, Joint United Nations Program on HIV/AIDS (UNAIDS), Ministry of Health (Uganda), Uganda Bureau of Statistics, Uganda Virus Research Institute, World Health Organization (WHO). Uganda Population-Based HIV Impact Assessment 2016-2017.	327593
Uganda	2017	Uganda Performance Monitoring and Accountability 2020 Survey, Round 5 2017	Survey microdata	Admin >1			X	X				X	Makerere University, School of Public Health at the College of Health Sciences and The Bill & Melinda Gates Institute for Population and Reproductive Health at The Johns Hopkins Bloomberg School of Public Health. Performance Monitoring and Accountability 2020 (PMA2020) Survey round 5, PMA2017/Uganda-R5. 2017. Uganda and Baltimore, Maryland, USA.	347043
Uganda	2018	Uganda Performance Monitoring and Accountability 2020 Survey, Round 6 2018	Survey microdata	Admin 1			X					X	Makerere University, School of Public Health at the College of Health Sciences and The Bill & Melinda Gates Institute for Population and Reproductive Health at The Johns Hopkins Bloomberg School of Public Health. Performance Monitoring and Accountability 2020 (PMA2020) Survey round 6, PMA2018/Uganda-R6. 2018. Uganda and Baltimore, Maryland, USA.	398722

Country	Years	Name	Type	Geographic Detail	Male Circumcision	STI Symptoms	Had Intercourse	Partner Away	Condom Last Time	Multiple Partners in Year (Males	Multiple Partners in Year (Females)	In Union	Citation	NID
United Republic of Tanzania	2001	Tanzania Household Budget Survey 2000-2001	Survey microdata	Admin 1								X	National Bureau of Statistics (Tanzania), Oxford Policy Management. Tanzania Household Budget Survey 2000-2001. Dar es Salaam, Tanzania: National Bureau of Statistics (Tanzania).	31740
United Republic of Tanzania	2003-04	Tanzania AIDS Indicator Survey 2003-2004	Survey microdata	GPS	X	X	X		X	X	X	X	National Bureau of Statistics (Tanzania), ORC Macro, Tanzania Commission for AIDS (TACAIDS). Tanzania AIDS Indicator Survey 2003-2004. Fairfax, United States of America: ICF International.	12630
United Republic of Tanzania	2004	Tanzania - Shinyanga Core Welfare Indicators Questionnaire Survey 2004	Survey microdata	Admin 2								X	Economic Development Initiatives (EDI), National Bureau of Statistics (Tanzania), Institute of Finance Management (Tanzania), World Bank (WB). Tanzania - Shinyanga Core Welfare Indicators Questionnaire Survey 2004. Bukoba, Tanzania: Economic Development Initiatives (EDI).	31786

Country	Years	Name	Type	Geographic Detail	Male Circumcision	STI Symptoms	Had Intercourse	Partner Away	Condom Last Time	Multiple Partners in Year (Males	Multiple Partners in Year (Females)	In Union	Citation	NID
United Republic of Tanzania	2004-05	Tanzania Demographic and Health Survey 2004-2005	Survey microdata	Admin 1	X	X	X	X	X	X	X	X	Macro International, Inc, National Bureau of Statistics (Tanzania). Tanzania Demographic and Health Survey 2004-2005. Fairfax, United States of America: ICF International.	20875
United Republic of Tanzania	2005	Tanzania Core Welfare Indicators Questionnaire Survey 2005	Survey microdata	Admin 2								X	Economic Development Initiatives (EDI), World Bank (WB). Tanzania Core Welfare Indicators Questionnaire Survey 2005. Bukoba, Tanzania: Economic Development Initiatives (EDI).	31797
United Republic of Tanzania	2006-07	Tanzania Core Welfare Indicators Questionnaire Survey 2006-2007	Survey microdata	Admin 2								X	Economic Development Initiatives (EDI), World Bank (WB). Tanzania Core Welfare Indicators Questionnaire Survey 2006-2007. Bukoba, Tanzania: Economic Development Initiatives (EDI).	31831

Country	Years	Name	Type	Geographic Detail	Male Circumcision	STI Symptoms	Had Intercourse	Partner Away	Condom Last Time	Multiple Partners in Year (Males	Multiple Partners in Year (Females)	In Union	Citation	NID
United Republic of Tanzania	2007	Tanzania Household Budget Survey 2007	Survey microdata	Admin 1								X	National Bureau of Statistics (Tanzania). Tanzania Household Budget Survey 2007. Dar es Salaam, Tanzania: National Bureau of Statistics (Tanzania).	31887
United Republic of Tanzania	2007-08	Tanzania HIV/AIDS and Malaria Indicator Survey 2007-2008	Survey microdata	GPS	X	X	X	X	X	X	X	X	Macro International, Inc, National Bureau of Statistics (Tanzania), Office of the Chief Government Statistician (OCGS) (Zanzibar), Tanzania Commission for AIDS (TACAIDS), Zanzibar AIDS Commission (ZAC). Tanzania HIV/AIDS and Malaria Indicator Survey 2007-2008. Fairfax, United States of America: ICF International.	12644
United Republic of Tanzania	2009-10	Tanzania Demographic and Health Survey 2009-2010	Survey microdata	GPS	X	X	X	X	X	X	X	X	ICF Macro, National Bureau of Statistics (Tanzania). Tanzania Demographic and Health Survey 2009-2010. Fairfax, United States of America: ICF International.	21331

Country	Years	Name	Type	Geographic Detail	Male Circumcision	STI Symptoms	Had Intercourse	Partner Away	Condom Last Time	Multiple Partners in Year (Males	Multiple Partners in Year (Females)	In Union	Citation	NID
United Republic of Tanzania	2011-12	Tanzania AIDS Indicator Survey 2011-2012	Survey microdata	GPS	X	X	X	X	X	X	X	X	ICF International, National Bureau of Statistics (Tanzania), Office of the Chief Government Statistician (OCGS) (Zanzibar), Tanzania Commission for AIDS (TACAIDS), Zanzibar AIDS Commission (ZAC). Tanzania AIDS Indicator Survey 2011-2012. Fairfax, United States of America: ICF International, 2013.	77395
United Republic of Tanzania	2012	Tanzania Household Budget Survey 2011-2012	Survey microdata	Admin 1								X	National Bureau of Statistics (Tanzania). Tanzania Household Budget Survey 2011-2012. Dar es Salaam, Tanzania: National Bureau of Statistics (Tanzania), 2014.	243012
United Republic of Tanzania	2015	Tanzania National Panel Survey 2014-2016	Survey microdata	GPS								X	National Bureau of Statistics (Tanzania), World Bank. Tanzania National Panel Survey 2014-2016. Washington DC, United States of America: World Bank.	311265

Country	Years	Name	Type	Geographic Detail	Male Circumcision	STI Symptoms	Had Intercourse	Partner Away	Condom Last Time	Multiple Partners in Year (Males	Multiple Partners in Year (Females)	In Union	Citation	NID
United Republic of Tanzania	2015-16	Tanzania Demographic and Health Survey 2015-2016	Survey microdata	GPS	X		X	X	X			X	ICF International, Ministry of Health (Zanzibar), Ministry of Health, Community Development, Gender, Elderly and Children (MoHCDEC) (Tanzania), National Bureau of Statistics (Tanzania), Office of the Chief Government Statistician (OCGS) (Zanzibar). Tanzania Demographic and Health Survey 2015-2016. Fairfax, United States of America: ICF International, 2016.	218593
United Republic of Tanzania	2017	Tanzania HIV Impact Survey 2016-2017	Survey microdata	Admin 1	X								Centers for Disease Control and Prevention (CDC), ICAP, Columbia University Mailman School of Public Health, Ministry of Health (Zanzibar), Ministry of Health, Community Development, Gender, Elderly and Children (MoHCDEC) (Tanzania), National AIDS Control Program (NACP) (Tanzania), National Bureau of Statistics (Tanzania), National Health Laboratory Quality Assurance and Training Centre (NHLQATC) (Tanzania), Office of the Chief Government Statistician (OCGS) (Zanzibar), Tanzania Commission for AIDS (TACAIDS), Zanzibar AIDS Commission (ZAC), Zanzibar Integrated HIV, Tuberculosis and Leprosy Program (ZIHTLP). Tanzania HIV Impact Survey 2016-2017.	327591
Zambia	2000	Zambia Sexual Behavior Survey 2000	Survey microdata	Admin 2	X	X	X	X		X	X	X	Central Board of Health (Zambia), Central Statistical Office (Zambia), MEASURE Evaluation Project, Carolina Population Center, University of North Carolina, University of Zambia, Zambian National AIDS Council. Zambia Sexual Behavior Survey 2000. Lusaka, Zambia: Central Statistical Office (Zambia).	27924

Country	Years	Name	Type	Geographic Detail	Male Circumcision	STI Symptoms	Had Intercourse	Partner Away	Condom Last Time	Multiple Partners in Year (Males	Multiple Partners in Year (Females)	In Union	Citation	NID
Zambia	2001-02	Zambia Demographic and Health Survey 2001-2002	Survey microdata	Admin 2		X	X	X	X	X	X	X	Central Board of Health (Zambia), Central Statistical Office (Zambia), Macro International, Inc. Zambia Demographic and Health Survey 2001-2002. Fairfax, United States of America: ICF International.	21102
Zambia	2003	Zambia Living Conditions Monitoring Survey 2002-2003	Survey microdata	Admin 2								X	Central Statistical Office (Zambia). Zambia Living Conditions Monitoring Survey 2002-2003. Lusaka, Zambia: Central Statistical Office (Zambia).	14027
Zambia	2003	Zambia Sexual Behavior Survey 2003	Survey microdata	Admin 2	X	X	X	X				X	Central Board of Health (Zambia), Central Statistical Office (Zambia), MEASURE Evaluation Project, Carolina Population Center, University of North Carolina, Zambian National AIDS Council. Zambia Sexual Behavior Survey 2003. Lusaka, Zambia: Central Statistical Office (Zambia).	27952

Country	Years	Name	Type	Geographic Detail	Male Circumcision	STI Symptoms	Had Intercourse	Partner Away	Condom Last Time	Multiple Partners in Year (Males	Multiple Partners in Year (Females)	In Union	Citation	NID
Zambia	2005	Zambia Sexual Behavior Survey 2005	Survey microdata	Admin 2	X	X	X	X		X	X	X	Central Statistical Office (Zambia), Department for International Development (DFiD) (United Kingdom), Family Health International, MEASURE Evaluation Project, Carolina Population Center, University of North Carolina, Ministry of Health (Zambia), University of Zambia, Zambian National AIDS Council. Zambia Sexual Behavior Survey 2005. Lusaka, Zambia: Central Statistical Office (Zambia).	27987
Zambia	2007	Zambia Demographic and Health Survey 2007	Survey microdata	GPS	X	X	X	X	X	X	X	X	Central Statistical Office (Zambia), Macro International, Inc. Zambia Demographic and Health Survey 2007. Fairfax, United States of America: ICF International.	21117
Zambia	2008	Zambia Global Fund Household Health Coverage Survey 2008	Survey microdata	Admin 3			X	X					Central Statistical Office (Zambia). Zambia Global Fund Household Health Coverage Survey 2008. Lusaka, Zambia: Central Statistical Office (Zambia).	26702

Country	Years	Name	Type	Geographic Detail	Male Circumcision	STI Symptoms	Had Intercourse	Partner Away	Condom Last Time	Multiple Partners in Year (Males	Multiple Partners in Year (Females)	In Union	Citation	NID
Zambia	2009	Zambia Sexual Behavior Survey 2009	Survey microdata	Admin 2	X	X	X	X		X	X	X	Central Statistical Office (Zambia), MEASURE Evaluation Project, Carolina Population Center, University of North Carolina, Ministry of Health (Zambia), University of Zambia, Zambian National AIDS Council. Zambia Sexual Behavior Survey 2009.	59339
Zambia	2010	Zambia Living Conditions Monitoring Survey 2010	Survey microdata	Admin 3								X	Central Statistical Office (Zambia). Zambia Living Conditions Monitoring Survey 2010.	58660
Zambia	2013-14	Zambia Demographic and Health Survey 2013-2014	Survey microdata	GPS	X	X	X	X	X	X	X	X	Central Statistical Office (Zambia), ICF International, Ministry of Health (Zambia), Tropical Diseases Research Centre, University Teaching Hospital (Zambia), University of Zambia. Zambia Demographic and Health Survey 2013-2014. Fairfax, United States of America: ICF International.	77516

Country	Years	Name	Type	Geographic Detail	Male Circumcision	STI Symptoms	Had Intercourse	Partner Away	Condom Last Time	Multiple Partners in Year (Males	Multiple Partners in Year (Females)	In Union	Citation	NID
Zambia	2016	Zambia Population-based HIV Impact Assessment 2016	Survey microdata	Admin 2	X	X	X	X	X	X	X	X	Ministry of Health, Zambia, Centers for Disease Control and Prevention (CDC), and ICAP at Columbia University. Zambia Population-based HIV Impact Assessment (ZAMPHIA) 2015-16: First Report. Lusaka, Zambia, Atlanta, Georgia and New York, New York, USA: Ministry of Health, CDC and ICAP. December 2016.	287630
Zimbabwe	2005	Zimbabwe - Chimanimani Behavioral Risks and HIV Serostatus Survey 2005	Survey report	Admin 2	X								Biomedical Research and Training Institute (Zimbabwe), Human Sciences Research Council, National Institute of Health Research, Ministry of Health and Child Welfare (NIHR) (Zimbabwe), Zimbabwe Central Statistics Office. Zimbabwe - Chimanimani Behavioral Risks and HIV Serostatus Survey 2005.	333662
Zimbabwe	2005-06	Zimbabwe Demographic and Health Survey 2005-2006	Survey microdata	GPS	X	X	X	X	X	X	X	X	Central Statistical Office (Zimbabwe), Macro International, Inc. Zimbabwe Demographic and Health Survey 2005-2006. Fairfax, United States of America: ICF International.	21163

Country	Years	Name	Type	Geographic Detail	Male Circumcision	STI Symptoms	Had Intercourse	Partner Away	Condom Last Time	Multiple Partners in Year (Males	Multiple Partners in Year (Females)	In Union	Citation	NID
Zimbabwe	2009	Zimbabwe Multiple Indicator Monitoring Survey 2009	Survey microdata	Admin 1								X	Central Statistical Office (Zimbabwe). Zimbabwe Multiple Indicator Monitoring Survey 2009. New York, United States of America: United Nations Children's Fund (UNICEF).	35493
Zimbabwe	2010-11	Zimbabwe Demographic and Health Survey 2010-2011	Survey microdata	GPS	X	X	X	X	X	X	X	X	ICF Macro, Zimbabwe National Statistics Agency. Zimbabwe Demographic and Health Survey 2010-2011. Calverton, United States of America: ICF Macro, 2012.	55992
Zimbabwe	2014	Zimbabwe Multiple Indicator Cluster Survey 2014	Survey microdata	Admin 1	X		X		X	X	X	X	United Nations Children's Fund (UNICEF), Zimbabwe National Statistics Agency. Zimbabwe Multiple Indicator Cluster Survey 2014. New York, United States of America: United Nations Children's Fund (UNICEF), 2015.	152720

Country	Years	Name	Type	Geographic Detail	Male Circumcision	STI Symptoms	Had Intercourse	Partner Away	Condom Last Time	Multiple Partners in Year (Males	Multiple Partners in Year (Females)	In Union	Citation	NID
Zimbabwe	2015	Zimbabwe Demographic and Health Survey 2015	Survey microdata	GPS	X	X	X	X	X	X	X	X	ICF International, National Microbiology Reference Laboratory, Harare Central Hospital (NMRL) (Zimbabwe), Zimbabwe National Statistics Agency. Zimbabwe Demographic and Health Survey 2015. Fairfax, United States of America: ICF International, 2016.	157066
Zimbabwe	2016	Zimbabwe Population-based HIV Impact Assessment 2015-2016	Survey report	Admin 1	X								Ministry of Health and Child Care (MOHCC), Zimbabwe, Centers for Disease Control and Prevention (CDC), and ICAP at Columbia University. Zimbabwe Population-based HIV Impact Assessment (ZIMPHIA) 2015-16: First Report. Harare, Zimbabwe, Atlanta, Georgia and New York, New York, USA: Ministry of Health, CDC and ICAP. December 2016.	287631

24

Table S6: Fitted model parameters

	Central sub-Saharan Africa			Eastern sub-Saharan Africa			Southern sub-Saharan Africa			Western sub-Saharan Africa		
Parameter	0.025th quantile	0.500th quantile	0.975th quantile	0.025th quantile	0.500th quantile	0.975th quantile	0.025th quantile	0.500th quantile	0.975th quantile	0.025th quantile	0.500th quantile	0.975th quantile
β_0	0.253685	0.515121	0.770286	-0.53288	-0.50422	-0.47701	-0.14979	-0.10188	-0.05592	-0.15705	-0.03732	0.078244
β_1 (GAM)	-0.02418	0.008061	0.041656	-0.02213	0.007645	0.037226	-0.16251	-0.12408	-0.08363	0.060003	0.092049	0.126741
β_1 (BRT)	1.403007	1.482141	1.559645	1.315374	1.33394	1.351832	1.800981	1.83124	1.860829	1.219898	1.267047	1.310912
β_1 (Lasso)	-0.35894	-0.2707	-0.18163	-0.06546	-0.03467	-0.00289	-0.39136	-0.34116	-0.28706	-0.19268	-0.1486	-0.10299
β_2	0.451629	0.498326	0.542133	0.098234	0.112439	0.125856	0.254796	0.266349	0.277243	0.380631	0.407404	0.432713
ρ_{time} for Z_1	0.883892	0.908635	0.928452	0.897637	0.906859	0.915387	0.895411	0.910193	0.923208	0.932207	0.942362	0.951111
ρ_{time} for Z_2	0.865307	0.920794	0.95215	0.965366	0.978747	0.986807	0.961727	0.975977	0.984737	0.953252	0.972237	0.983287
ρ_{age} for Z_2	0.716841	0.827258	0.901701	0.746117	0.822233	0.884198	0.637884	0.738138	0.816578	0.714179	0.812046	0.881451
ρ_{sex} for Z_2	0.533708	0.74552	0.87028	0.610472	0.741752	0.838326	0.583274	0.709504	0.804087	0.527538	0.704386	0.828723
Variance for ϵ_i	0.048589	0.055504	0.062965	0.057004	0.059642	0.062256	0.013574	0.014608	0.015661	0.103492	0.108788	0.114034
Variance for Z_1	0.176178	0.227678	0.287913	0.283422	0.310762	0.33768	0.104448	0.123508	0.143115	0.188747	0.224224	0.261827
Variance for Z_2	0.190976	0.329295	0.561731	0.277958	0.46078	0.755944	0.306212	0.489873	0.77307	0.208818	0.346227	0.567555
Variance for Z_3	0.043876	0.094688	0.212505	0.576524	0.912741	1.475894	0.026892	0.051968	0.101989	0.115152	0.182138	0.291149
ρ_{age} for Z_3	0.654685	0.838997	0.930165	0.985394	0.991968	0.995635	0.602617	0.797226	0.903595	0.804755	0.884715	0.933775
Range for Z_1	0.071699	0.083626	0.097649	0.026409	0.028002	0.029697	0.042837	0.04682	0.051193	0.046778	0.051456	0.056636
Variance for $U_{s[i]}$	0.091564	0.11615	0.146341	0.171158	0.190925	0.212143	0.054512	0.064978	0.076985	0.100137	0.112816	0.126748
