# Supplementary material for: Mapping age- and sex-specific HIV prevalence in adults in sub-Saharan Africa, 2000–2018
Source: BMC Med. 2022 Dec 19;20:488. doi: 10.1186/s12916-022-02639-z (PMC9760541; doi:10.1186/s12916-022-02639-z)
Supplement: Supplementary file 3 — Additional file 3: Supplemental figures.Figure S1. Prevalence of male circumcision. Figure S2. Prevalence of signs and symptoms of sexually transmitted infections. Figure S3. Prevalence of marriage or living as married. Figure S4. Prevalence of partner living elsewhere among females. Figure S5. Prevalence of condom use during most recent sexual encounter. Figure S6. Prevalence of sexual activity among young females. Figure S7. Prevalence of multiple partners among males in the past year. Figure S8. Prevalence of multiple partners among females in the past year. Figure S9. HIV prevalence predictions from the boosted regression tree model. Figure S10. HIV prevalence predictions from the generalized additive model. Figure S11. HIV prevalence predictions from the lasso regression model. Figure S12. Modeling regions. Figure S13. Age- and sex-specific vs. adult prevalence modeling. Figure S14. Data sensitivity. Figure S15. Model specification validation. Figure S16. Modeled and re-aggregated adult prevalence comparison. Figure S17. HIV prevalence raking factors for males. Figure S18. HIV prevalence raking factors for females. Figure S19. Age-specific HIV prevalence in males, 2000. Figure S20. Age-specific HIV prevalence in females, 2000. Figure S21. Age-specific HIV prevalence in males, 2005. Figure S22. Age-specific HIV prevalence in females, 2005. Figure S23. Age-specific HIV prevalence in males, 2010. Figure S24. Age-specific HIV prevalence in females, 2010. Figure S25. Age-specific HIV prevalence in males, 2018. Figure S26. Age-specific HIV prevalence in females, 2018. Figure S27. Age-specific uncertainty interval range estimates in males, 2000. Figure S28. Age-specific uncertainty interval range estimates in females, 2000. Figure S29. Age-specific uncertainty interval range estimates in males, 2005. Figure S30. Age-specific uncertainty interval range estimates in females, 2005. Figure S31. Age-specific uncertainty interval range estimates in males, 2010. Figure S32. [file 12916_2022_2639_MOESM3_ESM.pdf]

Additional File 3: Supplemental Figures

Mapping age- and sex-specific HIV prevalence in adults in sub-Saharan Africa, 2000–2018

Contents

Figure S1: Prevalence of male circumcision 3
Figure S2: Prevalence of signs and symptoms of sexually transmitted infections 4
Figure S3: Prevalence of marriage or living as married 5
Figure S4: Prevalence of partner living elsewhere among females 6
Figure S5: Prevalence of condom use during most recent sexual encounter 7
Figure S6: Prevalence of sexual activity among young females 8
Figure S7: Prevalence of multiple partners among males in the past year 9
Figure S8: Prevalence of multiple partners among females in the past year 10
Figure S9: HIV prevalence predictions from the boosted regression tree model 11
Figure S10: HIV prevalence predictions from the generalized additive model 12
Figure S11: HIV prevalence predictions from the lasso regression model 13
Figure S12: Modeling regions 14
Figure S13: Age- and sex-specific vs. adult prevalence modeling 15
Figure S14: Data sensitivity 16
Figure S15: Model specification validation 17
Figure S16: Modeled and re-aggregated adult prevalence comparison 18
Figure S17: HIV prevalence raking factors for males 19
Figure S18: HIV prevalence raking factors for females 20
Figure S19: Age-specific HIV prevalence in males, 2000 21
Figure S20: Age-specific HIV prevalence in females, 2000 23
Figure S21: Age-specific HIV prevalence in males, 2005 25
Figure S22: Age-specific HIV prevalence in females, 2005 27
Figure S23: Age-specific HIV prevalence in males, 2010 29
Figure S24: Age-specific HIV prevalence in females, 2010 31
Figure S25: Age-specific HIV prevalence in males, 2018 33
Figure S26: Age-specific HIV prevalence in females, 2018 35

Figure S27: Age-specific uncertainty interval range estimates in males, 2000 37
Figure S28: Age-specific uncertainty interval range estimates in females, 2000 39
Figure S29: Age-specific uncertainty interval range estimates in males, 2005 41
Figure S30: Age-specific uncertainty interval range estimates in females, 2005 43
Figure S31: Age-specific uncertainty interval range estimates in males, 2010 45
Figure S32: Age-specific uncertainty interval range estimates in females, 2010 47
Figure S33: Age-specific uncertainty interval range estimates in males, 2018 49
Figure S34: Age-specific uncertainty interval range estimates in females, 2018 51
Figure S35: Change in HIV prevalence in males, 2000–2005 53
Figure S36: Change in HIV prevalence in females, 2000–2005 55
Figure S37: Change in HIV prevalence in males, 2005–2010 57
Figure S38: Change in HIV prevalence in females, 2005–2010 59
Figure S39: Change in HIV prevalence in males, 2010–2018 61
Figure S40: Change in HIV prevalence in females, 2010–2018 63
Figure S41: Spatial mesh for geostatistical models 65

Figure S1: Prevalence of male circumcision

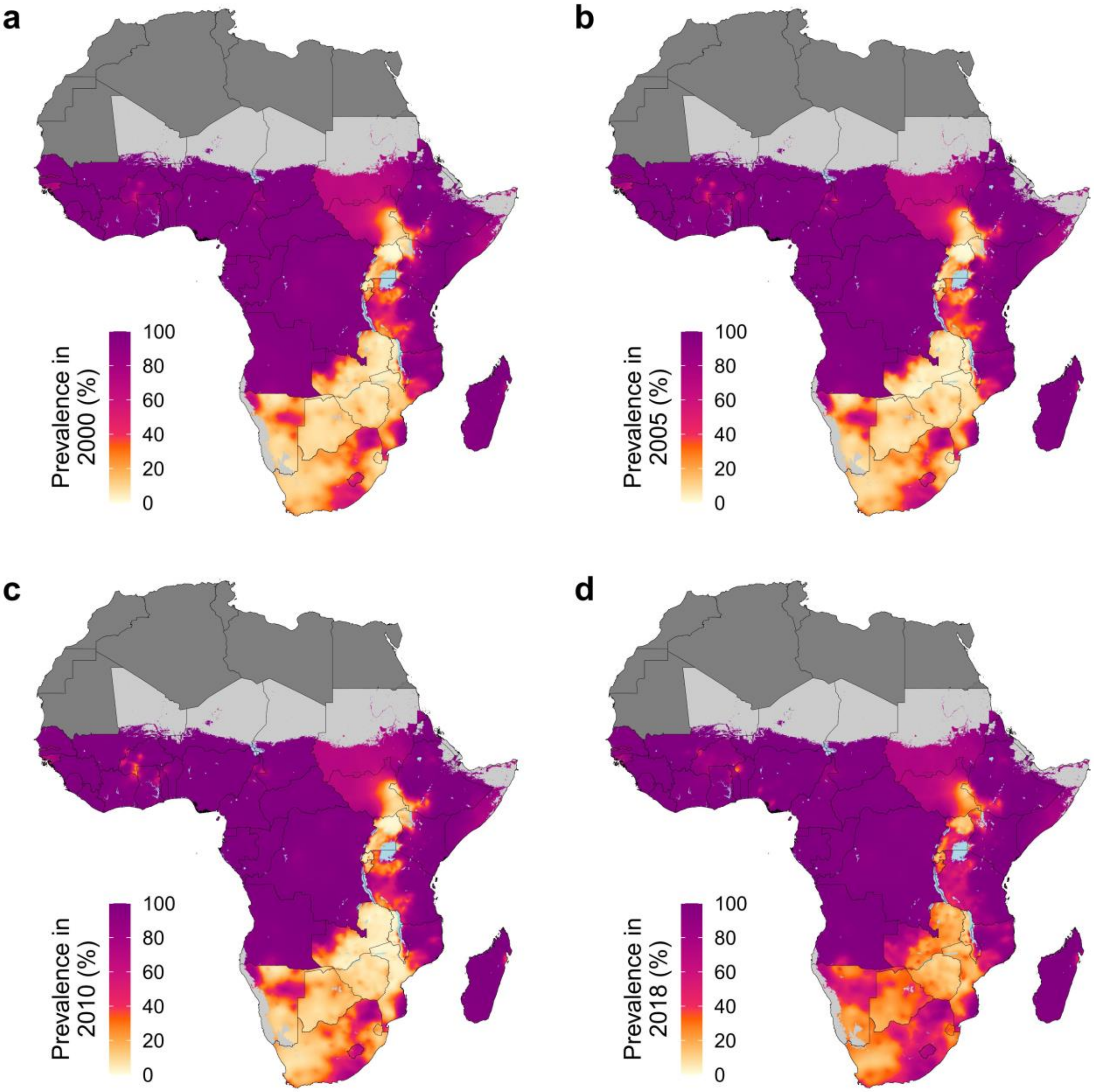

Figure S1: Prevalence of male circumcision. Prevalence of male circumcision, including medical or traditional circumcision, among males ages 15–49 years at the 5 x 5-km grid cell level in (a) 2000, (b) 2005, (c) 2010, and (d) 2018. Maps reflect administrative boundaries, land cover, lakes, and population; grid cells with fewer than ten people per 1 x 1-km and classified as “barren or sparsely vegetated” are colored in light grey. Countries colored in dark grey were not included in the analysis.

Figure S2: Prevalence of signs and symptoms of sexually transmitted infections

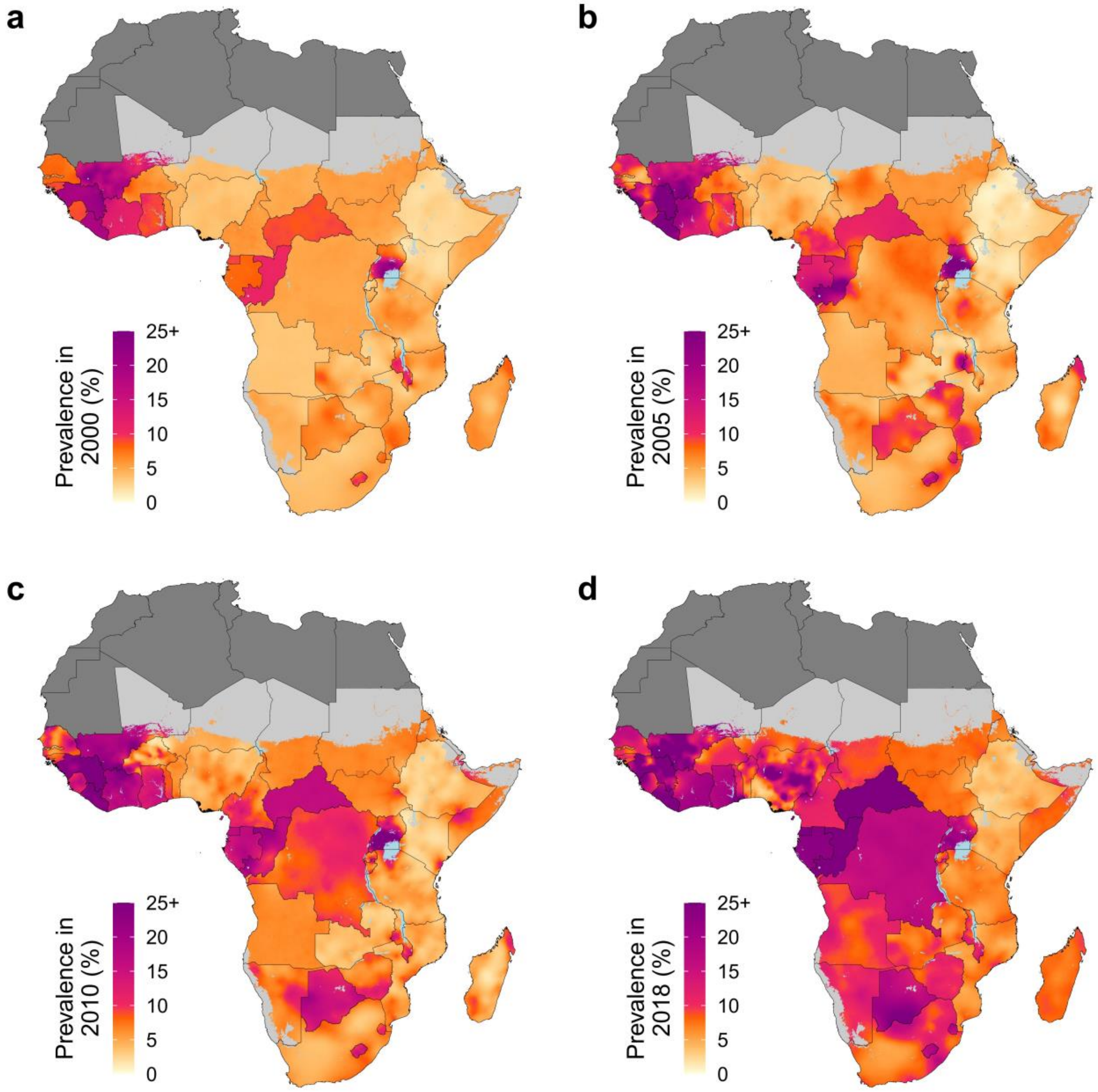

Figure S2: Prevalence of signs and symptoms of sexually transmitted infections. Prevalence of self-reported STI symptoms (genital discharge and/or genital ulcer/sore) in the last 12 months among adults ages 15–49 years at the 5 x 5-km grid cell level in (a) 2000, (b) 2005, (c) 2010, and (d) 2018. Maps reflect administrative boundaries, land cover, lakes, and population; grid cells with fewer than ten people per 1 x 1-km and classified as “barren or sparsely vegetated” are colored in light grey. Countries colored in dark grey were not included in the analysis.

Figure S3: Prevalence of marriage or living as married

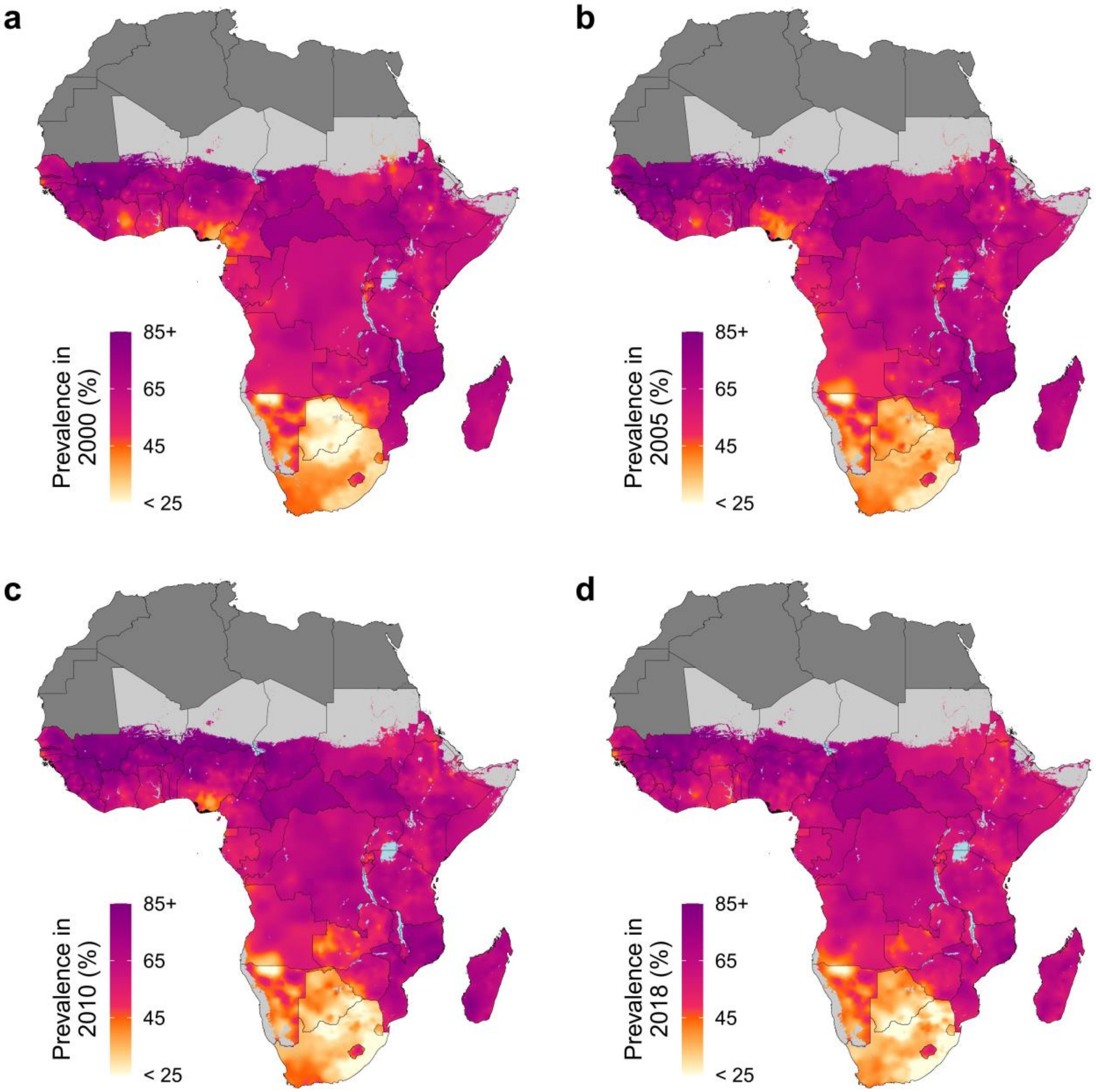

Figure S3: Prevalence of marriage or living as married. Prevalence of marriage or living as married among adults ages 15–49 years at the 5 x 5-km grid cell level in (a) 2000, (b) 2005, (c) 2010, and (d) 2018. Maps reflect administrative boundaries, land cover, lakes, and population; grid cells with fewer than ten people per 1 x 1-km and classified as "barren or sparsely vegetated" are colored in light grey. Countries colored in dark grey were not included in the analysis.

Figure S4: Prevalence of partner living elsewhere among females

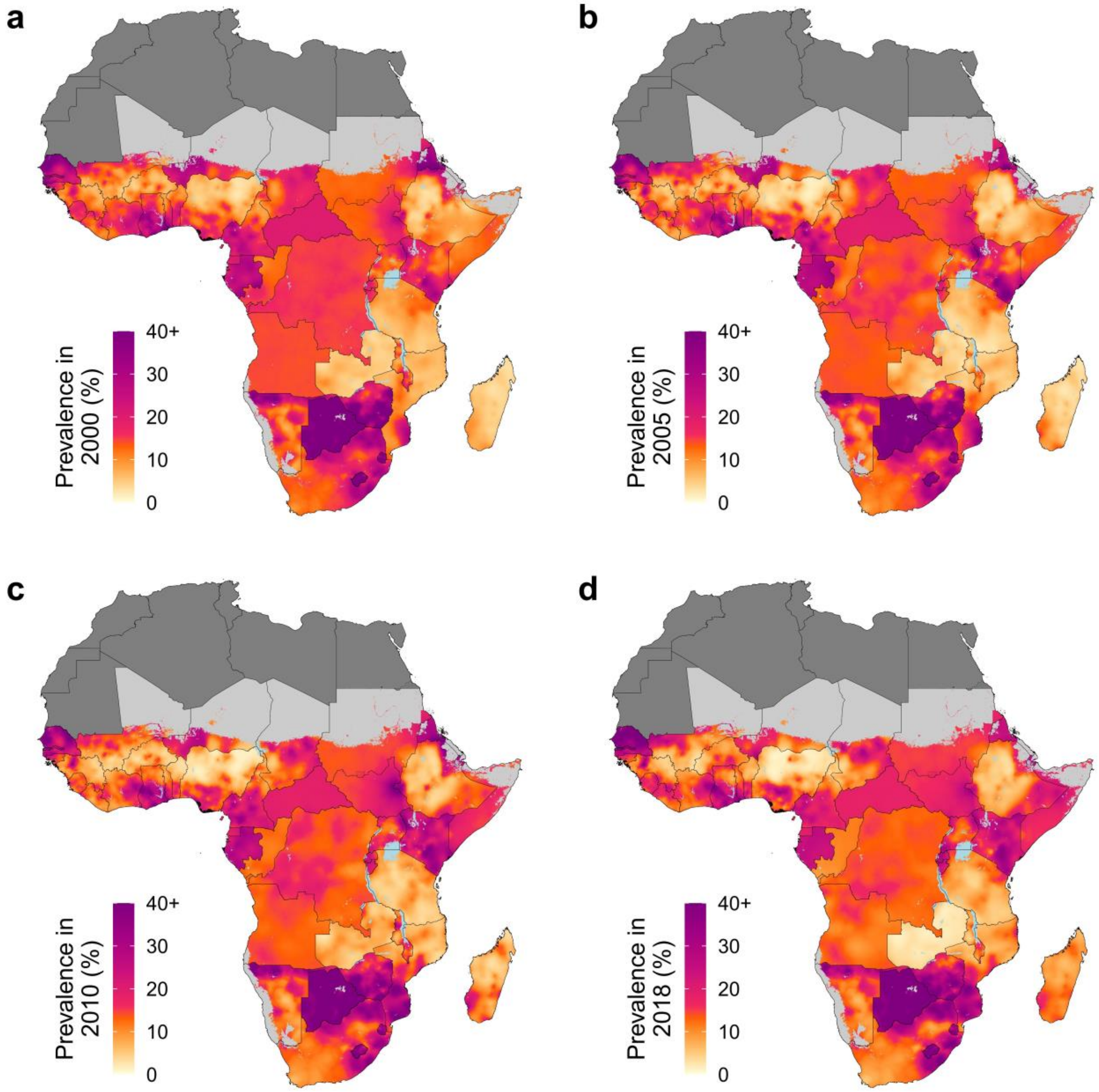

Figure S4: Prevalence of partner living elsewhere among women. Prevalence of partner living elsewhere among females ages 15–49 years at the 5 x 5-km grid cell level in (a) 2000, (b) 2005, (c) 2010, and (d) 2018. Maps reflect administrative boundaries, land cover, lakes, and population; grid cells with fewer than ten people per 1 x 1-km and classified as “barren or sparsely vegetated” are colored in light grey. Countries colored in dark grey were not included in the analysis.

Figure S5: Prevalence of condom use during most recent sexual encounter

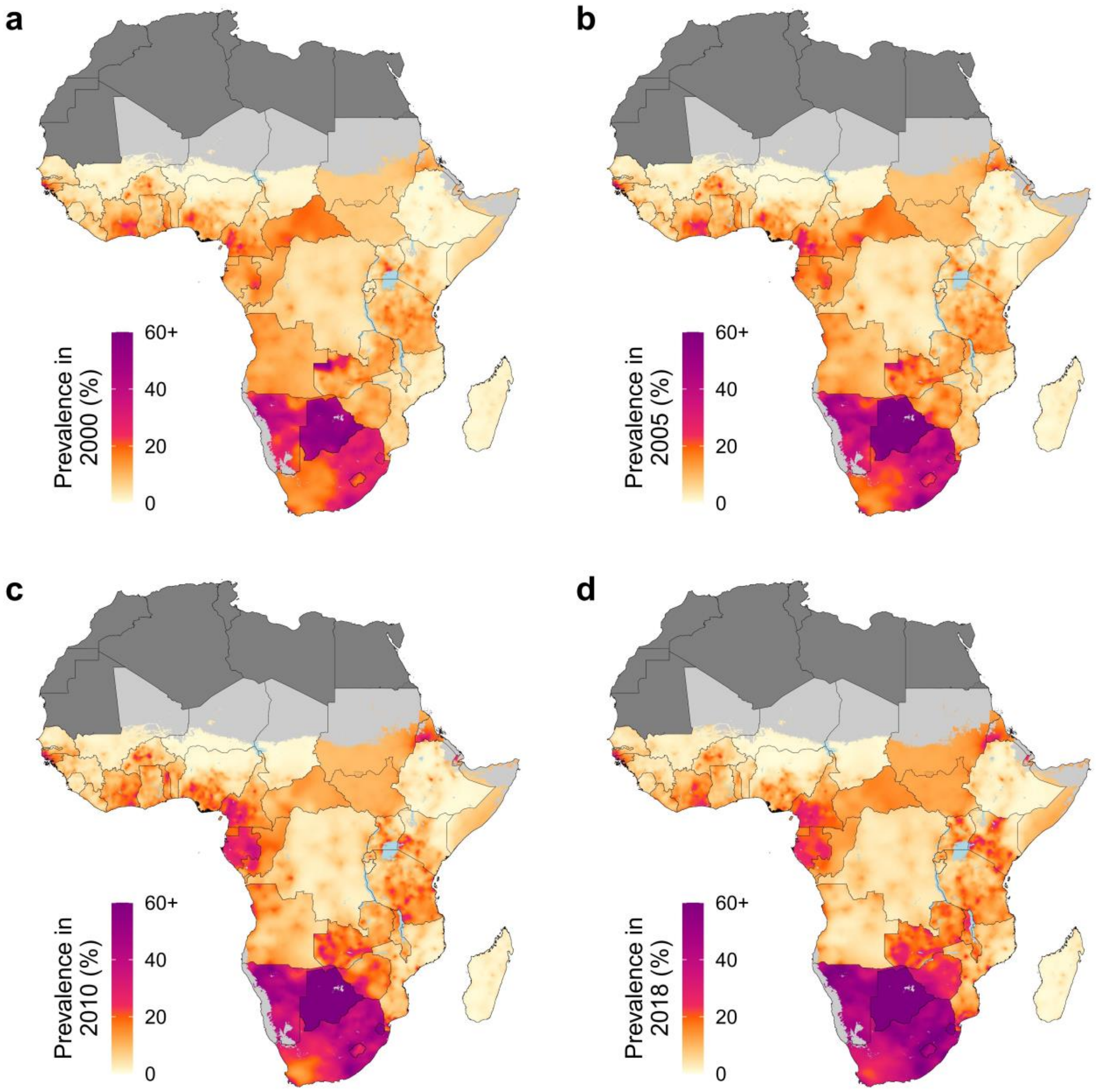

Figure S5: Prevalence of condom use during most recent sexual encounter. Prevalence of condom use during most recent sexual encounter within the last 12 months among adults ages 15–49 years at the 5 x 5-km grid cell level in (a) 2000, (b) 2005, (c) 2010, and (d) 2018. Maps reflect administrative boundaries, land cover, lakes, and population; grid cells with fewer than ten people per 1 x 1-km and classified as "barren or sparsely vegetated" are colored in light grey. Countries colored in dark grey were not included in the analysis.

Figure S6: Prevalence of sexual activity among young females

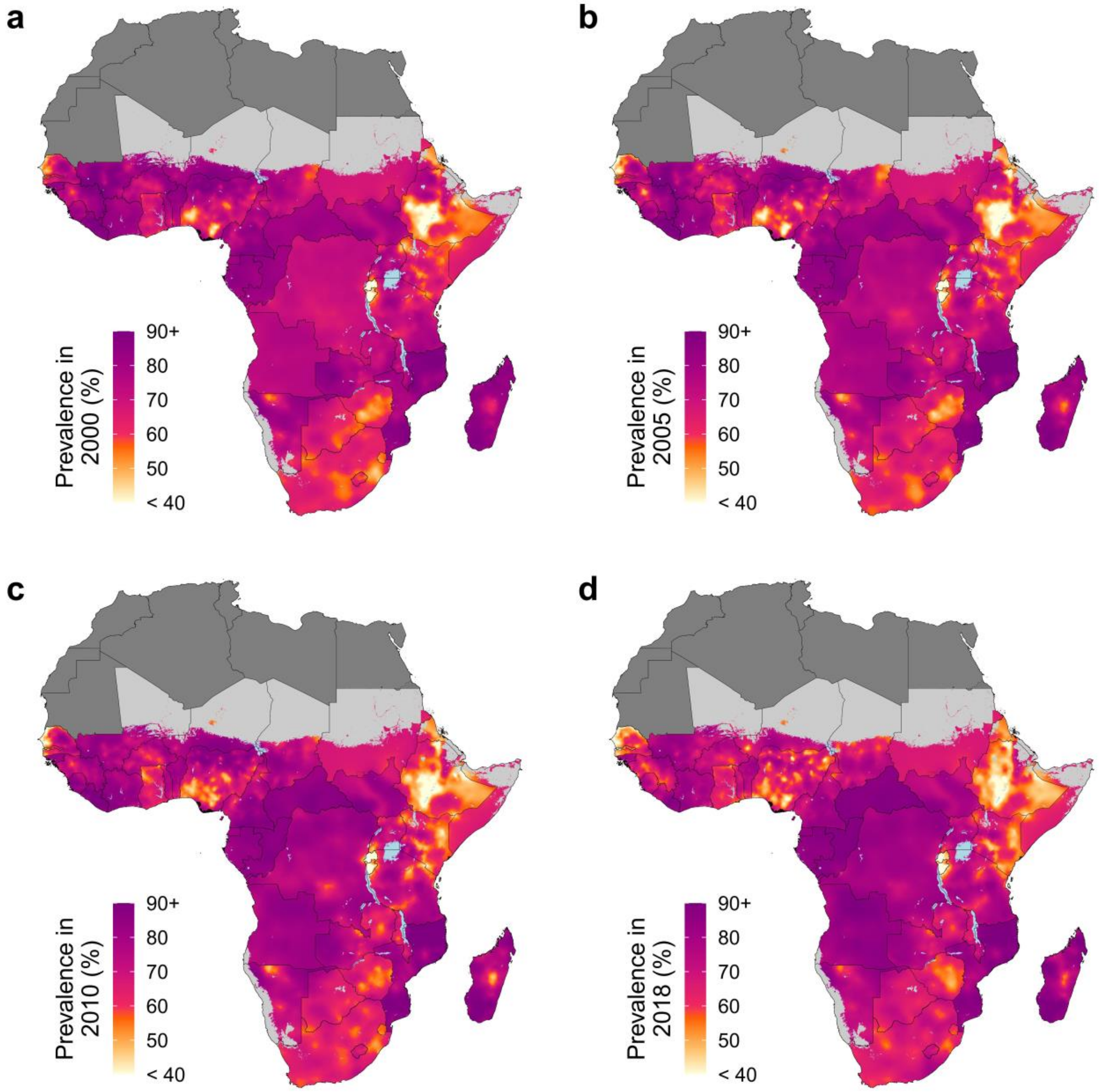

Figure S6: Prevalence of sexual activity among young females. Prevalence of sexual activity among females ages 15–24 years at the 5 x 5-km grid cell level in (a) 2000, (b) 2005, (c) 2010, and (d) 2018. Maps reflect administrative boundaries, land cover, lakes, and population; grid cells with fewer than ten people per 1 x 1-km and classified as “barren or sparsely vegetated” are colored in light grey. Countries colored in dark grey were not included in the analysis.

Figure S7: Prevalence of multiple partners among males in the past year

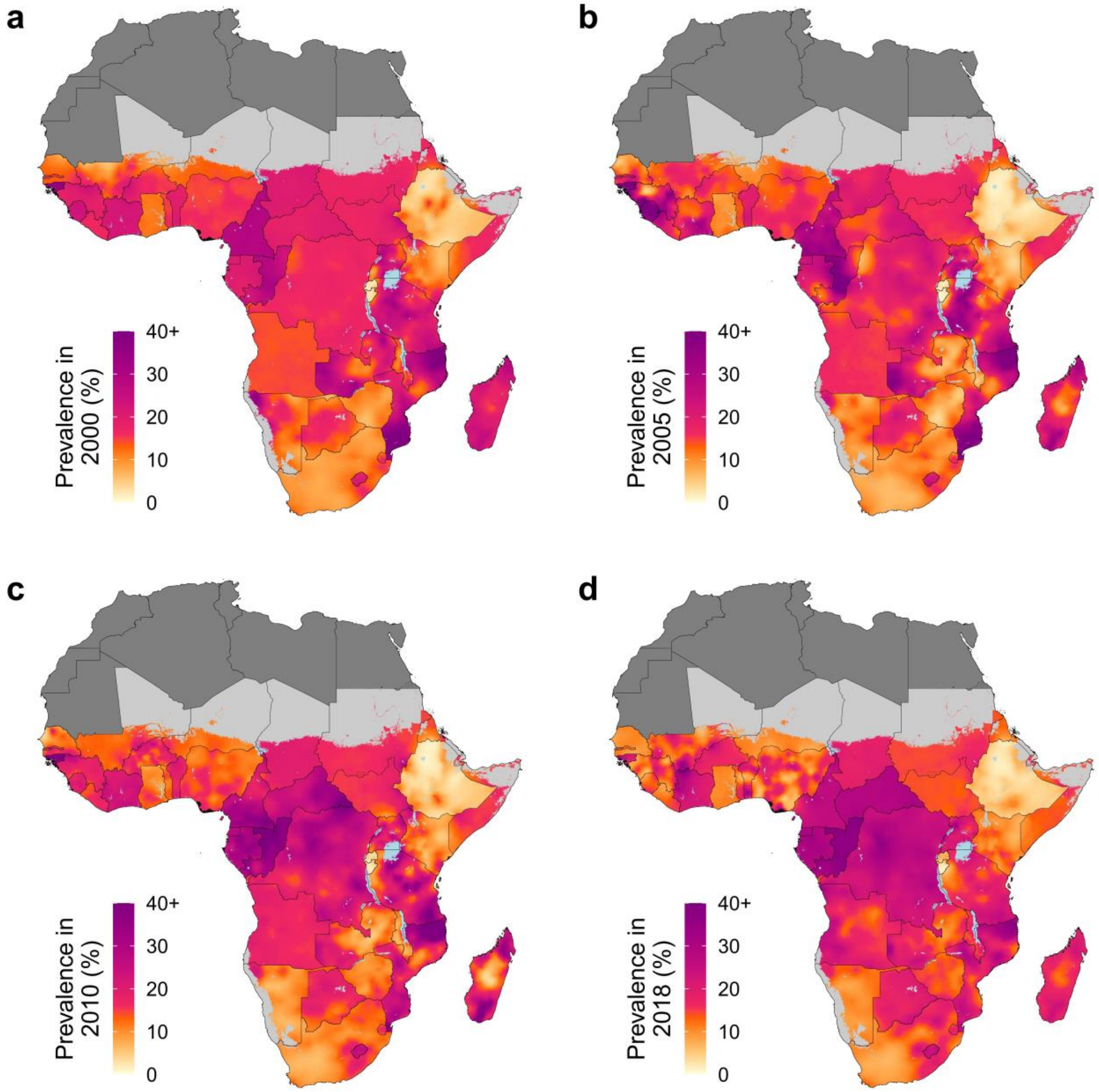

Figure S7: Prevalence of multiple partners among males in the past year. Prevalence of multiple partners among males ages 15–49 years at the 5 x 5-km grid cell level in (a) 2000, (b) 2005, (c) 2010, and (d) 2018. Maps reflect administrative boundaries, land cover, lakes, and population; grid cells with fewer than ten people per 1 x 1-km and classified as “barren or sparsely vegetated” are colored in light grey. Countries colored in dark grey were not included in the analysis.

Figure S8: Prevalence of multiple partners among females in the past year

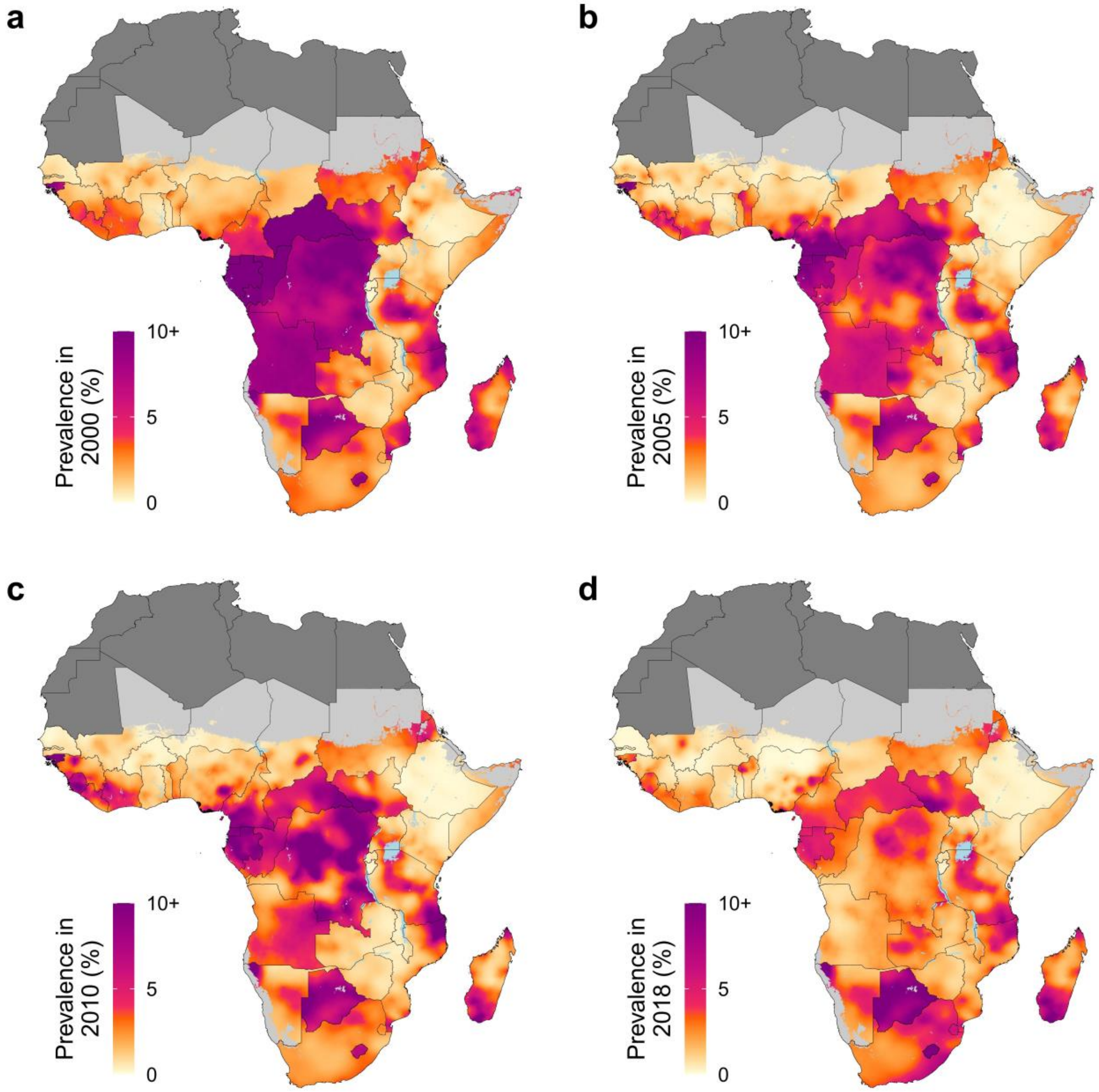

Figure S8: Prevalence of multiple partners among females in the past year. Prevalence of multiple partners among females ages 15–49 years at the 5 x 5-km grid cell level in (a) 2000, (b) 2005, (c) 2010, and (d) 2018. Maps reflect administrative boundaries, land cover, lakes, and population; grid cells with fewer than ten people per 1 x 1-km and classified as "barren or sparsely vegetated" are colored in light grey. Countries colored in dark grey were not included in the analysis.

Figure S9: HIV prevalence predictions from the boosted regression tree model

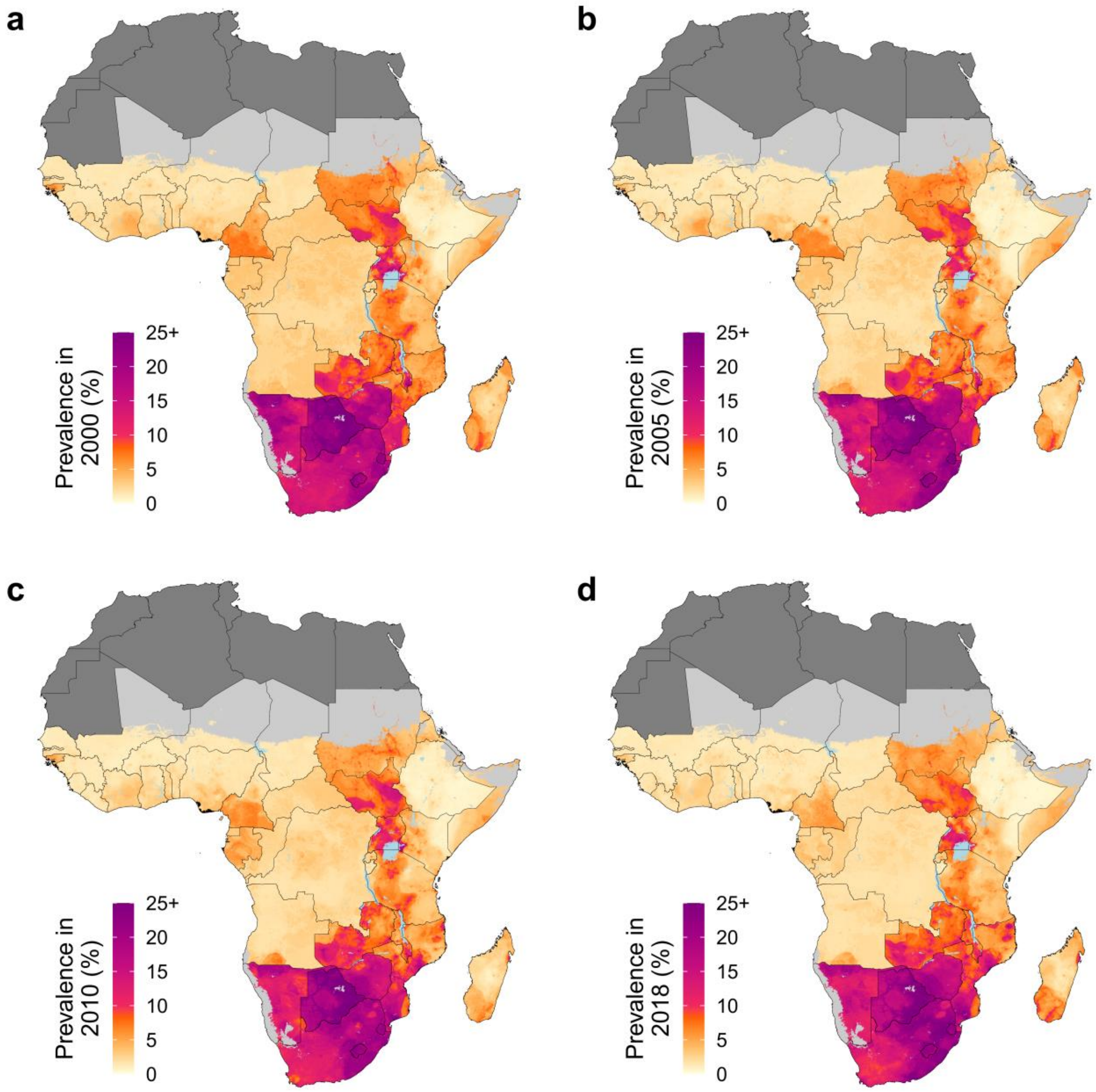

Figure S9: HIV prevalence predictions from the boosted regression tree model. HIV prevalence among adults ages 15–49 years at the 5 x 5-km grid cell level in (a) 2000, (b) 2005, (c) 2010, and (d) 2018 as estimated in the boosted regression tree model. Maps reflect administrative boundaries, land cover, lakes, and population; grid cells with fewer than ten people per 1 x 1-km and classified as "barren or sparsely vegetated" are colored in light grey. Countries colored in dark grey were not included in the analysis.

Figure S10: HIV prevalence predictions from the generalized additive model

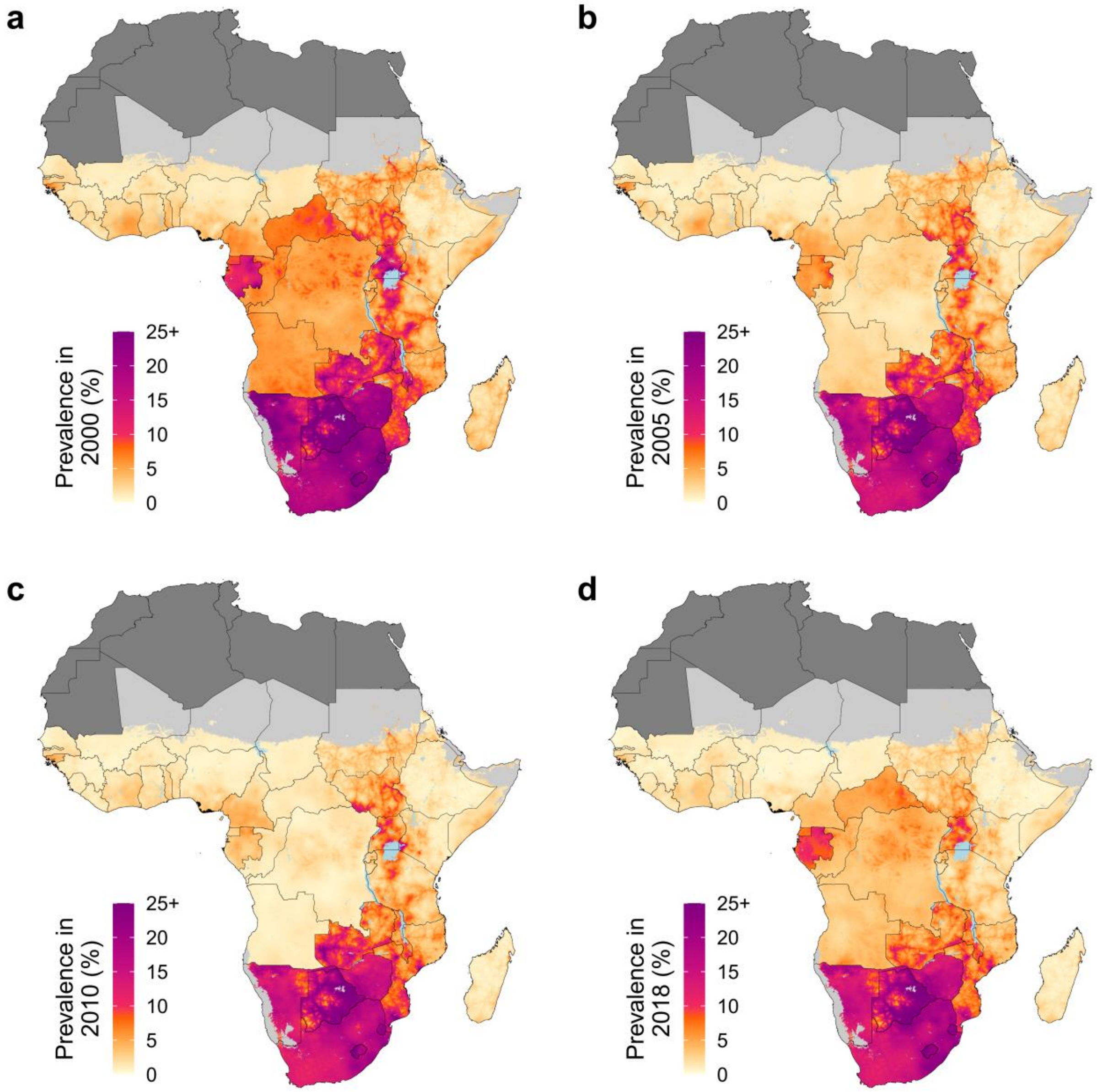

Figure S10: HIV prevalence predictions from the generalized additive model. HIV prevalence among adults ages 15–49 years at the 5 x 5-km grid cell level in (a) 2000, (b) 2005, (c) 2010, and (d) 2018 as estimated in the generalized additive model. Maps reflect administrative boundaries, land cover, lakes, and population; grid cells with fewer than ten people per 1 x 1-km and classified as "barren or sparsely vegetated" are colored in light grey. Countries colored in dark grey were not included in the analysis.

Figure S11: HIV prevalence predictions from the lasso regression model

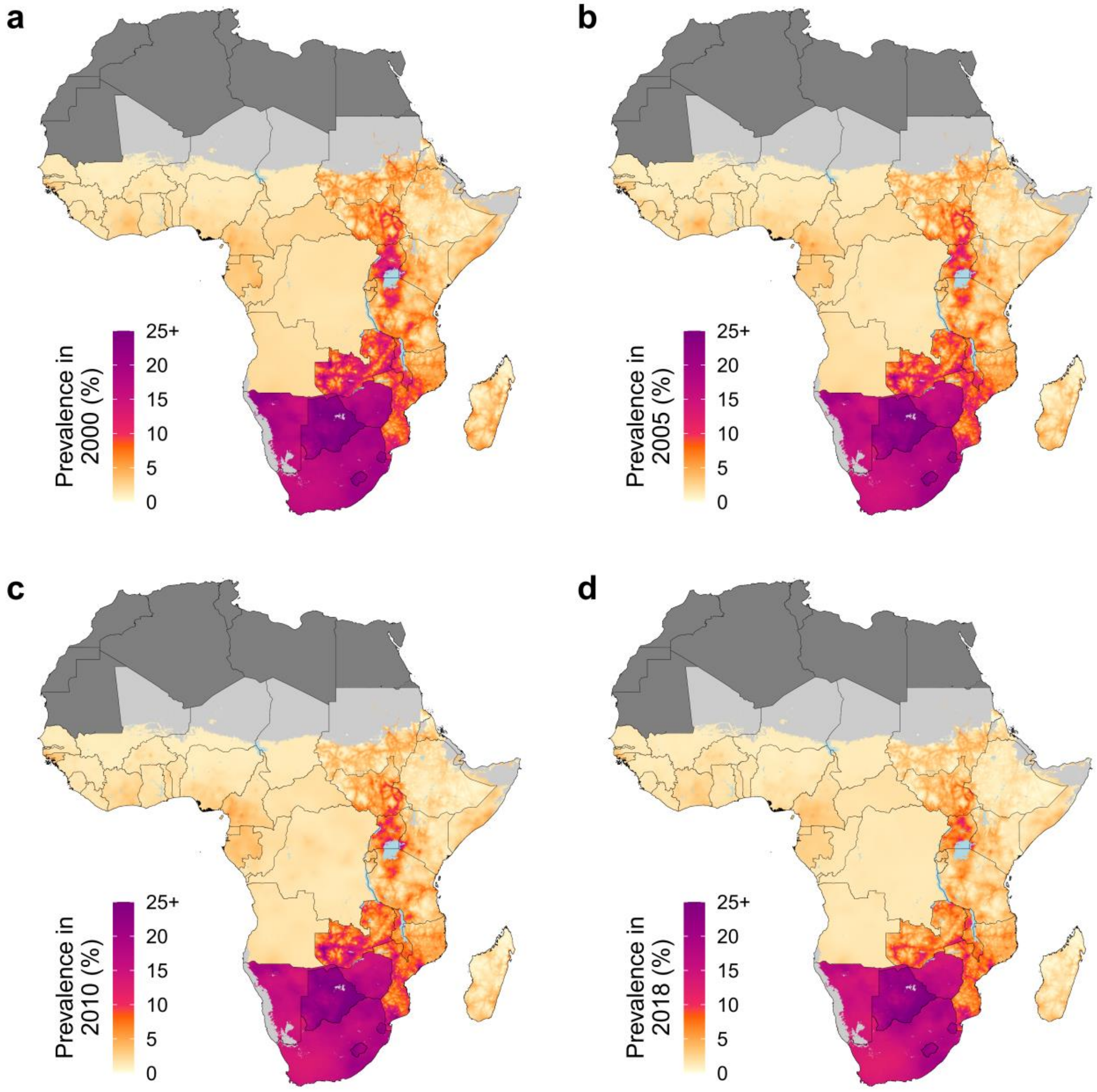

Figure S11: HIV prevalence predictions from the lasso regression model. HIV prevalence among adults ages 15–49 years at the 5 x 5-km grid cell level in **(a)** 2000, **(b)** 2005, **(c)** 2010, and **(d)** 2018 as estimated in the lasso regression model. Maps reflect administrative boundaries, land cover, lakes, and population; grid cells with fewer than ten people per 1 x 1-km and classified as “barren or sparsely vegetated” are colored in light grey. Countries colored in dark grey were not included in the analysis.

Figure S12: Modeling regions

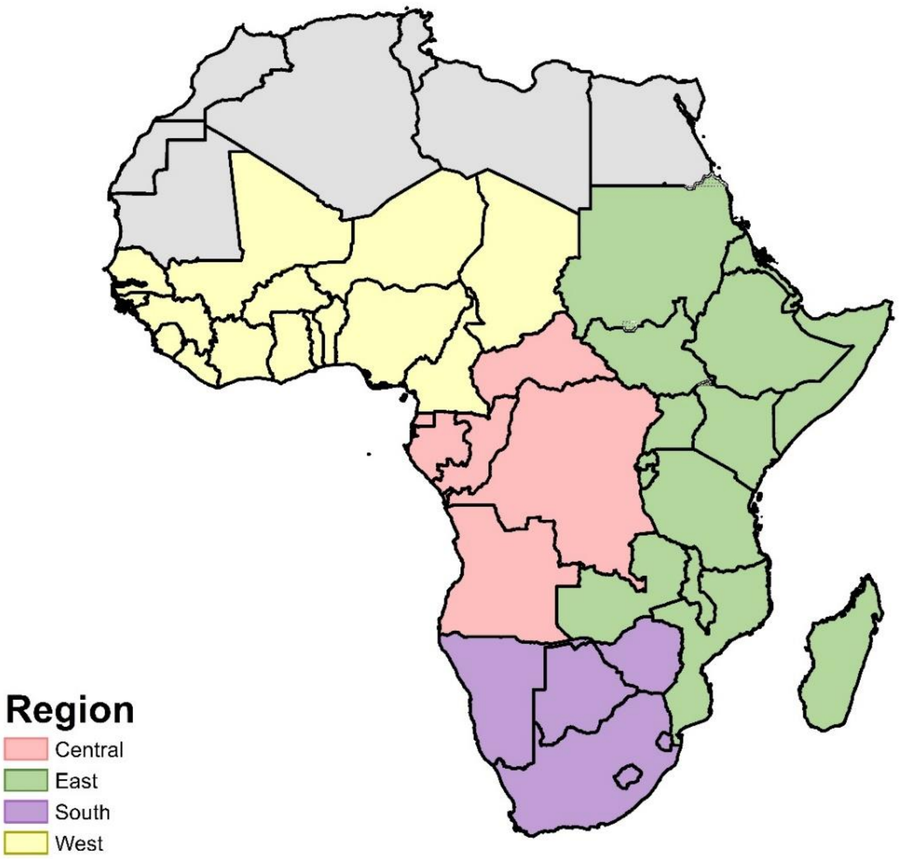

Figure S12: Modeling regions. Modeling regions were based on the four Global Burden of Disease Study (GBD) regions in sub-Saharan Africa: Central, East, South, and West. We removed Cape Verde, Comoros, São Tomé and Príncipe, and Mauritania from our analysis due to data missingness. Countries coloured in grey were not included in the analysis.

Figure S13: Age- and sex-specific vs. adult prevalence modeling

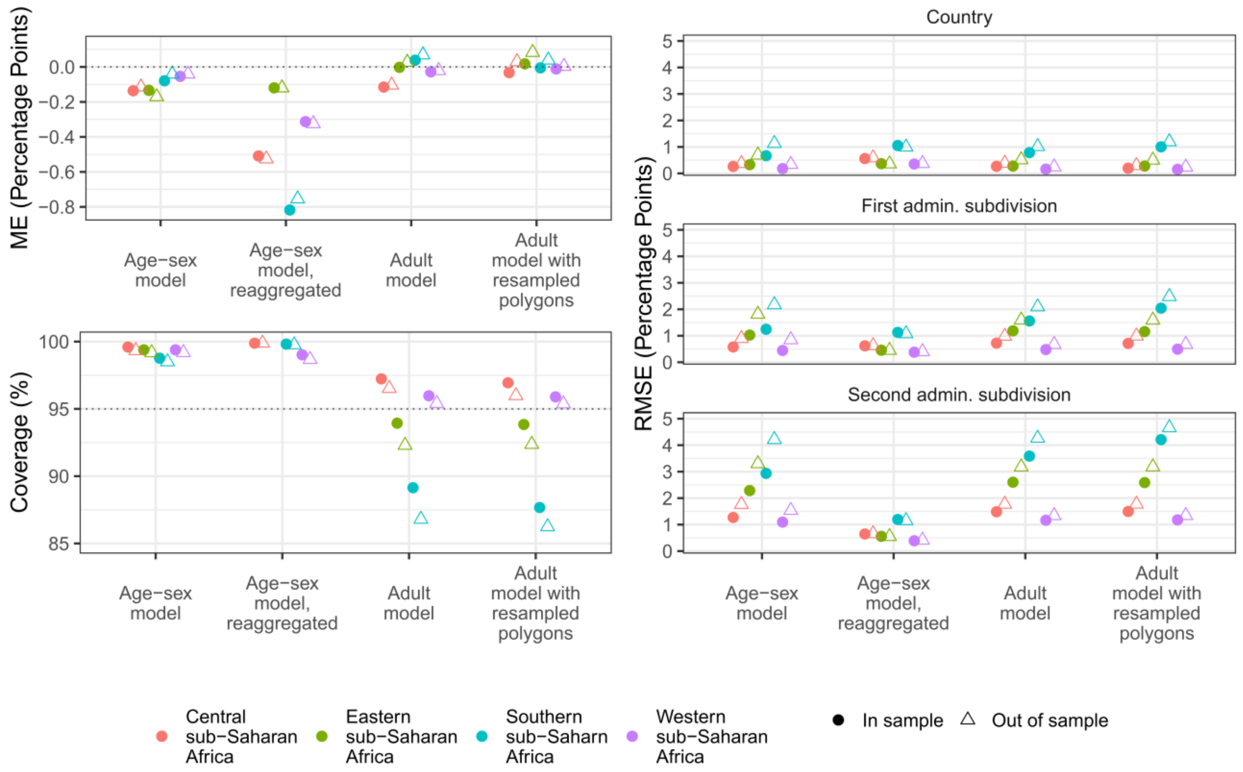

Figure S13: Age- and sex-specific vs. adult prevalence modeling. Mean error (ME; top left), 95% prediction interval coverage ('coverage'; bottom left), and root-mean-square error by administrative level of aggregation (RMSE; right) for comparing the results of the final age- and sex-specific model, the results of the same model re-aggregated to the adult level, the results of HIV prevalence modeled at the adult level, and the results of HIV prevalence modeled at the adult level, using polygon resampling techniques. Results are presented by modeling region (indicated by color) and for in- and out-of-sample results (indicated by shape). Note that for the Central sub-Saharan Africa "Adult model", out-of-sample estimates were based on the results of four folds rather than five, due to non-convergence in one out-of-sample model.

Figure S14: Data sensitivity

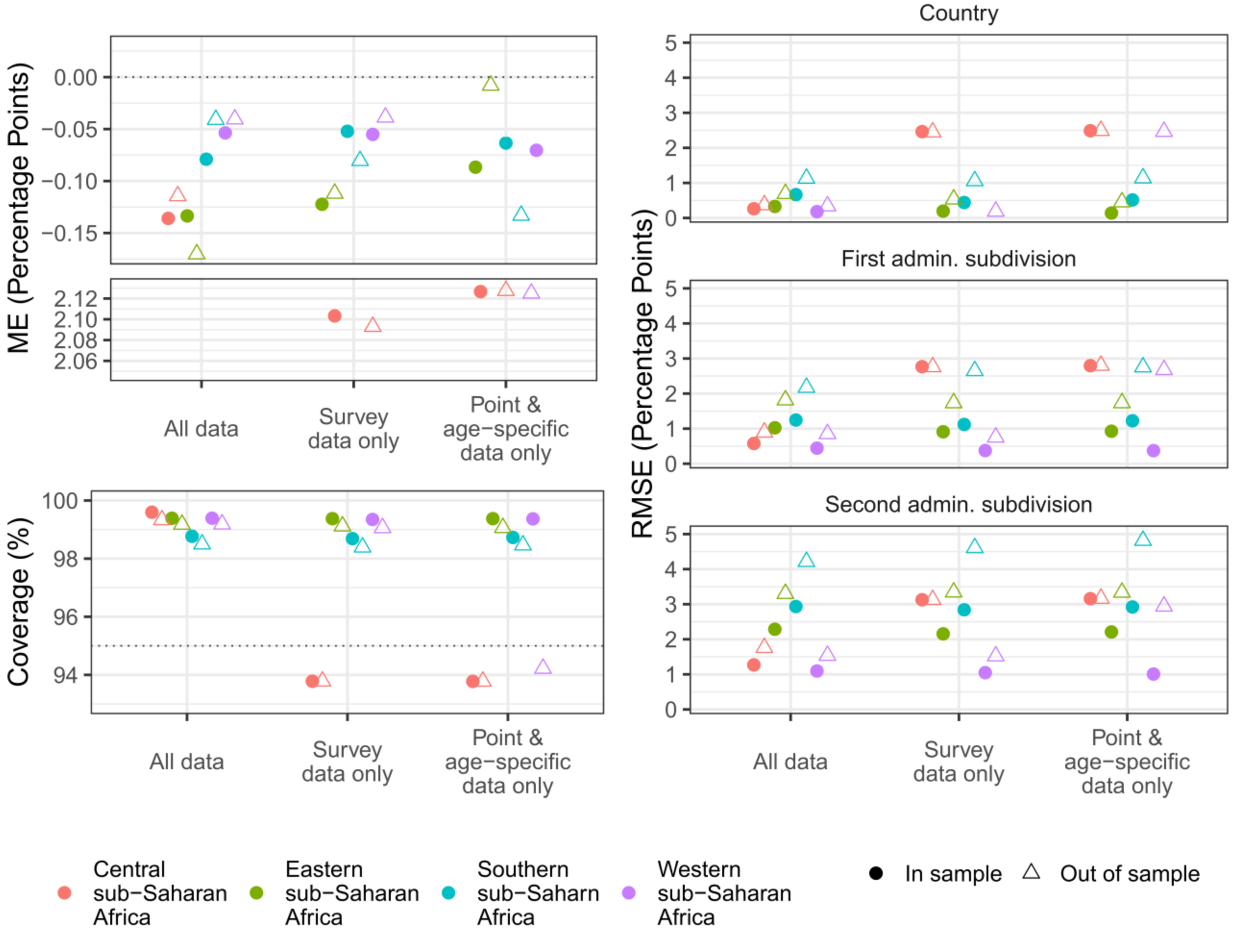

Figure S14: Data sensitivity. Mean error (ME; top left), 95% prediction interval coverage ('coverage'; bottom left), and root-mean-square error by administrative level of aggregation (RMSE; right) for comparing the results of the final model with all data included, with only location- and age-specific data included, and with only survey data included. Results are presented by modeling region (indicated by color) and for in- and out-of-sample results (indicated by shape).

Figure S15: Model specification validation

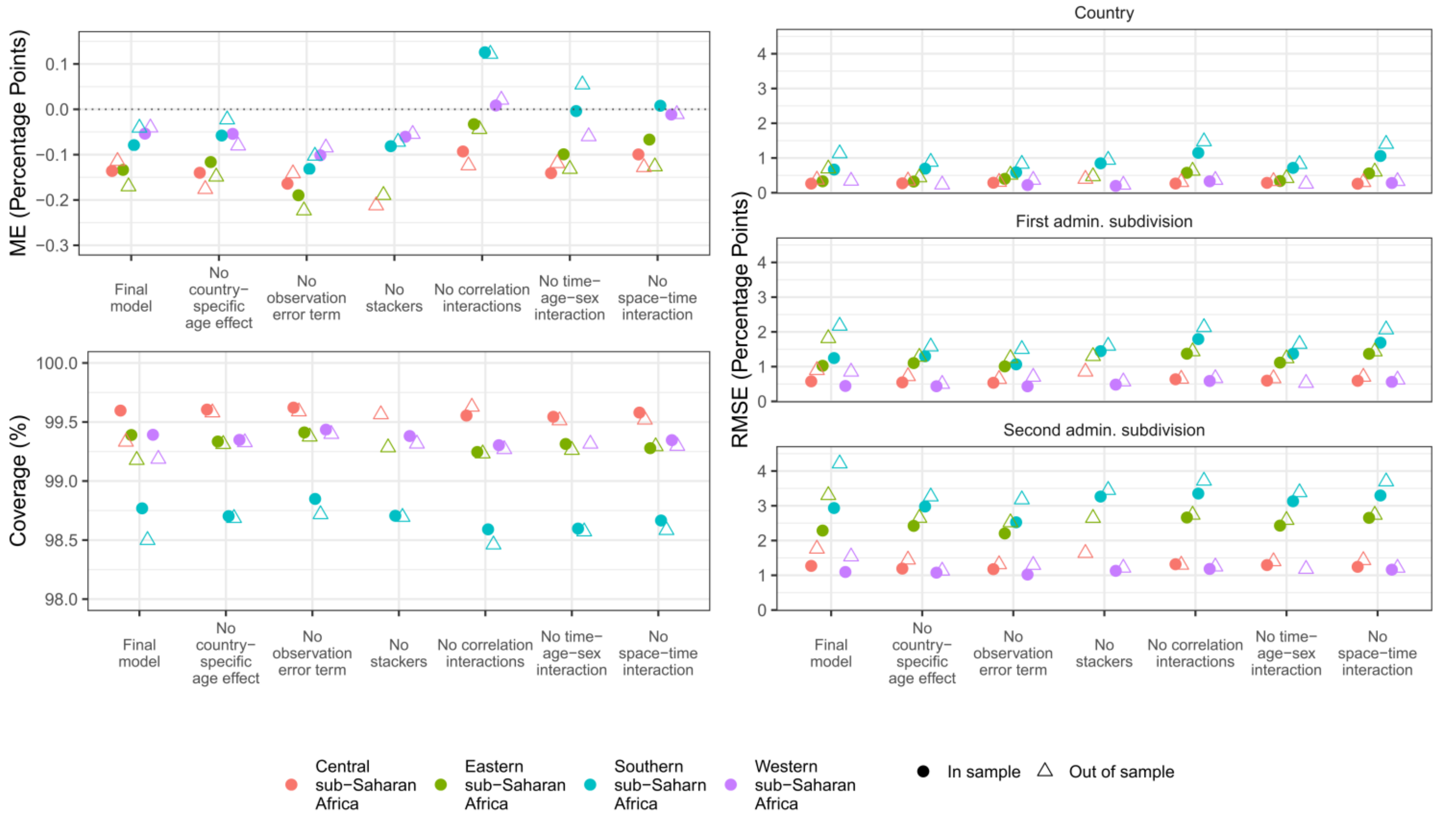

Figure S15: Model specification validation. Mean error (ME; top left), 95% prediction interval coverage ('coverage'; bottom left), and root-mean-square error by administrative level of aggregation (RMSE; right) comparing the results of the final model configuration with that of models with individual terms missing. Results are presented by modeling region (indicated by color) and for in- and out-of-sample results (indicated by shape). Note that for the Central sub-Saharan Africa "No stackers" model, no in-sample results are presented, and out-of-sample estimates were based on the results of three folds rather than five, due to non-convergence in the in-sample model and two out-of-sample models. Eastern sub-Saharan Africa also experienced non-convergence in one out-of-sample "No stackers" model, as well as in the in-sample "No observation error term" model.

Figure S16: Modeled and re-aggregated adult prevalence comparison

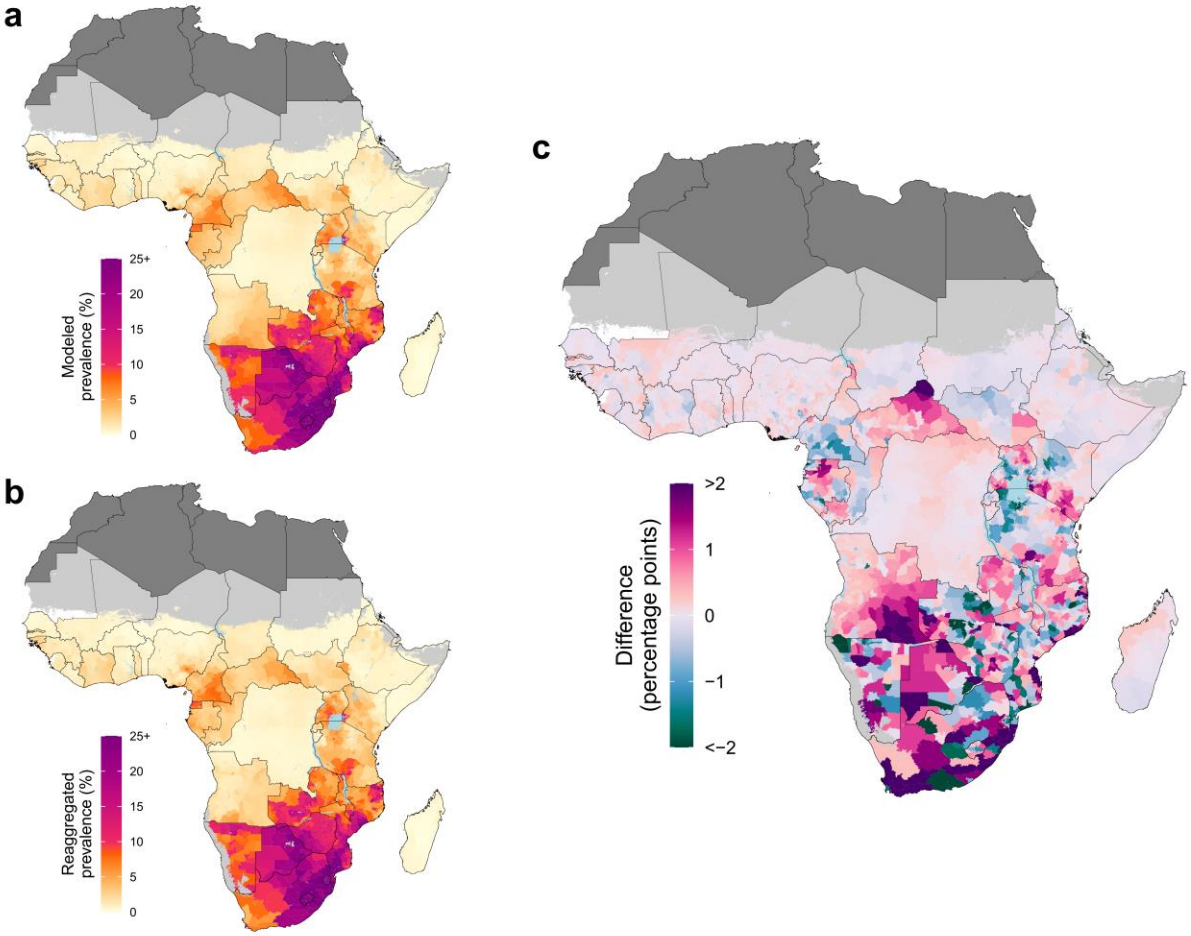

Figure S16: Modeled and re-aggregated adult prevalence comparison. A comparison of HIV prevalence estimates at the second administrative level in 2018 for adults 15–49 years when modeled **(a)** at the adult level versus **(b)** modeled at the age- and sex-specific level, and subsequently re-aggregated for adults 15–49 years. **(c)** The difference between modeled prevalence and re-aggregated prevalence. Both modeled and re-aggregated prevalence estimates were calibrated to GBD 2019 estimates[1]. Maps reflect administrative boundaries, land cover, lakes, and population; grid cells with fewer than ten people per 1 x 1-km and classified as "barren or sparsely vegetated" are colored in light grey. Countries colored in dark grey were not included in the analysis.

1. GBD 2019 Diseases and Injuries Collaborators. Global burden of 369 diseases and injuries in 204 countries and territories, 1990–2019: a systematic analysis for the Global Burden of Disease Study 2019. Lancet. 2020; 396:1204–22.

Figure S17: HIV prevalence raking factors for males

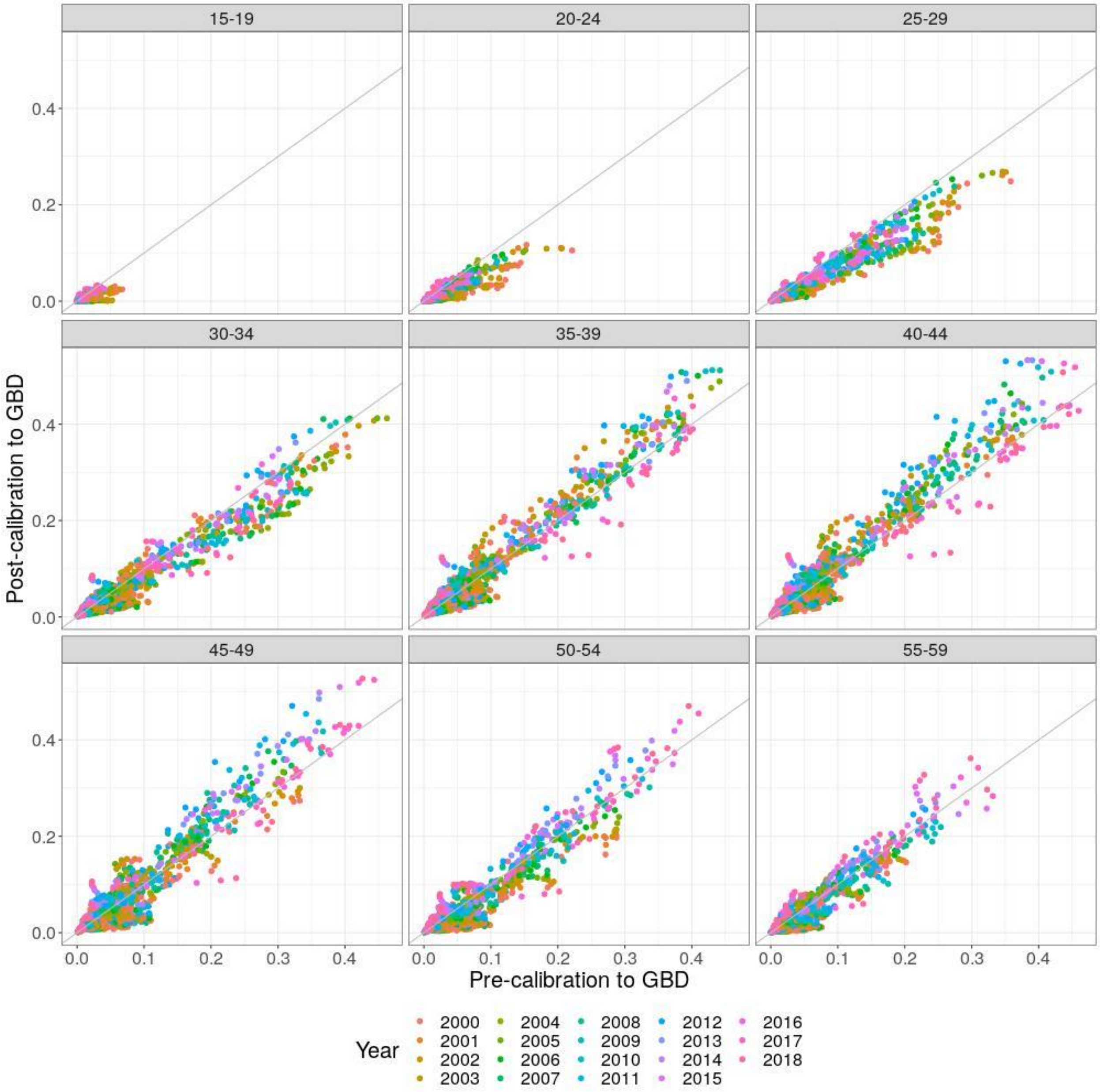

Figure S17: HIV prevalence raking factors for males. Comparison of country-level, age-specific HIV prevalence estimates for males, derived by population-weighting 5 x 5-km grid cell estimates before (x-axis) and after (y-axis) calibration to GBD 2019 by age group (panel), year (color) and country (individual point).

Figure S18: HIV prevalence raking factors for females

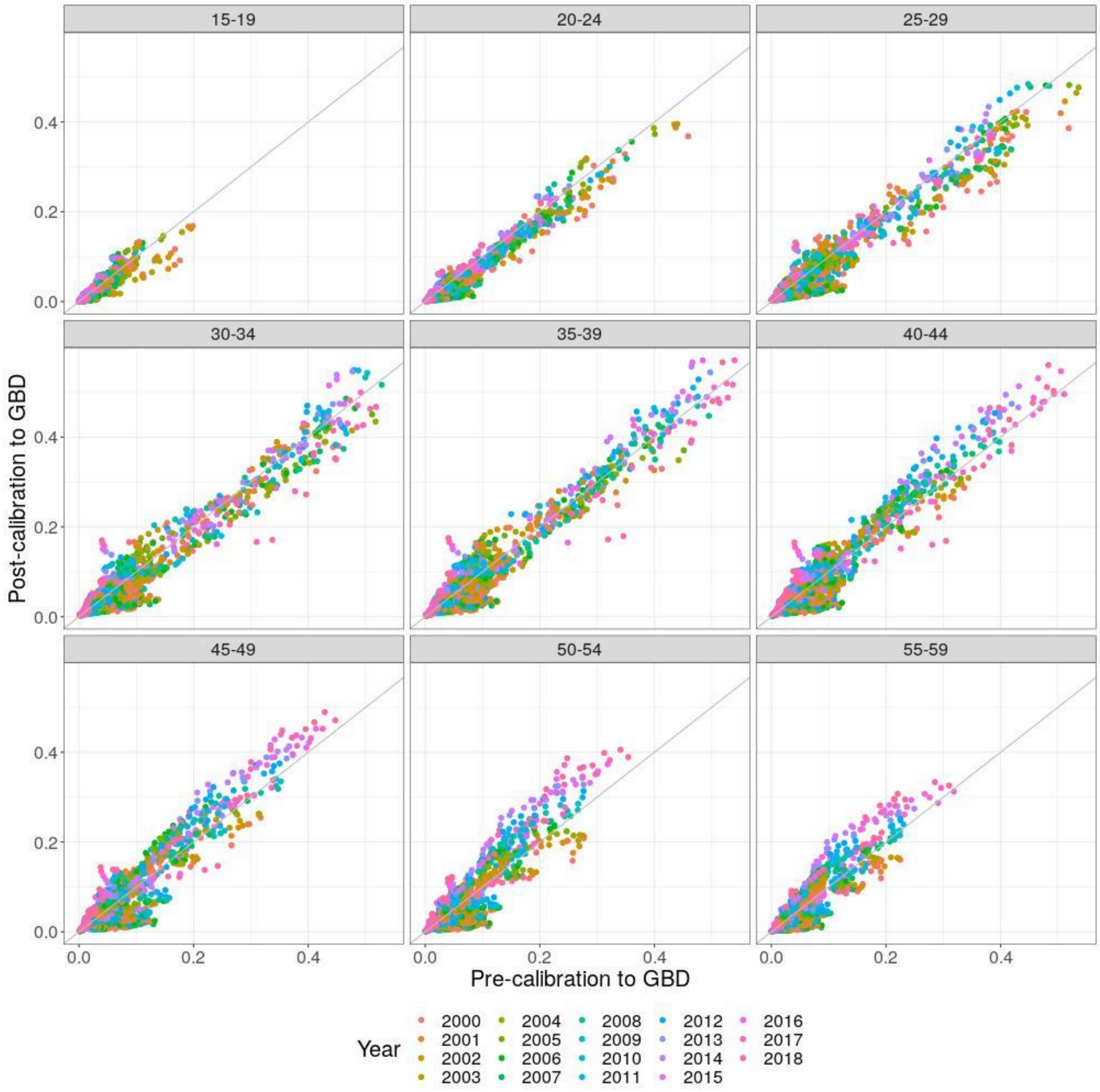

Figure S18: HIV prevalence raking factors for females. Comparison of country-level, age-specific HIV prevalence estimates for females derived by population-weighting 5 x 5-km grid cell estimates before (x-axis) and after (y-axis) calibration to GBD 2019 by age group (panel), year (color) and country (individual point).

Figure S19: Age-specific HIV prevalence in males, 2000

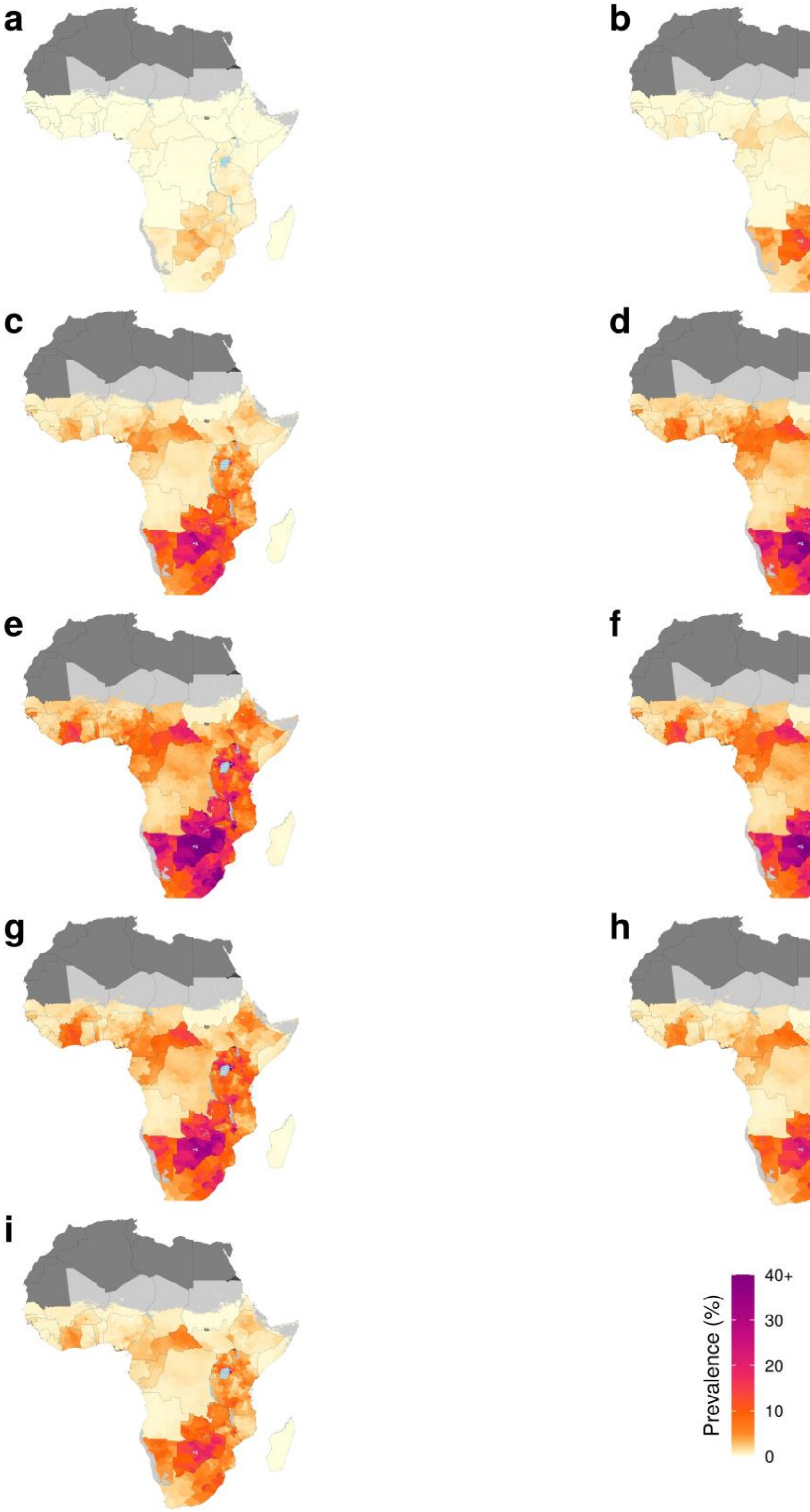

Figure S19: Age-specific HIV prevalence in males, 2000. Male HIV prevalence estimates in the year 2000, at the second administrative level, for (a) ages 15–19 years; (b) ages 20-24 years; (c) ages 25-29 years; (d)

ages 30-34 years; (e) ages 35-39 years; (f) ages 40-44 years; (g) ages 45-49 years; (h) ages 50-54 years; and (i) ages 55-59 years. Maps reflect national boundaries, land cover, lakes, and population; areas with fewer than ten people per 1 × 1 km, and classified as barren or sparsely vegetated, are colored light grey. Countries colored in dark grey were not included in the analysis.

Figure S20: Age-specific HIV prevalence in females, 2000

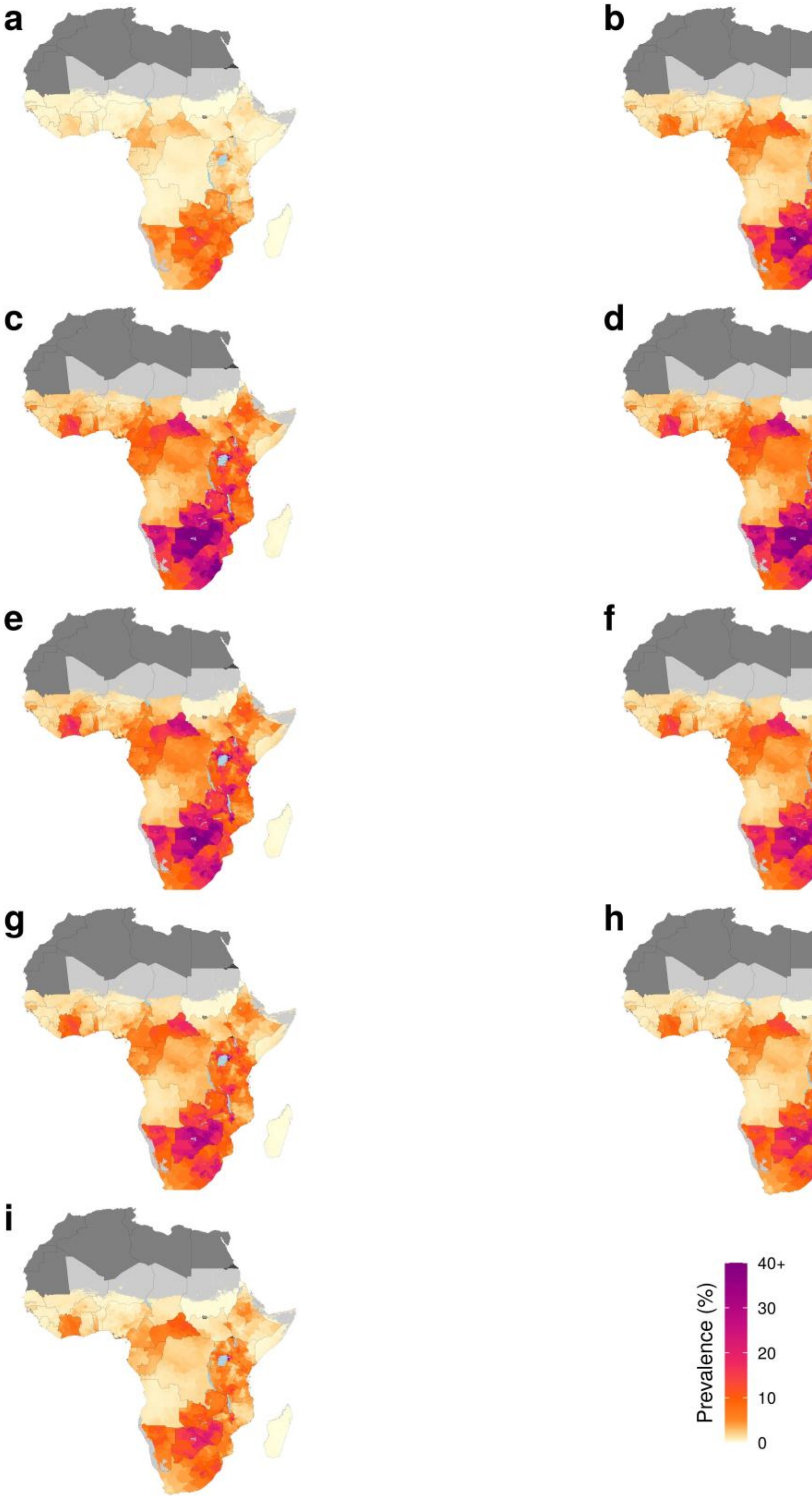

Figure S20: Age-specific HIV prevalence in females, 2000. Female HIV prevalence estimates in the year 2000, at the second administrative level, for (a) ages 15–19 years; (b) ages 20-24 years; (c) ages 25-29

years; (d) ages 30-34 years; (e) ages 35-39 years; (f) ages 40-44 years; (g) ages 45-49 years; (h) ages 50-54 years; and (i) ages 55-59 years. Maps reflect national boundaries, land cover, lakes, and population; areas with fewer than ten people per 1 × 1 km, and classified as barren or sparsely vegetated, are colored light grey. Countries colored in dark grey were not included in the analysis.

Figure S21: Age-specific HIV prevalence in males, 2005

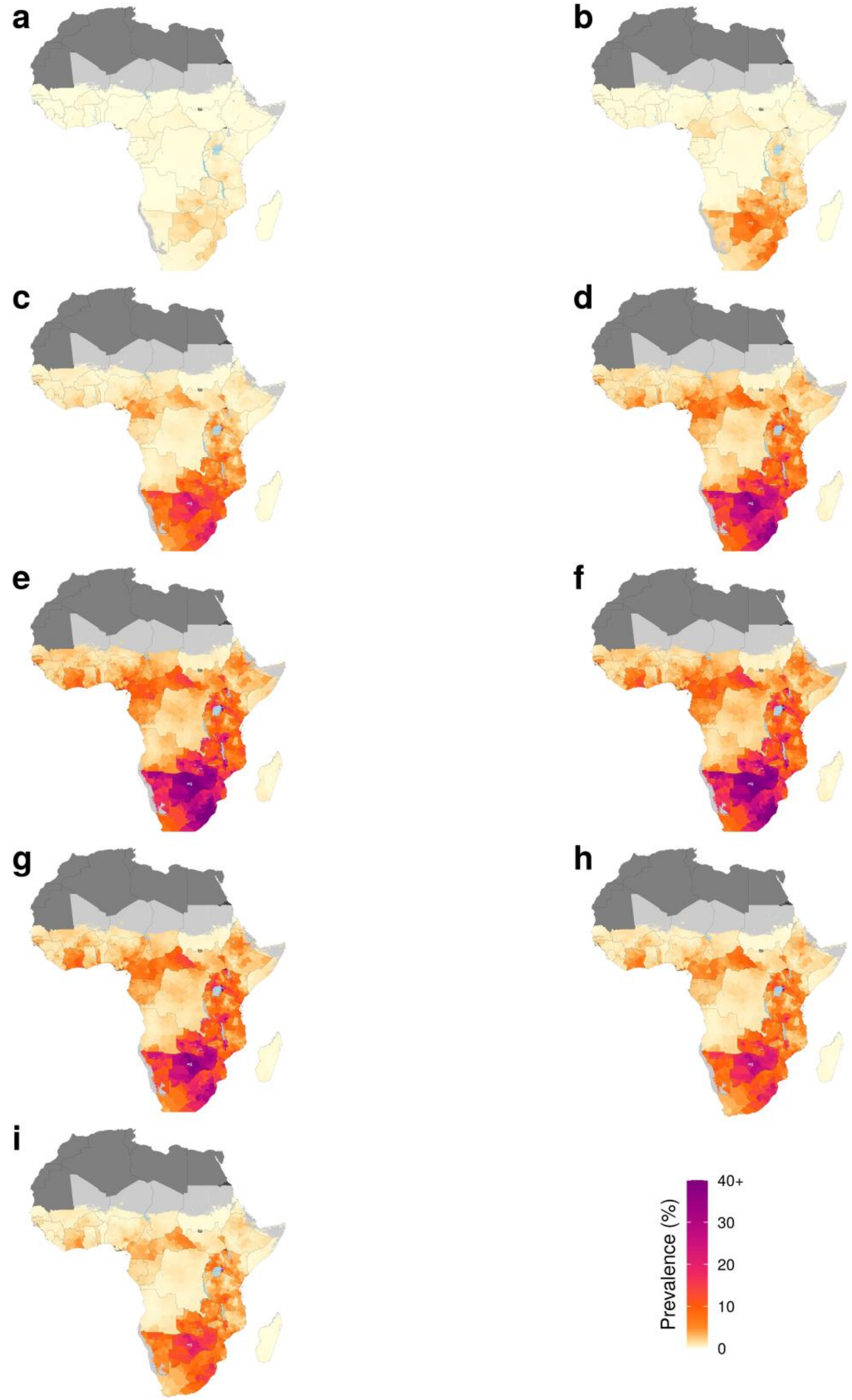

Figure S21: Age-specific HIV prevalence in males, 2005. Male HIV prevalence estimates in the year 2005, at the second administrative level, for (a) ages 15–19 years; (b) ages 20-24 years; (c) ages 25-29 years; (d) ages 30-34 years; (e) ages 35-39 years; (f) ages 40-44 years; (g) ages 45-49 years; (h) ages 50-54 years; and (i) ages 55-59 years. Maps reflect national boundaries, land cover, lakes, and population; areas with fewer than ten people per 1 × 1 km, and classified as barren or sparsely vegetated, are colored light grey. Countries colored in dark grey were not included in the analysis.

Figure S22: Age-specific HIV prevalence in females, 2005

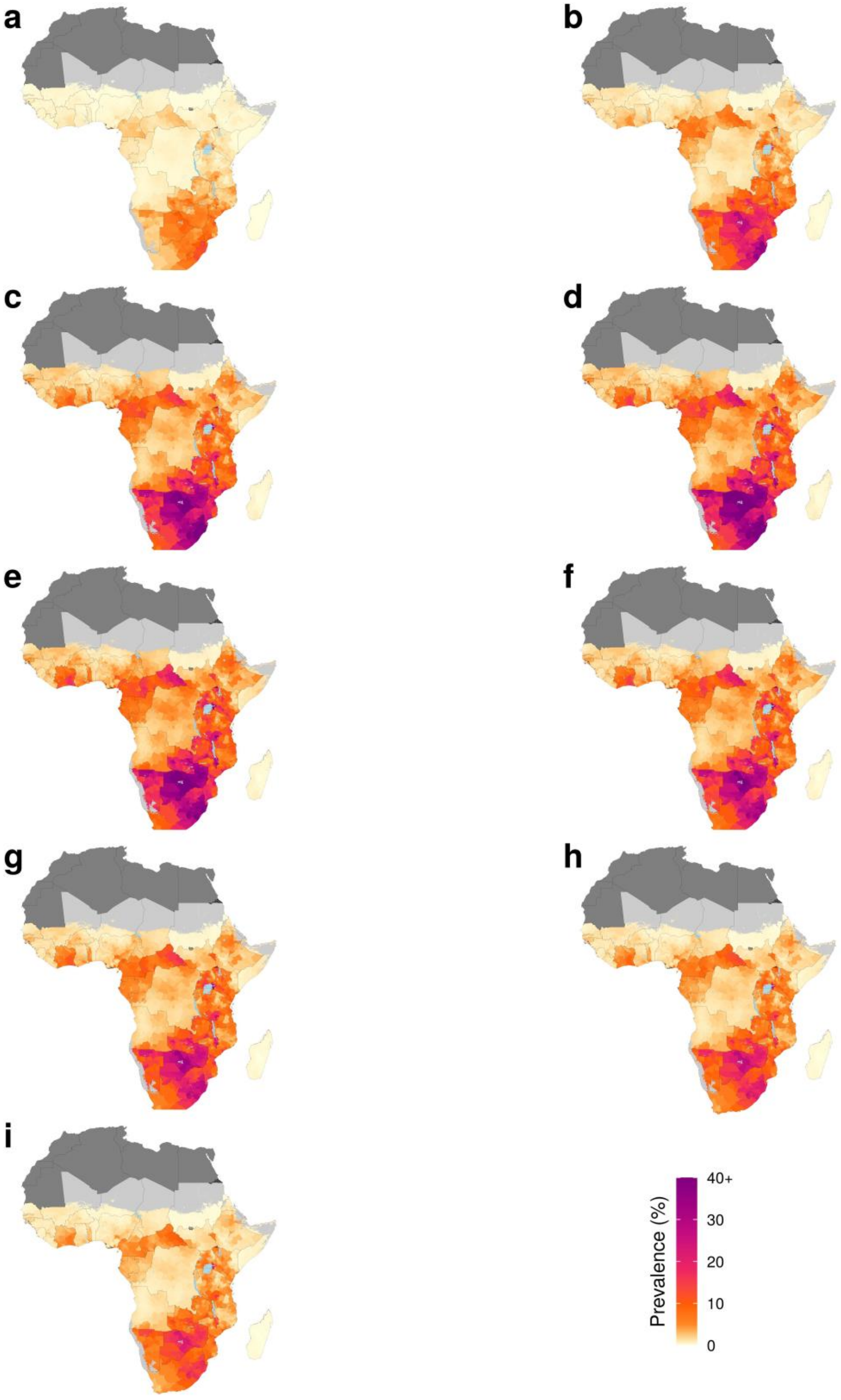

Figure S22: Age-specific HIV prevalence in females, 2005. Female HIV prevalence estimates in the year 2005, at the second administrative level, for (a) ages 15–19 years; (b) ages 20-24 years; (c) ages 25-29 years; (d) ages 30-34 years; (e) ages 35-39 years; (f) ages 40-44 years; (g) ages 45-49 years; (h) ages 50-54 years; and (i) ages 55-59 years. Maps reflect national boundaries, land cover, lakes, and population; areas with fewer than ten people per 1 × 1 km, and classified as barren or sparsely vegetated, are colored light grey. Countries colored in dark grey were not included in the analysis.

Figure S23: Age-specific HIV prevalence in males, 2010

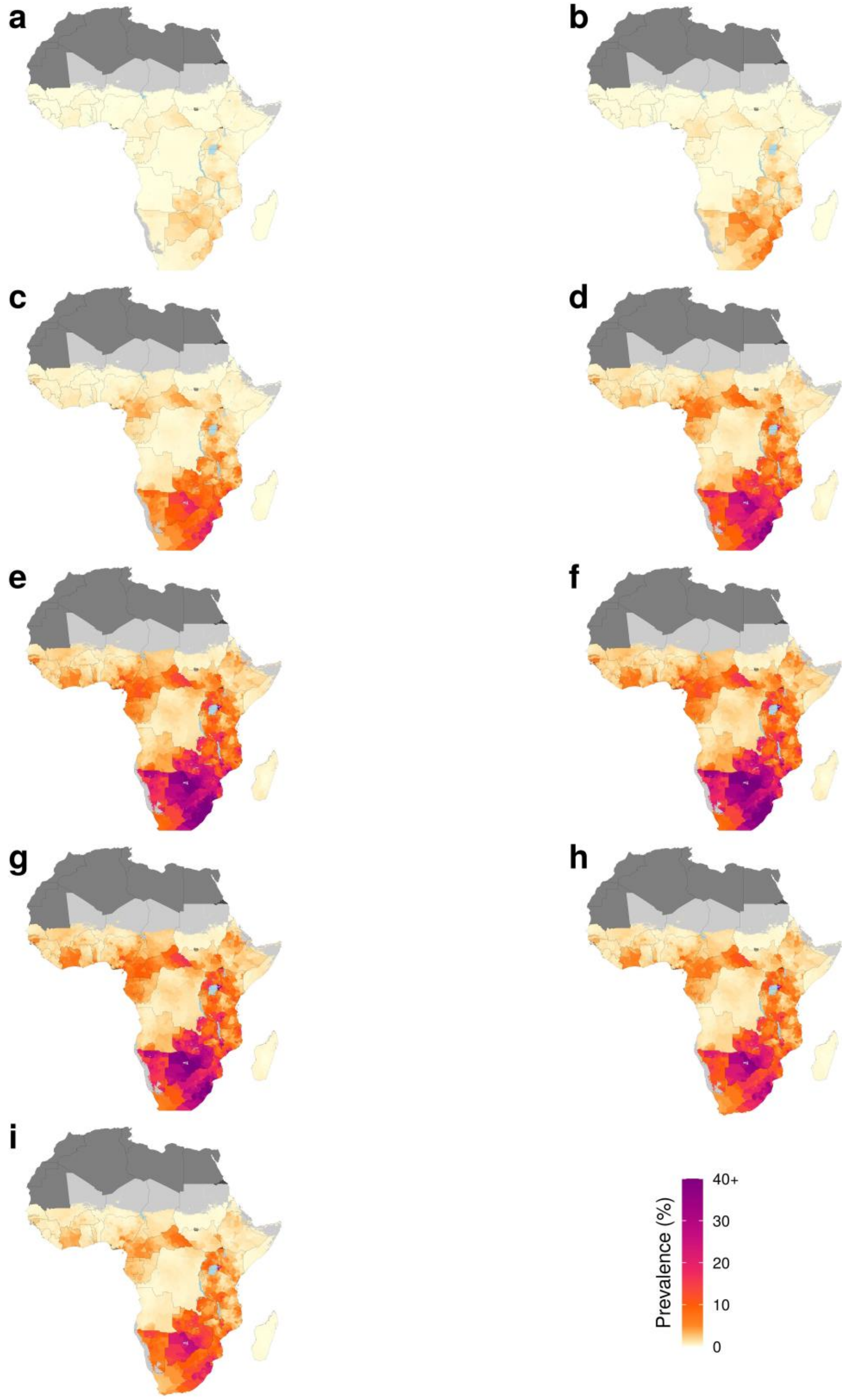

Figure S23: Age-specific HIV prevalence in males, 2010. Male HIV prevalence estimates in the year 2010, at the second administrative level, for (a) ages 15–19 years; (b) ages 20-24 years; (c) ages 25-29 years; (d) ages 30-34 years; (e) ages 35-39 years; (f) ages 40-44 years; (g) ages 45-49 years; (h) ages 50-54 years; and (i) ages 55-59 years. Maps reflect national boundaries, land cover, lakes, and population; areas with fewer than ten people per 1 × 1 km, and classified as barren or sparsely vegetated, are colored light grey. Countries colored in dark grey were not included in the analysis.

244 # Figure S24: Age-specific HIV prevalence in females, 2010

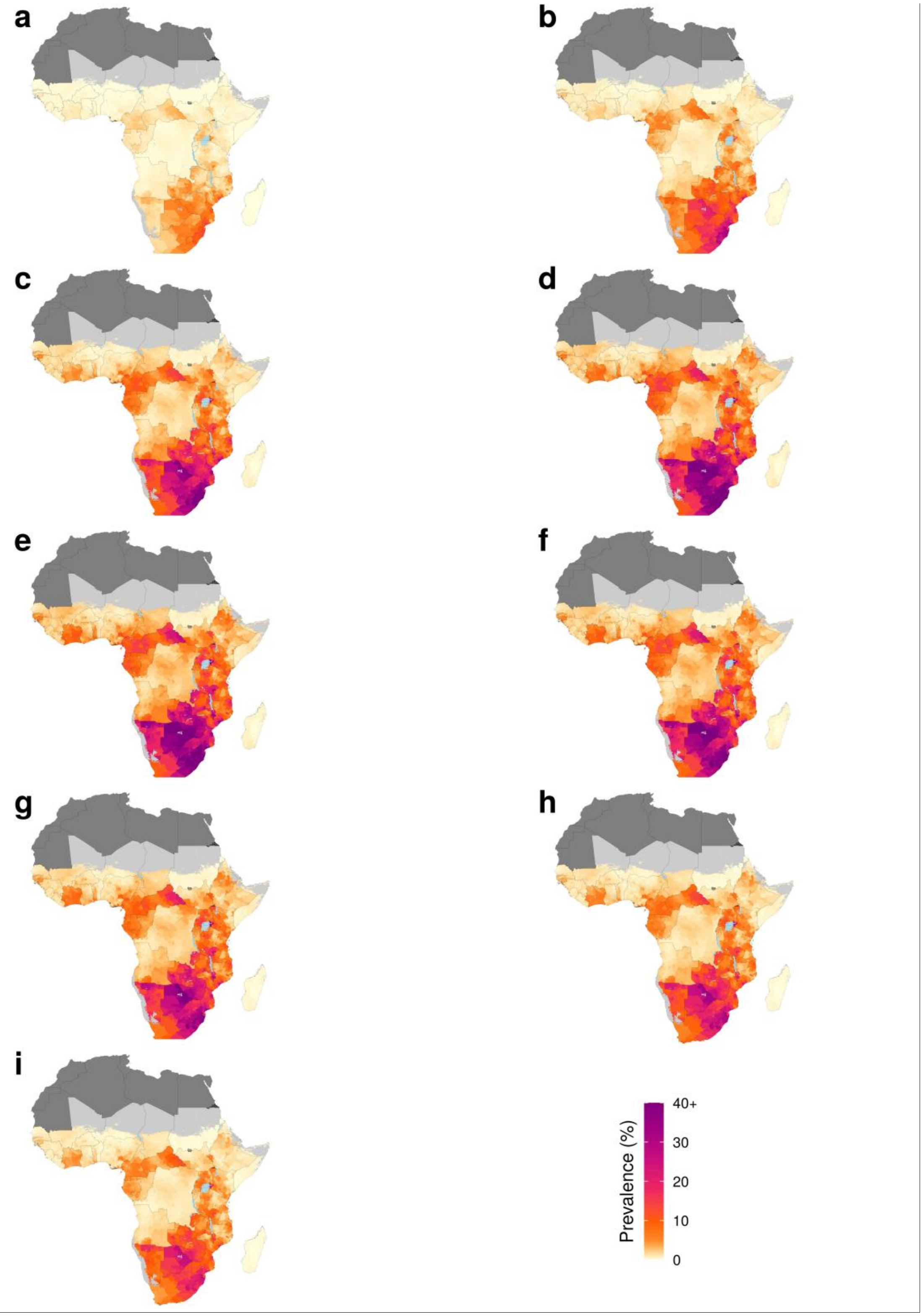

Figure S24: Age-specific HIV prevalence in females, 2010. Female HIV prevalence estimates in the year 2010, at the second administrative level, for (a) ages 15–19 years; (b) ages 20-24 years; (c) ages 25-29 years; (d) ages 30-34 years; (e) ages 35-39 years; (f) ages 40-44 years; (g) ages 45-49 years; (h) ages 50-54 years; and (i) ages 55-59 years. Maps reflect national boundaries, land cover, lakes, and population; areas with fewer than ten people per 1 × 1 km, and classified as barren or sparsely vegetated, are colored light grey. Countries colored in dark grey were not included in the analysis.

Figure S25: Age-specific HIV prevalence in males, 2018

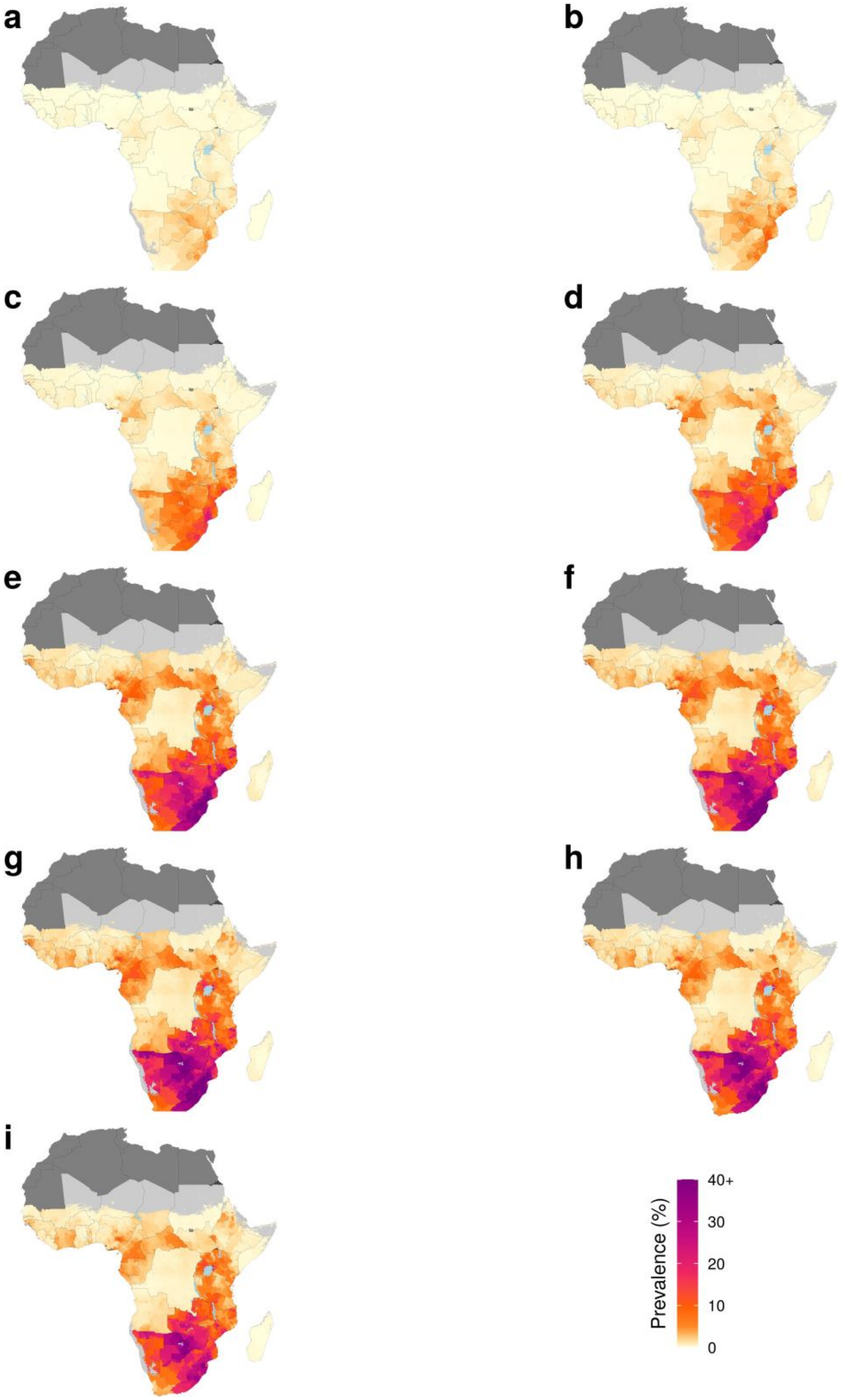

Figure S25: Age-specific HIV prevalence in males, 2018. Male HIV prevalence estimates in the year 2018, at the second administrative level, for (a) ages 15–19 years; (b) ages 20-24 years; (c) ages 25-29 years; (d) ages 30-34 years; (e) ages 35-39 years; (f) ages 40-44 years; (g) ages 45-49 years; (h) ages 50-54 years; and (i) ages 55-59 years. Maps reflect national boundaries, land cover, lakes, and population; areas with fewer than ten people per 1 × 1 km, and classified as barren or sparsely vegetated, are colored light grey. Countries colored in dark grey were not included in the analysis.

Figure S26: Age-specific HIV prevalence in females, 2018

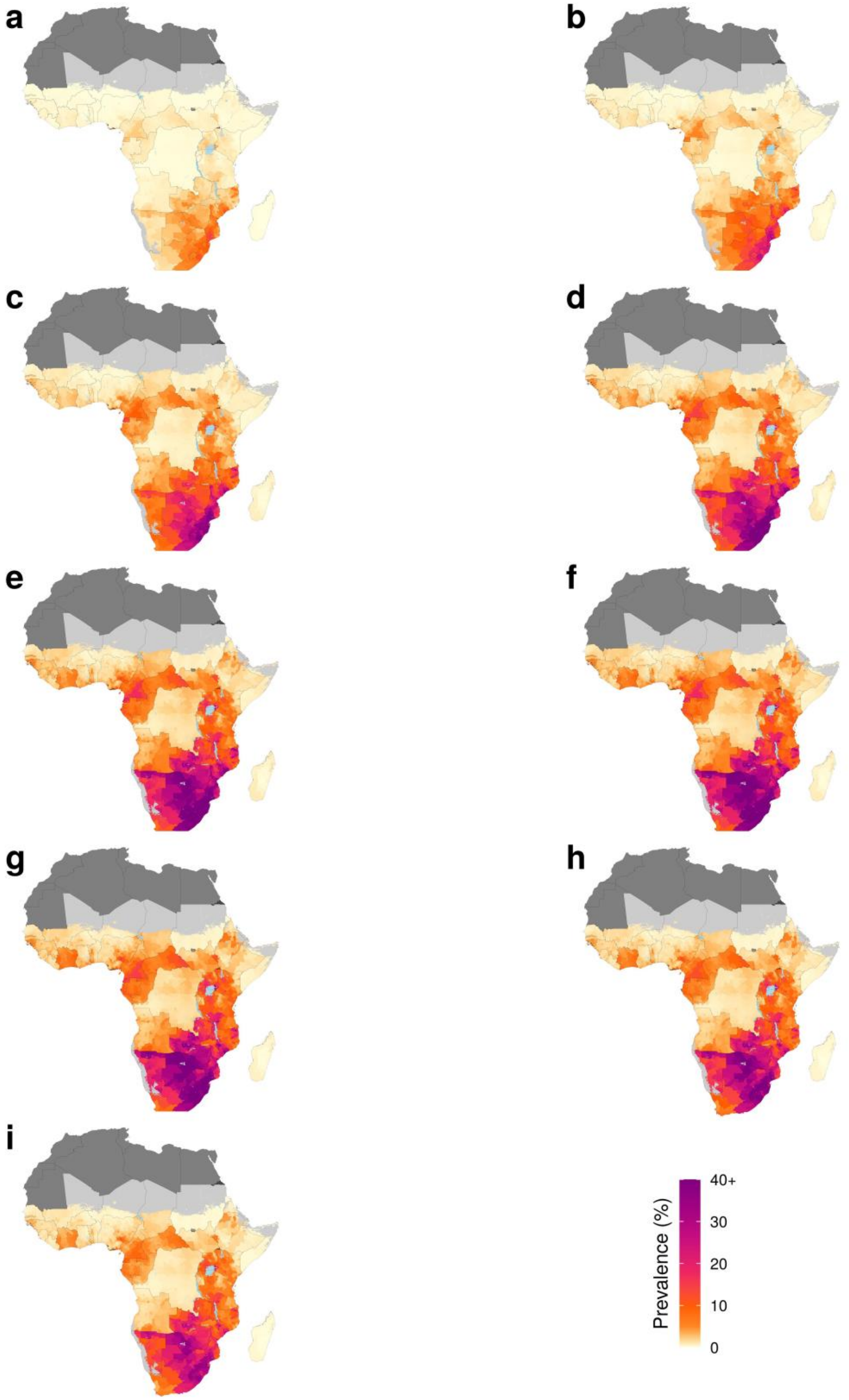

Figure S26: Age-specific HIV prevalence in females, 2018. Female HIV prevalence estimates in the year 2018, at the second administrative level, for (a) ages 15–19 years; (b) ages 20-24 years; (c) ages 25-29 years; (d) ages 30-34 years; (e) ages 35-39 years; (f) ages 40-44 years; (g) ages 45-49 years; (h) ages 50-54 years; and (i) ages 55-59 years. Maps reflect national boundaries, land cover, lakes, and population; areas with fewer than ten people per 1 × 1 km, and classified as barren or sparsely vegetated, are colored light grey. Countries colored in dark grey were not included in the analysis.

Figure S27: Age-specific uncertainty interval range estimates in males, 2000

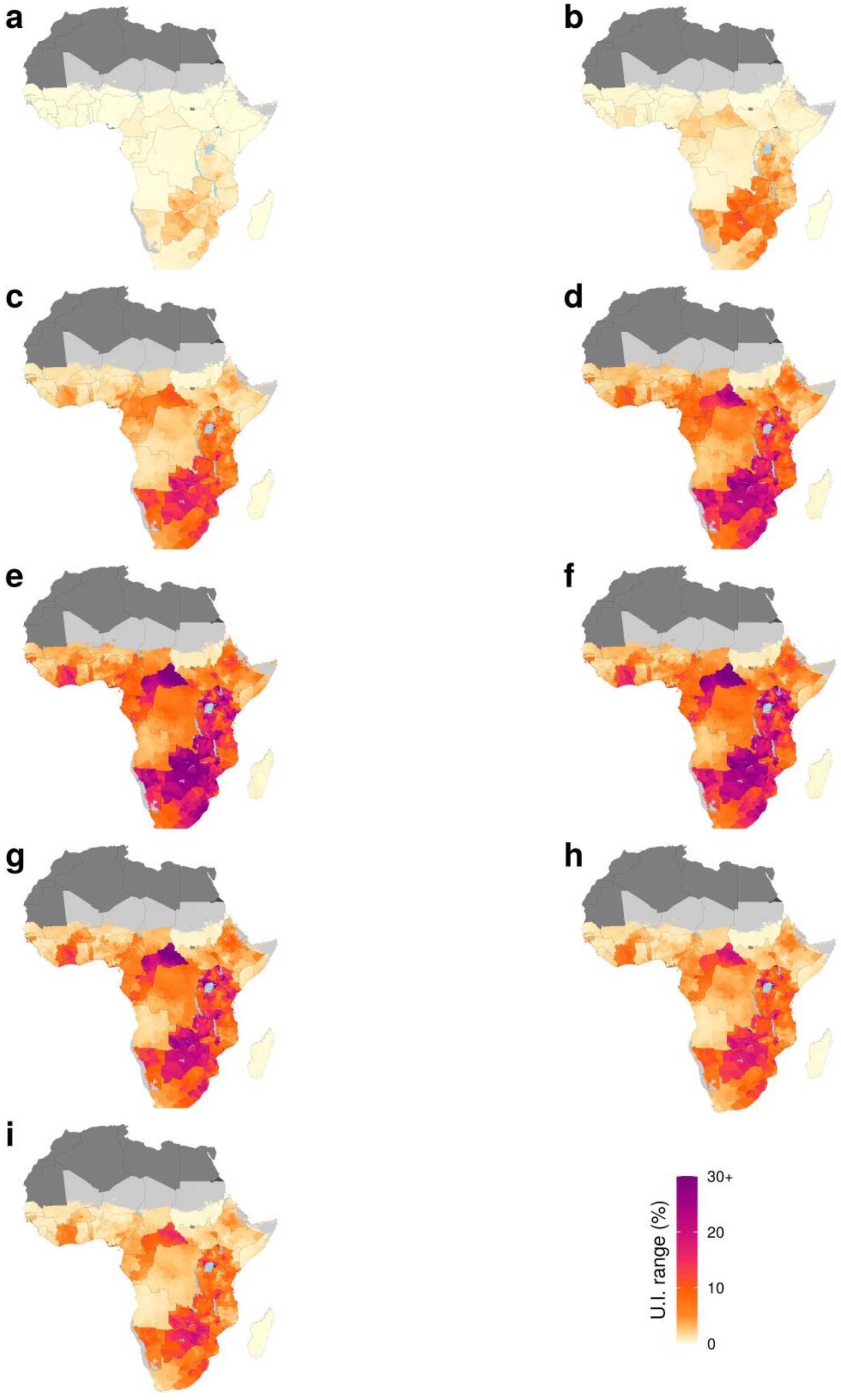

Figure S27: Age-specific uncertainty interval range estimates in males, 2000. Uncertainty interval range for male HIV prevalence estimates in the year 2000, at the second administrative level, for **(a)** ages 15–19 years; (b) ages 20-24 years; **(c) ages 25-29 years; (d) ages 30-34 years; (e) ages 35-39 years; (f) ages 40-44 years; (g) ages 45-49 years; (h) ages 50-54 years; and (i) ages 55-59 years.** Maps reflect national boundaries, land cover, lakes, and population; areas with fewer than ten people per 1 × 1 km, and classified as barren or sparsely vegetated, are colored light grey. Countries colored in dark grey were not included in the analysis.

281 Figure S28: Age-specific uncertainty interval range estimates in females,
282 2000

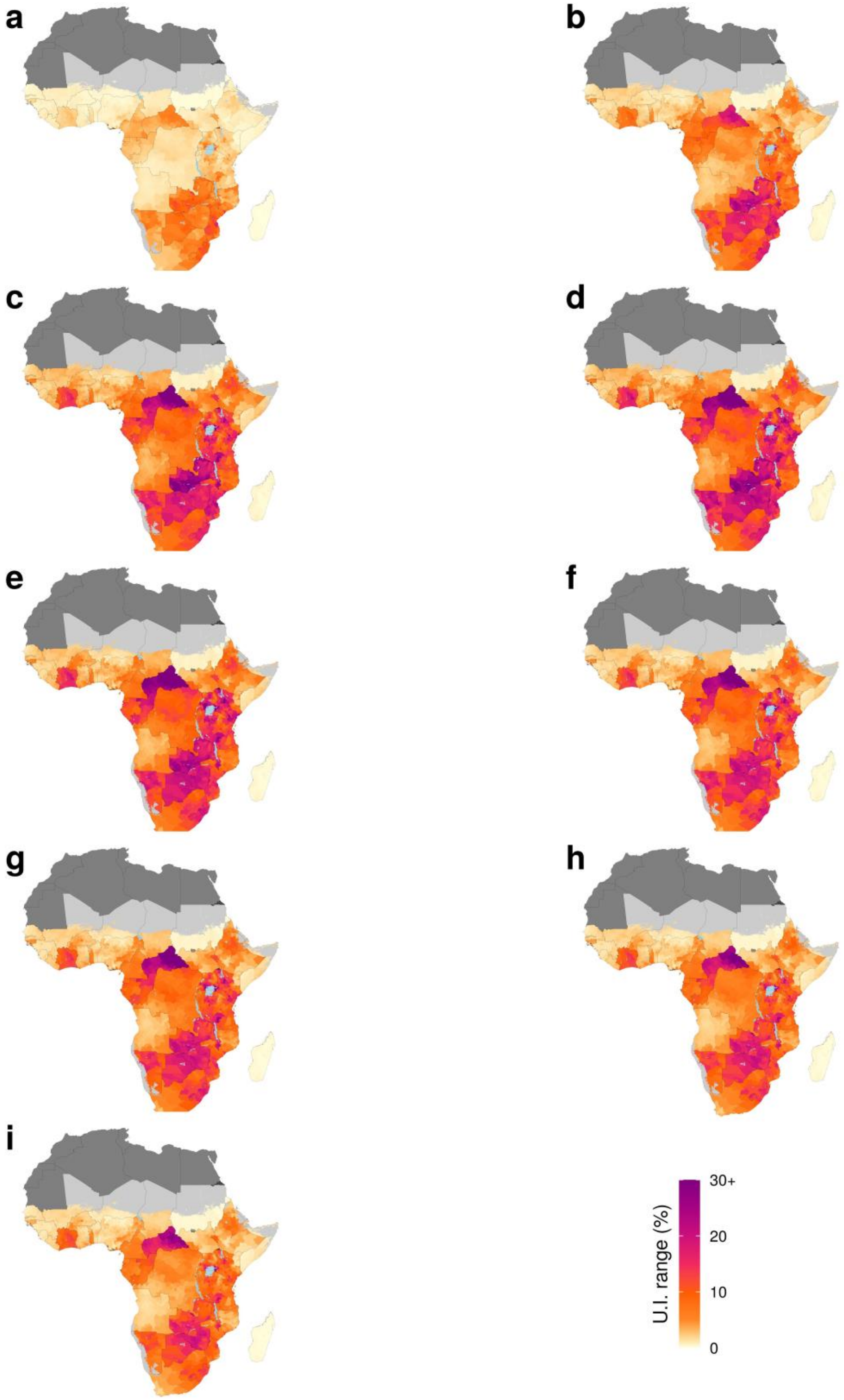

283

Figure S28: Age-specific uncertainty interval range estimates in females, 2000. Uncertainty interval range for female HIV prevalence estimates in the year 2000, at the second administrative level, for (a) ages 15–19 years; (b) ages 20-24 years; (c) ages 25-29 years; (d) ages 30-34 years; (e) ages 35-39 years; (f) ages 40-44 years; (g) ages 45-49 years; (h) ages 50-54 years; and (i) ages 55-59 years. Maps reflect national boundaries, land cover, lakes, and population; areas with fewer than ten people per 1 × 1 km, and classified as barren or sparsely vegetated, are colored light grey. Countries colored in dark grey were not included in the analysis.

Figure S29: Age-specific uncertainty interval range estimates in males, 2005

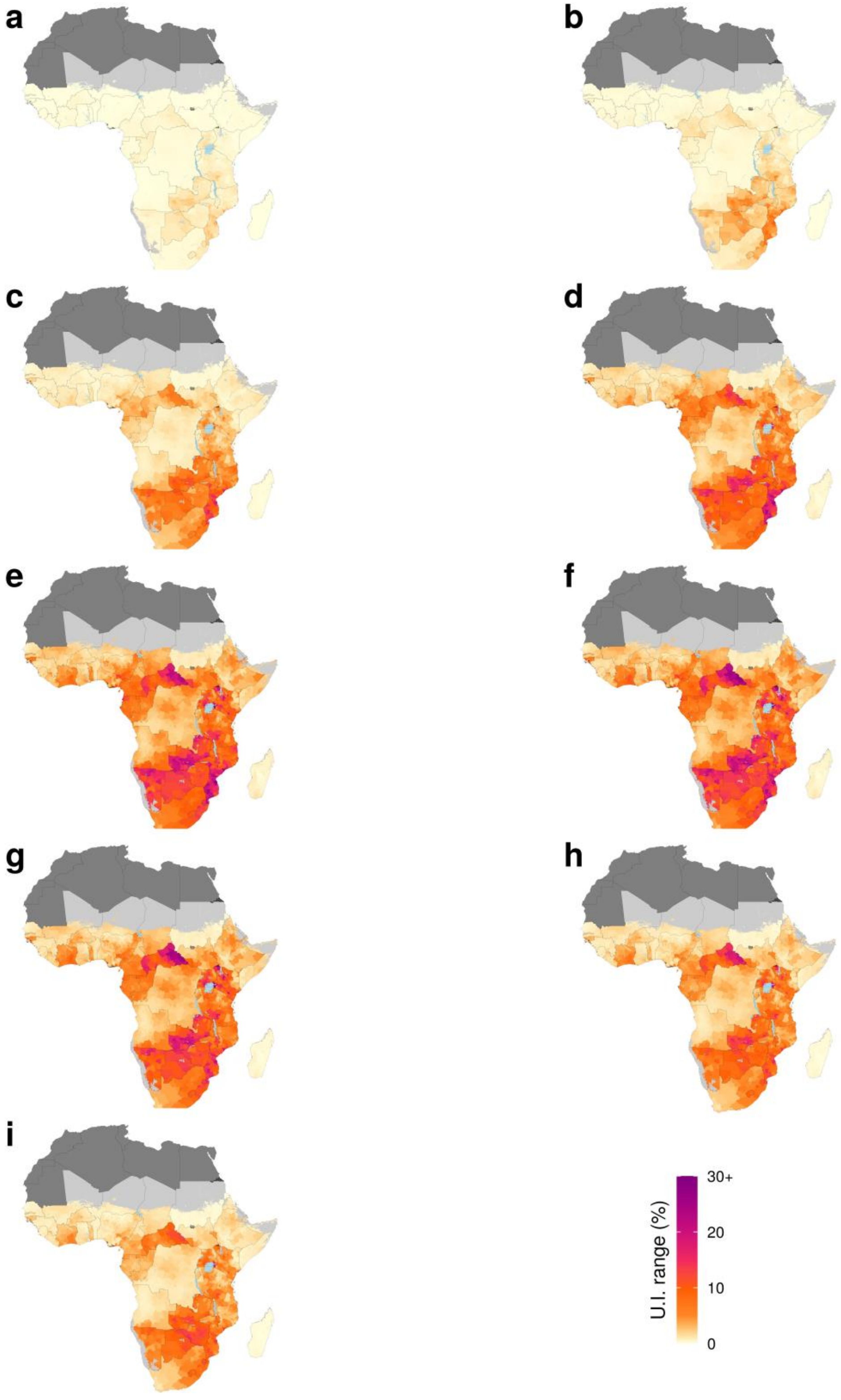

Figure S29: Age-specific uncertainty interval range estimates in males, 2005. Uncertainty interval range for male HIV prevalence estimates in the year 2005, at the second administrative level, for (a) ages 15–19 years; (b) ages 20-24 years; (c) ages 25-29 years; (d) ages 30-34 years; (e) ages 35-39 years; (f) ages 40-44 years; (g) ages 45-49 years; (h) ages 50-54 years; and (i) ages 55-59 years. Maps reflect national boundaries, land cover, lakes, and population; areas with fewer than ten people per 1 × 1 km, and classified as barren or sparsely vegetated, are colored light grey. Countries colored in dark grey were not included in the analysis.

Figure S30: Age-specific uncertainty interval range estimates in females, 2005

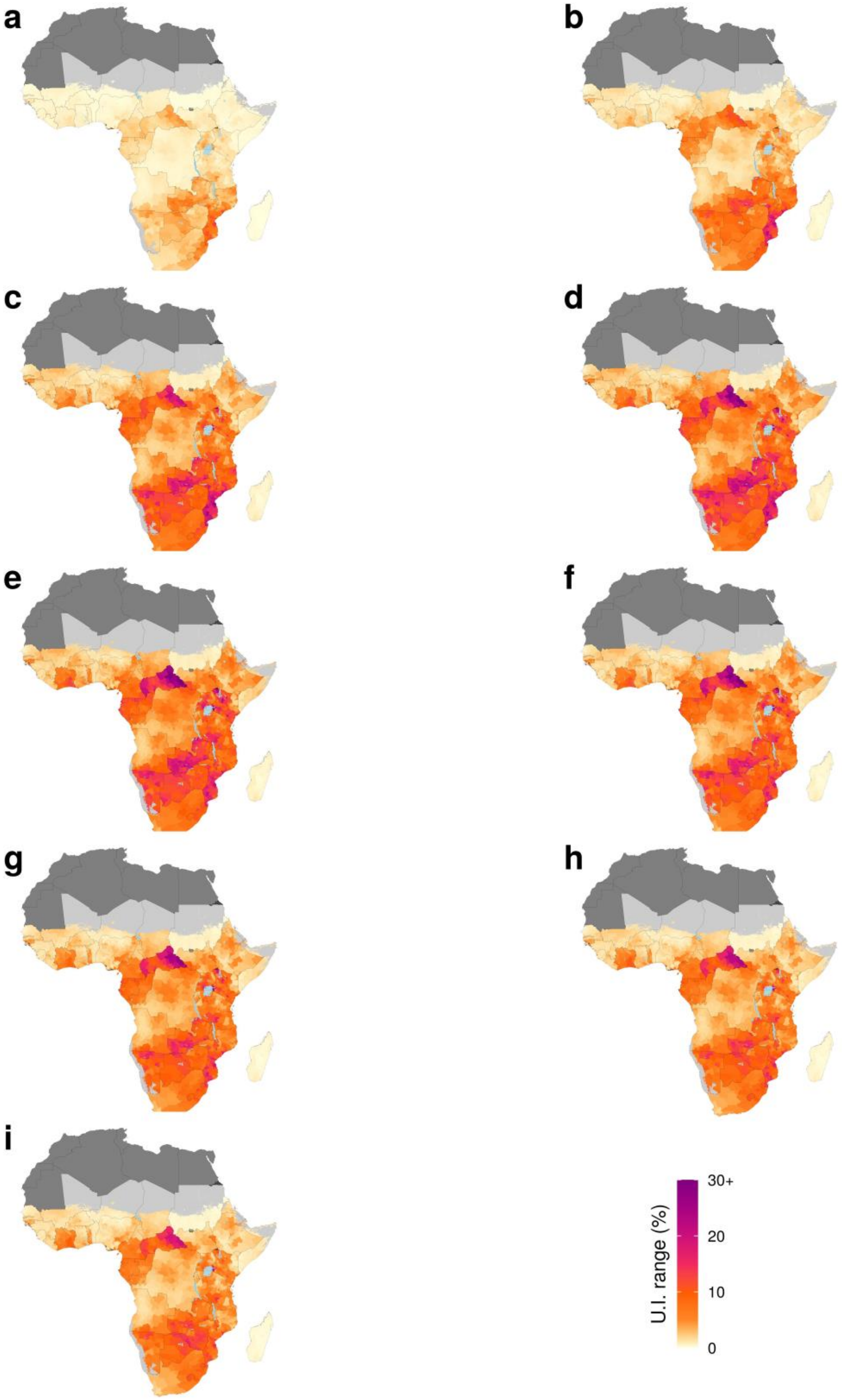

Figure S30: Age-specific uncertainty interval range estimates in females, 2005. Uncertainty interval range for female HIV prevalence estimates in the year 2005, at the second administrative level, for (a) ages 15–19 years; (b) ages 20-24 years; (c) ages 25-29 years; (d) ages 30-34 years; (e) ages 35-39 years; (f) ages 40-44 years; (g) ages 45-49 years; (h) ages 50-54 years; and (i) ages 55-59 years. Maps reflect national boundaries, land cover, lakes, and population; areas with fewer than ten people per 1 × 1 km, and classified as barren or sparsely vegetated, are colored light grey. Countries colored in dark grey were not included in the analysis.

Figure S31: Age-specific uncertainty interval range estimates in males, 2010

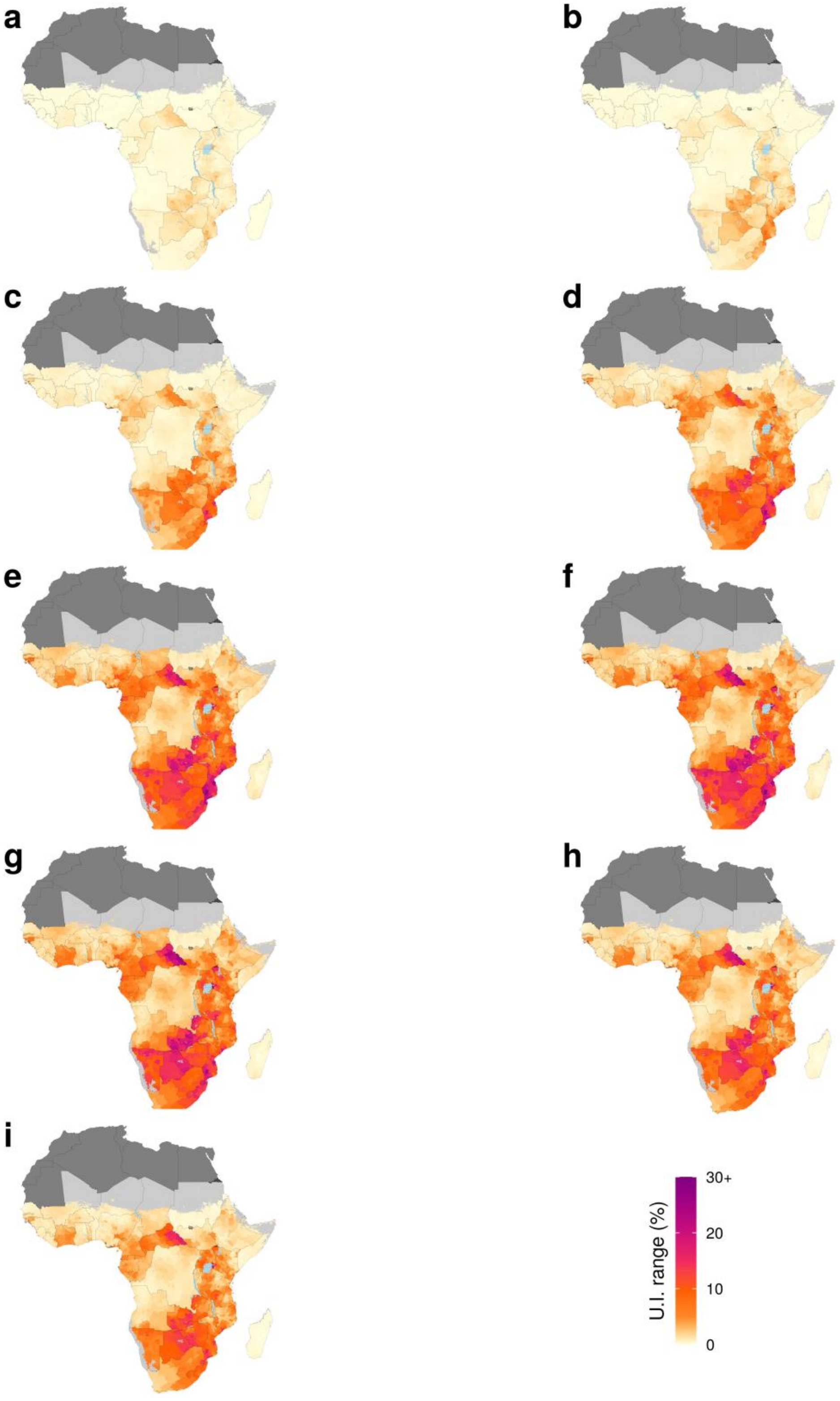

Figure S31: Age-specific uncertainty interval range estimates in males, 2010. Uncertainty interval range for male HIV prevalence estimates in the year 2010, at the second administrative level, for (a) ages 15–19 years; (b) ages 20-24 years; (c) ages 25-29 years; (d) ages 30-34 years; (e) ages 35-39 years; (f) ages 40-44 years; (g) ages 45-49 years; (h) ages 50-54 years; and (i) ages 55-59 years. Maps reflect national boundaries, land cover, lakes, and population; areas with fewer than ten people per 1 × 1 km, and classified as barren or sparsely vegetated, are colored light grey. Countries colored in dark grey were not included in the analysis.

Figure S32: Age-specific uncertainty interval range estimates in females, 2010

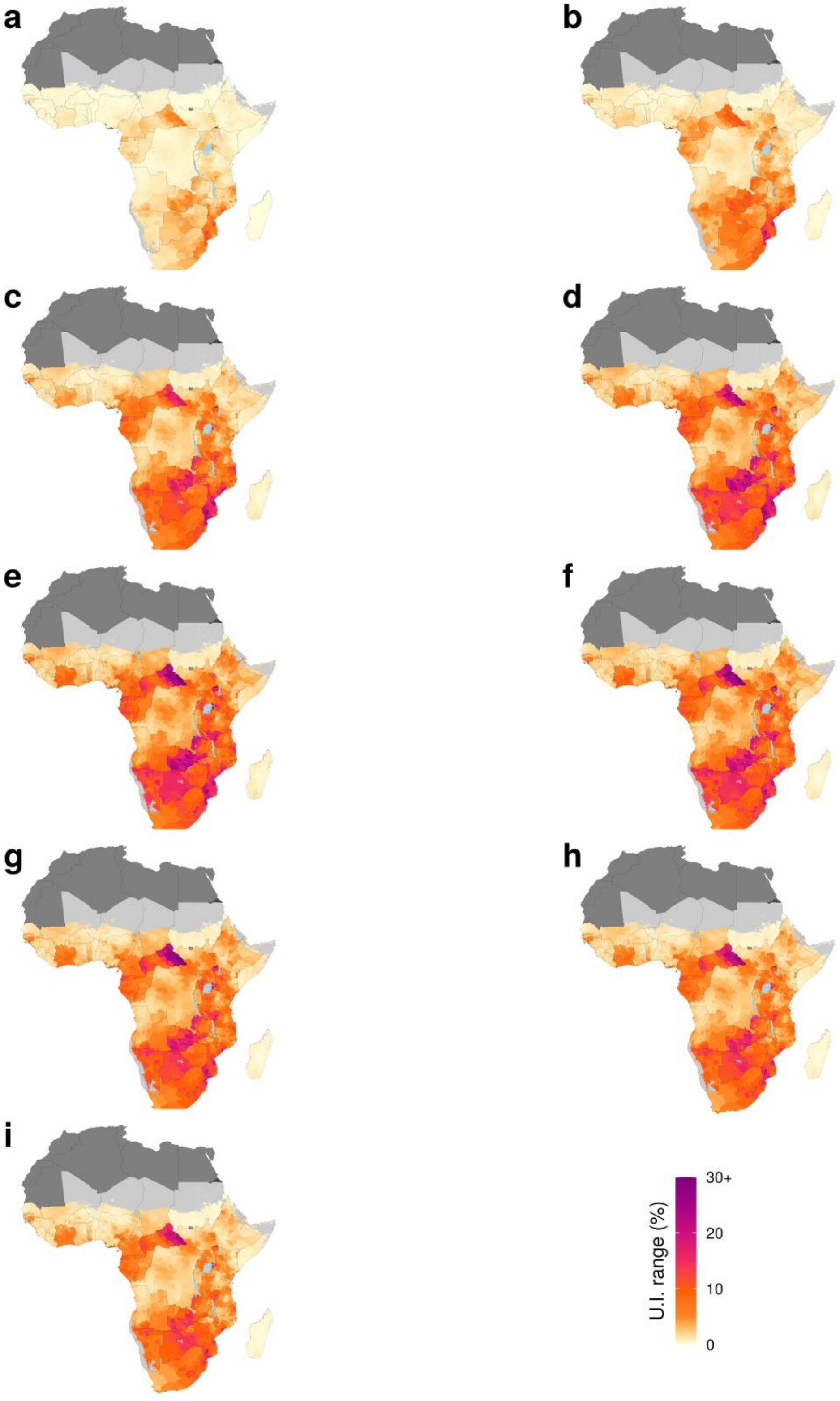

Figure S32: Age-specific uncertainty interval range estimates in females, 2010. Uncertainty interval range for female HIV prevalence estimates in the year 2010, at the second administrative level, for **(a)** ages 15–19 years; (b) ages 20-24 years; **(c) ages 25-29 years; (d) ages 30-34 years; (e) ages 35-39 years; (f) ages 40-44 years; (g) ages 45-49 years; (h) ages 50-54 years; and (i) ages 55-59 years.** Maps reflect national boundaries, land cover, lakes, and population; areas with fewer than ten people per 1 × 1 km, and classified as barren or sparsely vegetated, are colored light grey. Countries colored in dark grey were not included in the analysis.

Figure S33: Age-specific uncertainty interval range estimates in males, 2018

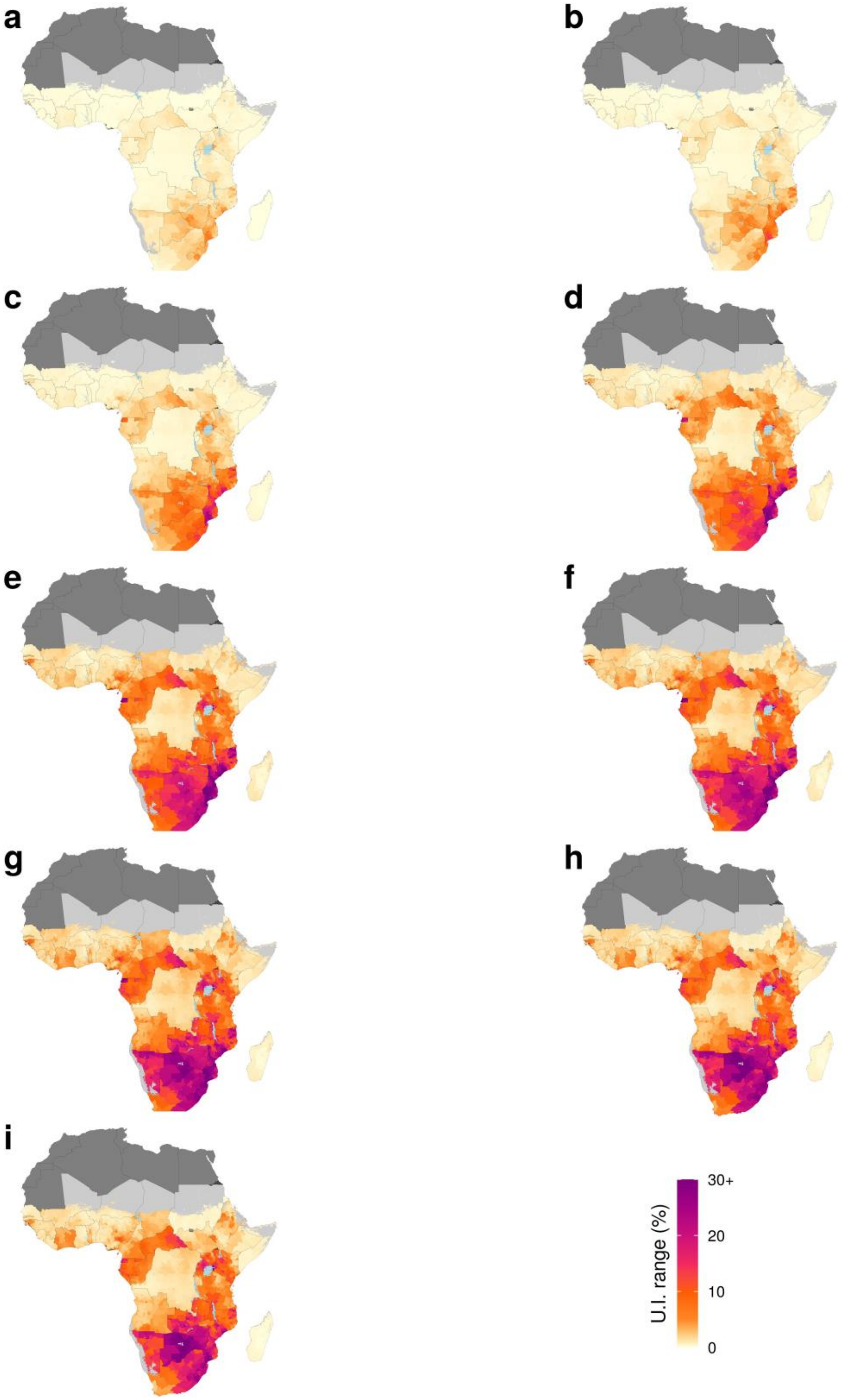

Figure S33: Age-specific uncertainty interval range estimates in males, 2018. Uncertainty interval range for male HIV prevalence estimates in the year 2018, at the second administrative level, for (a) ages 15–19 years; (b) ages 20-24 years; (c) ages 25-29 years; (d) ages 30-34 years; (e) ages 35-39 years; (f) ages 40-44 years; (g) ages 45-49 years; (h) ages 50-54 years; and (i) ages 55-59 years. Maps reflect national boundaries, land cover, lakes, and population; areas with fewer than ten people per 1 × 1 km, and classified as barren or sparsely vegetated, are colored light grey. Countries colored in dark grey were not included in the analysis.

Figure S34: Age-specific uncertainty interval range estimates in females, 2018

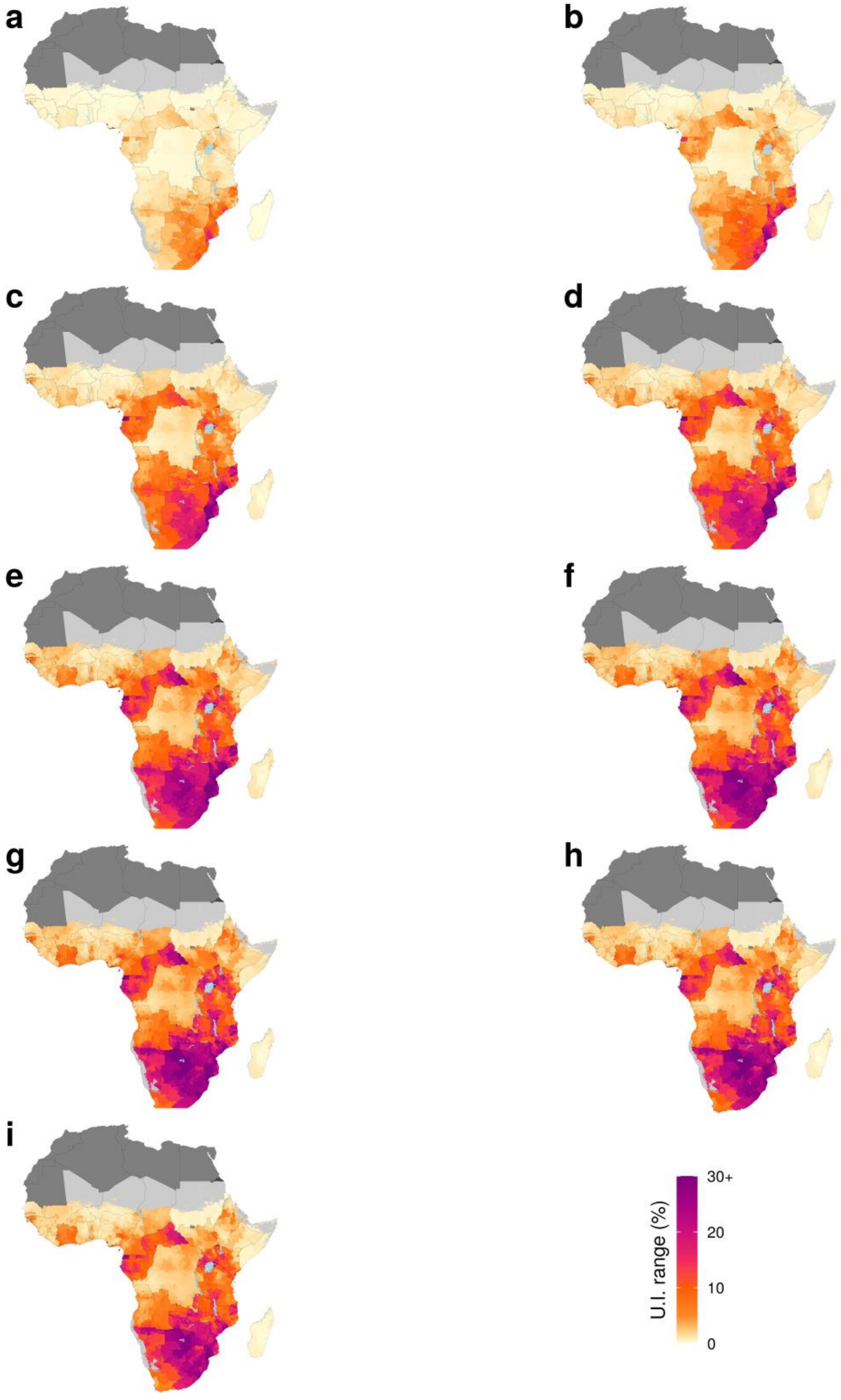

Figure S34: Age-specific uncertainty interval range estimates in females, 2018. Uncertainty interval range for female HIV prevalence estimates in the year 2010, at the second administrative level, for (a) ages 15–19 years; (b) ages 20-24 years; (c) ages 25-29 years; (d) ages 30-34 years; (e) ages 35-39 years; (f) ages 40-44 years; (g) ages 45-49 years; (h) ages 50-54 years; and (i) ages 55-59 years. Maps reflect national boundaries, land cover, lakes, and population; areas with fewer than ten people per 1 × 1 km, and classified as barren or sparsely vegetated, are colored light grey. Countries colored in dark grey were not included in the analysis.

Figure S35: Change in HIV prevalence in males, 2000–2005

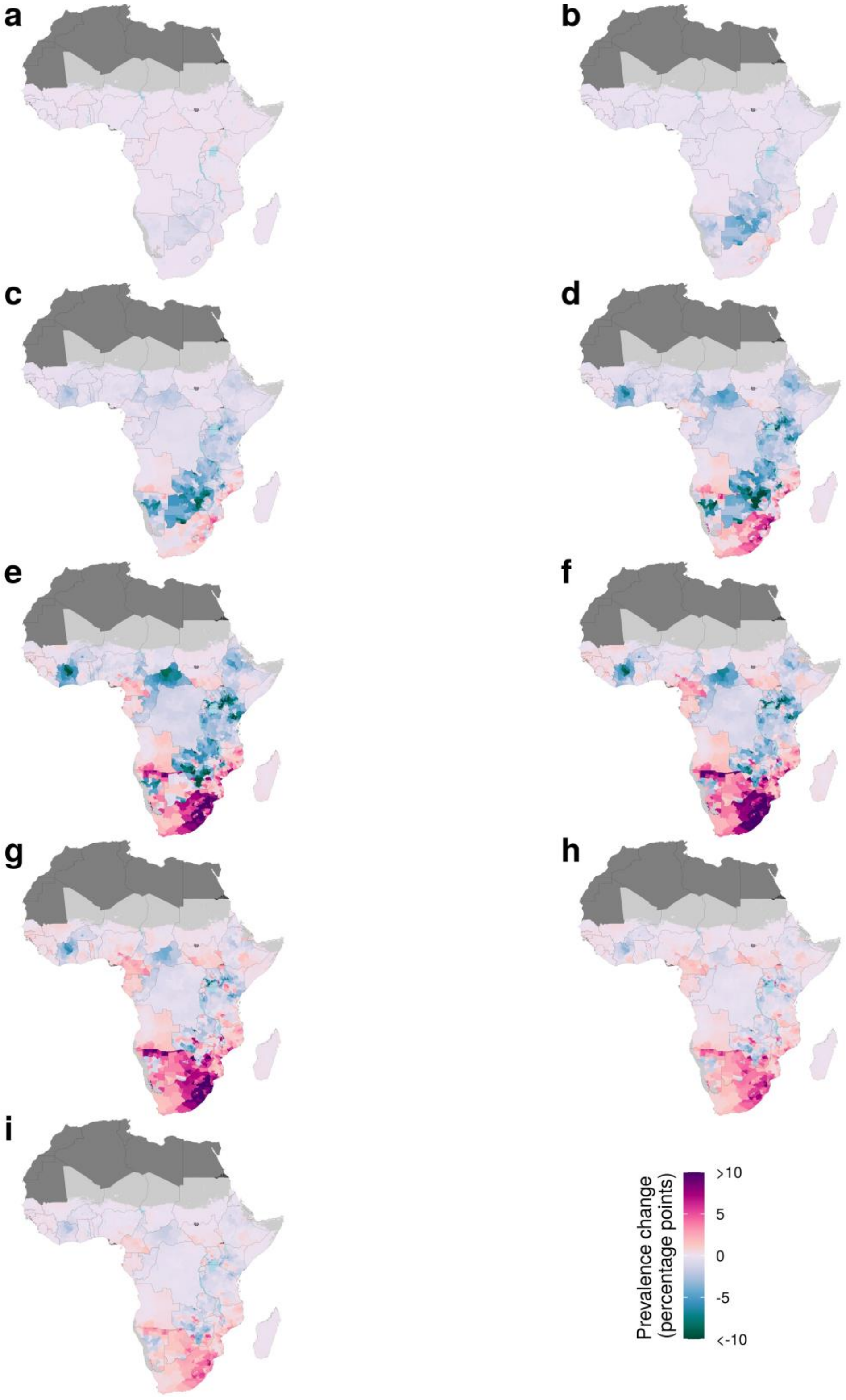

Figure S35: Change in HIV prevalence in males, 2000-2005. Absolute change in male HIV prevalence at the second administrative level for (a) ages 15–19 years; (b) ages 20-24 years; (c) ages 25-29 years; (d) ages 30-34 years; (e) ages 35-39 years; (f) ages 40-44 years; (g) ages 45-49 years; (h) ages 50-54 years; and (i) ages 55-59 years. Maps reflect national boundaries, land cover, lakes, and population; areas with fewer than ten people per 1 × 1 km, and classified as barren or sparsely vegetated, are colored light grey. Countries colored in dark grey were not included in the analysis.

Figure S36: Change in HIV prevalence in females, 2000–2005

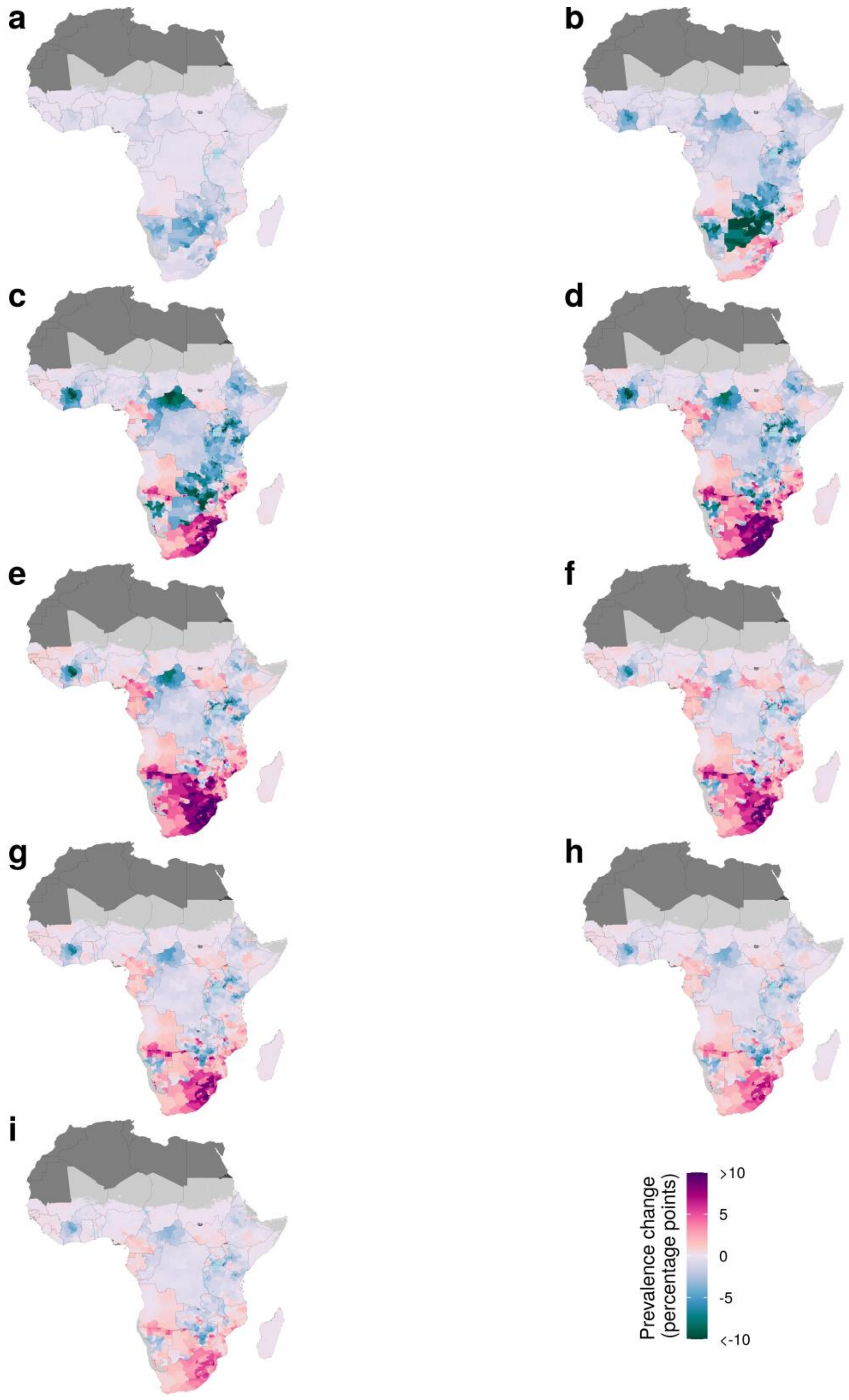

Figure S36: Change in HIV prevalence in females, 2000-2005. Absolute change in female HIV prevalence at the second administrative level for (a) ages 15–19 years; (b) ages 20-24 years; (c) ages 25-29 years; (d) ages 30-34 years; (e) ages 35-39 years; (f) ages 40-44 years; (g) ages 45-49 years; (h) ages 50-54 years; and (i) ages 55-59 years. Maps reflect national boundaries, land cover, lakes, and population; areas with fewer than ten people per 1 × 1 km, and classified as barren or sparsely vegetated, are colored light grey. Countries colored in dark grey were not included in the analysis.

367 # Figure S37: Change in HIV prevalence in males, 2005–2010

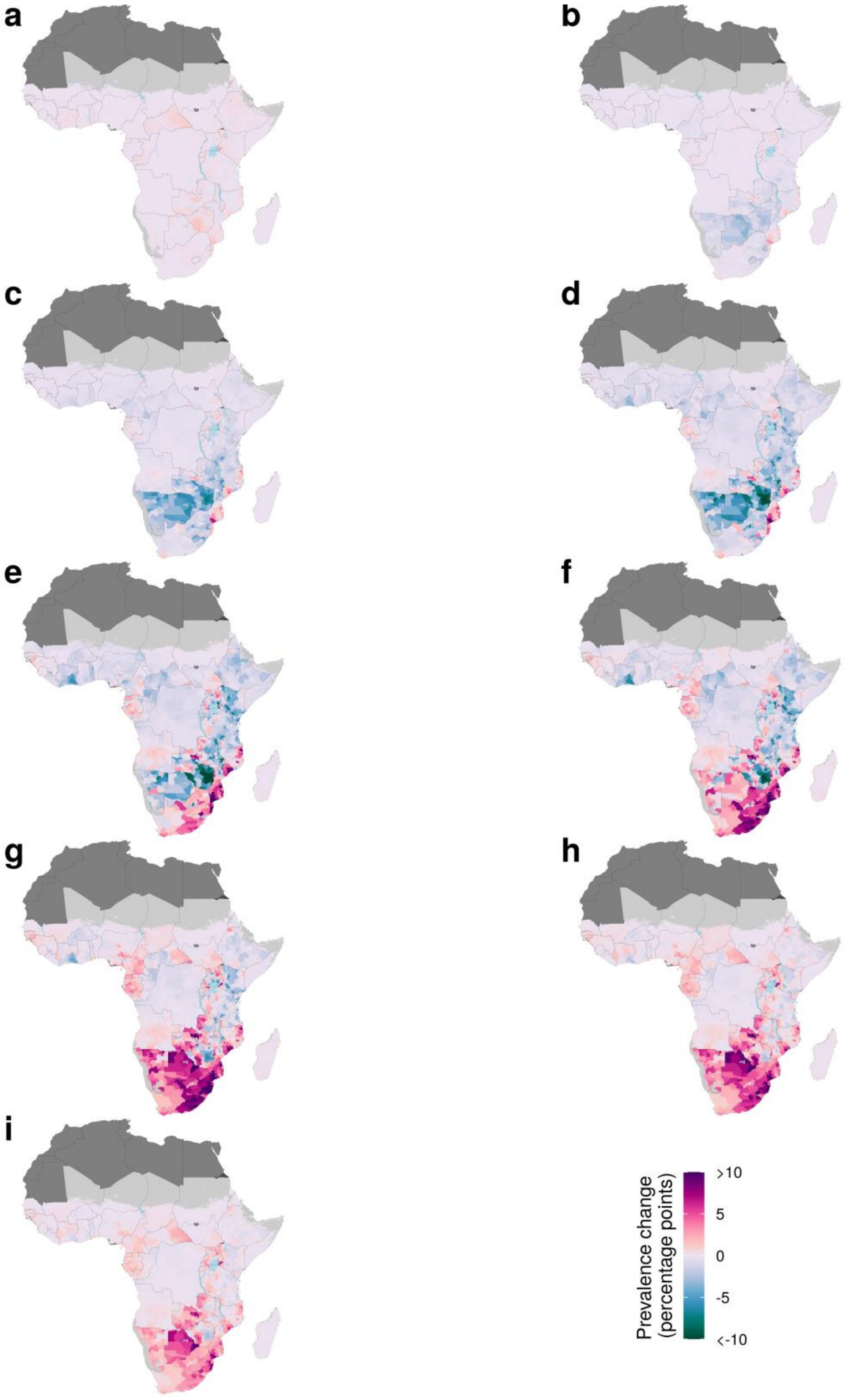

368

Figure S37: Change in HIV prevalence in males, 2005-2010. Absolute change in male HIV prevalence at the second administrative level for (a) ages 15–19 years; (b) ages 20-24 years; (c) ages 25-29 years; (d) ages 30-34 years; (e) ages 35-39 years; (f) ages 40-44 years; (g) ages 45-49 years; (h) ages 50-54 years; and (i) ages 55-59 years. Maps reflect national boundaries, land cover, lakes, and population; areas with fewer than ten people per 1 × 1 km, and classified as barren or sparsely vegetated, are colored light grey. Countries colored in dark grey were not included in the analysis.

Figure S38: Change in HIV prevalence in females, 2005–2010

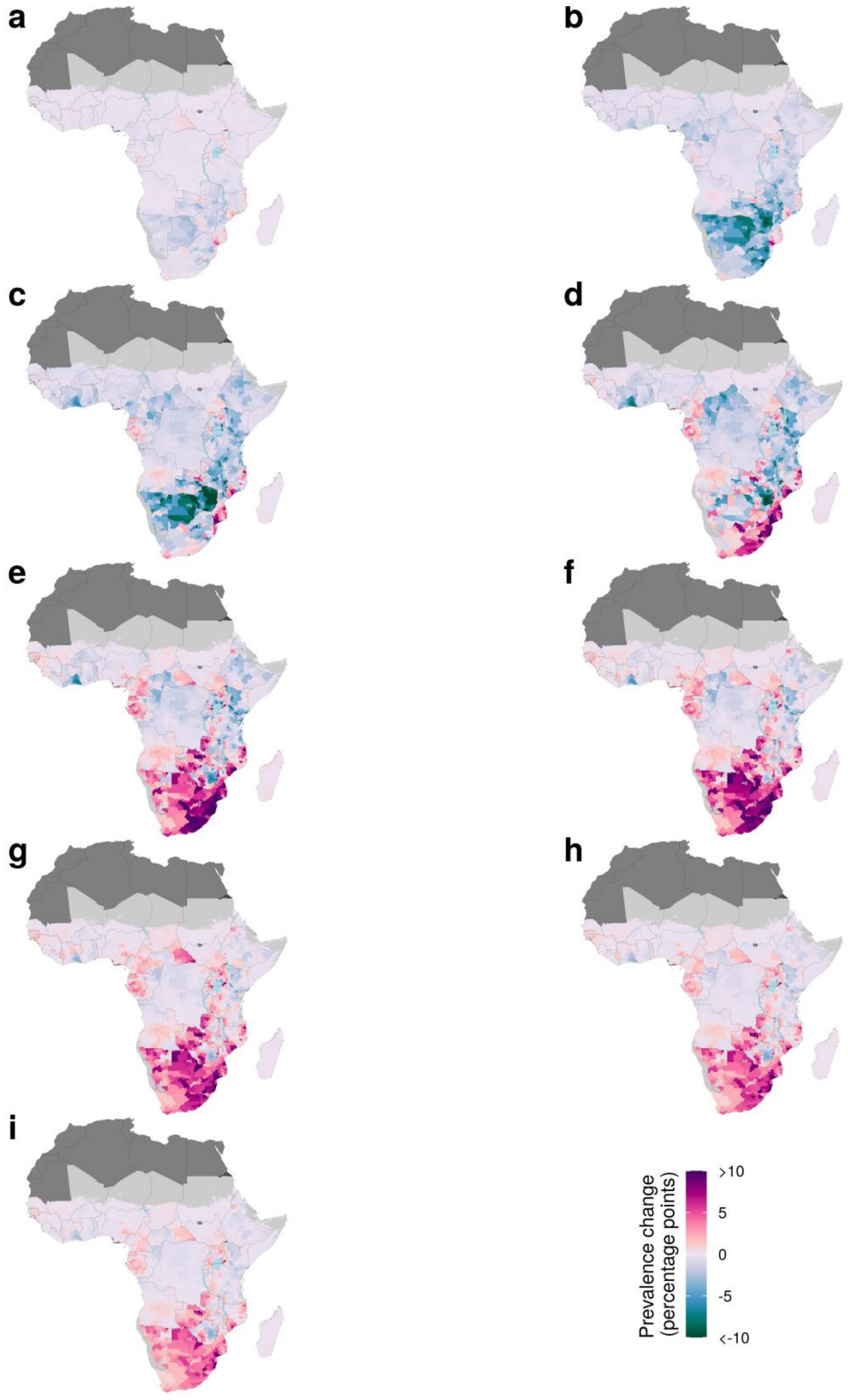

Figure S38: Change in HIV prevalence in females, 2005-2010. Absolute change in female HIV prevalence at the second administrative level for (a) ages 15–19 years; (b) ages 20-24 years; (c) ages 25-29 years; (d) ages 30-34 years; (e) ages 35-39 years; (f) ages 40-44 years; (g) ages 45-49 years; (h) ages 50-54 years; and (i) ages 55-59 years. Maps reflect national boundaries, land cover, lakes, and population; areas with fewer than ten people per 1 × 1 km, and classified as barren or sparsely vegetated, are colored light grey. Countries colored in dark grey were not included in the analysis.

383 Figure S39: Change in HIV prevalence in males, 2010–2018

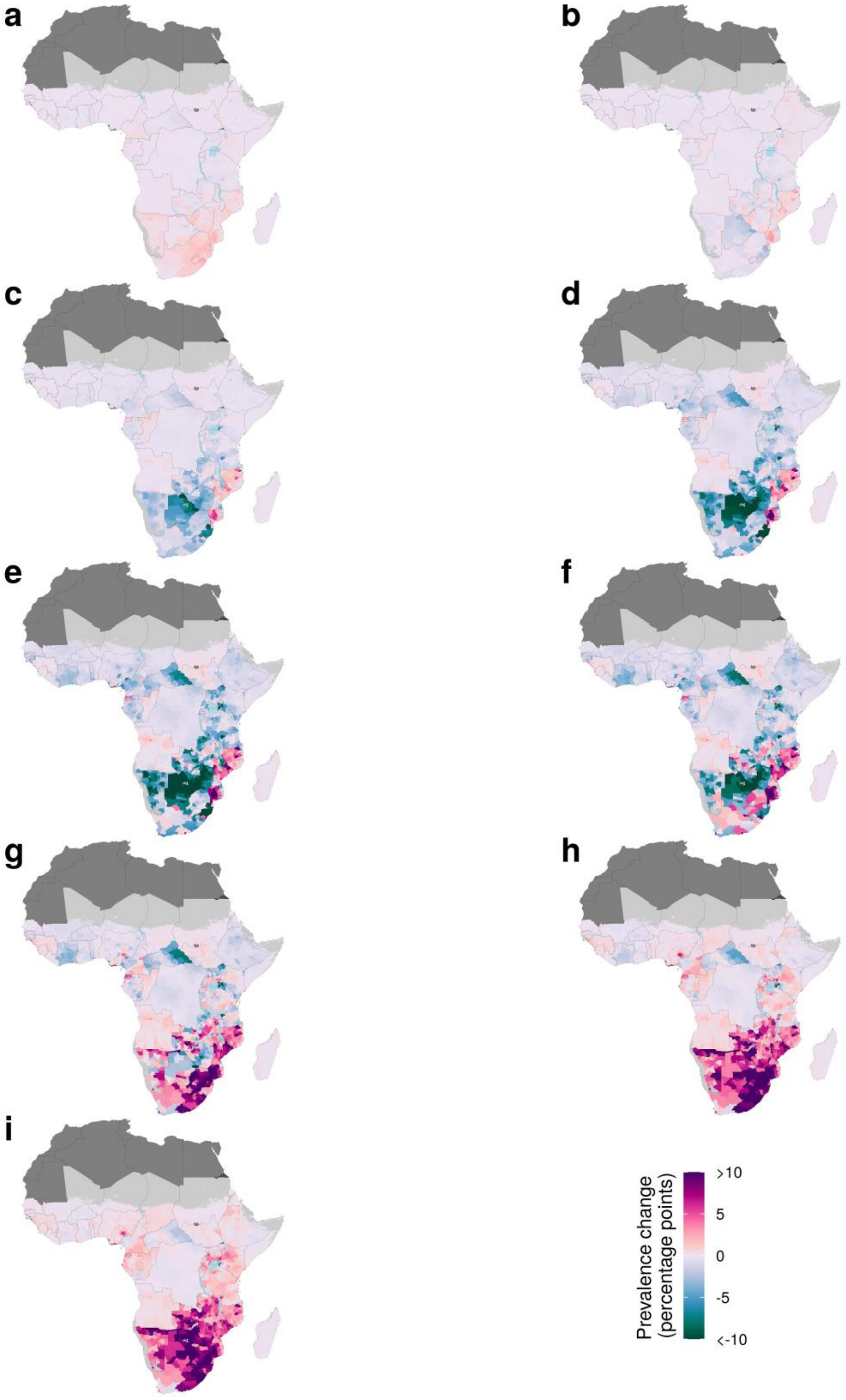

384

Figure S39: Change in HIV prevalence in males, 2010-2018. Absolute change in male HIV prevalence at the second administrative level for (a) ages 15–19 years; (b) ages 20-24 years; (c) ages 25-29 years; (d) ages 30-34 years; (e) ages 35-39 years; (f) ages 40-44 years; (g) ages 45-49 years; (h) ages 50-54 years; and (i) ages 55-59 years. Maps reflect national boundaries, land cover, lakes, and population; areas with fewer than ten people per 1 × 1 km, and classified as barren or sparsely vegetated, are colored light grey. Countries colored in dark grey were not included in the analysis.

Figure S40: Change in HIV prevalence in females, 2010–2018

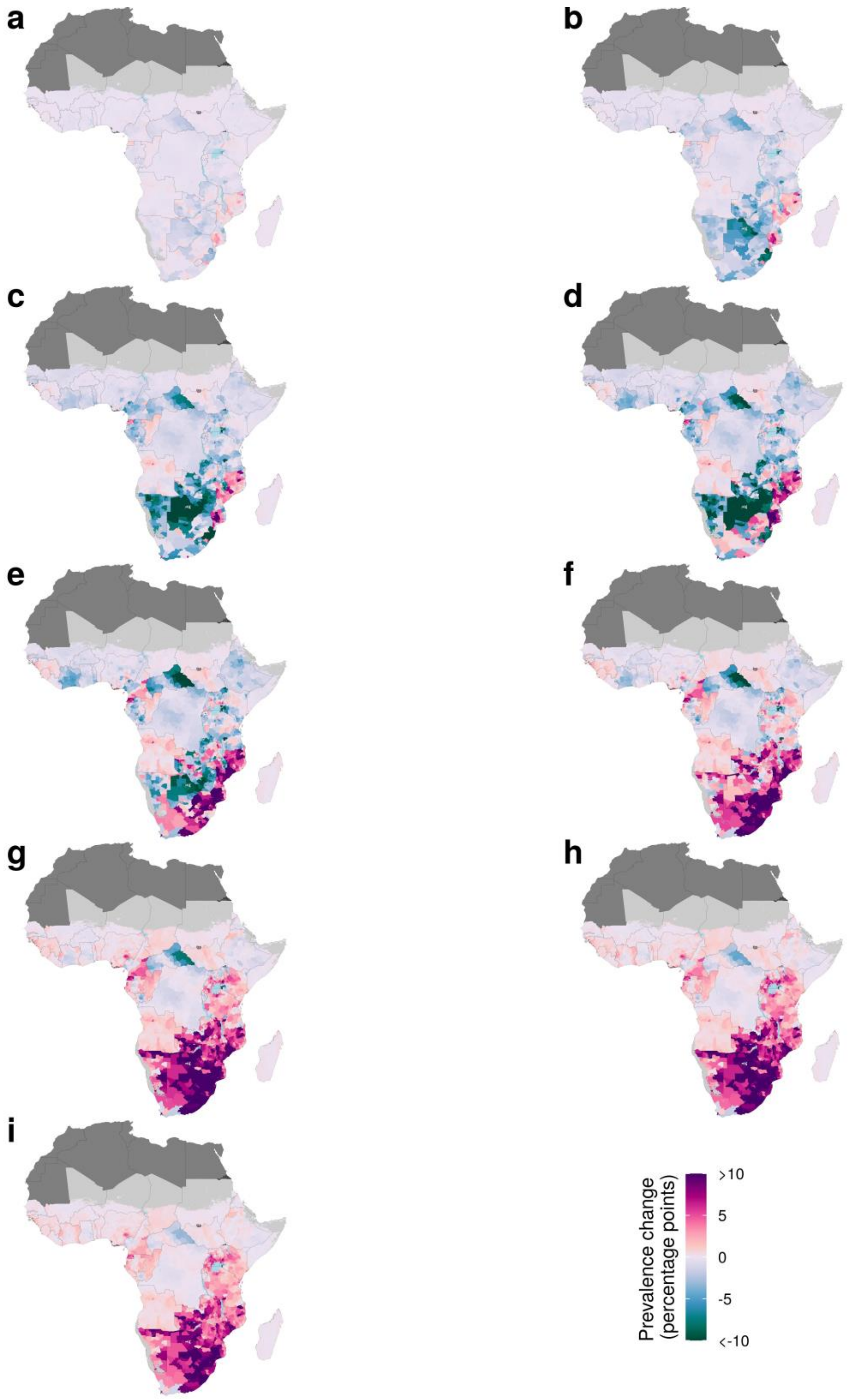

Figure S40: Change in HIV prevalence in females, 2010-2018. Absolute change in female HIV prevalence at the second administrative level for (a) ages 15–19 years; (b) ages 20-24 years; (c) ages 25-29 years; (d) ages 30-34 years; (e) ages 35-39 years; (f) ages 40-44 years; (g) ages 45-49 years; (h) ages 50-54 years; and (i) ages 55-59 years. Maps reflect national boundaries, land cover, lakes, and population; areas with fewer than ten people per 1 × 1 km, and classified as barren or sparsely vegetated, are colored light grey. Countries colored in dark grey were not included in the analysis.

Figure S41: Spatial mesh for geostatistical models

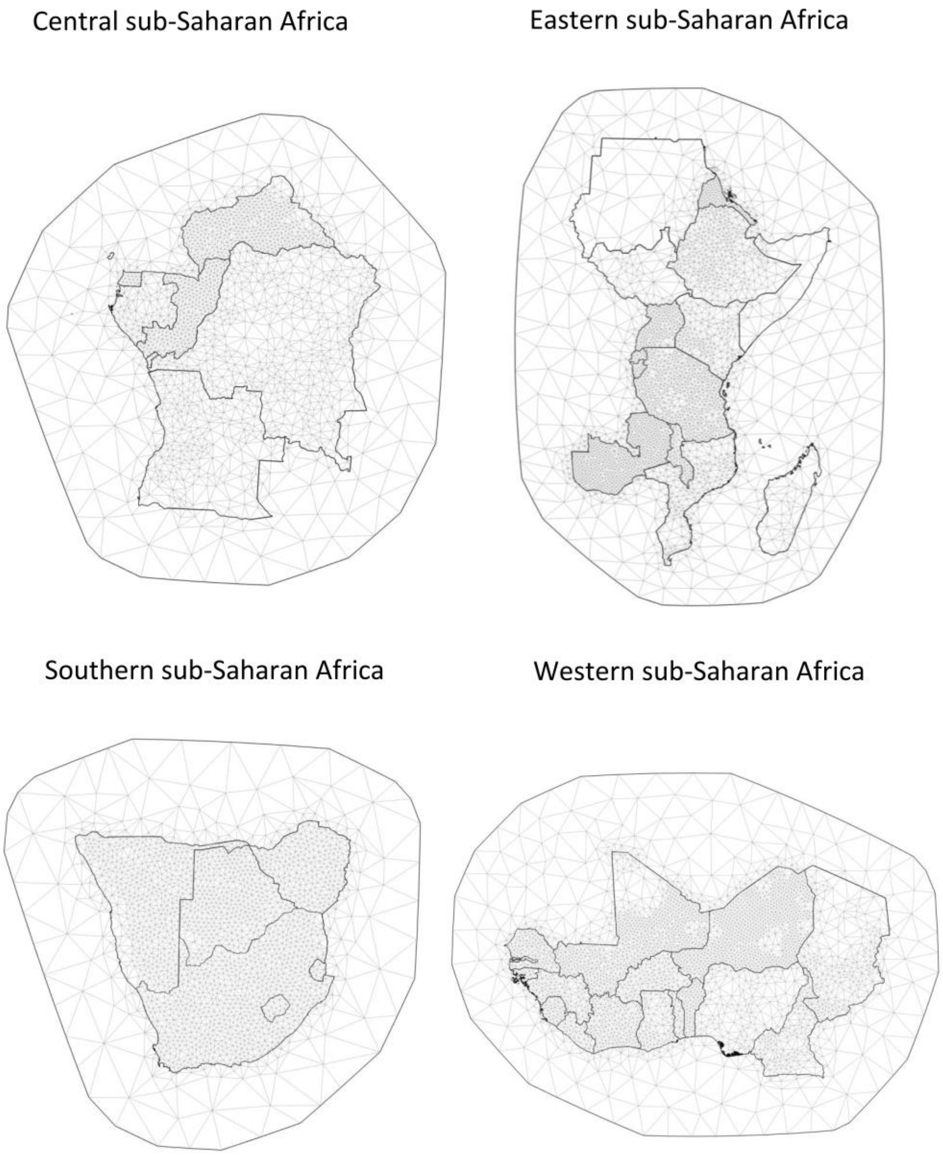

Figure S41: Space mesh for geostatistical models. The finite elements mesh used to fit the space-time correlated error for each region, overlaid on the countries in that region. Both the fine-scale mesh over land in the modeling region and the coarser buffer region mesh are shown.
